# Phosphorylation-Dependent Assembly of a 14-3-3 Mediated Signaling Complex during Red Blood Cell Invasion by Plasmodium falciparum Merozoites

**DOI:** 10.1128/mBio.01287-20

**Published:** 2020-08-18

**Authors:** Kunal R. More, Inderjeet Kaur, Quentin Giai Gianetto, Brandon M. Invergo, Thibault Chaze, Ravi Jain, Christéle Huon, Petra Gutenbrunner, Hendrik Weisser, Mariette Matondo, Jyoti S. Choudhary, Gordon Langsley, Shailja Singh, Chetan E. Chitnis

**Affiliations:** a Malaria Parasite Biology and Vaccines Unit, Department of Parasites and Insect Vectors, Institut Pasteur, Paris, France; b International Centre for Genetic Engineering and Biotechnology (ICGEB), New Delhi, India; c Proteomics Platform, Mass Spectrometry for Biology Unit, USR CNRS 2000, Institut Pasteur, Paris, France; d Bioinformatics and Biostatistics HUB, Computational Biology Department, USR CNRS 3756, Institut Pasteur, Paris, France; e EMBL-European Bioinformatics Institute, Wellcome Genome Campus, Hinxton, United Kingdom; f Proteomic Mass Spectrometry Group, Wellcome Trust Sanger Institute, Hinxton, United Kingdom; g Special Centre for Molecular Medicine, Jawaharlal Nehru University, New Delhi, India; h Institut Cochin, Inserm U1016, Paris, France; NIAID/NIH

**Keywords:** host cell invasion, host-parasite interaction, malaria, signaling

## Abstract

Red blood cell (RBC) invasion by *Plasmodium* merozoites requires multiple steps that are regulated by signaling pathways. Exposure of P. falciparum merozoites to the physiological signal of low K^+^, as found in blood plasma, leads to a rise in cytosolic Ca^2+^, which mediates microneme secretion, motility, and invasion. We have used global phosphoproteomic analysis of merozoites to identify signaling pathways that are activated during invasion. Using quantitative phosphoproteomics, we found 394 protein phosphorylation site changes in merozoites subjected to different ionic environments (high K^+^/low K^+^), 143 of which were Ca^2+^ dependent. These included a number of signaling proteins such as catalytic and regulatory subunits of protein kinase A (PfPKAc and PfPKAr) and calcium-dependent protein kinase 1 (PfCDPK1). Proteins of the 14-3-3 family interact with phosphorylated target proteins to assemble signaling complexes. Here, using coimmunoprecipitation and gel filtration chromatography, we demonstrate that Pf14-3-3I binds phosphorylated PfPKAr and PfCDPK1 to mediate the assembly of a multiprotein complex in P. falciparum merozoites. A phospho-peptide, P1, based on the Ca^2+^-dependent phosphosites of PKAr, binds Pf14-3-3I and disrupts assembly of the Pf14-3-3I-mediated multiprotein complex. Disruption of the multiprotein complex with P1 inhibits microneme secretion and RBC invasion. This study thus identifies a novel signaling complex that plays a key role in merozoite invasion of RBCs. Disruption of this signaling complex could serve as a novel approach to inhibit blood-stage growth of malaria parasites.

## Abstract (precis)

Invasion of red blood cells (RBCs) by Plasmodium falciparum merozoites is a complex process that is regulated by intricate signaling pathways. Here, we used phosphoproteomic profiling to identify the key proteins involved in signaling events during invasion. We found changes in the phosphorylation of various merozoite proteins, including multiple kinases previously implicated in the process of invasion. We also found that a phosphorylation-dependent multiprotein complex including signaling kinases assembles during the process of invasion. Disruption of this multiprotein complex impairs merozoite invasion of RBCs, providing a novel approach for the development of inhibitors to block the growth of blood-stage malaria parasites.

## INTRODUCTION

The clinical symptoms of malaria are attributed to the blood stage of the parasite life cycle during which Plasmodium falciparum merozoites invade and multiply within host red blood cells (RBCs). Following the development of mature schizonts, newly formed merozoites egress and invade uninfected RBCs to initiate a new cycle of infection. The invasion of RBCs by P. falciparum merozoites is a complex multistep process that is mediated by specific molecular interactions between parasite ligands and RBC receptors (1, 2). These parasite ligands are initially located in internal secretory organelles called micronemes and rhoptries. They are released to the merozoite surface in tightly regulated steps during RBC invasion (1, 2). Invasion is driven by the invading parasite and requires activation of the merozoite motility machinery (2). The signaling pathways that trigger apical organelle release and activate merozoite motility to enable host cell invasion are not completely understood.

Protein phosphorylation is known to be the primary regulator of biological signaling pathways (3). Protein phosphorylation/dephosphorylation acts as a molecular switch that can lead to diverse outcomes, including activation or deactivation of enzymes, preparation of proteins for degradation, translocation of proteins, and establishment of protein-protein interactions leading to the formation of functional multiprotein complexes (4). A number of kinases responsible for protein phosphorylation are activated by second messengers such as calcium (Ca^2+^) or cyclic nucleotides such as cAMP and cGMP. A rise in cytosolic levels of these second messengers in response to external stimuli can regulate the activity of Ca^2+^-dependent and cAMP/cGMP-dependent kinases that play a role in activating specific physiological responses. Examples of such kinases in malaria parasites include a family of P. falciparum calcium-dependent protein kinases (PfCDPKs) that are activated by a rise in cytosolic Ca^2+^ levels as well as protein kinase A (PKA) and protein kinase G (PKG), which are activated by the cyclic nucleotides, cAMP and cGMP, respectively (5, 6).

Exposure of P. falciparum merozoites to a low-potassium (K^+^) environment in plasma during blood-stage growth triggers a rise in Ca^2+^ and cAMP, which activates PfCDPK1 and PfPKA, respectively (5–8). Both PfCDPK1 and PfPKA have been shown to play roles in RBC invasion by P. falciparum merozoites (9–11). In addition, a rise in cGMP levels in merozoites activates PKG, which plays an essential role in merozoite egress (12). These kinases modulate phosphorylation of diverse target proteins to activate merozoite motility, as well as secretion of invasion-related proteins such as P. falciparum 175-kDa erythrocyte binding antigen (PfEBA175; PF3D7_0731500) and apical merozoite antigen-1 (PfAMA1; PF3D7_1133400) from the micronemes to the merozoite surface (6, 10–13). Following microneme secretion, the engagement of PfEBA175 with its receptor glycophorin A triggers another signaling cascade that leads to the release of rhoptry proteins such as reticulocyte binding protein homologue 2b (PfRH2b; PF3D7_1335300) (7). The secretion of microneme and rhoptry proteins seals the engagement of the merozoite with the RBC and enables completion of the invasion process.

The cross talk between signaling pathways activated by different second messengers and the effector kinases that they regulate to mediate apical organelle secretion and merozoite motility during host cell invasion are not fully understood. Phosphoproteome analysis can identify merozoite proteins that are targets for phosphorylation by kinases and provide information about the signaling pathways that are activated in a cell in response to external stimuli. Phosphorylation-dependent formation of multiprotein signaling complexes plays a key role in the regulation of diverse cellular processes (14–16). For example, in human cells, phosphorylation of membrane-associated guanylate kinase-like domain-containing protein (CARMA) by protein kinase C (PKC) leads to the formation of the CARMA1-Bcl10-MALT1 (CBM) complex, which activates the transcription factor NF-κB to regulate cell survival, activation, and proliferation (14). A family of scaffold proteins, referred to as the 14-3-3 family, binds phosphorylated proteins to assemble signaling complexes in diverse systems (15, 16). For example, in the brain, a 14-3-3ζ dimer simultaneously binds and bridges the cytoskeletal protein tau and glycogen synthase kinase, GSK3β, to stimulate tau phosphorylation, which in turn, regulates microtubule dynamics (15). In the case of Arabidopsis thaliana, calcium-dependent phosphorylation of a basic region/leucine-zipper (bZIP) transcription factor FD leads to the formation of a florigen complex with flowering locus T protein that is mediated by 14-3-3 and regulates flowering (16). Phosphoproteomic analysis of P. falciparum schizonts also reported the formation of a phosphorylation-dependent high-molecular-weight protein complex involving calcium-dependent protein kinase-1 (PfCDPK1, PF3D7_0217500) (13), although the precise composition of the complex was not defined.

In this study, we present a phospho-protein profile of P. falciparum merozoites and identify signal-dependent phosphorylation events that play important roles in the RBC invasion process. Importantly, we describe the formation of a dynamic, phosphorylation-dependent, high-molecular-weight complex involving PfCDPK1 and PfPKAr (PF3D7_1223100) and define the role of Pf14-3-3I (PF3D7_0818200) in the assembly of this complex. Disruption of this Pf14-3-3-mediated protein complex with a peptide mimic inhibits RBC invasion by merozoites, providing a novel strategy to block blood-stage growth of malaria parasites.

## RESULTS

### Phosphoproteome analysis of P. falciparum merozoites.

**FIG 1:**
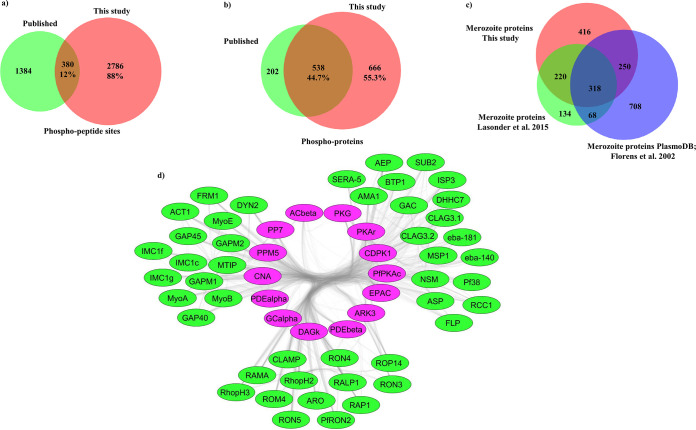
The phosphoproteome of P. falciparum merozoites (a) Venn diagram depicting overlap in phosphorylation sites between the published merozoite phosphoproteome (17) and this study. (b) Venn diagram depicting overlap in phosphoproteins between the published merozoite phosphoproteome (17) and this study. (c) Venn diagram showing overlap between merozoite proteins detected in a published merozoite phosphoproteome (17), published merozoite proteomes (18, 19), and this study. (d) Signaling- and invasion-related proteins from clustered subnetwork 1 identified from the merozoite interaction network of phosphorylated proteins using the MCODE clustering algorithm. Protein interaction data were downloaded from STRING for phosphoproteins found in this study and visualized in Cytoscape. The MCODE clustering algorithm was used for the generation of the subnetwork of highly interacting proteins. Each cluster was curated for Gene Ontology (GO) terms using PlasmoDB to identify overrepresented molecular function categories. The interaction network of proteins corresponding to the molecular function categories “invasion of host cells” and “signaling related proteins” from MCODE cluster 1 is shown here. Invasion-related proteins are in green, while signaling-related proteins are in pink.

Merozoites released from synchronized P. falciparum schizonts were purified as described previously (7) and processed for mass-spectrometric phosphoproteome analysis. The workflow used for phosphoproteomics and data analysis is outlined in Fig. S1 in the supplemental material. Data set S1 provides the list of phosphorylated proteins and phosphosites identified in P. falciparum merozoites. It includes identification of 3,700 phosphorylation sites on 3,166 phosphopeptides from 1,204 distinct P. falciparum proteins with phosphorylation site localization probabilities of >0.75 for MaxQuant and phosphoscore of >11 for OpenMS pipeline (Fig. 1a, Data set S1). Comparison with the only other published P. falciparum merozoite phosphoproteome (17) identified 2,786 phosphosites and 666 merozoite phosphoproteins that were unique to our study, whereas 380 phosphosites and 538 phosphoproteins were common (Fig. 1a and b). A comparison of the proteins in the merozoite phosphoproteome from this study with previously described merozoite proteomes (18, 19) reveals a greater than 47% overlap (Fig. 1c). Comparison of proteins in the merozoite phosphoproteome from Lasonder et al. (17) with previously described merozoite proteomes (18, 19) showed that 52% of proteins were common (Fig. 1c), which is similar to the overlap observed in our study. Differences in proteins detected in merozoite proteome/phosphoproteome data sets are common and can be attributed to differences in experimental and analytical methods used in these studies. Differences in phosphosite identification between studies might also be due to differences in experimental conditions, as well as differences in methods used for merozoite isolation, phosphopeptide enrichment, and data analysis. This study expands significantly the repertoire of phosphorylated P. falciparum merozoite proteins.

10.1128/mBio.01287-20.1 FIG S1 Schematic representation of workflows for phosphoproteomics, quantitative phosphoproteomics, and data analysis. (a) Workflow for total phosphoproteomic analysis of P. falciparum merozoites. The steps to identify phosphopeptides in tryptic digests of proteins derived from merozoites are shown. After digestion with trypsin, peptides were fractionated by strong cation exchange (SCX) chromatography, enriched for phosphopeptides, first by Fe^3+^-based immobilized metal affinity chromatography (IMAC) and then by enrichment of phosphopeptides on TiO2/ZrO2 tips and analyzed by LC-MS/MS. (b) Workflow for phosphoproteomic data analyses. All the raw files were converted to MZML file format using MSConvertGUI and analyzed with the OpenMS pipeline using the Mascot and MSGF+ algorithms followed by postprocessing with Percolator, as well as the MaxQuant pipeline for peptide and protein assignment. Peptides and proteins commonly identified by these pipelines were used for further bioinformatics analyses, including gene ontology, identification of protein interaction networks, and motif analysis. (c) Workflow for differential quantitative phosphoproteomic analysis of P. falciparum merozoites in different ionic conditions. Isolated free merozoites were resuspended in high-K^+^-containing buffer mimicking intracellular ionic conditions (IC), low-K^+^-containing buffer mimicking extracellular ionic conditions (EC), and EC buffer supplemented with the intracellular Ca^2+^ chelator, BAPTA-AM. Proteins were isolated and digested with trypsin to generate peptides. Peptides from different treatment conditions were labeled separately with tandem mass tags (TMT) (IC, 128; EC, 129; EC-BA, 130) and combined. TMT-labeled peptides were fractionated using hydrophilic interaction liquid chromatography (HILIC), enriched for phosphopeptides, first by Fe^3+^-based IMAC and then by enrichment of phosphopeptides on TiO_2_/ZrO_2_ tips, and analyzed by LC-MS/MS. Download FIG S1, PDF file, 0.1 MB. Copyright © 2020 More et al. 2020 More et al. This content is distributed under the terms of the Creative Commons Attribution 4.0 International license.

10.1128/mBio.01287-20.6 DATA SET S1 Summary of phosphopeptides and phosphosites identified in the P. falciparum merozoite phosphoproteome. Download Data Set S1, XLSX file, 0.6 MB. Copyright © 2020 More et al. 2020 More et al. This content is distributed under the terms of the Creative Commons Attribution 4.0 International license.

To identify potential interactions in the merozoite phosphoproteome, we mapped the phosphoproteins to predicted protein-protein functional associations from the STRING database (20) and constructed a protein interaction network. The interactome is composed of 1,201 nodes/proteins covering 99% of the phosphoproteome and 28,534 edges, which correspond to predicted protein-protein interactions (Fig. S2). This interaction network was analyzed for the presence of densely connected subnetworks using the MCODE clustering algorithm (21), which identified 17 subnetworks representing 50% (600) of proteins (Data set S2). Gene ontology analysis of the MCODE clusters was performed using the PlasmoDB database (Data set S2). Enriched in MCODE cluster 1 are 132 proteins relevant to host cell invasion (Fig. 1d). The phosphorylated proteins in MCODE cluster 1 include signaling-related proteins such as protein kinase G (PfPKG; PF3D7_1436600), guanylate cyclase (PfGC; PF3D7_1138400), protein kinase A regulatory subunit (PfPKAr; PF3D7_1223100), protein kinase A catalytic subunit (PfPKAc; PF3D7_0934800), calcium-dependent protein kinase 1 (PfCDPK1; PF3D7_0217500) and calcium-dependent protein phosphatase calcineurin (PfCNA; PF3D7_0802800), as well as invasion-related parasite proteins such as merozoite surface protein-1 (MSP1; PF3D7_0930300), erythrocyte binding antigens (EBA181, PF3D7_0102500; EBA140, PF3D7_1301600), apical merozoite antigen 1 (AMA1), rhoptry neck proteins (RON2, PF3D7_1452000; RON3, PF3D7_1252100; RON4, PF3D7_1116000), and parasite proteins responsible for motility, such as inner membrane complex proteins PfIMC1c (PF3D7_1003600) and PfIMC1g (PF3D7_0525800), GAP45 (PF3D7_1222700), GAP40 (PF3D7_0515700), MyoA (PF3D7_1342600), MyoB (PF3D7_0503600), and MTIP (PF3D7_1246400) (Fig. 1d). The presence of both calcium and cyclic nucleotide responsive effectors in MCODE cluster 1 (Fig. 1d) indicates significant cross talk between these second messengers at the time of invasion.

10.1128/mBio.01287-20.2 FIG S2 The predicted protein-protein interaction network of P. falciparum merozoites. Protein interaction data downloaded from STRING for phosphoproteins found in this study and visualized in Cytoscape. The MCODE clustering algorithm was used for the generation of the subnetwork of highly interacting proteins. MCODE clusters are circled, numbered accordingly, and shown in specific colors to differentiate them from nonclustered proteins that are shown in the middle of the network. Download FIG S2, PDF file, 2.6 MB. Copyright © 2020 More et al. 2020 More et al. This content is distributed under the terms of the Creative Commons Attribution 4.0 International license.

10.1128/mBio.01287-20.7 DATA SET S2 Gene Ontology enrichment results for MCODE clusters. Download Data Set S2, XLSX file, 0.1 MB. Copyright © 2020 More et al. 2020 More et al. This content is distributed under the terms of the Creative Commons Attribution 4.0 International license.

### Exposure of P. falciparum merozoites to an ionic environment mimicking blood plasma induces changes in protein phosphorylation.

We showed previously that merozoites respond to changes in their ionic environment, especially changes in potassium ion (K^+^) concentration (7, 8). Exposure of merozoites to a low K^+^ environment, which is characteristic of extracellular ionic conditions in blood plasma, serves as a signal to trigger a rise in Ca^2+^ and cAMP to activate signaling cascades (7, 8). We performed quantitative phosphoproteomics on merozoites resuspended in buffers mimicking intracellular and extracellular ionic conditions (IC buffer and EC buffer) to identify differences in protein phosphorylation. This resulted in the identification of 1,499 unique phosphosites corresponding to 587 P. falciparum proteins (Data set S3). Ca^2+^-dependent changes in phosphorylation were identified by studying differences in phosphorylation of merozoite proteins in intracellular (IC) buffer compared to either extracellular (EC) buffer or EC buffer plus BAPTA-AM (EC-BA) (Data set S3).

10.1128/mBio.01287-20.8 DATA SET S3 Comparative changes in the phosphorylation of P. falciparum merozoite phosphopeptides. Download Data Set S3, XLSX file, 0.2 MB. Copyright © 2020 More et al. 2020 More et al. This content is distributed under the terms of the Creative Commons Attribution 4.0 International license.

**FIG 2:**
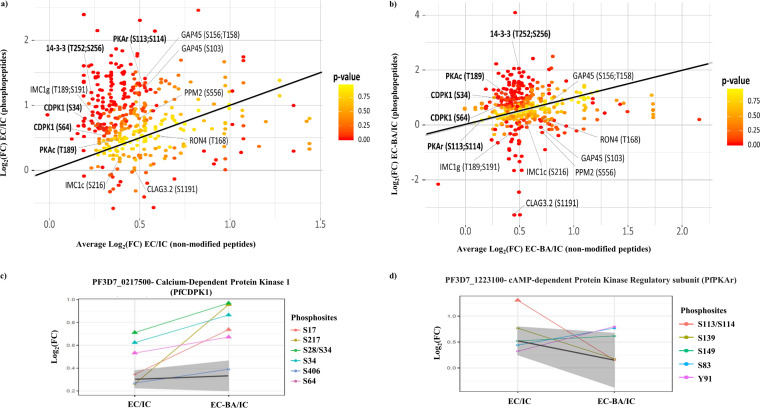
Signal-dependent changes in the phosphoproteome of P. falciparum merozoites. (a) Fold changes in abundance of phosphopeptides in merozoites in EC buffer with low K^+^ compared to IC buffer with high K^+^ were plotted against fold changes of nonphosphopeptides. (b) Fold changes in phosphopeptides in merozoites in EC + BAPTA-AM (EC-BA) buffer compared to IC buffer plotted against fold changes of nonphosphopeptides. *P* values for fold change in phosphorylation (color coded) were calculated compared to the changes in nonphosphorylated peptides for respective proteins. Key proteins and their phosphosites with significant alterations are shown. (c) Fold changes in individual phosphorylations in EC buffer compared with IC and EC-BA buffer compared with IC buffer for PfCDPK1. The gray area represents the fold change in abundance of nonphosphorylated peptides from the corresponding proteins. Phosphosites with significant changes in abundance lie outside the gray area and are denoted with triangles. Phosphorylation of Ser 28/Ser 34 and Ser 64 of PfCDPK1 was significantly higher in EC and EC-BA buffer than in IC buffer. (d) Fold changes in individual phosphorylations in EC buffer compared with IC and EC-BA buffer compared with IC buffer for PfPKAr. The gray area represents the fold change in abundance of nonphosphorylated peptides from the corresponding proteins. Phosphosites with significant changes in abundance lie outside the gray area and are denoted with triangles. Phosphorylation of PfPKAr on Ser 113/Ser 114 was significantly higher in EC buffer, but not EC-BA buffer, compared to IC buffer.

Proteins exhibiting statistically significant fold changes in phosphorylation at specific amino acid residues in merozoites in EC buffer compared to IC buffer and in EC-BA buffer compared to IC buffer were identified. Peptides from the same proteins without any phosphorylation were quantified and used to normalize for differences in concentration of proteins in merozoite samples under different conditions. Based on results of two independent biological replicates with each replicate analyzed two times using mass spectrometry, we identified 394 phosphoresidues as significantly altered when merozoites are exposed to EC buffer compared to IC buffer. Of these, phosphorylation at 143 sites is blocked by the Ca^2+^ chelator BAPTA-AM (Data sets S3 and S4). Changes in phosphorylation of some key signaling related proteins, such as PfPKAr, PfCDPK1, and Pf14-3-3I were observed (Fig. 2a and b). Phosphorylation of PfCDPK1 on Ser 28/34 and Ser 64 was significantly upregulated in merozoites exposed to EC buffer compared to IC buffer (Fig. 2a and c). Chelation of Ca^2+^ with BAPTA-AM had no effect on these phosphorylation events (Fig. 2b and c). Phosphorylation on Ser 17 and Ser 217 of PfCDPK1 was found to be higher in merozoites in EC-BA buffer than those in IC buffer. However, there was no increase in phosphorylation of Ser 17 and Ser 217 in EC buffer compared with IC buffer (Fig. 2c). In contrast, phosphorylation of PfPKAr in EC buffer at Ser 113/Ser 114 was dependent on the presence of Ca^2+^ (Fig. 2d). Corresponding spectra and quantification profiles for phosphorylation of PfCDPK1 on Ser 28/34 and of PfPKAr on Ser 113/Ser 114 are shown in Fig. S3. PfCDPK1 and PfPKAr are known to be involved in RBC invasion by merozoites (10, 11). We therefore further investigated the relevance of changes in their phosphorylation status to the process of invasion.

10.1128/mBio.01287-20.3 FIG S3 Representative spectra and quantification profile. (a) Representative spectra demonstrating phosphorylation of Ser28 and Ser34 in PfCDPK1. (b) Representative spectra demonstrating phosphorylation on PfPKAr at Ser 113 and Ser 114. The relative abundance of reporter ions from two experiments is also shown. Peptides were labeled with TMT mass tags 128 (IC), 129 (EC), and 130 (EC-BA). Download FIG S3, PDF file, 0.6 MB. Copyright © 2020 More et al. 2020 More et al. This content is distributed under the terms of the Creative Commons Attribution 4.0 International license.

The changes in phosphorylation of key signaling proteins, PfCDPK1 and PfPKAr, in EC buffer compared to IC buffer were also confirmed using antiphosphoserine antibodies. Lysates of P. falciparum merozoites in IC and EC buffers were used for immunoprecipitation (IP) with anti-PfCDPK1 and anti-PfPKAr sera. The IPs were separated by SDS-PAGE, and PfCDPK1 and PfPKAr were detected by Western blotting. Similar levels of PfCDPK1 and PfPKAr were found in IC and EC conditions (Fig. S4a). The blots were also probed with antiphosphoserine antibodies to determine the levels of serine phosphorylation in these proteins (Fig. S4a). Western blotting with antiphosphoserine antibodies confirmed that the levels of phosphorylated serines in PfCDPK1 and PfPKAr were higher in EC buffer than those in IC buffer (Fig. S4a). Moreover, each IP sample in EC buffer showed multiple proteins with increased serine phosphorylation compared to IC buffer (Fig. S4a), suggesting that these phosphorylated proteins may interact with each other to form a multiprotein complex.

10.1128/mBio.01287-20.4 FIG S4 **(**a) Level of phosphorylation of serines in P. falciparum merozoite signaling proteins in response to exposure to an extracellular ionic environment with low K^+^. Lysates of merozoites in intracellular buffer (IC) and extracellular buffer (EC) were used for immunoprecipitation with anti-PfCDPK1 and anti-PfPKAr sera. The imunoprecipitates (IP) were probed with anti-phospho-serine antibodies as well as anti-PfCDPK1 and anti-PfPKAr sera. PfCDPK1 and PfPKAr have higher levels of serine phosphorylation in EC than in IC buffer (black boxes). (b) Detection P. falciparum protein kinase G (PfPKG) in immunoprecipitates with anti-PfCDPK1, anti-PfPKAr, and anti-Pf14-3-3I sera. Merozoite lysates were immunoprecipitated (IP) with anti-PfCDPK1, anti-PfPKAr, and anti-Pf14-3-3I sera and probed for the presence of PfPKG by Western blotting. PfPKG was detected in the merozoite lysate supernatant (S) but not in the IP pellets (P). Download FIG S4, PDF file, 0.8 MB. Copyright © 2020 More et al. 2020 More et al. This content is distributed under the terms of the Creative Commons Attribution 4.0 International license.

### Formation of a multiprotein complex involving PfPKAr, PfCDPK1, and Pf14-3-3I in P. falciparum merozoites.

To investigate the interactions of signaling proteins, PfPKAr and PfCDPK1, in merozoites, we immunoprecipitated PfPKAr and PfCDPK1 from merozoite lysates using specific polyclonal sera and identified interacting proteins in the IPs by mass spectrometry. The presence of PfPKAr in IPs with anti-PfCDPK1 sera was confirmed by detection of multiple PfPKAr peptides with greater than 50% sequence coverage (Table 1). Similarly, the presence of PfCDPK1 is confirmed in IPs performed with anti-PfPKAr sera. Multiple PfCDPK1 peptides, with greater than 50% sequence coverage, are detected in IPs performed with anti-PfPKAr sera (Table 1). In addition, the scaffold protein, Pf14-3-3I, is also detected in IPs performed with both anti-PfCDPK1 and anti-PfPKAr sera with multiple Pf14-3-3I peptides detected that provide greater than 50% sequence coverage. The presence of Pf14-3-3I is further confirmed by detection of PfCDPK1 and PfPKAr peptides with greater than 50% sequence coverage in IPs performed with anti-Pf14-3-3I sera (Table 1). These studies suggest that Pf14-3-3I, PfCDPK1 and PfPKAr interact to form a multiprotein complex in P. falciparum merozoites (Table 1). In addition to PfPKAr, PfCDPK1, and Pf14-3-3I, four other proteins (elongation factor 1α [EF 1α; PF3D7_1357000], glyceraldehyde-3-phosphate dehydrogenase [GAPDH; PF3D7_1462800], phosphoethanolamine *N*-methyltransferase [PMT; PF3D7_1343000], and actin-depolymerizing factor 1 [ADF1; PF3D7_0503400]) were detected at similar stringency levels in IPs with all three sera, (anti-PfPKAr, anti-PfCDPK1, and anti-Pf14-3-3I sera) (Data set S5). PfPKAr is known to interact with PfPKAc, the PKA catalytic domain. PfPKAc peptides were also detected in pull downs with anti-PfPKAr, anti-PfCDPK1, and anti-Pf14-3-3I sera (Table 1). A number of other proteins are detected by mass spectrometry at low stringency in the IPs (Data set S5). Interestingly, PfPKG, which has been shown to play a role in merozoite egress, was not detected by mass spectrometry in IPs with anti-PfPKAr, anti-PfCDPK1, and anti-Pf14-3-3 sera. PfPKG was also not detected by Western blotting in IPs of merozoite lysates performed with anti-PfPKAr, anti-PfCDPK1, and anti-Pf14-3-3I sera (Fig. S4b), confirming the absence of PfPKG in the multiprotein complex.

**TABLE 1:** Identification of PfCDPK1, PfPKAr, Pf14-3-3I and PfPKAc by mass spectrometry^*a*^

Protein detected in IP		MaxQuant score	IP with anti-PfPKAr		IP with anti-PfCDPK1		IP with anti-Pf14-3-3I	
Protein ID	Protein name		No. of unique peptides	Sequence coverage (%)	No. of unique peptides	Sequence coverage (%)	No. of unique peptides	Sequence coverage (%)
PF3D7_0217500	PfCDPK1	323.31	39	62	57	66.6	43	66.4
PF3D7_1223100	PfPKAr	323.31	36	69.2	49	74.6	34	71.4
PF3D7_0818200	Pf14-3-3I	323.31	20	67.2	19	58.4	20	74
PF3D7_0934800	PfPKAc	53.62	7	21.6	4	9.9	5	15.8

a The complete list of proteins identified in the immunoprecipitates is reported in Data set S5.

10.1128/mBio.01287-20.9 DATA SET S4 Graphical representation of changes in phosphorylation of P. falciparum merozoite phosphopeptides. Download Data Set S4, PDF file, 0.2 MB. Copyright © 2020 More et al. 2020 More et al. This content is distributed under the terms of the Creative Commons Attribution 4.0 International license.

10.1128/mBio.01287-20.10 DATA SET S5 Identification of proteins in immunoprecipitates of P. falciparum merozoite lysates made with anti-Pf14-3-3I, anti-PfCDPK1, or anti-PfPKAr sera. Download Data Set S5, XLSX file, 0.1 MB. Copyright © 2020 More et al. 2020 More et al. This content is distributed under the terms of the Creative Commons Attribution 4.0 International license.

**FIG 3:**
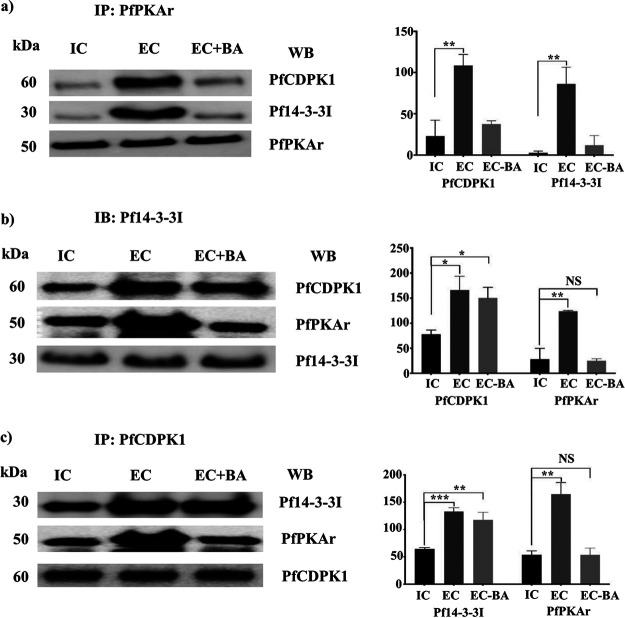
Calcium-dependent interaction of PfPKAr with PfCDPK1 and Pf14-3-3I leads to formation of a multiprotein complex in P. falciparum merozoites. (a) PfPKAr was immunoprecipitated (IP) from merozoites in IC buffer mimicking intracellular ionic conditions with high K^+^ (IC), EC buffer mimicking extracellular ionic conditions with low K^+^, or EC buffer with intracellular Ca^2+^ chelator BAPTA-AM (EC-BA). The presence of PfCDPK1, Pf14-3-3I, and PfPKAr in IPs was confirmed by Western blotting (WB). Graphs show the average intensity normalized with the protein immunoprecipitated for each condition from three independent experiments. A representative Western blotting image from one of three independent experiments is shown. (b and c) Representative Western blots (WB) performed with specific antisera for PfCDPK1, PfPKAr, and Pf14-3-3I on IPs with anti-Pf14-3-3I and anti-PfCDPK1 sera, respectively. Quantification of interaction partners (mean ± SEM) from 3 independent experiments (*n* = 3) is shown in bar graphs. *, *P* < 0.05; **, *P* < 0.005; ***, *P* < 0.0005; by *t* test. NS, nonsignificant, *P* > 0.05.

The IPs described above were carried out with lysates made from merozoites resuspended in RPMI 1640, which has low K^+^ levels. Next, we investigated if the interactions of Pf14-3-3I, PfPKAr, and PfCDPK1 are dynamic, if they depend on the external ionic environment of merozoites, and if intracellular Ca^2+^ plays a role in these interactions. Lysates of merozoites resuspended in IC, EC, and EC-BA buffers were used for IP with specific sera against PfCDPK1, PfPKAr, and Pf14-3-3I. The IP eluates were probed for the presence of interacting partners. PfCDPK1 and Pf14-3-3I were detected in IPs generated using specific anti-PfPKAr sera with lysates prepared from merozoites in EC buffer (Fig. 3a). In contrast, the amounts of PfCDPK1 and Pf14-3-3I in IP eluates with anti-PfPKAr sera were significantly lower in lysates prepared from merozoites in IC and EC-BA buffers (Fig. 3a). The interaction of PfPKAr with PfCDPK1 and Pf14-3-3I is thus favored when merozoites are exposed to a low-K^+^ environment. Moreover, the reduced signal in IPs in the case of merozoites in EC-BA buffer indicates that this interaction requires Ca^2+^. However, the interaction between Pf14-3-3I and PfCDPK1 is not dependent on the presence of Ca^2+^ (Fig. 3b and c). Collectively, these observations suggest that PfPKAr, PfCDPK1, and Pf14-3-3I form a multiprotein complex when merozoites are exposed to a low-K^+^ environment. The interaction of PfPKAr with the multiprotein complex is dependent on the presence of intracellular Ca^2+^, whereas the interaction of PfCDPK1 is independent of Ca^2+^.

**FIG 4:**
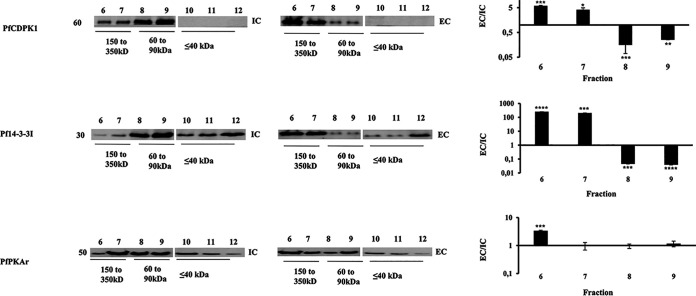
Analysis of assembly of multiprotein complexes with PfPKAr, PfCDPK1. and Pf14-3-3 by gel filtration chromatography. Lysates were prepared from merozoites in IC and EC buffers and fractionated using a Superdex 200 gel filtration column. Fractions were probed for the presence of PfCDPK1, Pf14-3-3I, and PfPKAr by Western blotting. Representative Western blots are shown for one out of three independent experiments. The presence of proteins in fractions corresponding to high-molecular-weight proteins (lanes 6 to 7 corresponding to >150 kDa and lanes 8 to 9 corresponding to 60 to 90 kDa) was reproducible across 3 independent experiments. Intensity for each lane (lanes 6 to 9) was measured using ImageJ software, and the ratio of EC/IC was calculated. The graph represents the average of the EC/IC ratio for each protein from three independent experiments. Mean ± SEM is shown for 3 independent experiments (*n* = 3). *, *P* < 0.05; **, *P* < 0.005; ***, *P* < 0.0005; by *t* test.

Size exclusion chromatography was also used to detect the presence of the multiprotein complex in merozoite lysates. When lysates were prepared from merozoites treated with IC buffer, PfPKAr, PfCDPK1, and Pf14-3-3I primarily migrated at positions reflecting their monomeric or dimeric sizes (Fig. 4). Some Pf14-3-3 and PfPKAr proteins were found in the higher-molecular-weight fractions in IC buffer, as 14-3-3 can exist as a homodimer (22) and PfPKAr interacts with PfPKAc. In contrast, when lysates were prepared from merozoites in EC buffer, PfPKAr, PfCDPK1, and Pf14-3-3I were primarily present in a high-molecular-weight complex migrating between 150 to 250 kDa (Fig. 4). Assembly of the PfPKAr, PfCDPK1, and Pf14-3-3I complex in merozoites is thus dynamic in nature and assembles in merozoites exposed to a low-K^+^ ionic environment.

### Recombinant Pf14-3-3I binds specifically to a phosphopeptide based on PfPKAr.

**FIG 5:**
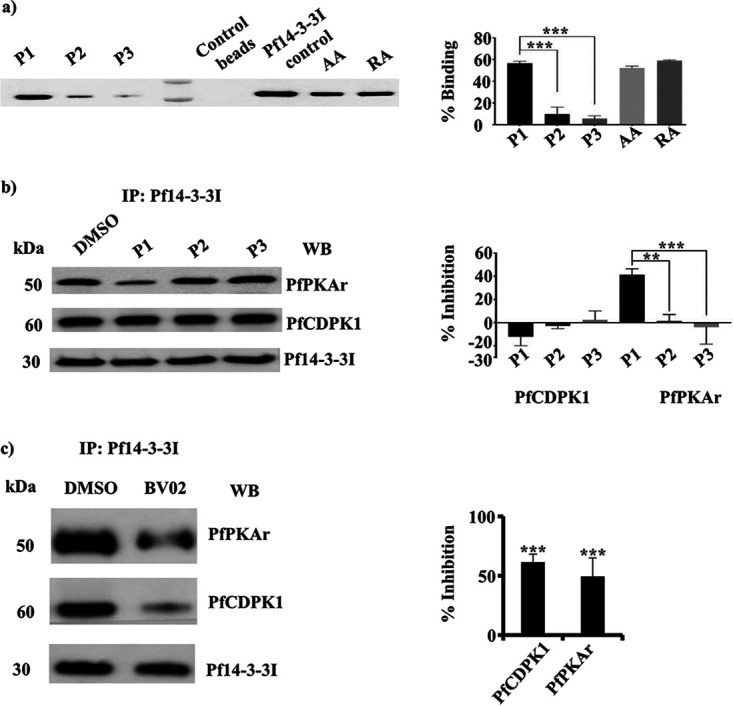
Inhibition of interaction between PfPKAr and Pf14-3-3I by a PfPKAr-derived phosphopeptide, P1, and small-molecule inhibitor, BV02, of Pf14-3-3I. (a) Binding of Pf14-3-3I with phosphopeptide P1 from PfPKAr. Phosphopeptide P1 (NDDGpSpSDG) based on the amino acid sequence of calcium-dependent phosphosites on PfPKAr encompassing Ser 113 and Ser 114, nonphosphorylated control peptide P2 (NDDGSSDG), and peptide P3 (pSDNGpSGDD), a control phosphopeptide based on scrambled P1 sequence, were tested for binding to Pf14-3-3I. Peptides P1, P2, and P3 were immobilized on agarose beads and allowed to interact with recombinant Pf14-3-3I. Bound recombinant Pf14-3-3I was detected in eluates by Western blotting. Phosphopeptides AA and RA, which are known to bind 14-3-3 were used as positive controls. Control beads with no immobilized peptides were used as a negative control. Plots show the average percentage binding calculated for binding of recombinant Pf14-3-3I to each peptide from 2 independent experiments (*n* = 2). A representative Western blotting image from one out of 2 independent experiments is shown. b) Peptide P1 inhibits binding of Pf14-3-3I to PfPKAr in merozoites. Merozoites were treated with 100 μM P1, P2, P3, or RPMI. Merozoites were lysed, and lysates were used for immunoprecipitation (IP) with anti-Pf14-3-3I sera. The presence of PfCDPK1 and PfPKAr in the immunoprecipitates was investigated using Western blotting. The percent inhibition of binding of PfCDPK1 and PfPKAr with Pf14-3-3I was calculated. The plot shows the average percent inhibition of binding for two independent experiments (*n* = 2). P1 decreases binding of PfPKAr to Pf14-3-3I but has no effect on PfCDPK1 binding to Pf14-3-3I. (c) BV02, a small-molecule inhibitor of 14-3-3 interactions with phosphopeptides, inhibits binding of Pf14-3-3I to PfCDPK1 and PfPKAr in merozoites. Merozoites were treated with 2 μM BV02 or DMSO and lysed, and lysates were used for immunoprecipitation with specific anti-Pf14-3-3I serum (IP; Pf14-3-3I). The presence of PfCDPK1 and PfPKAr in the immunoprecipitates was confirmed using Western blotting (WB). The plot shows the mean percent inhibition of binding (± SEM) for three independent experiments (*n* = 3). *, *P* < 0.05; **, *P* < 0.005; ***, *P* < 0.0005; by *t* test. NS, not significant (*P* > 0.05).

Phosphorylation of PfPKAr at Ser 113 and Ser 114 following exposure of merozoites to EC buffer is dynamic and depends on intracellular Ca^2+^ levels (Fig. 2). The phosphorylation of PfPKAr and the interaction between PfPKAr and Pf14-3-3I are both dependent on the presence of Ca^2+^ (Fig. 2 and 3). As 14-3-3 family proteins are phospho-recognition scaffold proteins that participate in the formation of multiprotein complexes (15, 22), we hypothesized that interaction between Pf14-3-3I and PfPKAr requires Ca^2+^-dependent phosphorylation of PfPKAr at Ser 113 and Ser 114. To test this hypothesis, we synthesized three peptides, phosphopeptide P1 spanning the sequence of phosphorylated Ser 113 and Ser 114 (P1: NDDGpSpSDG), a nonphosphorylated peptide (P2: NDDGSSDG), and a scrambled phosphorylated P1 peptide with a random distribution of the phospho-Ser residues (P3: pSDNGpSGDD). These peptides were immobilized on agarose beads and incubated with recombinant glutathione *S*-transferase (GST)-tagged Pf14-3-3I protein. There was significant binding of Pf14-3-3I-GST to beads coated with synthetic peptide P1 but none or only marginal binding to beads coated with synthetic peptides P2 and P3 (Fig. 5a). Known 14-3-3 binding peptides AA (ARSHpSYPA) and RA (RLYHpSLPA) based on canonical 14-3-3 binding motifs (15) also showed binding to Pf14-3-3I-GST similar to peptide P1 in control experiments (Fig. 5a).

### Phosphopeptide from PfPKAr specifically inhibits interaction between Pf14-3-3I and PfPKAr in merozoites.

Given that phosphopeptide P1 can bind to Pf14-3-3I *in vitro*, we next tested if P1 can inhibit binding of Pf14-3-3I to PfPKAr to prevent multiprotein complex formation in merozoites. We first confirmed that peptide P1 can enter P. falciparum merozoites. Peptide P1 tagged with fluorophore fluorescein isothiocyanate (FITC) was incubated with merozoites and tested for uptake by detecting the internalized peptide by fluorimetry (Fig. S5). Uptake of P1-FITC was observed at concentrations above 25 μM (Fig. S5). Merozoites were incubated with peptides P1, P2, and P3 at 100 μM. Subsequently, merozoite lysates were used for IP with anti-Pf14-3-3I antisera. PfPKAr and PfCDPK1 were detected in the IPs by Western blotting. Phosphorylated peptide P1 inhibited the interaction between Pf14-3-3I and PfPKAr, while peptides P2 and P3 had no effect (Fig. 5b). Interestingly, none of the peptides (P1, P2, and P3) had any effect on the interaction between Pf14-3-3I and PfCDPK1. The small-molecule inhibitor BV02, which blocks binding of mammalian 14-3-3 to its phosphorylated target proteins (23, 24), inhibited binding of both PfPKAr and PfCDPK1 with Pf14-3-3I (Fig. 5c).

10.1128/mBio.01287-20.5 FIG S5 Entry of peptide P1 in P. falciparum schizonts and merozoites. Peptide P1, an 8-amino acid peptide based on the PfPKAr sequence spanning Ser 113 and Ser 114 was conjugated to fluorescein isothiocyanate (FITC) and incubated with P. falciparum cultures with late-stage schizonts for 10 min. Parasites were washed with RPMI1640, and uptake of Peptide P1-FITC was measured by fluorimetry and observed by fluorescence microscopy. Significant uptake was observed in late-stage schizonts and merozoites at concentrations above 25 μM. Download FIG S5, PDF file, 0.2 MB. Copyright © 2020 More et al. 2020 More et al. This content is distributed under the terms of the Creative Commons Attribution 4.0 International license.

### Blocking Pf14-3-3I interactions inhibits merozoite invasion of RBCs and microneme secretion.

**FIG 6:**
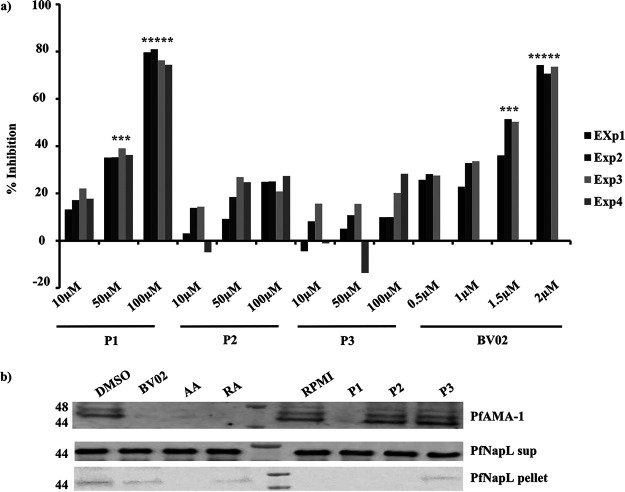
Inhibition of RBC invasion and microneme secretion by both PfPKAr-based phosphopeptide and Pf14-3-3I inhibitor. (a) Peptide P1 and BV02 both block RBC invasion by merozoites. P. falciparum merozoites were isolated and allowed to invade erythrocytes in the presence of increasing concentrations (10 μM, 50 μM, and 100 μM) of peptides P1 (NDDGpSpSDG, derived from the amino acid sequence of calcium-dependent phosphosite on PfPKAr encompassing Ser 113 and Ser 114), P2 (NDDGSSDG, nonphosphorylated control peptide), and P3 (pSDNGpSGDD, a control phosphopeptide based on scrambled P1 sequence) and increasing concentrations of 14-3-3 binding inhibitor, BV02 (0.5, 1, 1.5, and 2 μM). Newly invaded trophozoites were stained with SYBR green and scored by flow cytometry. Merozoites were allowed to invade erythrocytes in the absence of inhibitors using respective their solvents as controls. Percent invasion inhibition rates in the presence of inhibitors are shown for 3 (BV02) or 4 (P1, P2, P3) independent experiments. **, *P* < 0.005; ***, *P* < 0.005; *t* test. (b) Phosphopeptide P1, nonphosphorylated control peptide P2, scrambled phospho-peptide P3, phosphopeptides AA and RA based on 14-3-3 substrate binding sites, and BV02, a small-molecule 14-3-3 binding inhibitor, were tested for inhibition of PfAMA1 secretion by merozoites. Secretion of PfAMA1 was significantly reduced upon treatment of merozoites with phosphopeptides P1, AA, and RA and by BV02. Cytoplasmic protein PfNAPL was detected in the supernatant and used as a control for merozoite lysis. PfNAPL was detected in merozoite pellet and used as a control for normalization. A Western blotting image for one out of three independent experiments is shown. The plot shows the average percent inhibition of binding for three independent experiments. Mean ± SEM are shown (*n* = 3). **, *P* < 0.005 by *t* test.

Next, we investigated if disruption of Pf14-3-3I-mediated binding of PfPKAr and PfCDPK1 can inhibit RBC invasion. P. falciparum merozoites isolated in low K^+^ buffer were treated with increasing concentrations of peptides P1, P2, and P3 (10 μM, 50 μM, and 100 μM) and BV02 (0.5 μM, 1 μM, 1.5 μM, and 2 μM) and then incubated with RBCs in complete RPMI medium to allow invasion. Newly invaded ring-stage parasites were scored using flow cytometry. Treatment of merozoites with peptide P1 and BV02 reduced the efficiency of erythrocyte invasion in a dose-dependent manner (Fig. 6a). Control peptides, P2 (without phosphorylation) and P3 (scrambled phosphopeptide), had no inhibitory effect on invasion (Fig. 6a).

**FIG 7:**
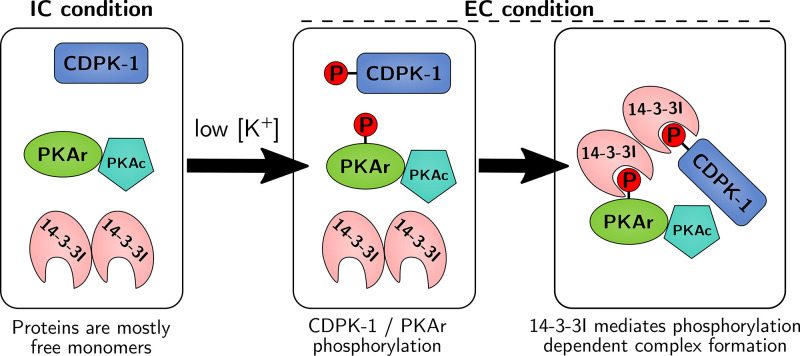
Model for assembly of a signaling complex during RBC invasion by P. falciparum merozoites. Exposure of merozoites to a low-K^+^ ionic environment as found in blood plasma triggers a signaling cascade, resulting in the phosphorylation of PfCDPK1 and PfPKAr. The scaffold protein Pf14-3-3I binds phosphorylated PfPKAr and PfCDPK1, leading to the formation of a high-molecular-weight multiprotein complex composed of PfCDPK1, PfPKAr, PfPKAc, and Pf14-3-3. Formation of this signaling complex plays a regulatory role in secretion of microneme proteins and merozoite invasion of RBCs.

The secretion of parasite invasion ligands from micronemes is a critical step in the invasion process. Given that PfCDPK1 and PfPKA have both been implicated in microneme secretion (10, 11), we examined if disruption of the high-molecular-weight multiprotein complex composed of PfCDPK1 and PfPKA can disrupt microneme secretion. Treatment of merozoites with peptide P1 inhibited secretion of microneme protein PfAMA1, whereas control peptides P2 and P3 had no effect (Fig. 6b). BV02 and peptides AA and RA also inhibit PfAMA1 discharge (Fig. 6b). Formation of the Pf14-3-3I-mediated multiprotein complex, which includes PfCDPK1 and PfPKA, thus appears to be important for regulation of microneme secretion, a key step in the invasion process (Fig. 7).

## DISCUSSION

Although there are many studies describing the intraerythrocytic phosphoproteomic profiles of P. falciparum blood stages, only one previous study described the phosphoproteome of free merozoites (17). However, the study did not address dynamic changes in the merozoite phosphoproteome during the key process of RBC invasion. Here, we investigated changes in the phosphoproteome of free merozoites following exposure to the natural environmental signal of low K^+^, as found in blood plasma during the process of invasion following egress of merozoites from schizonts.

This study identifies proteins that are phosphorylated in free merozoites isolated in a low K^+^ buffer. Protein-protein interaction networks were constructed for the identified phosphoproteins using the STRING database (20). Analysis of these networks using the MCODE clustering algorithm (21), identified 17 subnetworks or MCODE clusters (Fig. S2). Gene ontology analysis of the proteins in the MCODE clusters using the PlasmoDB database found that MCODE cluster 1 was enriched for invasion- and signaling-related proteins. The predicted interactions between the invasion- and signaling-related proteins in MCODE cluster 1 are shown in Fig. 1d. Some of the notable invasion-related proteins in MCODE cluster 1 include the inner membrane complex (IMC) proteins such as PfIMC1c and PfIMC1g that are associated with merozoite motility (1, 2). These proteins connect to myosin filaments that form the conserved molecular machinery for merozoite motility that is necessary for invasion. Various myosin molecules, PfMyoA, PfMyoB, and PfMyoE, as well as glideosome-associated proteins, PfGAP40 and PfGAP45, that are phosphorylated are also included in MCODE cluster 1. It was recently proposed that regulated phosphorylation of S19 in PfMyoA may enhance force generation during parasite invasion (25). In addition to merozoite motility proteins, several parasite proteins that mediate interactions with RBC receptors (e.g., EBA140, EBA181, MSP1, AMA1, RONs, and RhopH2) and proteases (SERA5, SUB2) that participate in maturation and activation of key effector proteins involved in invasion are also found in MCODE cluster 1 (Fig. 1d).

RBC invasion by merozoites is driven by the merozoite motility machinery and requires secretion and proteolytic maturation of parasite ligands that interact with RBC receptors. Timely activation of these molecular processes requires signaling pathways that respond to external signals. Signaling cascades are initiated at the time of merozoite egress and RBC invasion through the generation of second messengers, including Ca^2+^, cAMP, and cGMP (1, 2), which activate kinases, including PfCDPKs, PfPKA, and PfPKG. Exposure of merozoites to a low-K^+^ environment is a key external signal that leads to a rise in Ca^2+^ and cAMP in merozoites (7). Ca^2+^ activates PfCDPK1, which is implicated in RBC invasion (10). It has not been possible to knock out the gene for PfCDPK1 in wild-type P. falciparum, suggesting that it plays an important role in parasite multiplication (26). However, the wild-type PfCDPK1 gene could be replaced with a mutant copy with significantly reduced PfCDPK1 activity. These mutant parasites were viable but multiplied with reduced efficiency (26). Moreover, the mutant parasites had compensatory changes in expression of PfCDPK5 and PfCDPK6, as well as PfPKG, which may have enabled parasite growth in the absence of fully functional PfCDPK1 (26). The gene for PfCDPK1 could be deleted in these mutant parasites, which suggests that PfCDPK1 is dispensable for survival (27). A study that used conditional knockdown to deplete PfCDPK1 in wild-type P. falciparum confirmed that PfCDPK1 plays a role in RBC invasion and secretion of microneme proteins (10). Conditional depletion of PfCDPK1 blocked secretion of microneme protein PfEBA175, but surprisingly, did not affect secretion of PfAMA1, another microneme protein (10). This observation may be attributed to the presence of residual PfCDPK1 in the conditional knockdown parasites, where PfCDPK1 expression was reduced by 70%. Alternatively, an abundance of PfAMA1 in merozoites compared to other microneme proteins such as PfEBA175 may be responsible for the PfAMA1 secretion observed following partial PfCDPK1 knockdown in these parasites (10). Exposure of P. falciparum merozoites to low K^+^ also results in a rise in cytosolic cAMP in merozoites, which activates PfPKA (8). Conditional depletion of PfPKAc, the catalytic domain of PfPKA, reduces the efficiency of RBC invasion by merozoites (11). PfPKAc was previously shown to phosphorylate the cytoplasmic domain of PfAMA1 at S610 (11, 28). Phosphorylation of PfAMA1 at S610 was not detected in merozoites in this study. This may be due to differences in the conditions for merozoite isolation, since previous studies observed phosphorylation changes in late-stage schizonts, whereas this study used free merozoites collected following schizont rupture. The cyclic nucleotide cGMP regulates activity of the kinase PfPKG, which also plays an important role in merozoite egress during blood-stage growth (12). The natural signal that leads to a rise in cytosolic cGMP levels in merozoites is not known. Raising cGMP levels artificially by treating merozoites with phosphodiesterase inhibitors such as zaprinast, activates PfPKG and raises cytosolic levels of Ca^2+^, leading to microneme release and activation of merozoite egress from mature schizonts (12). MCODE cluster 1 includes several kinases, including calcium-dependent protein kinase, PfCDPK1, PfPKAc, PfPKAr, and PfPKG, suggesting that these phosphoproteins may interact in merozoites to mediate RBC invasion.

Given that exposure to low K^+^ provides the signal for activation of steps such as microneme release during invasion, we investigated changes in protein phosphorylation following the transfer of free merozoites from a buffer with high K^+^ (IC buffer) to one with low K^+^ (EC buffer). The role of intracellular Ca^2+^ in phosphorylation was also investigated by transferring merozoites to EC buffer with the intracellular Ca^2+^ chelator, BAPTA-AM (EC-BA). Phosphorylation changes occurred at 394 sites located on 314 peptides, with 76 peptides having dual phosphorylations when merozoites were transferred from IC to EC buffer. Out of these, 143 phosphorylation events were found to be Ca^2+^-dependent and were located on 119 peptides, 24 of which displayed dual phosphorylations. The employment of immobilized metal affinity chromatography (IMAC)-based enrichment identified many dual phosphorylation events which were not previously reported. Such dual phosphorylations can influence the activity of kinases. For example, dual phosphorylation of extracellular signal-regulated kinase 2 (ERK2) increases its activity by 10- to 100-fold (29). We observed many calcium-dependent dual phosphorylations on proteins with diverse functions in the life cycle of the parasite. These included proteins known to be involved in organelle secretion and invasion-related processes. For example, dual phosphorylation of inner membrane complex (IMC) protein PfIMC1g at Thr189/Ser191 and Tyr272/Ser274, and the glideosome-associated protein PfGAP45 at Ser156/Thr158 was observed. Both these proteins are known to be phosphorylated by calcium-dependent kinase, PfCDPK1 (10). We also observed Ca^2+^-dependent dual phosphorylation of PfPKAr at Ser 113/Ser 114. In contrast, dual phosphorylation at Ser 28/Ser 34 of PfCDPK1 was not Ca^2+^-dependent.

Interactions between PfPKAr and PfCDPK1 and between PfPKAr and Pf14-3-3I were observed previously (10, 30). Here, we demonstrate that these interactions are phosphorylation dependent and dynamic (Fig. 3). Moreover, we demonstrate the formation of a high-molecular-weight (150 to 250 kDa) multiprotein signaling complex that assembles in merozoites in response to an environmental signal; namely, a change in the environmental ionic composition (Fig. 4). PfCDPK1 was previously shown to be present in merozoites in a high-molecular-weight complex, but the composition and dynamic nature of the complex was not described (13). A related study showed using immunofluorescence microscopy that Pf14-3-3I colocalizes with PfCDPK1 at the periphery of merozoites (31). Our mass spectrometric analyses demonstrated that the complex contains PfCDPK1, Pf14-3-3I, PfPKAr, and PfPKAc. Interestingly, PfPKG, which has also been implicated in apical organelle secretion during merozoite invasion (12), is not present in this multiprotein signaling complex. A previous study on protein signaling complexes also did not detect PfPKG in the high-molecular-weight complex with PfCDPK1 (13).

Members of the 14-3-3 family of scaffold proteins bind target proteins in a phosphorylation-dependent manner through recognition of optimal consensus sequence motifs corresponding to mode I (RXXpS/pT), mode II (RXXXpS/pT), or mode III (RXXpS/pTX1-2), thus regulating a wide variety of cellular processes (32–35). Disruption of the interactions mediated by 14-3-3 proteins results in ablation of key cellular processes. Here, we show that peptides based on the dual phosphorylation of PfPKAr at Ser 113 and Ser 114 (peptide P1), as well as inhibitory peptides, AA and RA, that are based on consensus sequences in 14-3-3 binding proteins from mammalian cells, and 14-3-3-based inhibitory small molecule, BV02, all inhibited the formation of the multiprotein complex (Fig. 5b and c). The inhibitory peptides and BV02 also blocked secretion of microneme protein PfAMA1 and RBC invasion (Fig. 6), demonstrating that assembly of this signaling complex plays a critical role in these processes. A related study has confirmed the phosphorylation-dependent interaction of recombinant Pf14-3-3I and PfCDPK1 using enzyme-linked immunosorbent assay (ELISA) plate-based binding assays, as well as surface plasmon resonance (SPR) and isothermal calorimetry (ITC) (31). Moreover, the study showed that peptides AA and RA inhibit PfCDPK1 binding with Pf14-3-3I and block blood-stage parasite growth (31). Here, we demonstrate that phosphopeptides P1 and P2, which are based on PfPKAr sequences that interact with Pf14-3-3I, also inhibit interaction of PfPKAr with Pf14-3-3I in the merozoite to block miconeme secretion and RBC invasion by P. falciparum merozoites.

The 14-3-3 homologs in mammalian cells are known to serve as the central hub for signaling networks that regulate cell proliferation, adhesion, survival, and apoptosis (22). Given their central role in cell growth, 14-3-3 is also implicated in the development of cancer. Small-molecule inhibitors that target the scaffold function of 14-3-3 are being developed for cancer therapy (36). In this study, we showed that targeting the assembly of the multiprotein complex in merozoites mediated by Pf14-3-3I provides a novel strategy to inhibit the blood-stage growth of malaria parasites and may provide a strategy for the development of novel drugs against malaria.

## MATERIALS AND METHODS

### P. falciparum merozoite isolation.

P. falciparum 3D7 blood stages were cultured *in vitro*, and merozoites were isolated as previously described (7, 8, 37). Mature synchronized schizonts were transferred to IC buffer (142 mM KCl, 5 mM NaCl, 2 mM EGTA, 1 mM MgCl_2_, 5.6 mM glucose, and 25 mM HEPES, pH 7.2). Released merozoites were collected by centrifugation as described previously (7, 8) and resuspended in IC buffer, EC buffer (5 mM KCl, 142 mM NaCl, 1 mM CaCl_2_, 1 mM MgCl_2_, 5.6 mM glucose, and 25 mM HEPES, pH 7.2), or EC-BA buffer (EC buffer supplemented with 50 mM BAPTA-AM [Calbiochem]) at 37°C for 15 min with or without inhibitors as required. Merozoite pellets were prepared by centrifugation at 3,300 × *g* for 5 min and stored at –80°C for further analysis. Purity of merozoite preparations was confirmed by Giemsa staining the merozoite preparations and observation by light microscopy.

### Protein isolation, desalting, and digestion.

Isolated merozoites were lysed by incubation with urea lysis buffer on ice for 15 min followed by sonication for 3 × 30 s on ice. Protein concentration was quantified with a Pierce BCA protein assay kit as per the supplier’s protocol. Then, 6 to 7 mg of total protein was used for each biological replicate for the label-free phosphoproteomics experiment. For the quantitative experiment, 100 μg of total protein isolated from IC-, EC-, and EC-BA-treated merozoites was used for labeling as described below. The isolated proteins were reduced, alkylated, and digested with trypsin gold (Promega) with a 1:200 enzyme to substrate ratio at 37°C overnight. Tryptic digested peptides from the label-free experiment were desalted with reverse-phase tC_18_ SepPak solid-phase extraction cartridge 500 mg (Waters) as described previously (38).

### Ion-exchange fractionation of P. falciparum merozoite lysates.

Fractionation of desalted peptides was carried out with strong cation exchange (SCX) chromatography on a polySULFOETHYL A column as previously described (38). Then, 12 to 15 fractions of 4 ml were collected, lyophilized until the volume was reduced to 30%, and desalted as described above.

### Tandem mass tag (TMT) labeling and fractionation using hydrophilic interaction liquid chromatography (HILIC).

Proteins isolated from merozoites in IC, EC, and EC-BA buffers were digested with trypsin, and peptides were labeled separately with TMT tags with masses of 128, 129, and 130 as per the manufacturer’s instructions. Labeled peptide samples were combined and fractionated by HILIC using method described previously (39). Then, 12 to 15 fractions (0.5 ml) were collected and lyophilized for further use.

### Immobilized metal affinity chromatography (IMAC) and TiO_2_/ZrO_2_ phosphopeptide enrichment and desalting.

Combined IMAC-based phosphoproteomic enrichment and desalting were carried out as described previously (38). Peptide fractions were incubated on a rotating platform with IMAC beads (PHOS-Select iron affinity gel [Sigma]) for 1 h at room temperature (RT). During this time, StageTips (40) were prepared using Empore 3M C_18_ material (Fisher Scientific). After incubation, IMAC beads were added on top of the StageTips, and the flowthrough was collected and concentrated on a SpeedVac instrument. Phosphopeptides were eluted from IMAC resin onto C_18_ loaded tips and desalted. Phosphopeptides were eluted from C_18_ StageTips, lyophilized, and stored at –80°C. Flowthrough from IMAC was used further for phosphopeptide enrichment using TiO_2_/ZrO_2_ NuTip (Glygen) as per the manufacturer’s protocol. Phosphopeptides were eluted, concentrated by SpeedVac centrifugation, and stored at –20°C until further analysis.

### Liquid chromatography-tandem mass spectrometry (LC-MS/MS).

LC-MS/MS was carried out using a Nano LC-1000 HPLC nanoflow system (Thermo Fisher Scientific) and hybrid Orbitrap Velos Pro mass spectrometer (Thermo Fisher Scientific). Peptides were separated with a 120 min gradient using Acclaim PepMap100 C_18_ column and eluted onto the mass spectrometer. Data acquisition was performed in a data-dependent mode to automatically switch between MS and MS2. Full-scan MS spectra of intact peptides (*m/z* 350 to 1,000) were acquired in the Orbitrap with a resolution of 60,000. The top 20 precursors were sequentially isolated and fragmented in the high-energy collisional dissociation (HCD) cell. Dynamic exclusion was 50 s and a minimum 500 counts for nonlabeled, and 200 counts for TMT sets were required for MS2 selection.

### Data analysis for TMT and label-free phosphoproteomic data.

All raw files were searched against a P. falciparum database using an OpenMS pipeline (41) containing the two search engines Mascot and MSGF+, followed by Percolator postprocessing and phosphorylation analysis using PhosphoScoring, an implementation of the Ascore algorithm (42). The search parameters were as follows: carbamidomethylation of cysteines was set as a fixed modification; oxidation of methionine, protein N-terminal acetylation, and serine/threonine/tyrosine (STY) phosphorylation were set as variable modifications. The mass tolerances in MS and MS/MS were set to 20 ppm and 0.5 Da, respectively. A false-discovery rate of 1% was set up for both protein and peptide levels. In the TMT experiment, TMT-6plex labeling on lysine and N termini was searched for protein quantitation. A phosphoscore over 11 was considered a significant localization score.

Data were also searched using MaxQuant version 1.5.3.8 (with the Andromeda search engine) against a P. falciparum database. The following search parameters were applied: carbamidomethylation of cysteines was set as a fixed modification, and oxidation of methionine, protein N-terminal acetylation, and STY phosphorylation were set as variable modifications. The mass tolerances in MS and MS/MS were set to 5 ppm and 0.5 Da, respectively. A false-discovery rate of 1% was set up for both protein and peptide levels. TMT-6plex labeling on lysine and N termini was searched for protein quantitation. Phospho-localization probability of more than 75% was considered significant localization. Quantification from MaxQuant analysis was used for quantification of changes in the phosphorylation. All phospho-spectra of interest were manually validated.

To determine whether the variation of the quantification of a phosphopeptide is due to a variation in the abundance of the protein itself or due to a variation in the abundance of its modification, a statistical test was performed to compare the variation in abundance of each phosphopeptide to the abundance of the corresponding protein. To do this for a specific phosphopeptide, we first estimated the average, *m*, and standard deviation, *s*, of the log_2-fold_ change (log_2_FC) of the nonmodified peptides of the protein (where FC is equal to either $\overset{¯}{\mathrm{EC}}/\overset{¯}{\mathrm{IC}}$ or $\bar{\mathrm{EC}-\mathrm{BA}}/\bar{\mathrm{IC}}$, $\bar{x}$ being the average intensity observed for a peptide under the condition x). Assuming that the log_2_FC of the nonmodified peptides follows a normal distribution centered on *m* and having a standard deviation of *s*, we deduce that a *P* value related to the test that the measured log_2_FC for the phosphopeptide is equal to *m* by $2\times P_{N(m,s)}(\text{log}2(\text{FC}))$ if log2(FC)<m and $2\times (1-P_{N\left(m,s\right)}(\text{log}2(\text{FC})))$ if log2(FC)≥m, where $P_{N(m,s)}$ is the cumulative distribution function of N(m,s). Note that this *P* value is computed only when we have at least 3 nonmodified peptides with intensity values for a protein.

### Phosphorylated protein interaction network analysis.

The merozoite phosphoproteome interaction network was constructed using the STRING database (20) and visualized in Cytoscape version 3.7.1 (43). The merozoite phospho-interactome was analyzed for highly connected nodes with the molecular complex detection clustering algorithm MCODE (21).

### Gene ontology and motif analysis.

All P. falciparum gene ontology analyses were performed with the built-in result analysis tool for gene ontology on the PlasmoDB database. Phosphopeptides with a width of 15 amino acids were subjected to motif analysis using MotifX (44, 45). A background of the P. falciparum protein database was used for the analysis, and the occurrence threshold set to a default *P* value threshold of ≤1e^−6^ was used to identify enriched motifs.

### Immunoprecipitation (IP), LC-MS/MS, and data analysis.

IP of proteins from merozoites isolated in cRPMI 1640 medium (Roswell Park Memorial Institute 1640 medium supplemented with Albumax I) or resuspended in IC, EC, and EC-BA buffers or treated with specific inhibitors or peptides was performed with a Pierce coimmunoprecipitation (co-IP) kit as per the manufacturer’s protocol. The identity of proteins from the eluate of IP experiments was investigated using an Orbitrap Q Exactive Plus mass spectrometer (Thermo Fisher Scientific) or by Western blotting. Data were searched using MaxQuant as described above.

### Gel filtration on Superdex 200.

P. falciparum merozoites treated with IC or EC buffer were lysed and cleared by centrifugation. Proteins were fractionated with a Superdex 200 column (GE Healthcare; 10 × 300 mm). Fractions of 1 ml were collected and analyzed by Western blotting for the presence of PfPKAr, Pf14-3-3I, and PfCDPK1 in respective fractions.

### Binding of recombinant Pf14-3-3I to synthetic peptide-coated beads.

Peptides (AA, RA, P1, P2, and P3) were coupled to agarose beads using a co-IP kit (Pierce) as per the manufacturer’s instruction. Peptide-coated beads were incubated with GST-tagged Pf14-3-3I protein; noncoated beads were used as a control. Recombinant protein bound to the beads was eluted, and eluates were tested by Western blotting using anti-Pf14-3-3I mouse sera.

### Invasion assay with P. falciparum merozoites and flow cytometry.

Merozoites isolated as described above were treated with inhibitors (BV02 at 0.5 μM, 1 μM, 1.5 μM, and 2 μM) and peptides (P1, P2, and P3 at 10 μM, 50 μM, and 100 μM) for 15 min at 37°C followed by incubation with RBC in the presence of the inhibitor or peptide to allow invasion and growth for 24 h under standard culturing conditions. Solvents used to dissolve the inhibitors were used as a control. The parasitemia was determined by flow cytometry after staining with SYBR GreenI (Sigma) as previously described (46). Data were analyzed with FlowJo (Tree Star) and the percent inhibition of invasion was calculated using the formula (1 – *T*/*C*) × 100; where *T* and *C* denote parasitemia in treatment and control samples, respectively.

### Microneme secretion assay.

P. falciparum merozoites isolated in cRPMI were incubated for 15 min at 37°C with BV02 (2 μM), AA (100 μM), RA (100 μM), or DMSO (solvent) and with peptides P1, P2, P3 (100 μM each), or RPMI (solvent). Following incubation, merozoites and supernatants were separated by centrifugation, and the presence of PfAMA1 (microneme protein) and PfNapL (cytosolic protein used as lysis control) in the supernatant and PfNapL in the pellet (cytosolic protein used as a loading control) were detected by Western blotting as described above.

### Densitometry and statistical analysis.

ImageJ (NIH) software was used to perform densitometry of Western blots. The band intensity of the loading control was used for normalization. Statistical analysis for all the plots was performed using GraphPad Prism version 8.1.2 software. All experiments were analyzed using multiple *t* tests (assuming equal standard error [SE]), and *P* ≤ 0.05 was considered significant. *, *P* < 0.05; **, *P* < 0.005; ***, *P* < 0.0005. The graphs were plotted with a mean ± SE of the mean (SEM) of the population.

### Data availability.

The mass spectrometry-based proteomics data have been deposited in the ProteomeXchange Consortium (http://proteomecentral.proteomexchange.org) via the PRIDE (47) partner repository with the data set identifier PXD015093. All other relevant data are available from the authors upon request.
