# Supplementary material for: Phosphorylation-Dependent Assembly of a 14-3-3 Mediated Signaling Complex during Red Blood Cell Invasion by Plasmodium falciparum Merozoites
Source: mBio. 2020 Aug 18;11(4):e01287-20. doi: 10.1128/mBio.01287-20 (PMC7439480; doi:10.1128/mBio.01287-20)
Supplement: FIG S1 [file mBio.01287-20-sf001.pdf]

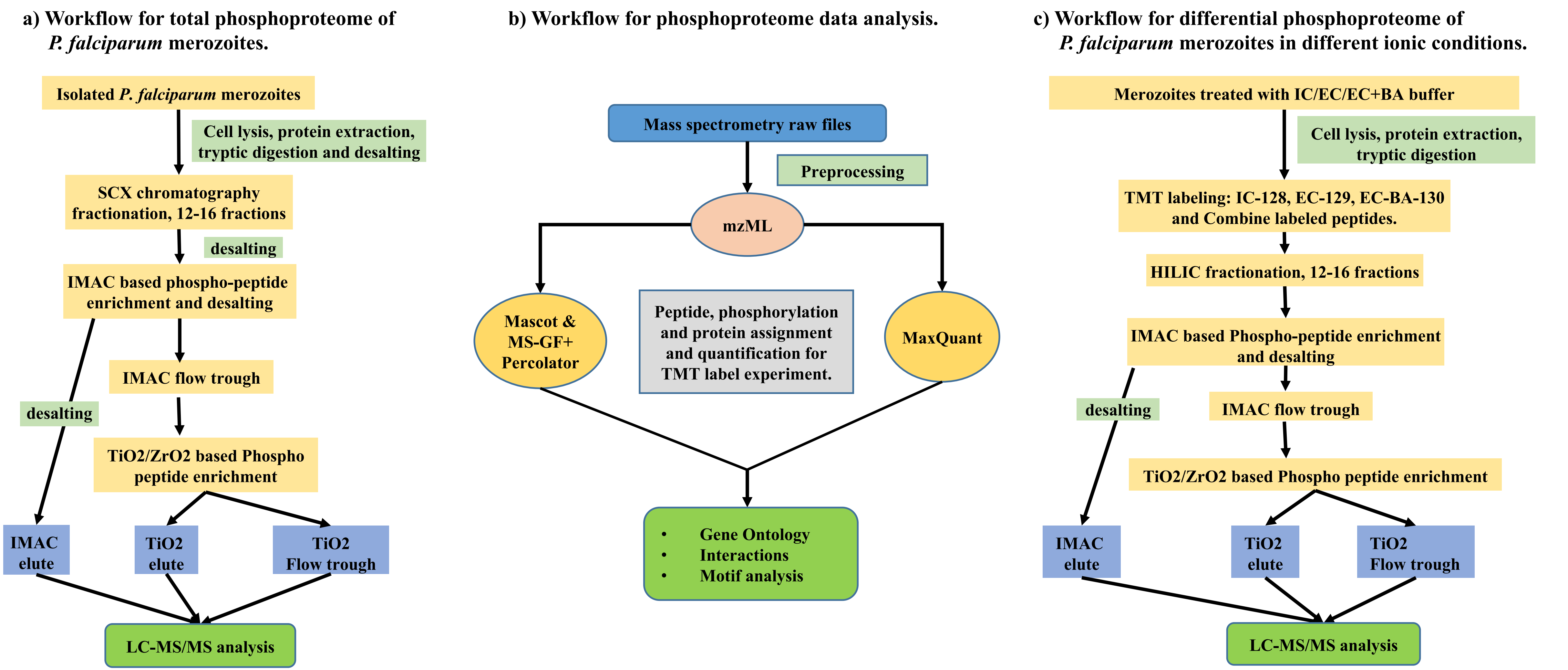

Figure S1. Schematic representation of workflows for phosphoproteomics, quantitative phosphoproteomics, and data analysis: a) Workflow for total phosphoproteomic analysis of *P. falciparum* merozoites. The steps to identify phosphopeptides in tryptic digests of proteins derived from merozoites are shown. After digestion with trypsin, peptides were fractionated by Strong Cation Exchange (SCX) chromatography, enriched for phosphopeptides, first by Fe^{3+}-based immobilized metal affinity chromatography (IMAC) followed by enrichment of phosphopeptides on TiO_2/ZrO_2 tips and analyzed by LC-MS/MS. **b)** Workflow for phosphoproteomic data analyses. All the raw files were converted to MZML file format using MSConvertGUI and analyzed with OpenMS pipeline using Mascot and MSGF+ algorithms followed by post-processing with Percolator, as well as MaxQuant pipeline for peptide and protein assignment. Peptides and proteins commonly identified by these pipelines were used for further bioinformatics analyses including gene ontology, identification of protein interaction networks and motif analysis. **c)** Workflow for differential quantitative phosphoproteomic analysis of *P. falciparum* merozoites in different ionic conditions**.** Isolated free merozoites were resuspended in high K^+ containing buffer mimicking intracellular ionic conditions (IC), low K^+ containing buffer mimicking extracellular ionic conditions (EC) and EC buffer supplemented with the intracellular Ca^{2+} chelator, BAPTA-AM. Proteins were isolated and digested with trypsin to generate peptides. Peptides from different treatment conditions were labeled separately with Tandem Mass Tags (TMT) (IC – 128, EC – 129, EC-BA – 130) and combined together. TMT labeled peptides were fractionated using hydrophilic interaction liquid chromatography (HILIC), enriched for phosphopeptides first by Fe^{3+}-based IMAC followed by enrichment of phosphopeptides on TiO_2/ZrO_2 tips and analyzed by LC-MS/MS.
