# Supplementary material for: Phosphorylation-Dependent Assembly of a 14-3-3 Mediated Signaling Complex during Red Blood Cell Invasion by Plasmodium falciparum Merozoites
Source: mBio. 2020 Aug 18;11(4):e01287-20. doi: 10.1128/mBio.01287-20 (PMC7439480; doi:10.1128/mBio.01287-20)
Supplement: FIG S2 [file mBio.01287-20-sf002.pdf]

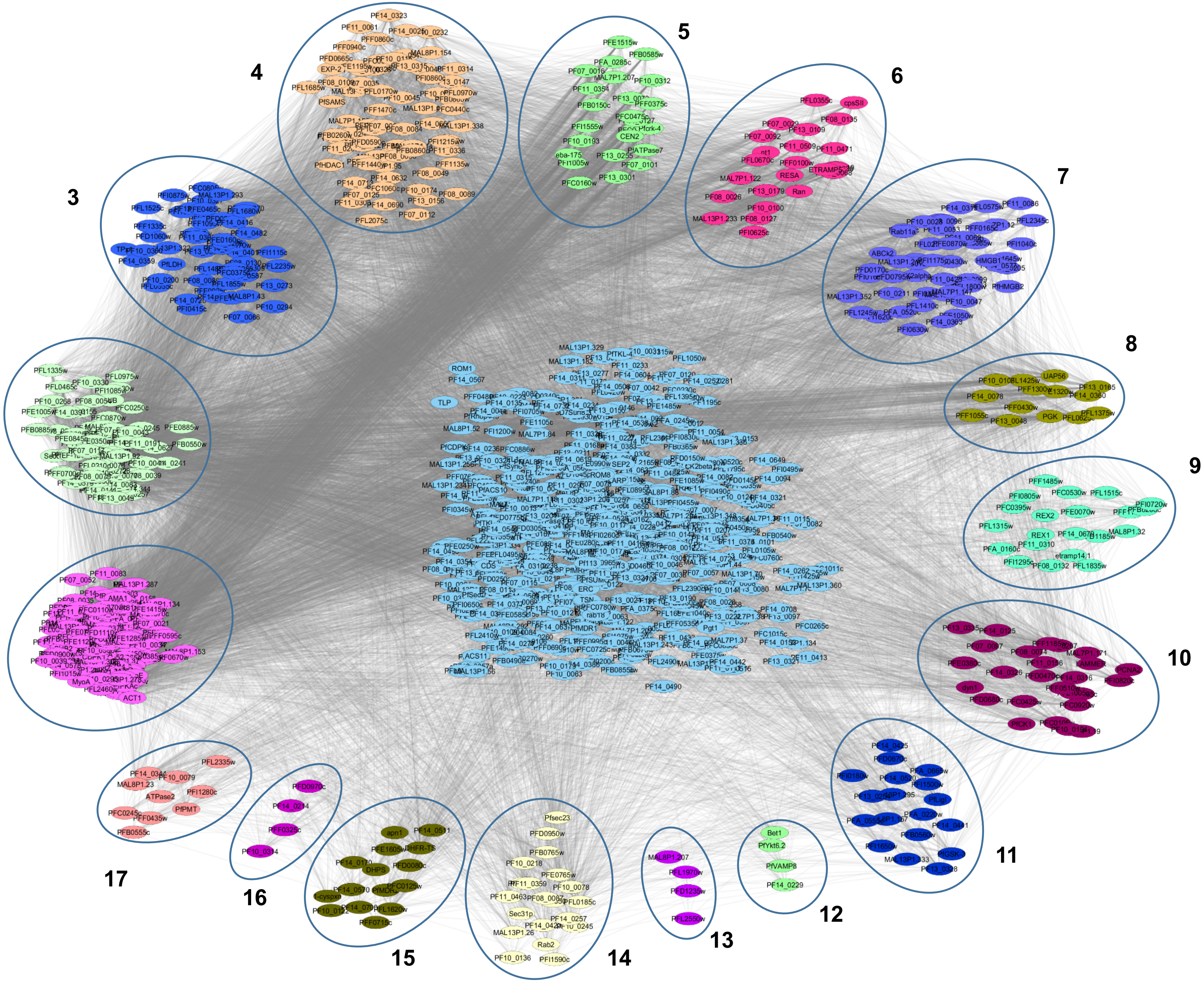

Figure S2. The predicted protein-protein interaction network of *P. falciparum* merozoites: Protein interaction data downloaded from STRING for phosphoproteins found in this study and visualized in Cytoscape. MCODE clustering algorithm is used for the generation of the subnetwork of highly interacting proteins. MCODE clusters are circled, numbered accordingly, and shown in specific colors to differentiate them from non-clustered proteins that are shown in the middle of the network. 17 out of the 24 clusters identified are shown.
