# Supplementary material for: Phosphorylation-Dependent Assembly of a 14-3-3 Mediated Signaling Complex during Red Blood Cell Invasion by Plasmodium falciparum Merozoites
Source: mBio. 2020 Aug 18;11(4):e01287-20. doi: 10.1128/mBio.01287-20 (PMC7439480; doi:10.1128/mBio.01287-20)
Supplement: FIG S3 [file mBio.01287-20-sf003.pdf]

a)

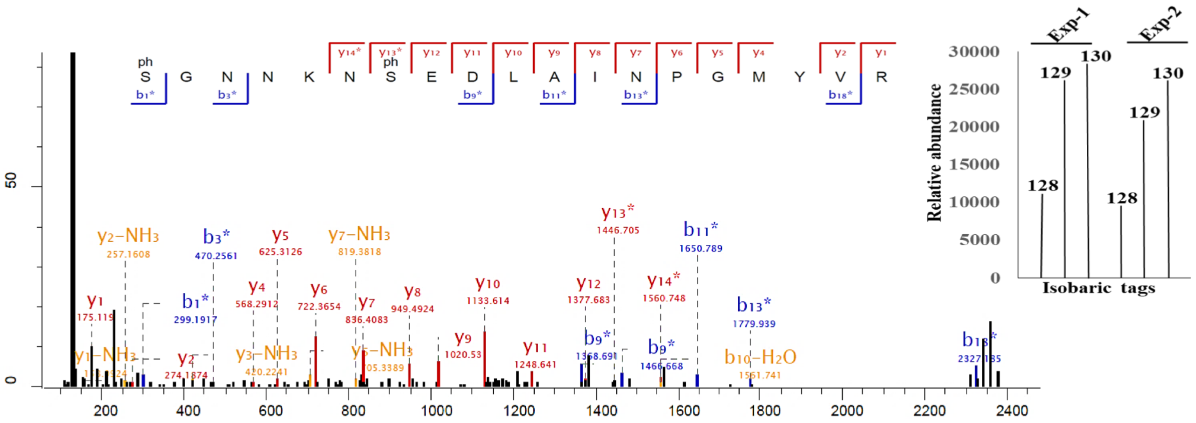

b)

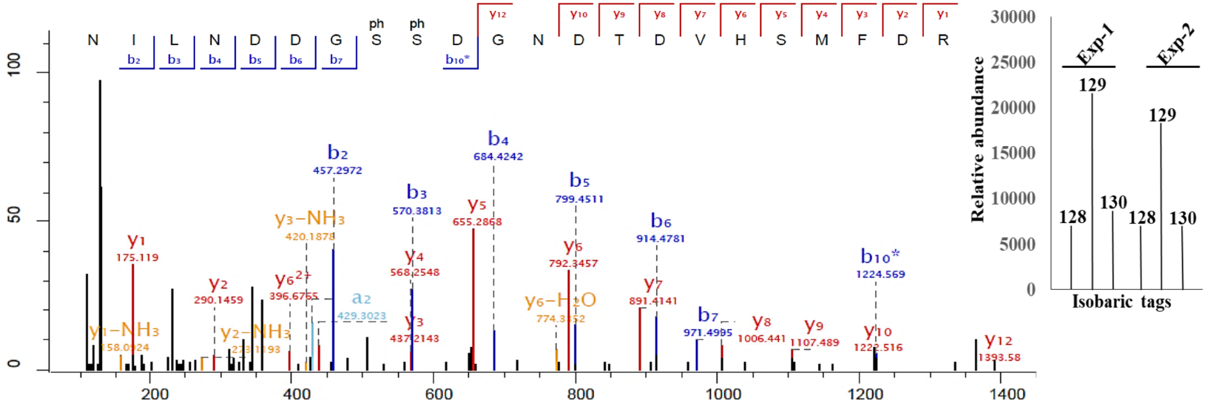

Figure S3. Representative spectra and quantification profile. a) Representative spectra demonstrating phosphorylation of Ser28 and Ser34 in PfCDPK1. **b)** Representative spectra demonstrating phosphorylation on PfPKAr at Ser113 and Ser114. The relative abundance of reporter ions from two experiments is also shown. Peptides were labeled with TMT mass tags 128 (IC), 129 (EC) and 130 (EC-BA).
