# Supplementary material for: Phosphorylation-Dependent Assembly of a 14-3-3 Mediated Signaling Complex during Red Blood Cell Invasion by Plasmodium falciparum Merozoites
Source: mBio. 2020 Aug 18;11(4):e01287-20. doi: 10.1128/mBio.01287-20 (PMC7439480; doi:10.1128/mBio.01287-20)
Supplement: FIG S4 [file mBio.01287-20-sf004.pdf]

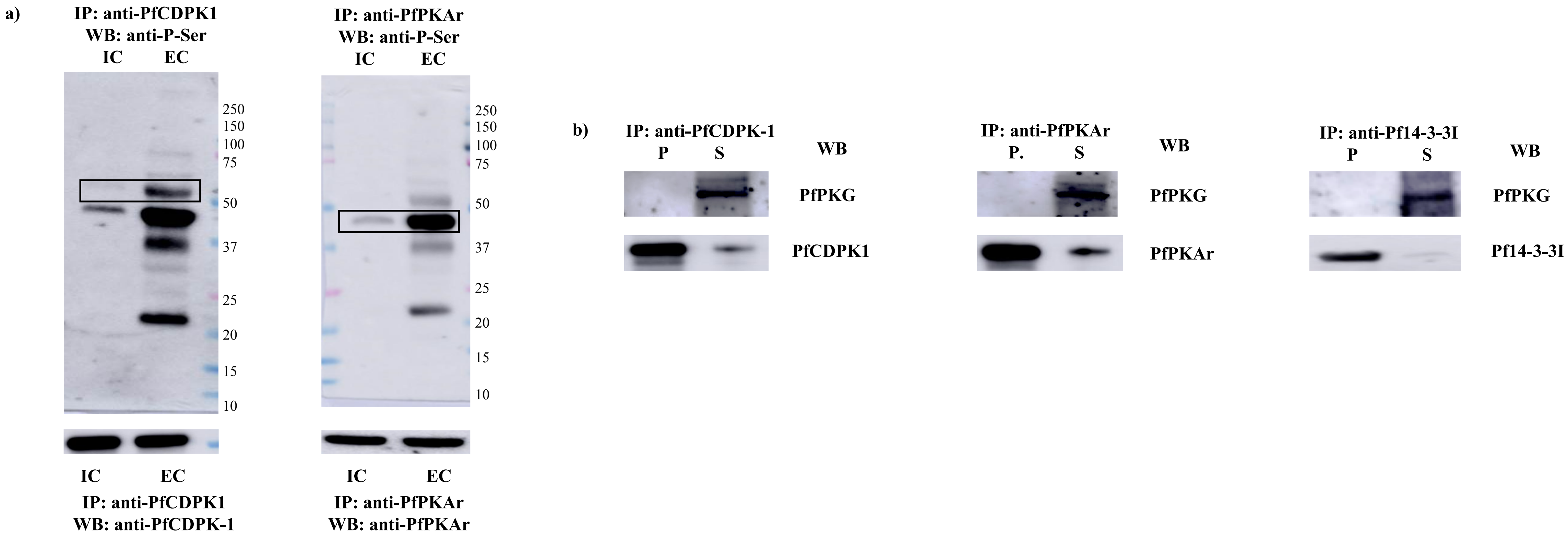

Figure S4. a.) Level of phosphorylation of serines in *P. falciparum* merozoite signaling proteins in response to exposure to extracellular ionic environment with low K^+. Lysates of merozoites in intracellular buffer (IC) and extracellular buffer (EC) were used for immunoprecipitation with anti-PfCDPK1 and anti-PfPKAr sera. The imunoprecipitates (IP) were probed with anti-phospho-serine antibodies as well anti-PfCDPK1 and anti-PfPKAr sera. PfCDPK1 and PfPKAr have higher levels of serine phosphorylation in EC compared to IC buffer (black boxes). **b.) Detection *P. falciparum* protein kinase G (PfPKG) in immunoprecipitates with anti-PfCDPK1, anti-PfPKAr and anti-Pf14-3-3I sera.** Merozoite lysates were immunoprecipitated (IP) with anti-PfCDPK1, anti-PfPKAr and anti-Pf14-3-3I sera and probed for presence of PfPKG by western blotting. PfPKG is detected in the merozoite lysate supernatant (S) but not in the IP pellets (P).
