# Supplementary material for: Phosphorylation-Dependent Assembly of a 14-3-3 Mediated Signaling Complex during Red Blood Cell Invasion by Plasmodium falciparum Merozoites
Source: mBio. 2020 Aug 18;11(4):e01287-20. doi: 10.1128/mBio.01287-20 (PMC7439480; doi:10.1128/mBio.01287-20)
Supplement: DATA SET S4 [file mBio.01287-20-sd004.pdf]

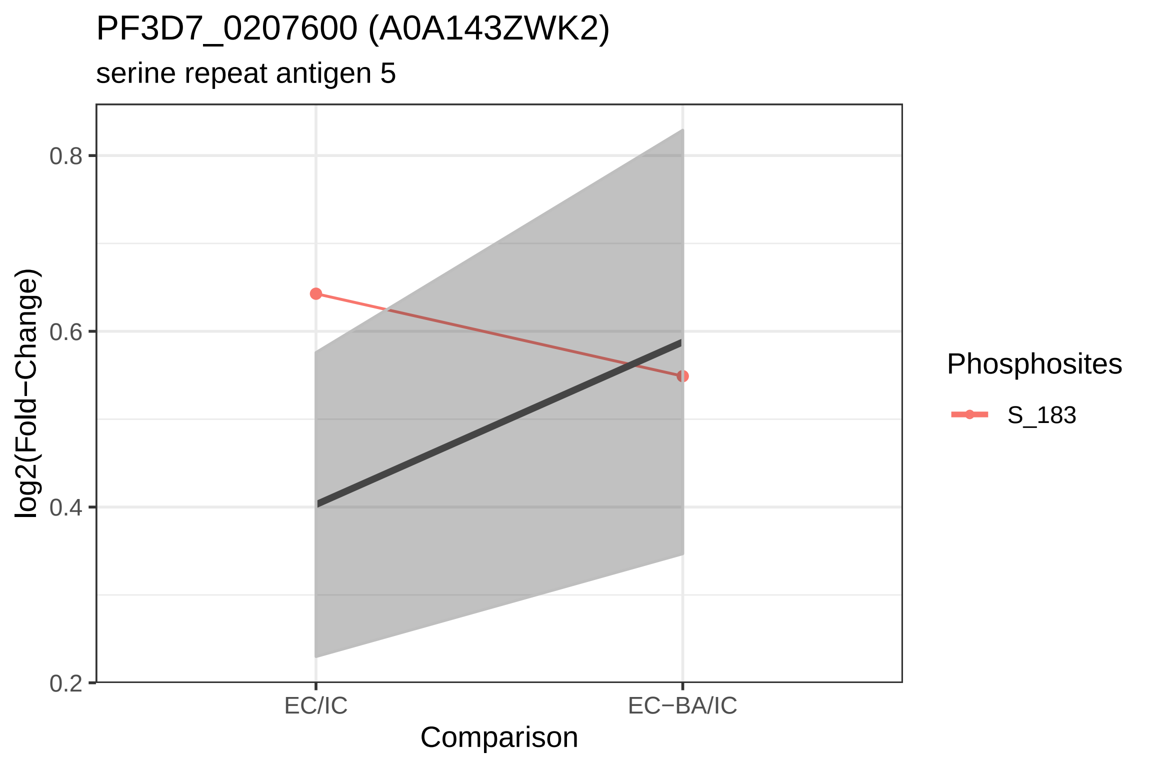

PF3D7_0207600 (A0A143ZWK2)
serine repeat antigen 5
log2(Fold−Change)
0.8
0.6
0.4
0.2
EC/IC
EC−BA/IC
Comparison
Phosphosites
S_183

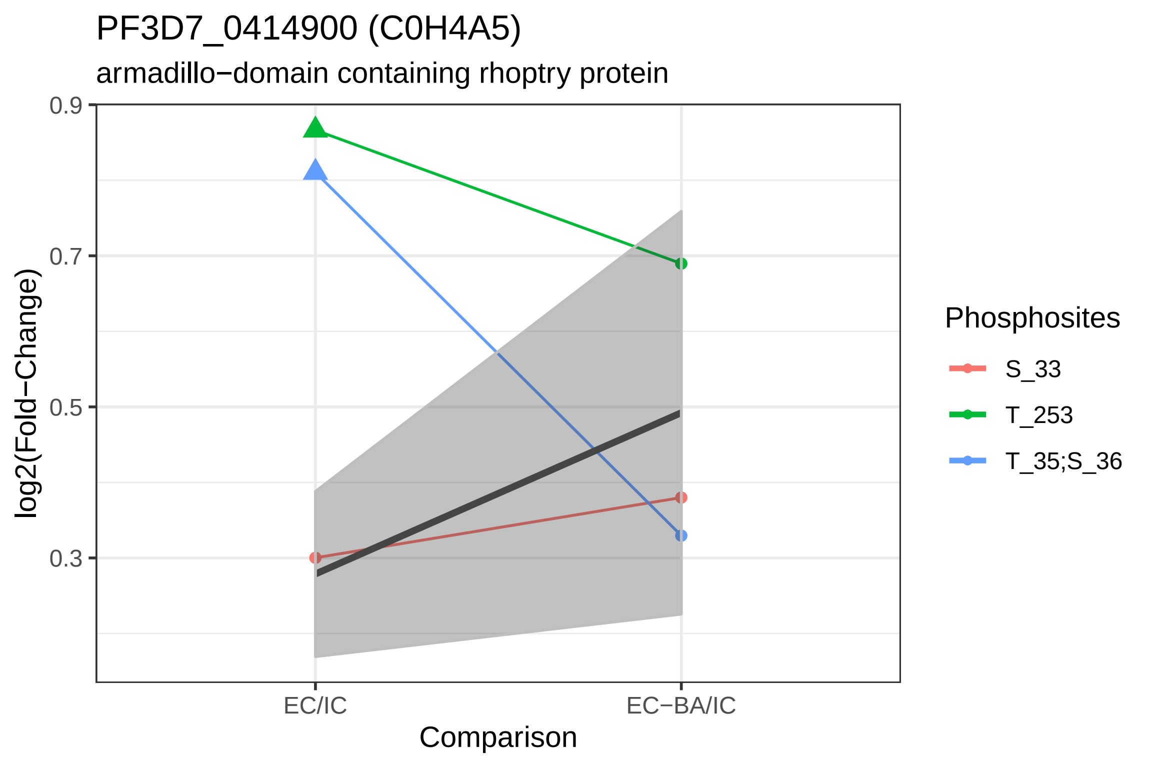
PF3D7_0414900 (C0H4A5)
armadillo−domain containing rhoptry protein
log2(Fold−Change)
0.9
0.7
0.5
0.3
EC/IC
EC−BA/IC
Comparison
Phosphosites
S_33
T_253
T_35;S_36

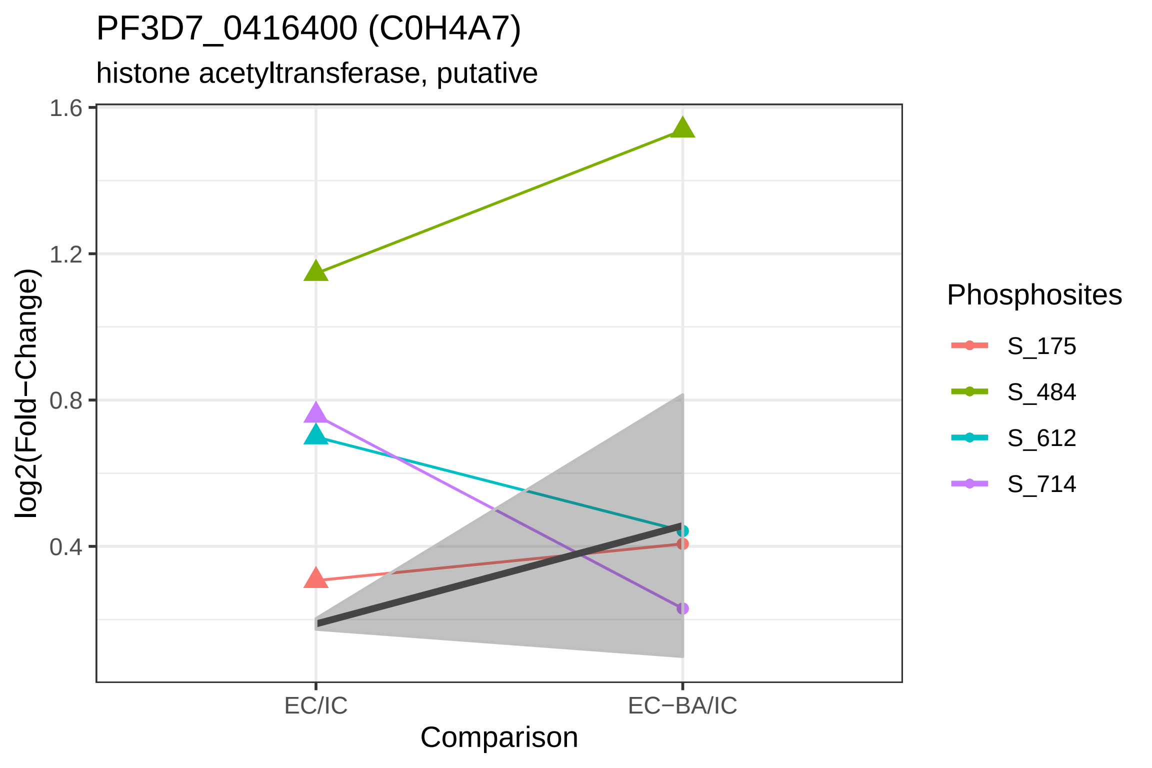

PF3D7_0416400 (C0H4A7)
histone acetyltransferase, putative
1.6
1.2
0.8
0.4
log2(Fold−Change)
EC/IC
EC−BA/IC
Comparison
Phosphosites
S_175
S_484
S_612
S_714

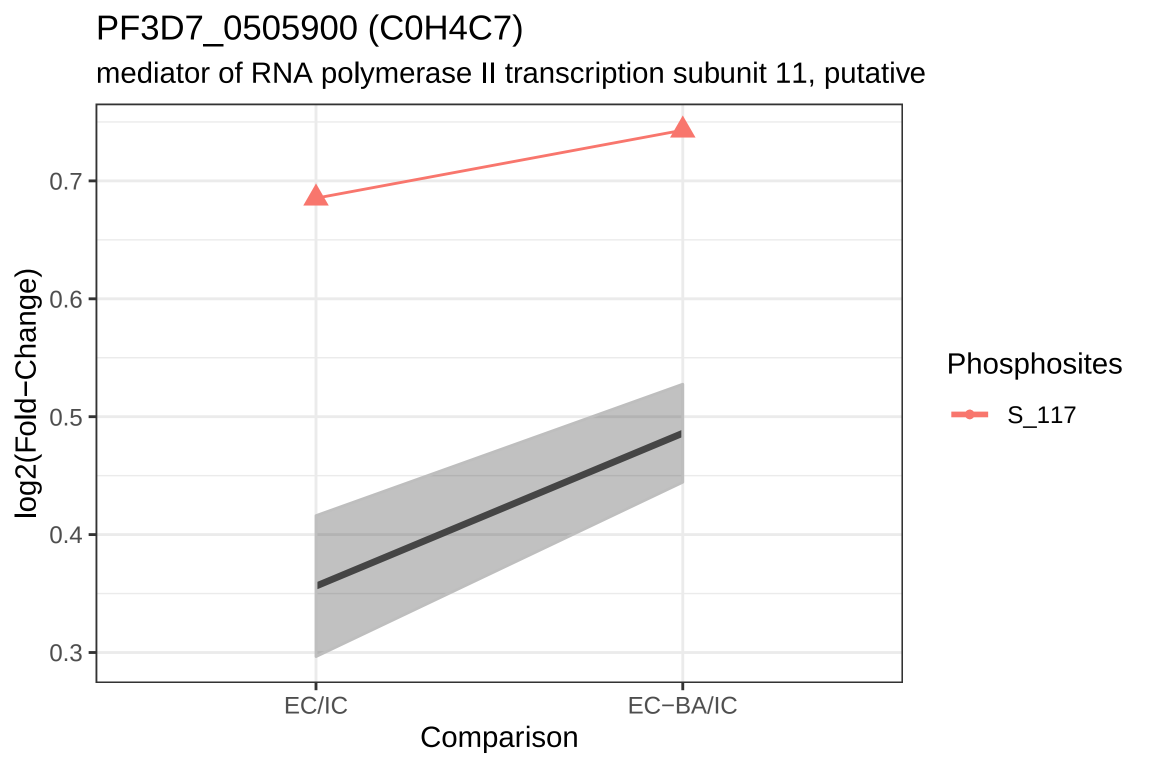

PF3D7_0505900 (C0H4C7)
mediator of RNA polymerase II transcription subunit 11, putative
log2(Fold−Change)
0.7
0.6
0.5
0.4
0.3
EC/IC
EC−BA/IC
Comparison
Phosphosites
S_117

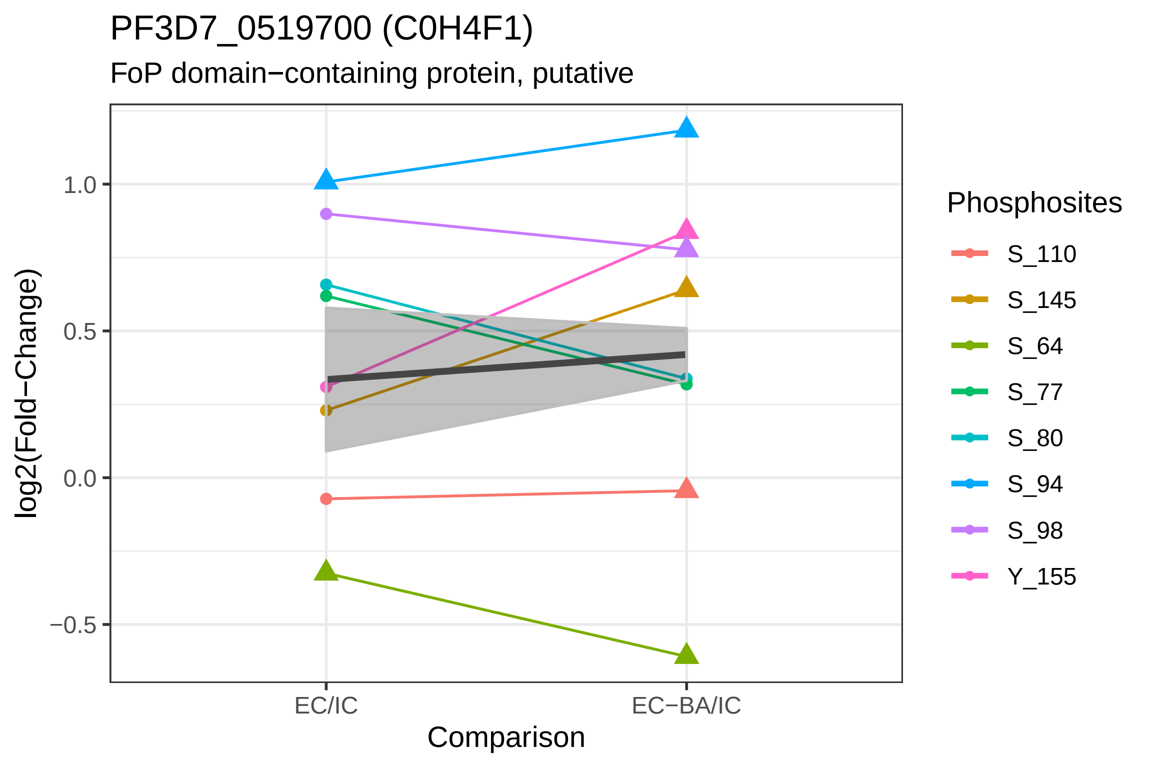

PF3D7_0519700 (C0H4F1)
FoP domain−containing protein, putative
log2(Fold−Change)
1.0
0.5
0.0
−0.5
EC/IC
EC−BA/IC
Comparison
Phosphosites
S_110
S_145
S_64
S_77
S_80
S_94
S_98
Y_155

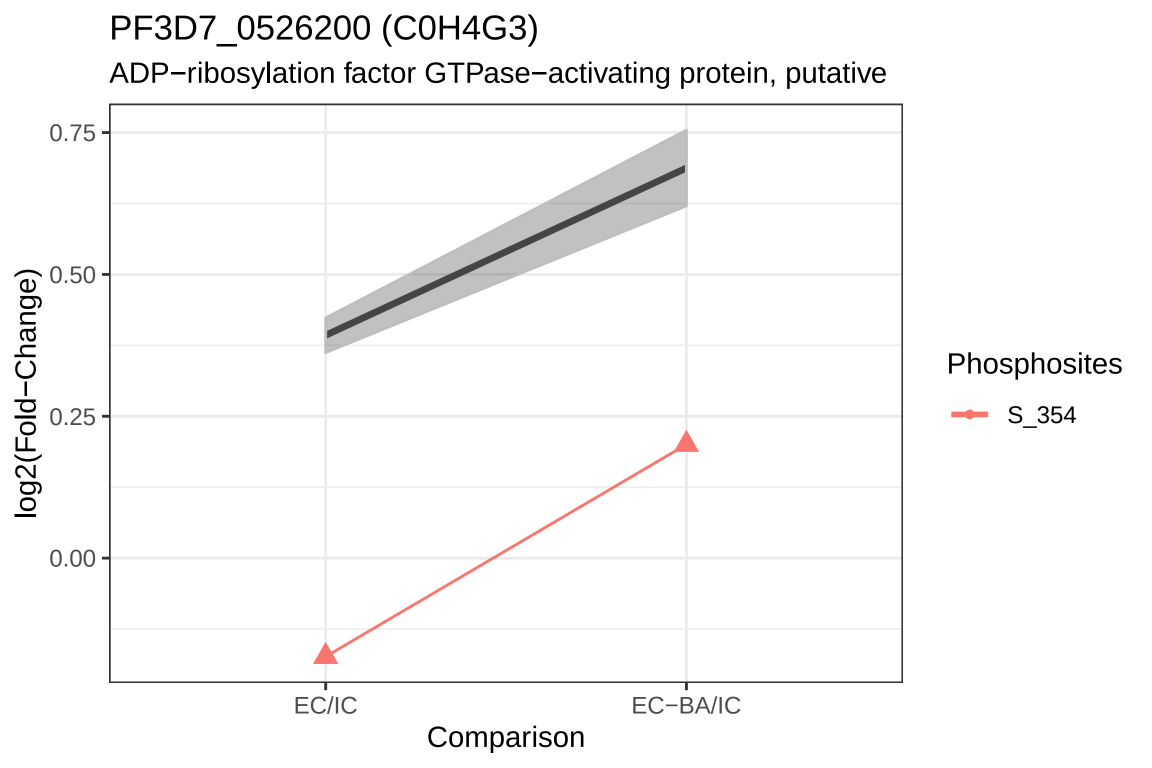
PF3D7_0526200 (C0H4G3)
ADP−ribosylation factor GTPase−activating protein, putative
log2(Fold−Change)
0.75
0.50
0.25
0.00
EC/IC
EC−BA/IC
Comparison
Phosphosites
S_354

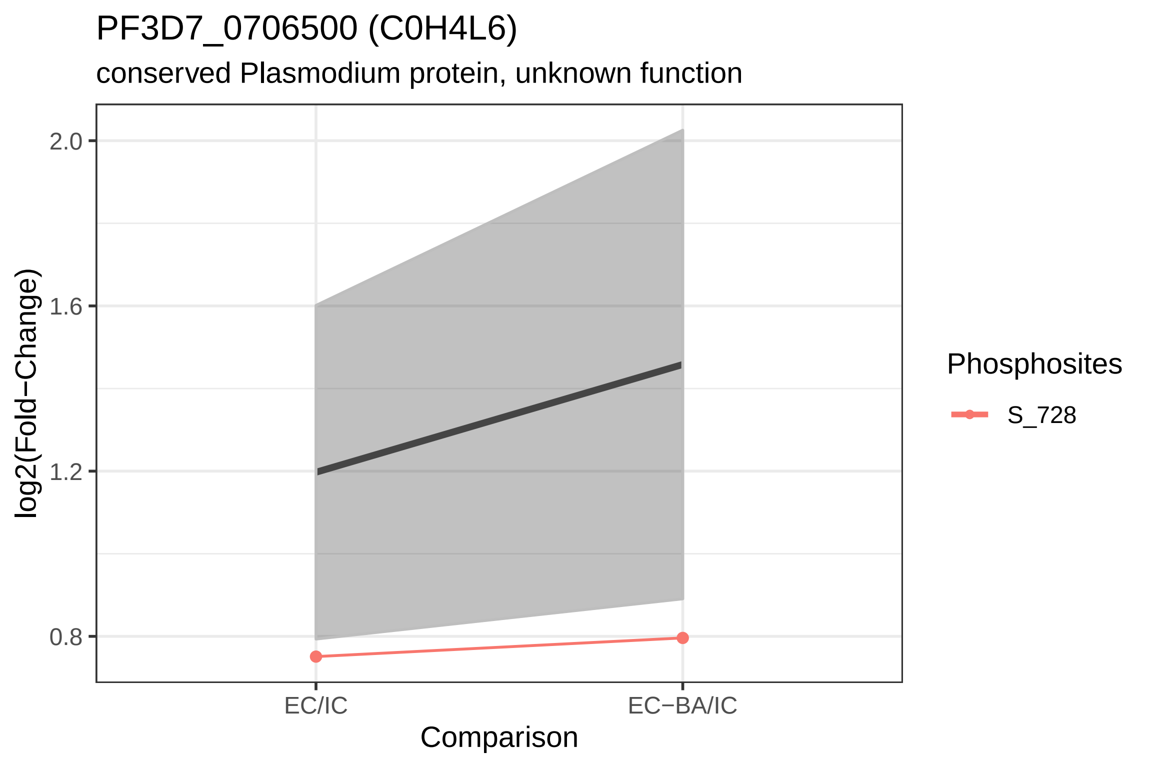
PF3D7_0706500 (C0H4L6)
conserved Plasmodium protein, unknown function
log2(Fold−Change)
2.0
1.6
1.2
0.8
EC/IC
EC−BA/IC
Comparison
Phosphosites
S_728

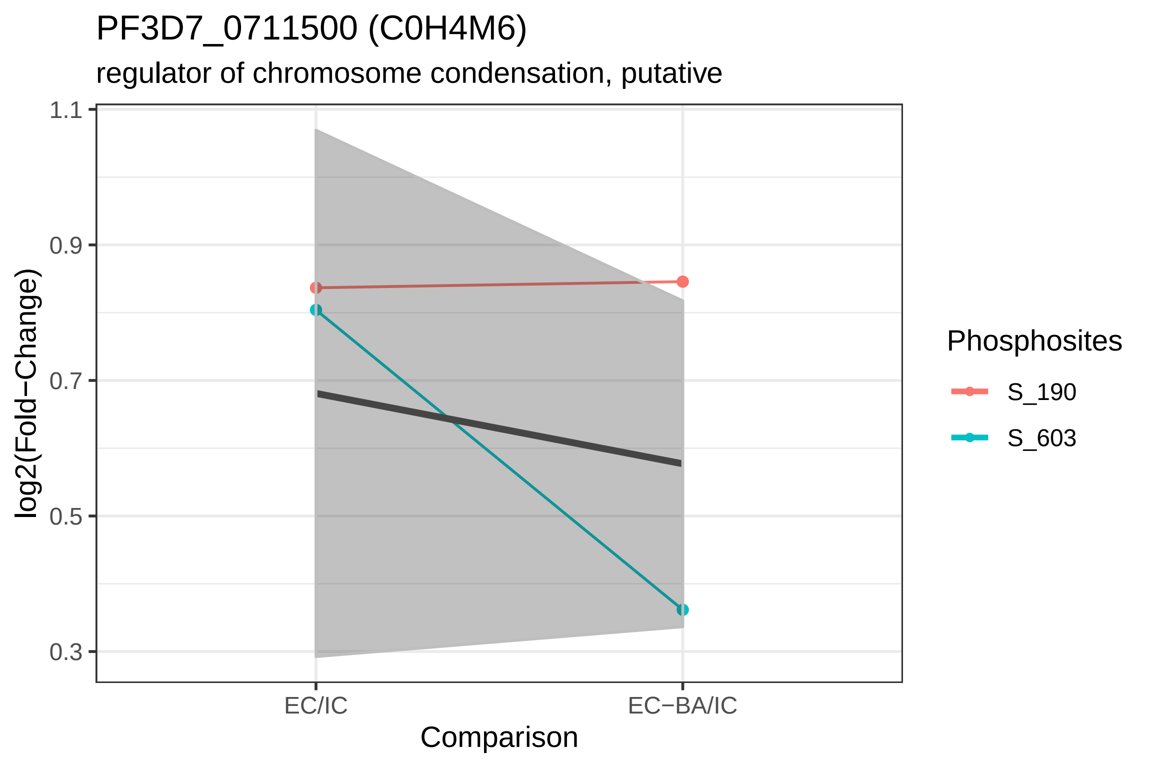

PF3D7_0711500 (C0H4M6)
regulator of chromosome condensation, putative
1.1
0.9
0.7
0.5
0.3
log2(Fold−Change)
EC/IC
EC−BA/IC
Comparison
Phosphosites
S_190
S_603

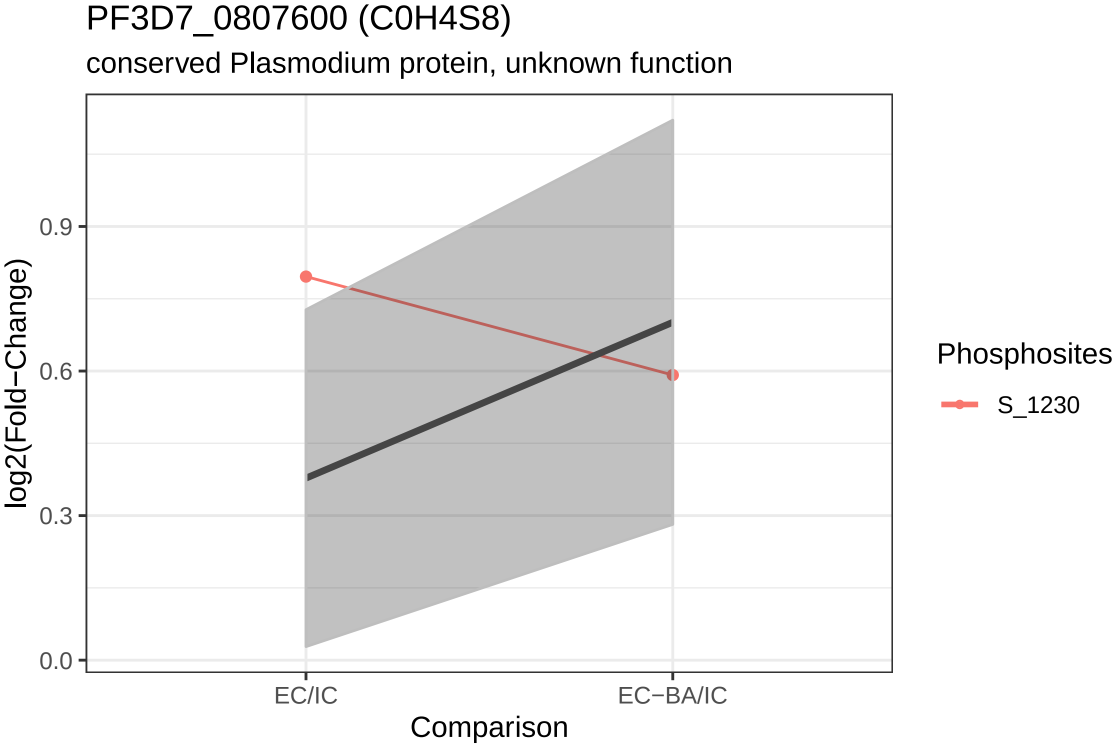
PF3D7_0807600 (C0H4S8)
conserved Plasmodium protein, unknown function
log2(Fold−Change)
0.0
0.3
0.6
0.9
EC/IC
EC−BA/IC
Comparison
Phosphosites
S_1230

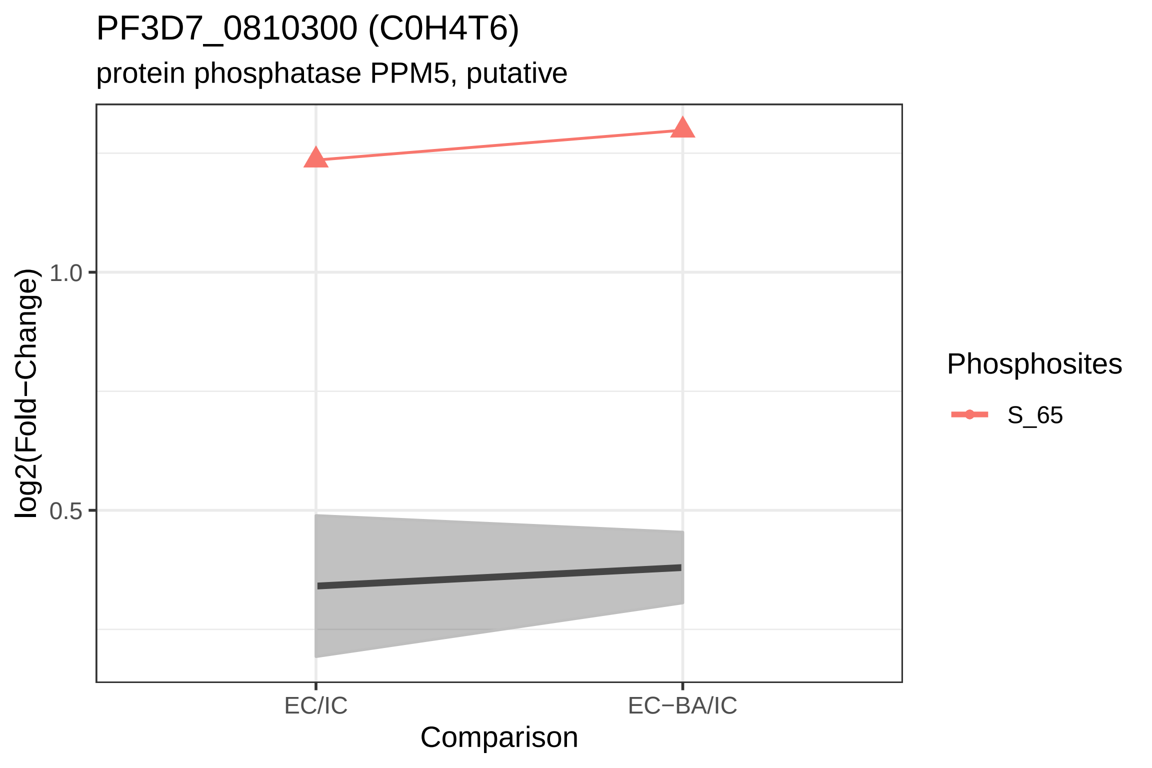
PF3D7_0810300 (C0H4T6)
protein phosphatase PPM5, putative
log2(Fold−Change)
1.0
0.5
EC/IC
EC−BA/IC
Comparison
Phosphosites
S_65

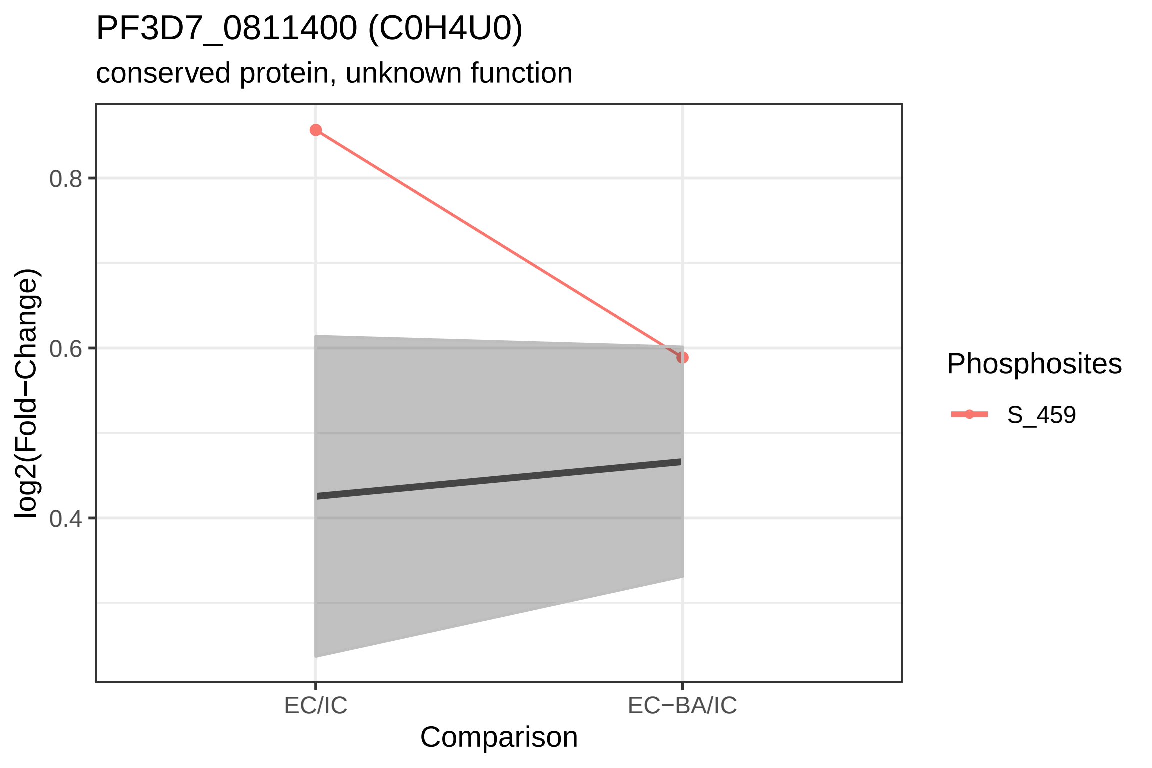
PF3D7_0811400 (C0H4U0)
conserved protein, unknown function
log2(Fold−Change)
0.8
0.6
0.4
EC/IC
EC−BA/IC
Comparison
Phosphosites
S_459

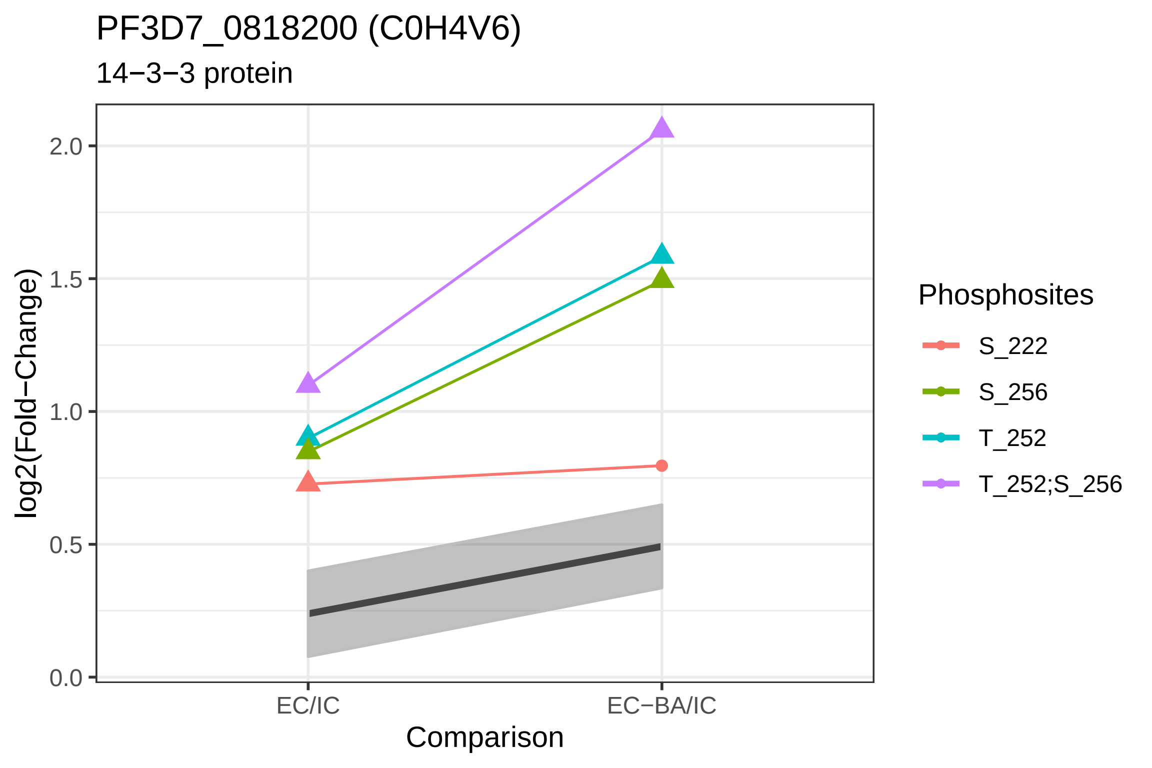
PF3D7_0818200 (C0H4V6)
14−3−3 protein
log2(Fold−Change)
2.0
1.5
1.0
0.5
0.0
EC/IC
EC−BA/IC
Comparison
Phosphosites
S_222
S_256
T_252
T_252;S_256

PF3D7_0821800 (C0H4W6)

protein transport protein SEC61 subunit beta, putative

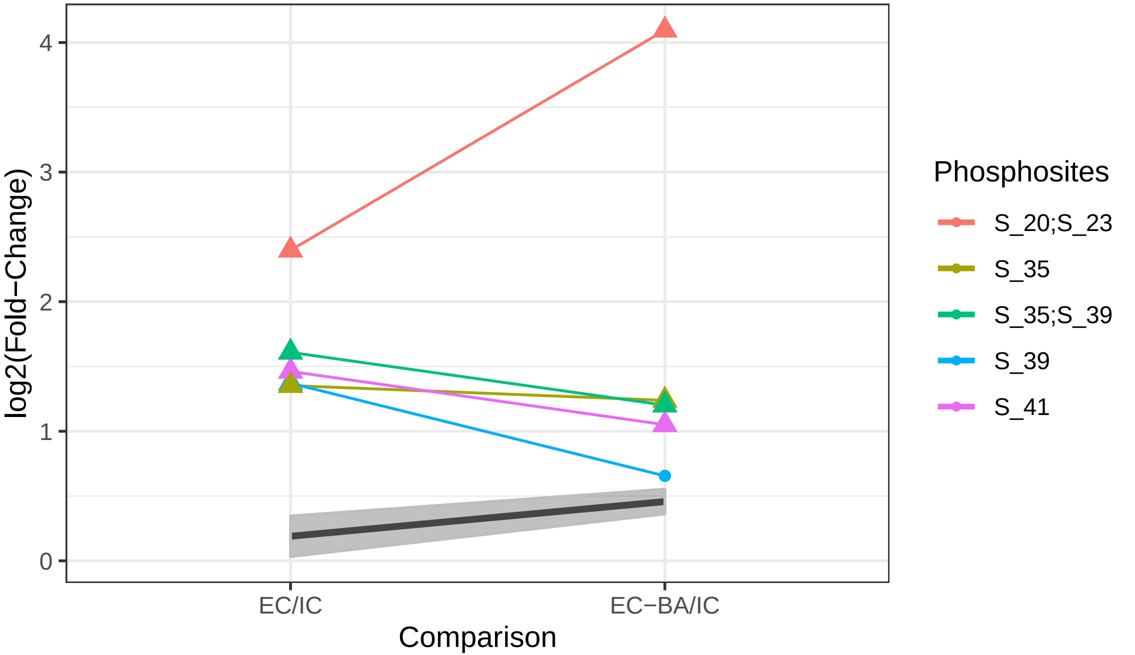

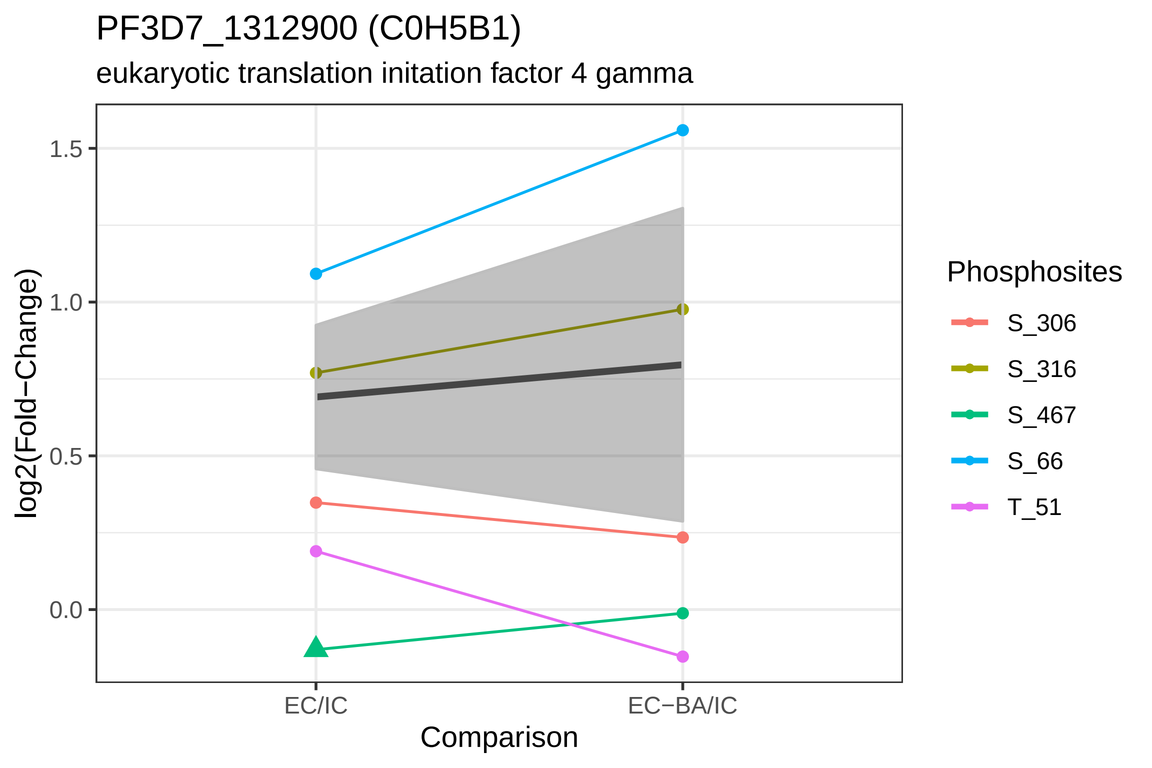

PF3D7_1312900 (C0H5B1)
eukaryotic translation initation factor 4 gamma
log2(Fold−Change)
1.5
1.0
0.5
0.0
EC/IC
EC−BA/IC
Comparison
Phosphosites
S_306
S_316
S_467
S_66
T_51

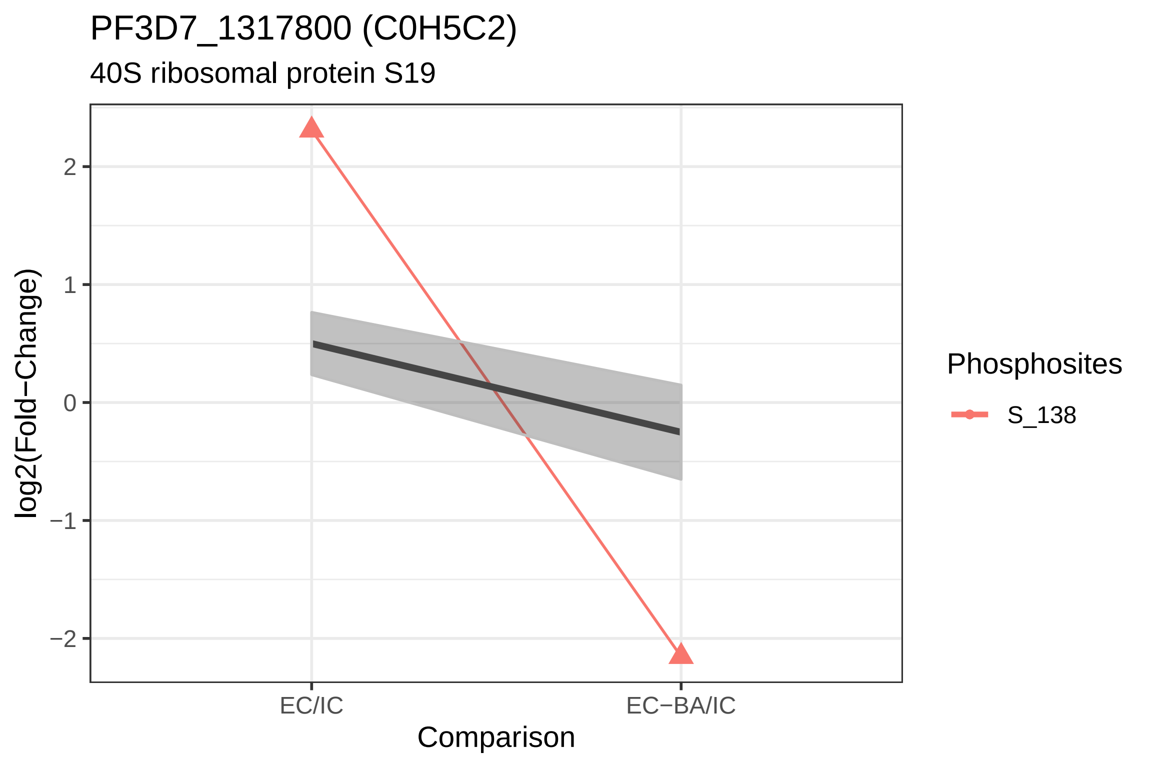
PF3D7_1317800 (C0H5C2)
40S ribosomal protein S19
log2(Fold−Change)
2
1
0
−1
−2
EC/IC
EC−BA/IC
Comparison
Phosphosites
S_138

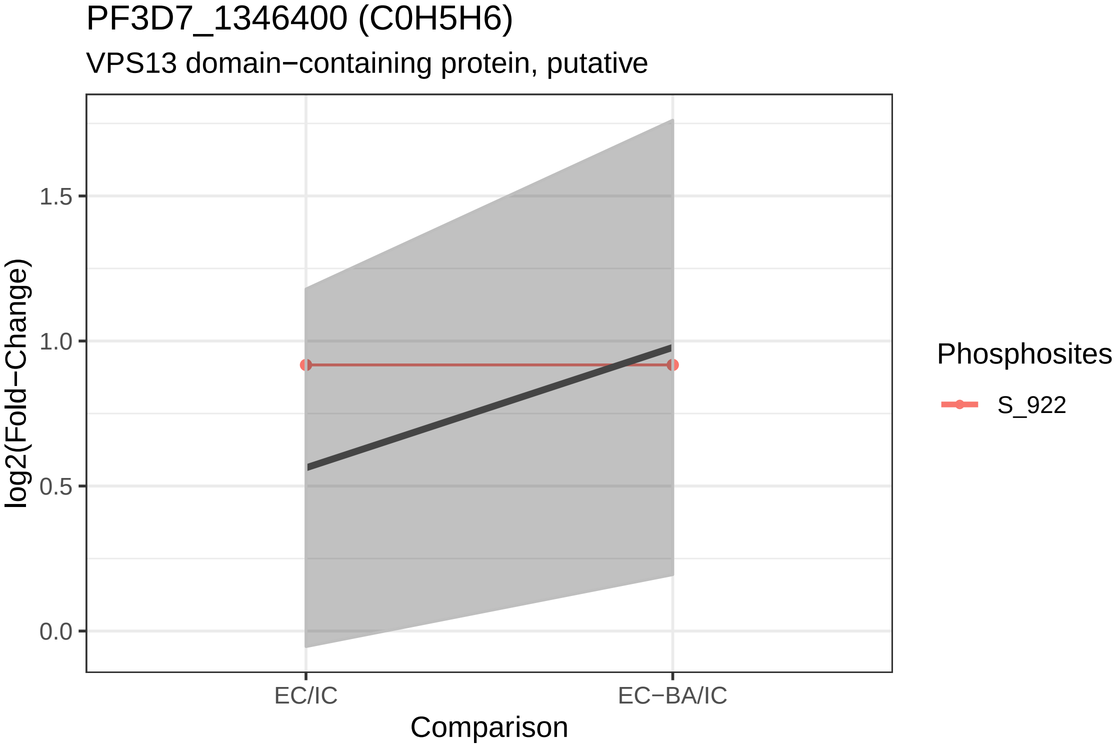
PF3D7_1346400 (C0H5H6)
VPS13 domain−containing protein, putative
log2(Fold−Change)
0.0
0.5
1.0
1.5
EC/IC
EC−BA/IC
Comparison
Phosphosites
S_922

PF3D7_0604500 (C6KSP3)

conserved Plasmodium protein, unknown function

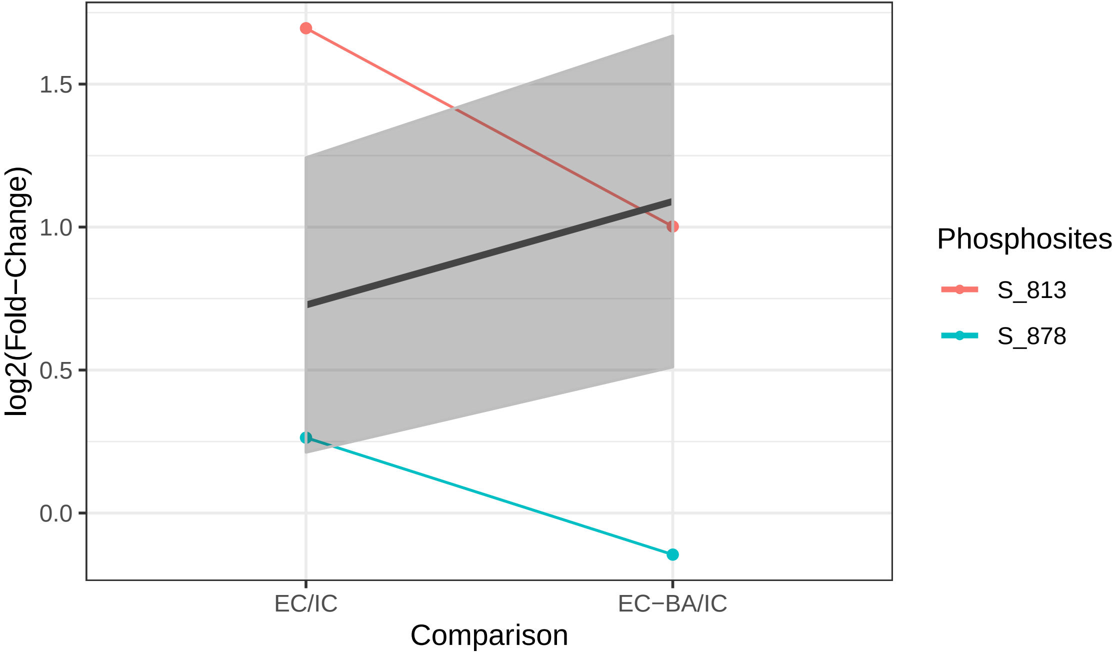

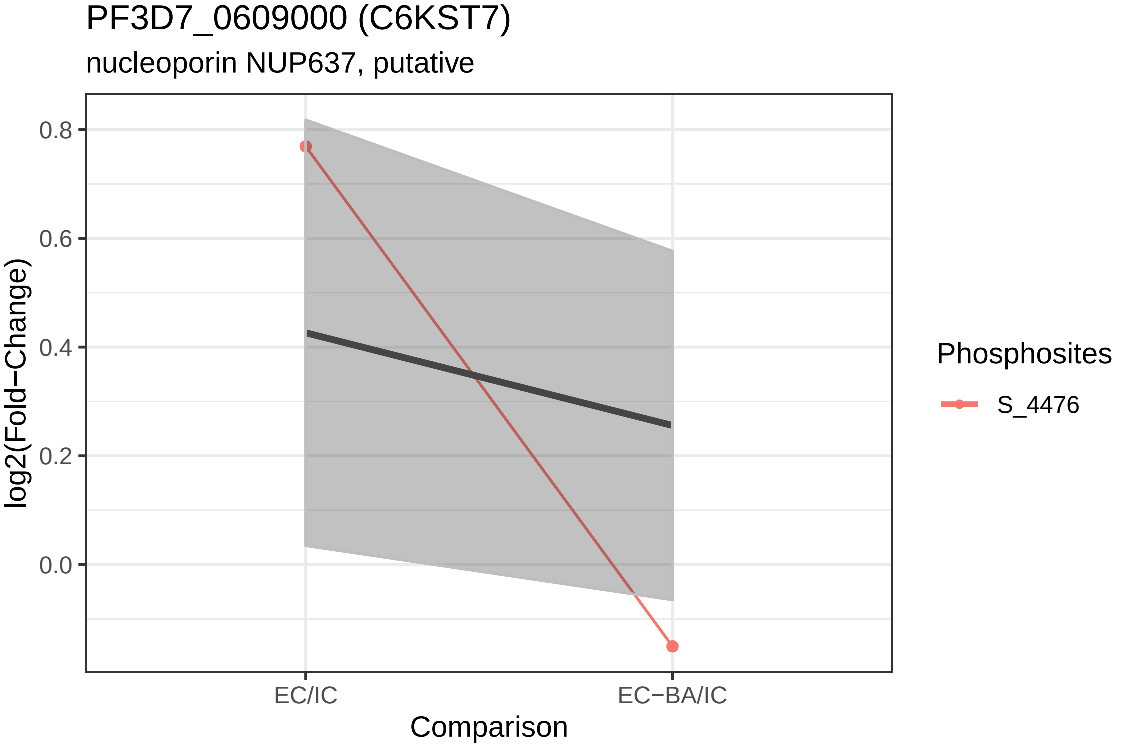
PF3D7_0609000 (C6KST7)
nucleoporin NUP637, putative
log2(Fold−Change)
0.8
0.6
0.4
0.2
0.0
EC/IC
EC−BA/IC
Comparison
Phosphosites
S_4476

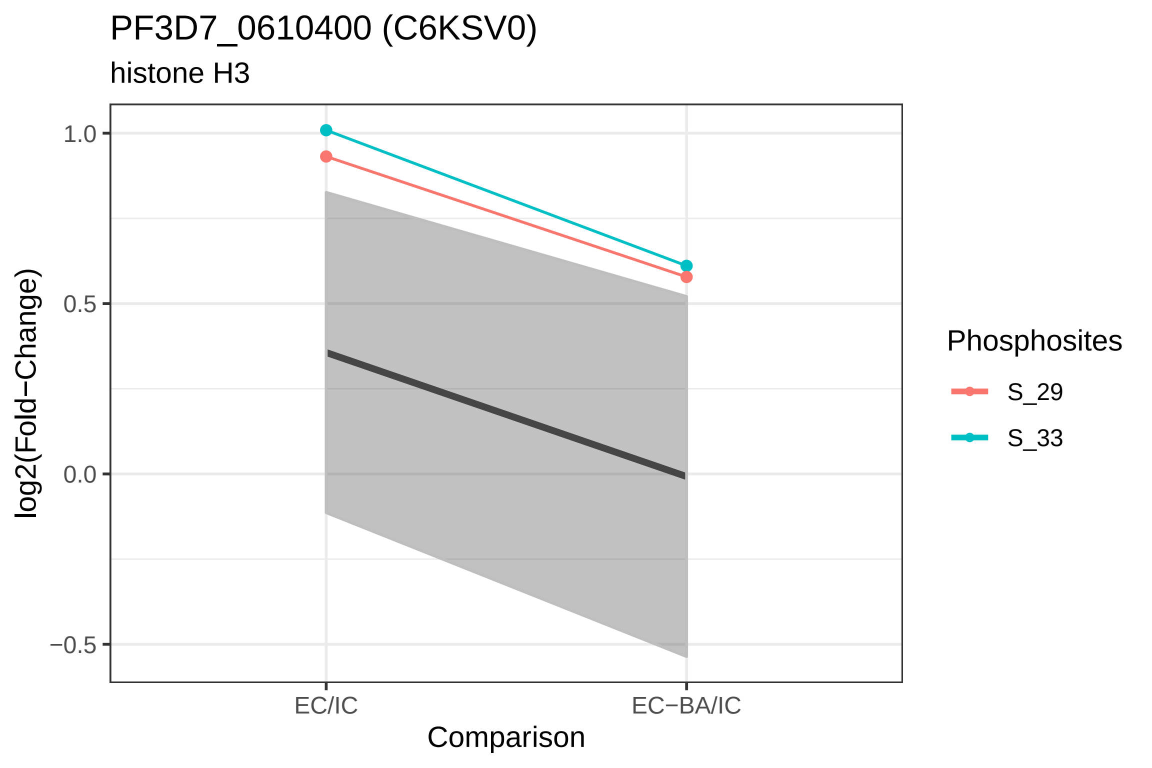
PF3D7_0610400 (C6KSV0)
histone H3
log2(Fold−Change)
1.0
0.5
0.0
−0.5
EC/IC
EC−BA/IC
Comparison
Phosphosites
S_29
S_33

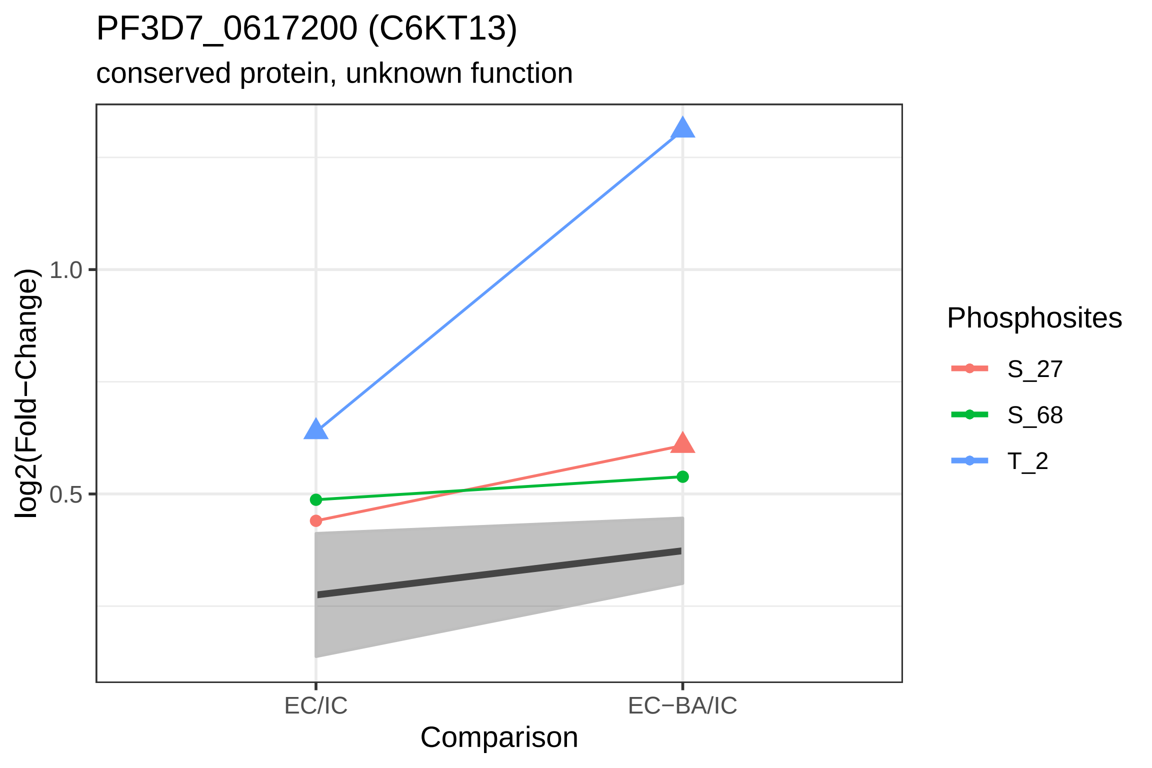

PF3D7_0617200 (C6KT13)
conserved protein, unknown function
log2(Fold−Change)
1.0
0.5
EC/IC
EC−BA/IC
Comparison
Phosphosites
S_27
S_68
T_2

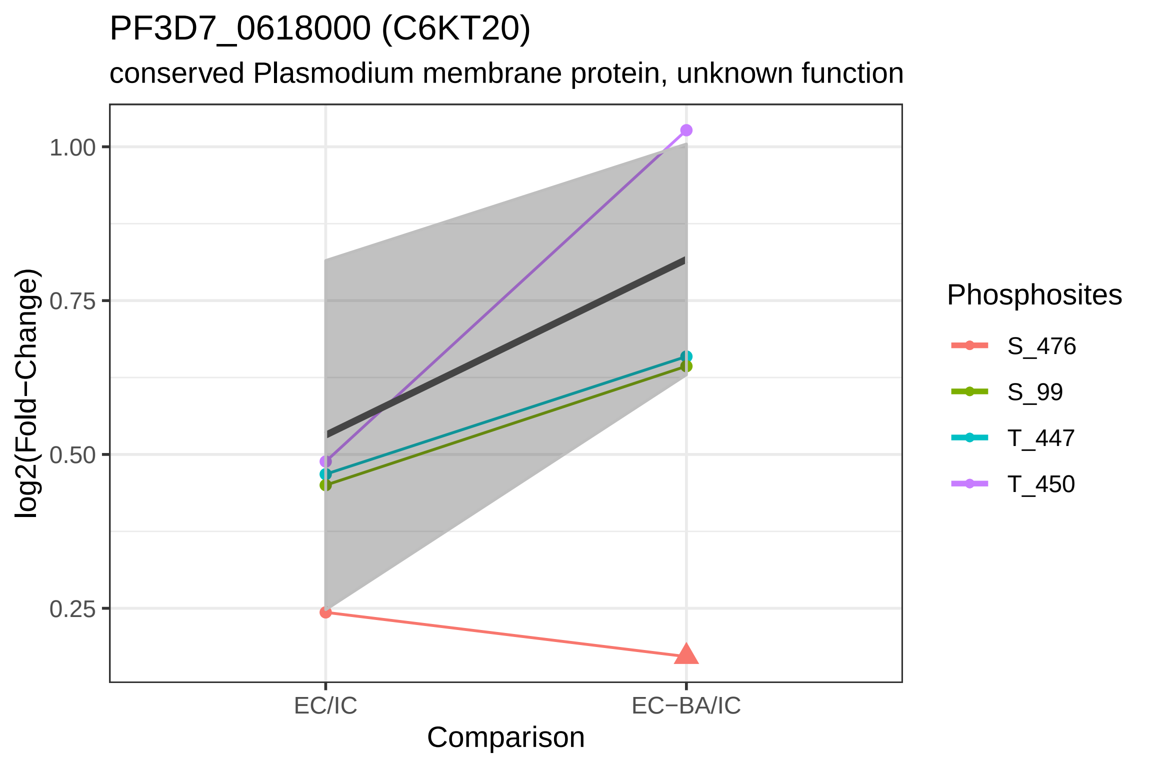
PF3D7_0618000 (C6KT20)
conserved Plasmodium membrane protein, unknown function
1.00
0.75
0.50
0.25
log2(Fold−Change)
EC/IC
EC−BA/IC
Comparison
Phosphosites
S_476
S_99
T_447
T_450

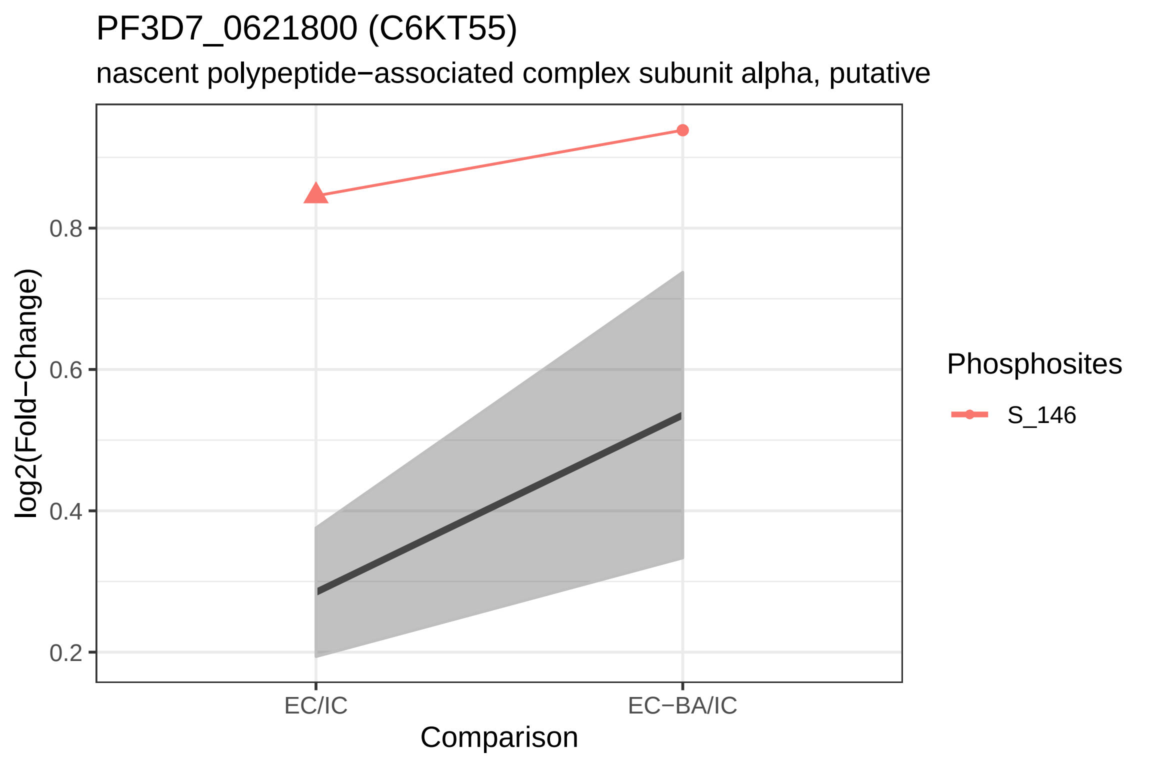
PF3D7_0621800 (C6KT55)
nascent polypeptide−associated complex subunit alpha, putative
log2(Fold−Change)
0.8
0.6
0.4
0.2
EC/IC
EC−BA/IC
Comparison
Phosphosites
S_146

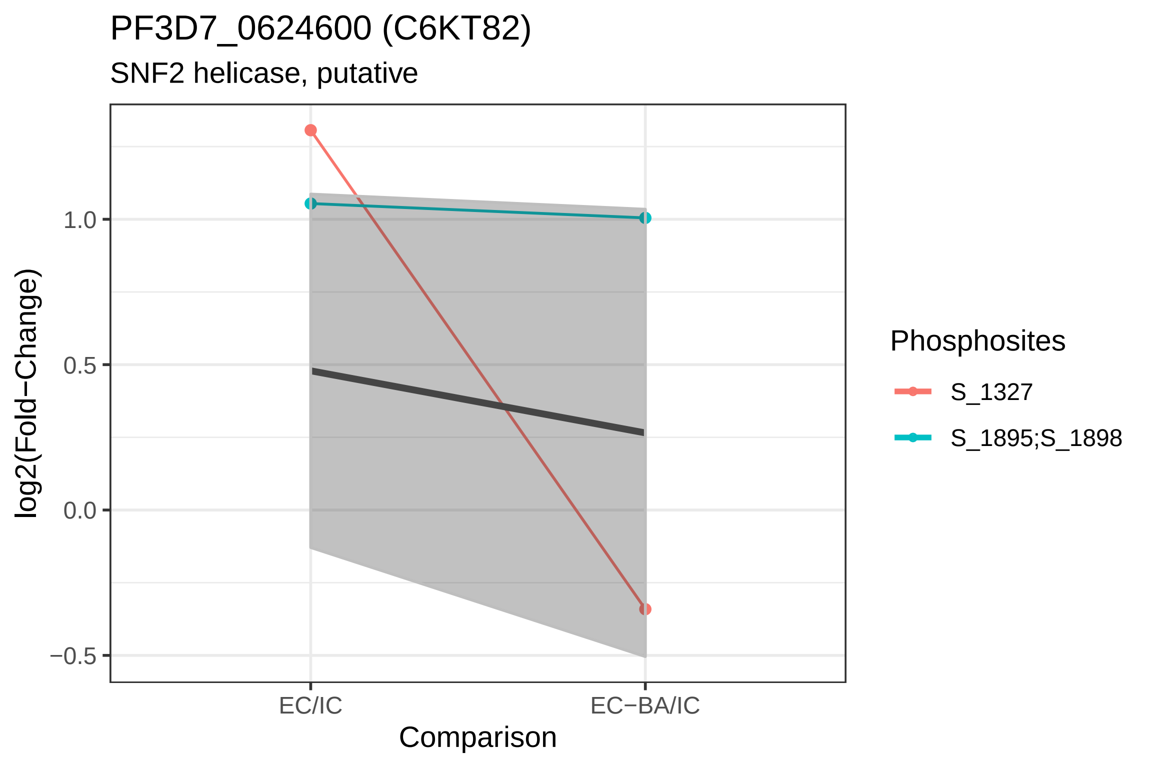
PF3D7_0624600 (C6KT82)
SNF2 helicase, putative
log2(Fold−Change)
1.0
0.5
0.0
−0.5
EC/IC
EC−BA/IC
Comparison
Phosphosites
S_1327
S_1895;S_1898

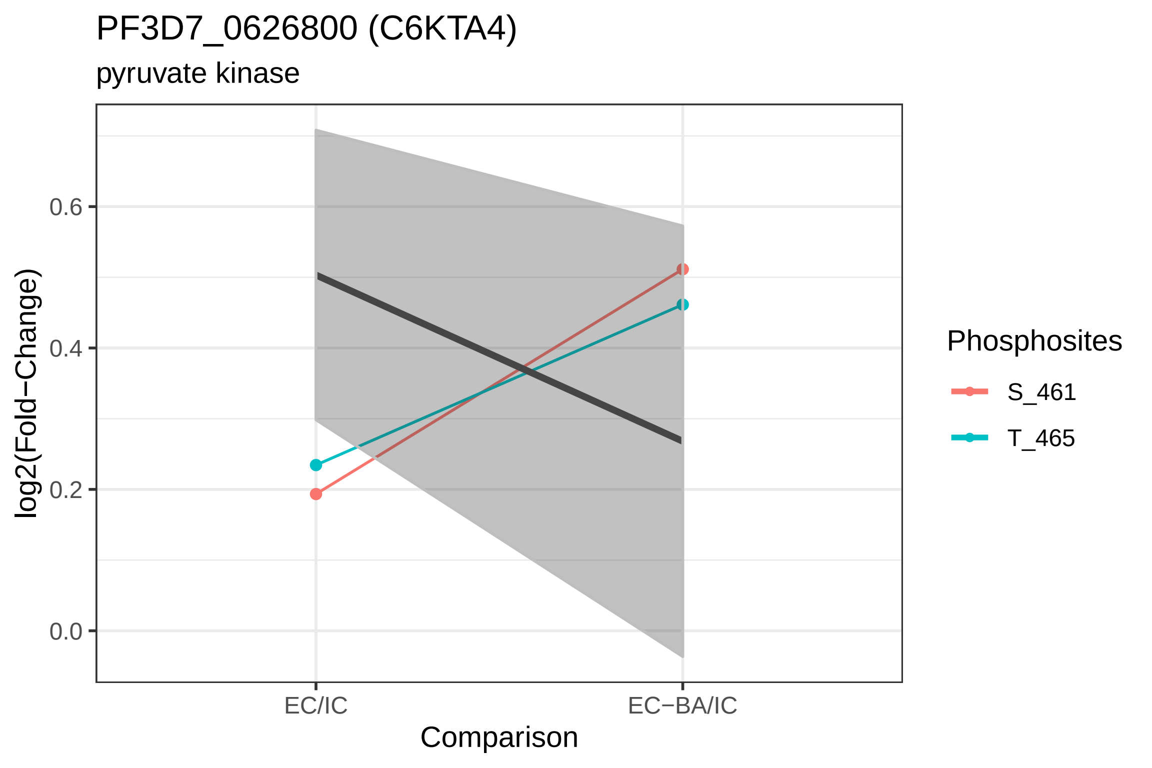
PF3D7_0626800 (C6KTA4)
pyruvate kinase
log2(Fold−Change)
0.0
0.2
0.4
0.6
EC/IC
EC−BA/IC
Comparison
Phosphosites
S_461
T_465

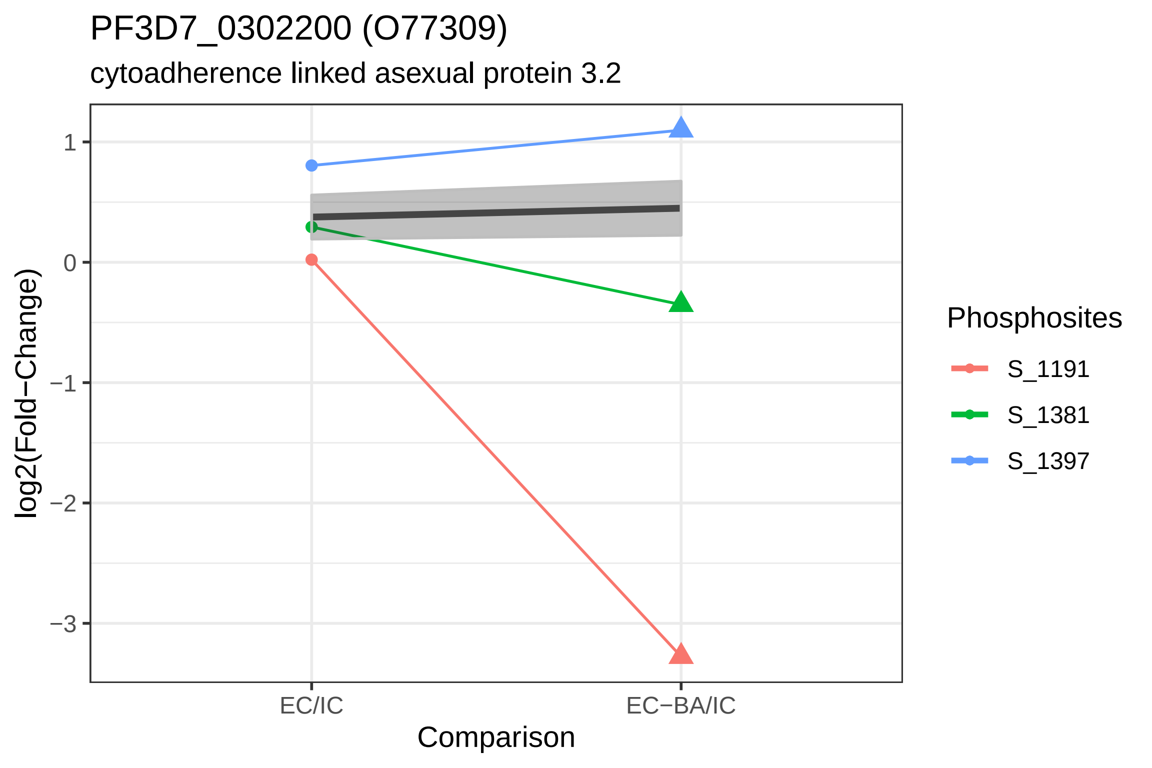
PF3D7_0302200 (O77309)
cytoadherence linked asexual protein 3.2
1
0
−1
−2
−3
log2(Fold−Change)
EC/IC
EC−BA/IC
Comparison
Phosphosites
S_1191
S_1381
S_1397

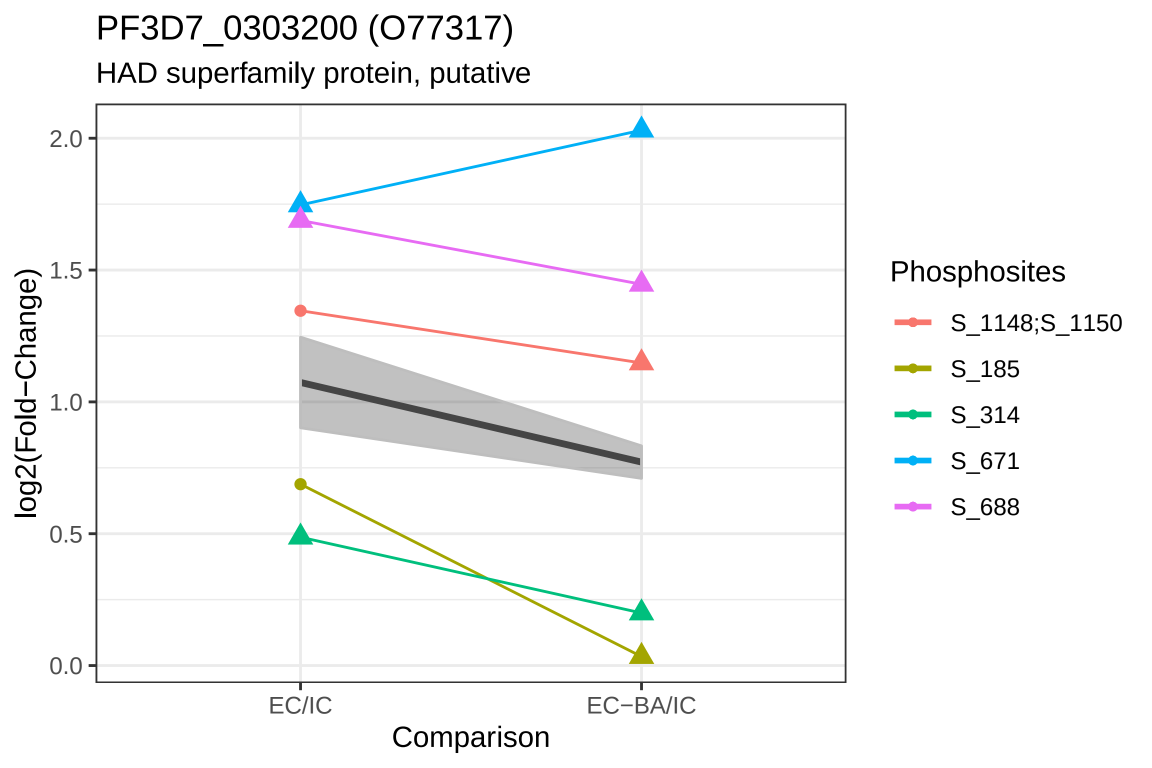
PF3D7_0303200 (O77317)
HAD superfamily protein, putative
log2(Fold−Change)
2.0
1.5
1.0
0.5
0.0
EC/IC
EC−BA/IC
Comparison
Phosphosites
S_1148;S_1150
S_185
S_314
S_671
S_688

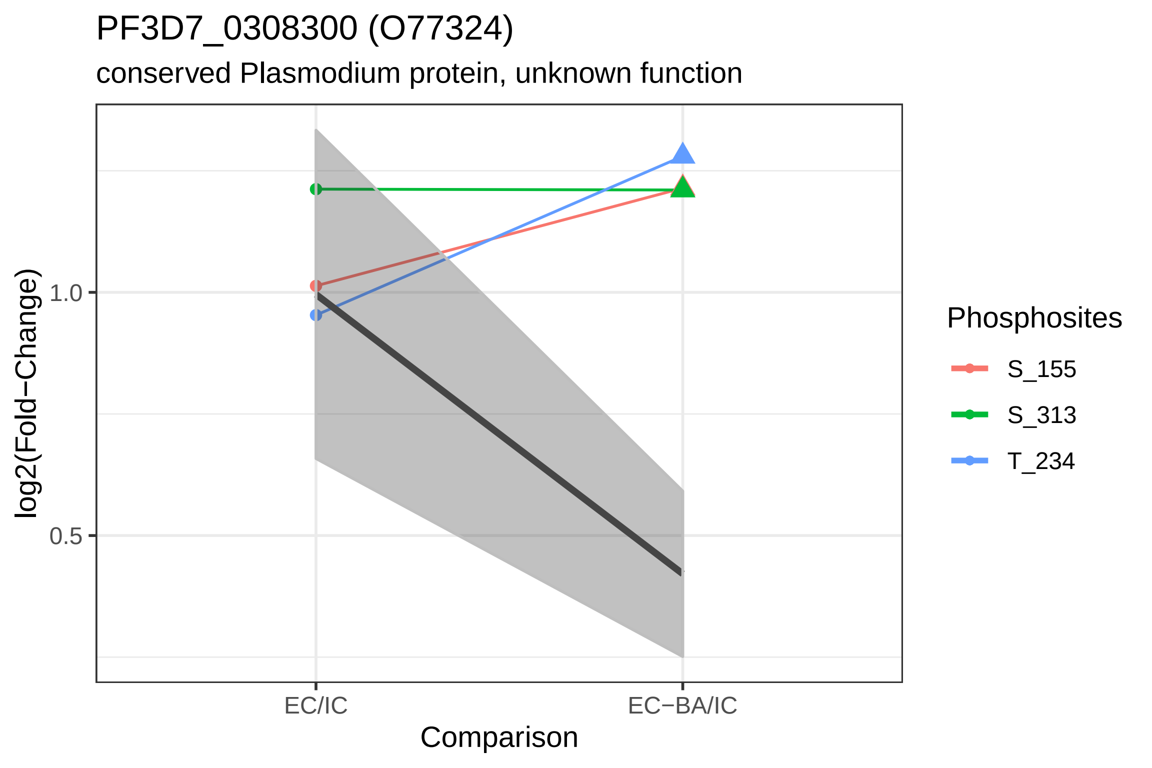
PF3D7_0308300 (O77324)
conserved Plasmodium protein, unknown function
log2(Fold−Change)
1.0
0.5
EC/IC
EC−BA/IC
Comparison
Phosphosites
S_155
S_313
T_234

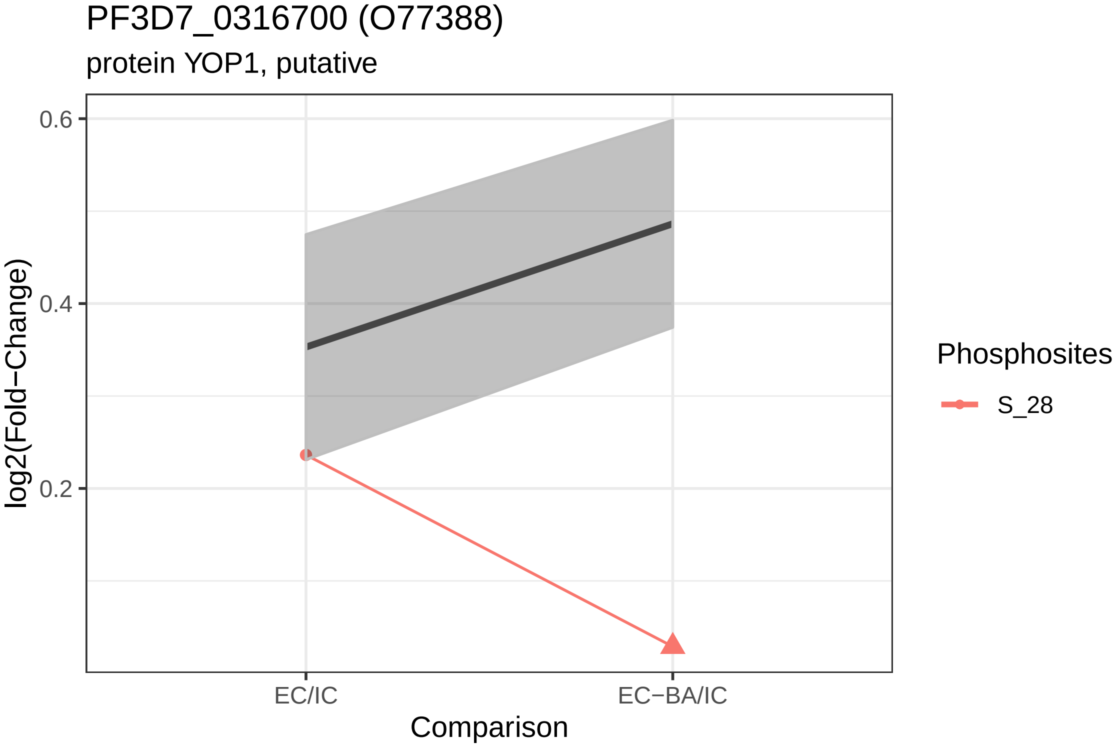
PF3D7_0316700 (O77388)
protein YOP1, putative
0.6
0.4
0.2
log2(Fold−Change)
EC/IC
EC−BA/IC
Comparison
Phosphosites
S_28

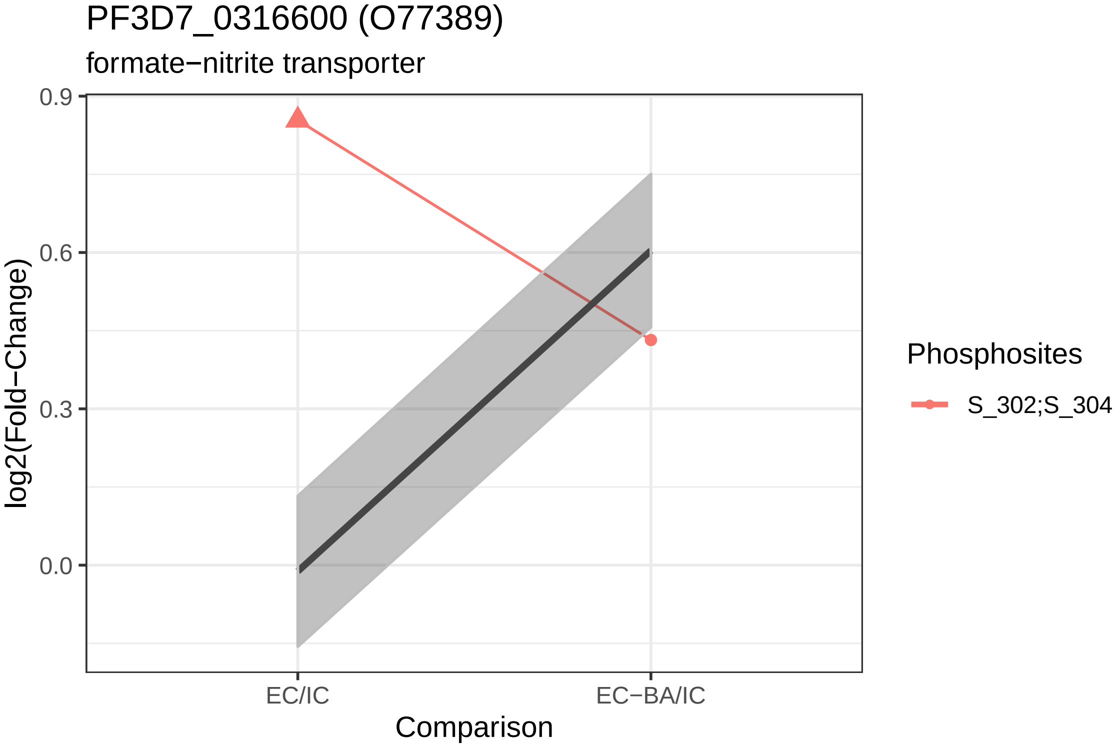

PF3D7_0316600 (O77389)
formate−nitrite transporter
0.9
0.6
0.3
0.0
log2(Fold−Change)
EC/IC
EC−BA/IC
Comparison
Phosphosites
S_302;S_304

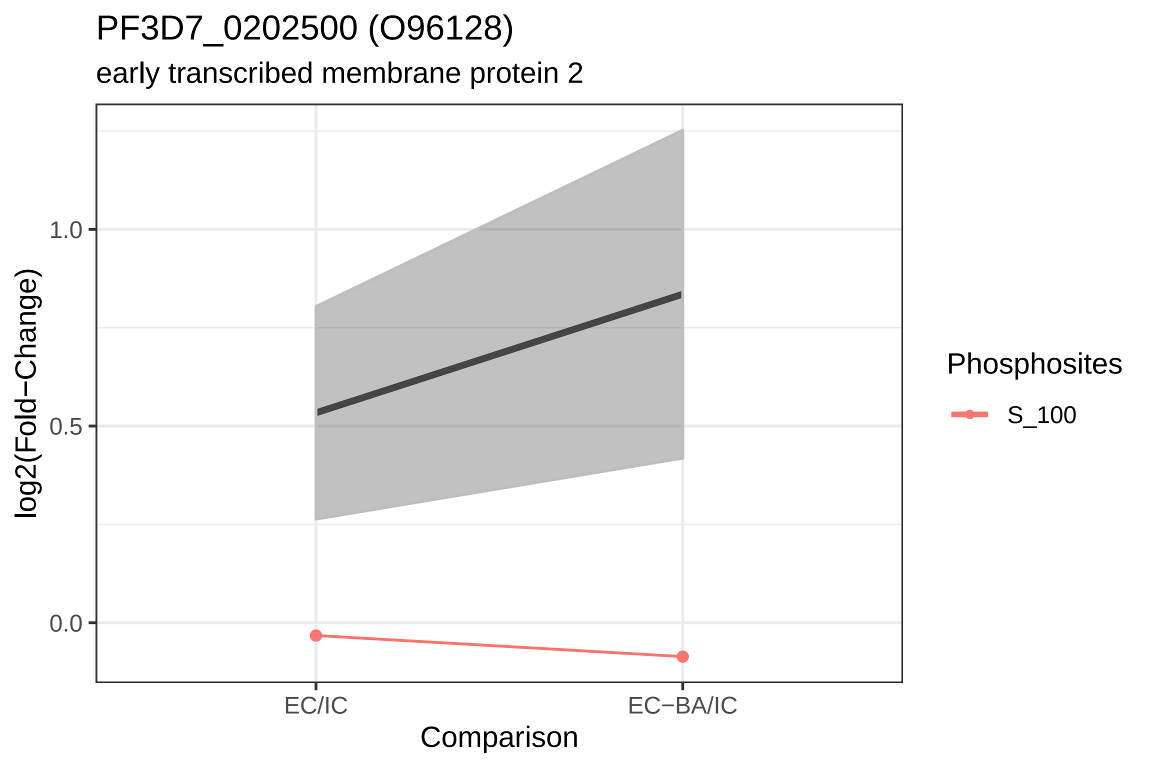
PF3D7_0202500 (O96128)
early transcribed membrane protein 2
log2(Fold−Change)
1.0
0.5
0.0
EC/IC
EC−BA/IC
Comparison
Phosphosites
S_100

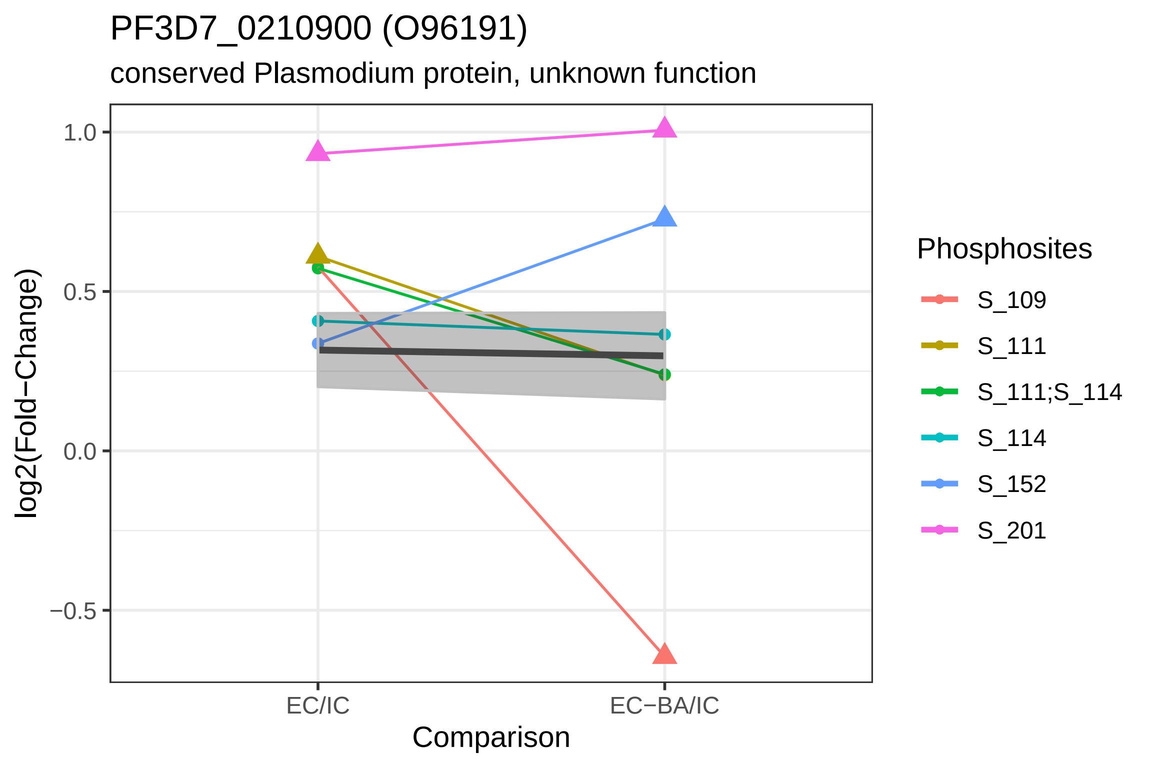
PF3D7_0210900 (O96191)
conserved Plasmodium protein, unknown function
log2(Fold−Change)
1.0
0.5
0.0
−0.5
EC/IC
EC−BA/IC
Comparison
Phosphosites
S_109
S_111
S_111;S_114
S_114
S_152
S_201

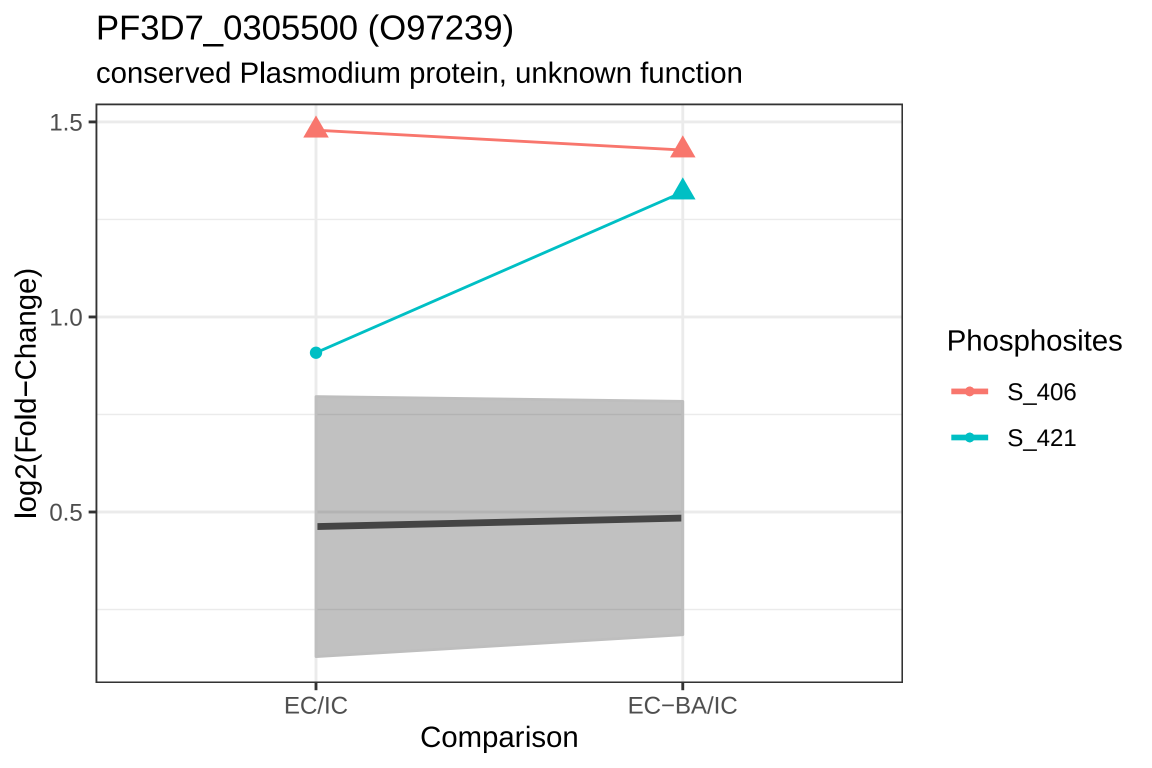

PF3D7_0305500 (O97239)
conserved Plasmodium protein, unknown function
log2(Fold−Change)
1.5
1.0
0.5
EC/IC
EC−BA/IC
Comparison
Phosphosites
S_406
S_421

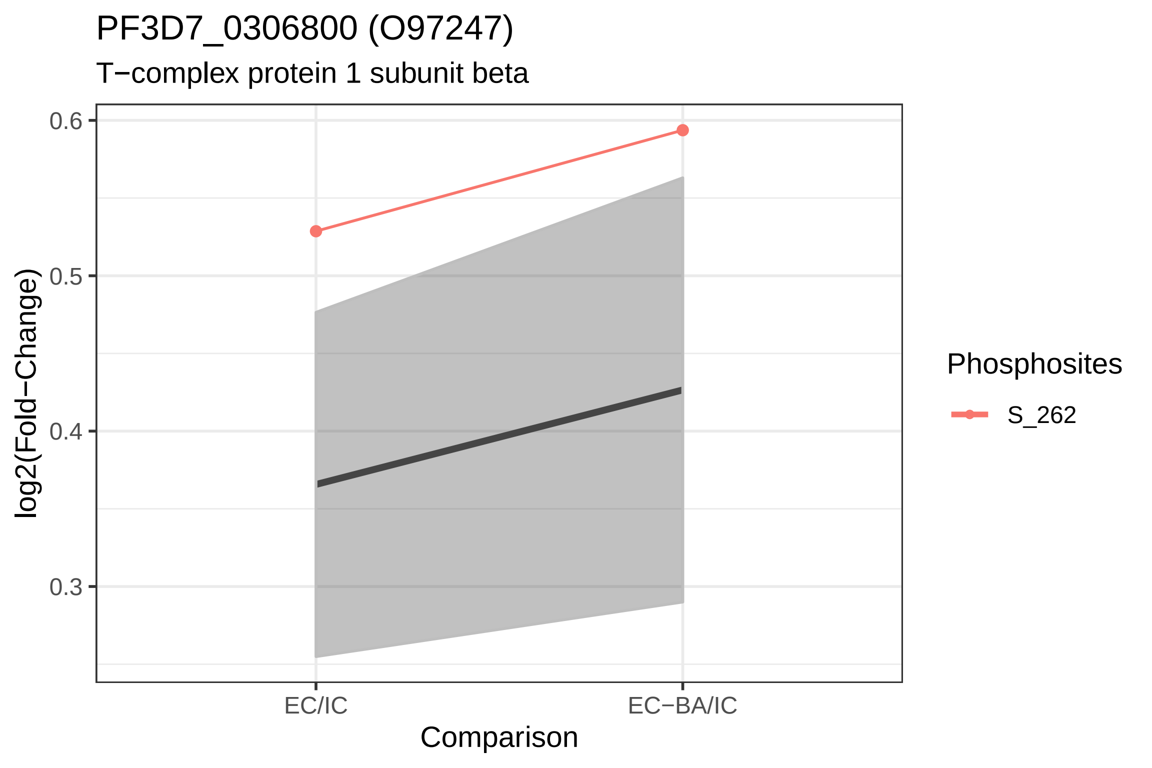

PF3D7_0306800 (O97247)
T−complex protein 1 subunit beta
0.6
0.5
0.4
0.3
log2(Fold−Change)
EC/IC
EC−BA/IC
Comparison
Phosphosites
S_262

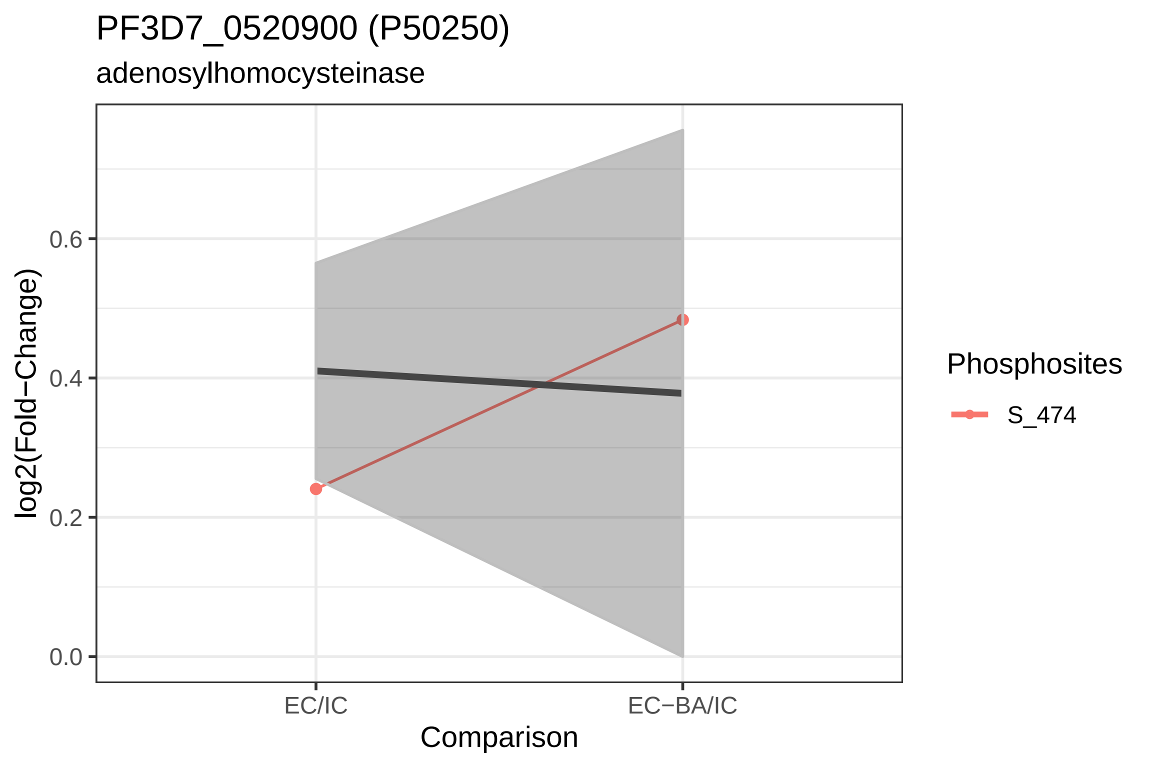

PF3D7_0520900 (P50250)
adenosylhomocysteinase
log2(Fold−Change)
0.6
0.4
0.2
0.0
EC/IC
EC−BA/IC
Comparison
Phosphosites
S_474

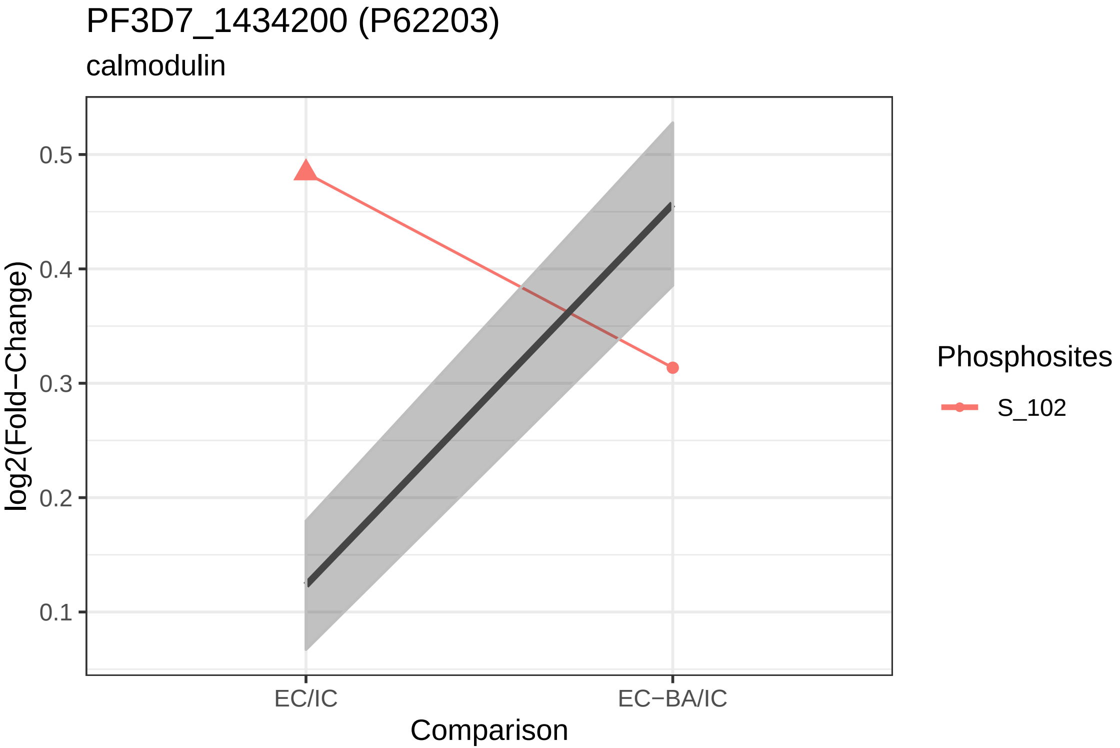

PF3D7_1434200 (P62203)
calmodulin
log2(Fold−Change)
0.5
0.4
0.3
0.2
0.1
EC/IC
EC−BA/IC
Comparison
Phosphosites
S_102

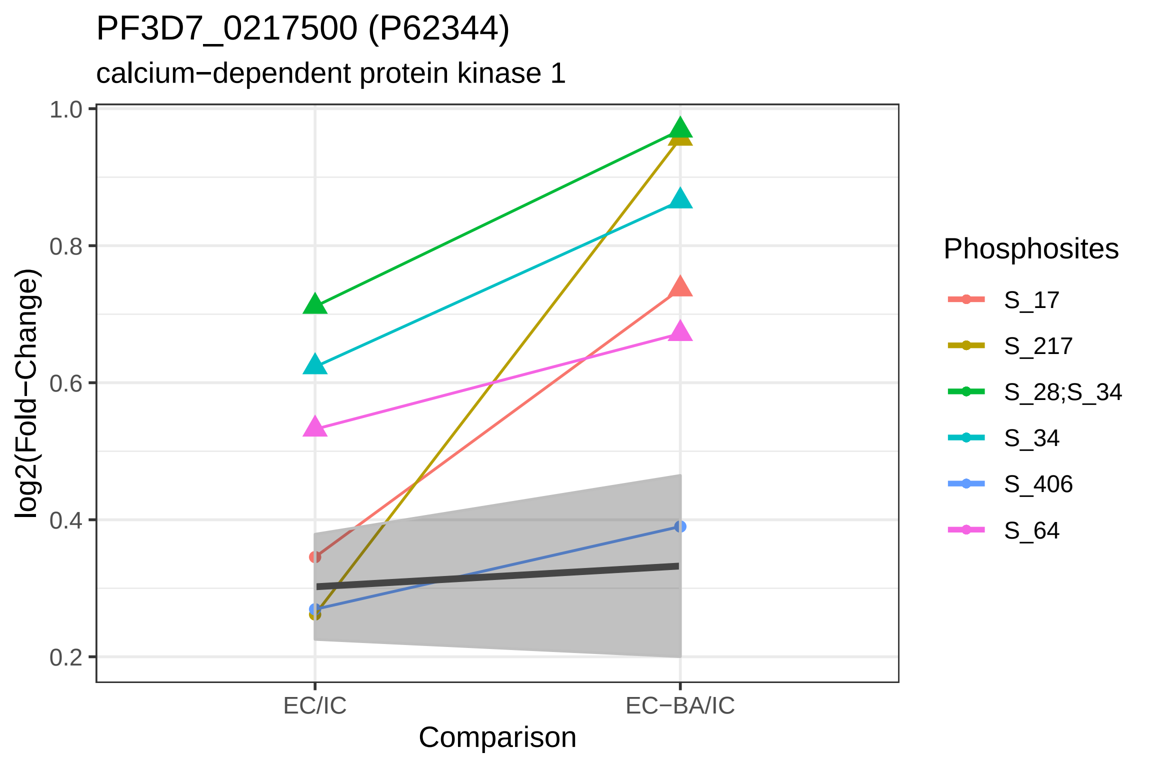

PF3D7_0217500 (P62344)
calcium−dependent protein kinase 1
1.0
0.8
0.6
0.4
0.2
log2(Fold−Change)
EC/IC
EC−BA/IC
Comparison
Phosphosites
S_17
S_217
S_28;S_34
S_34
S_406
S_64

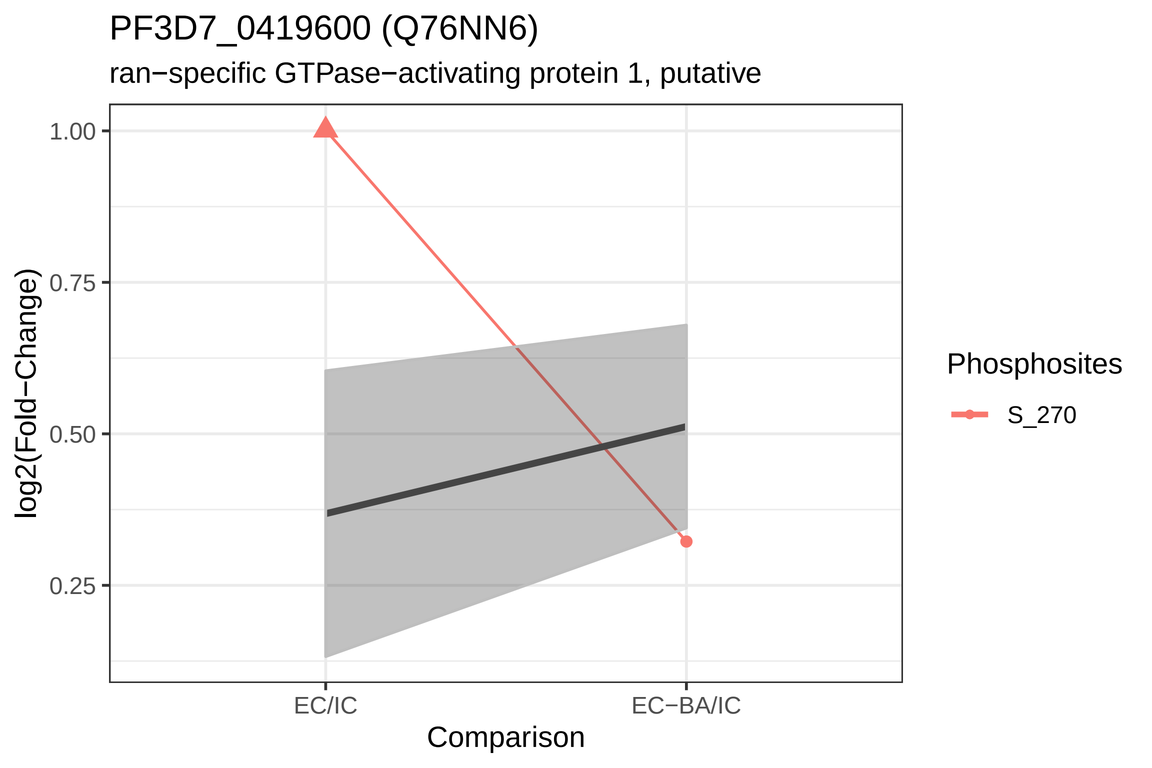
PF3D7_0419600 (Q76NN6)
ran−specific GTPase−activating protein 1, putative
log2(Fold−Change)
1.00
0.75
0.50
0.25
EC/IC
EC−BA/IC
Comparison
Phosphosites
S_270

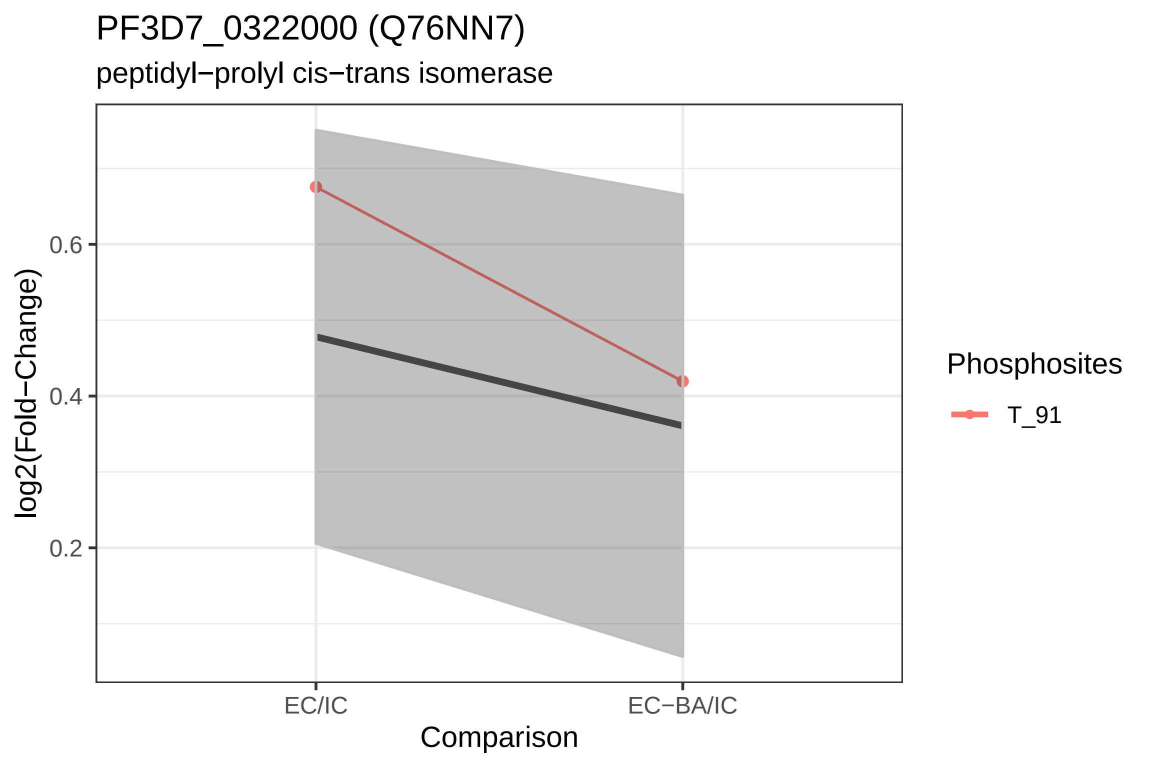

PF3D7_0322000 (Q76NN7)
peptidyl−prolyl cis−trans isomerase
log2(Fold−Change)
0.6
0.4
0.2
EC/IC
EC−BA/IC
Comparison
Phosphosites
T_91

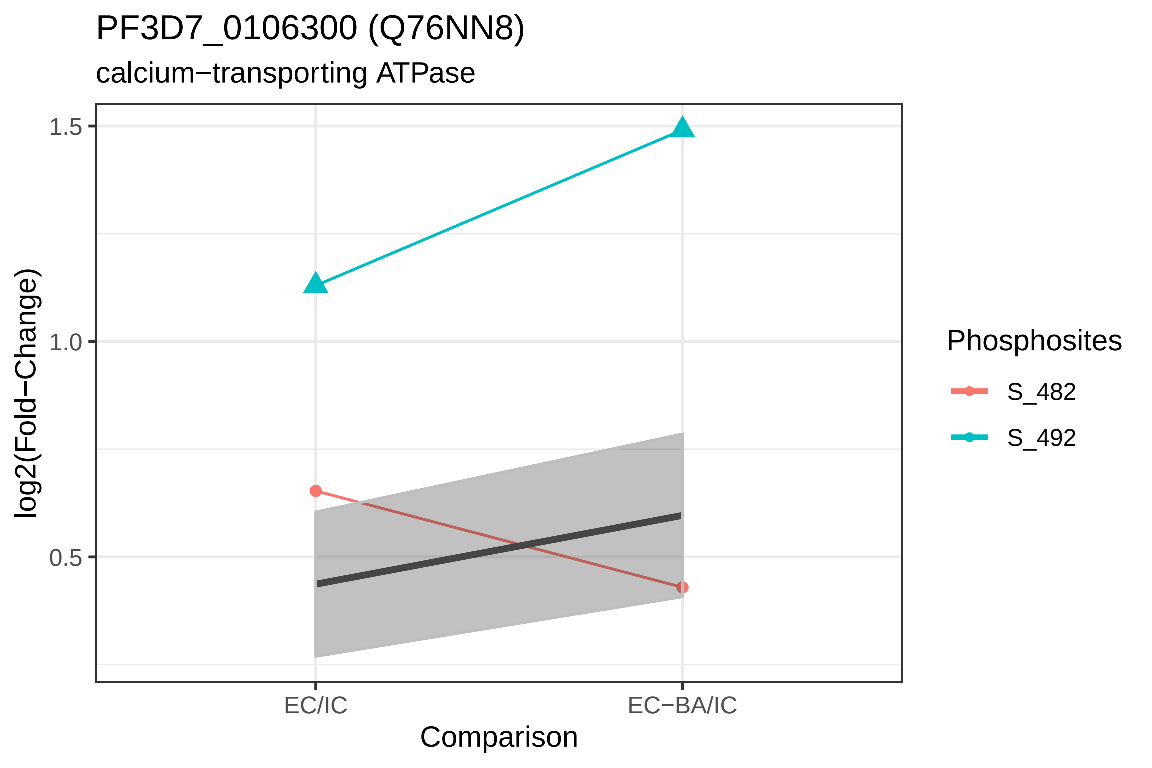
PF3D7_0106300 (Q76NN8)
calcium−transporting ATPase
1.5
1.0
0.5
log2(Fold−Change)
EC/IC
EC−BA/IC
Comparison
Phosphosites
S_482
S_492

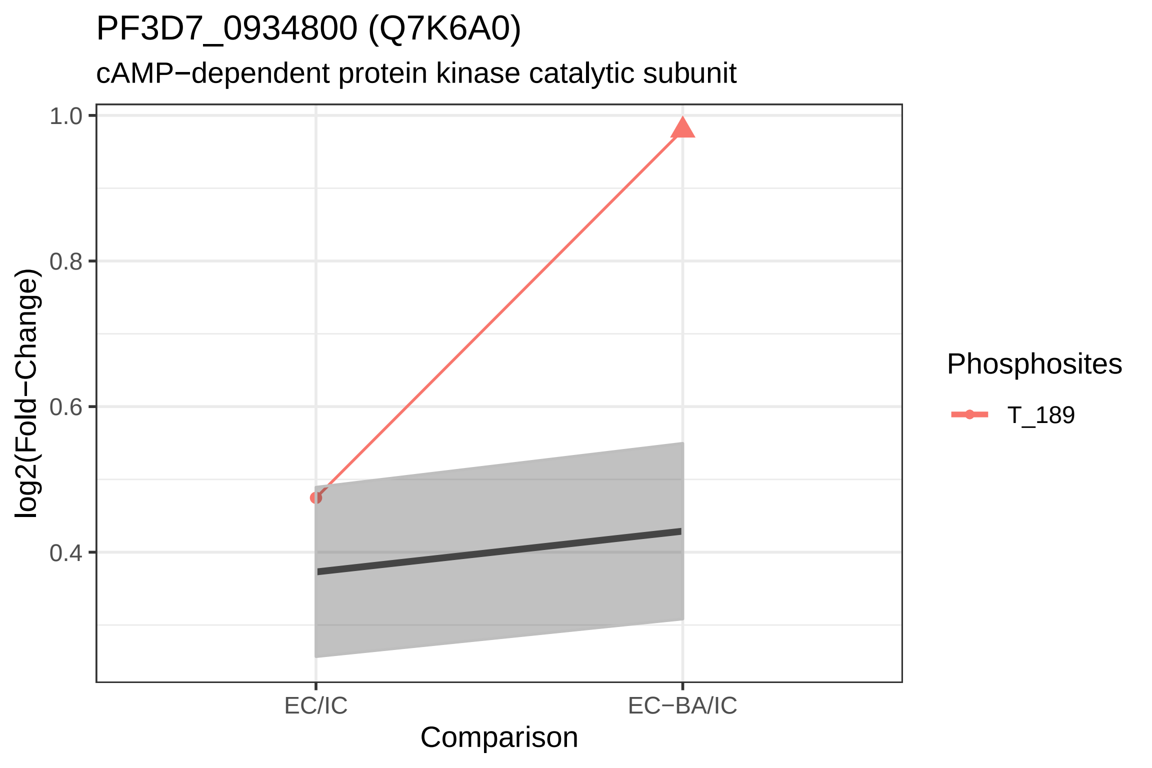
PF3D7_0934800 (Q7K6A0)
cAMP−dependent protein kinase catalytic subunit
log2(Fold−Change)
1.0
0.8
0.6
0.4
EC/IC
EC−BA/IC
Comparison
Phosphosites
T_189

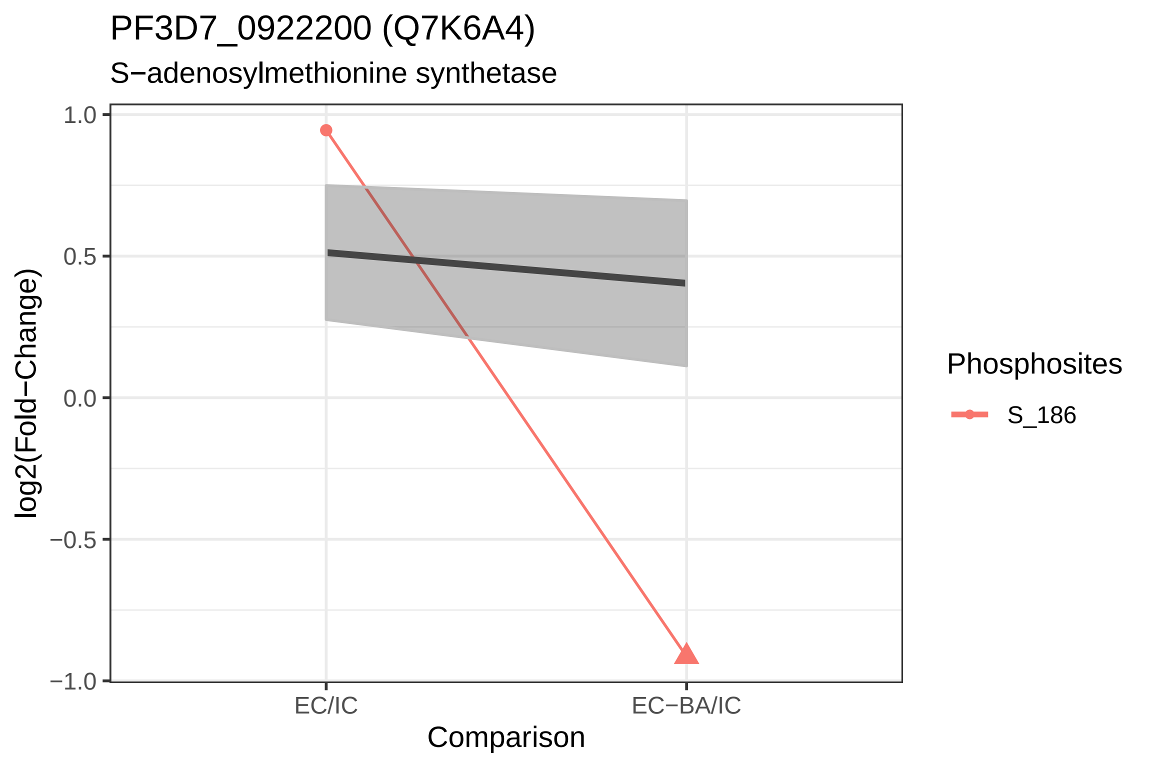

PF3D7_0922200 (Q7K6A4)
S−adenosylmethionine synthetase
log2(Fold−Change)
1.0
0.5
0.0
−0.5
−1.0
EC/IC
EC−BA/IC
Comparison
Phosphosites
S_186

PF3D7_0523000 (Q7K6A5)

multidrug resistance protein 1

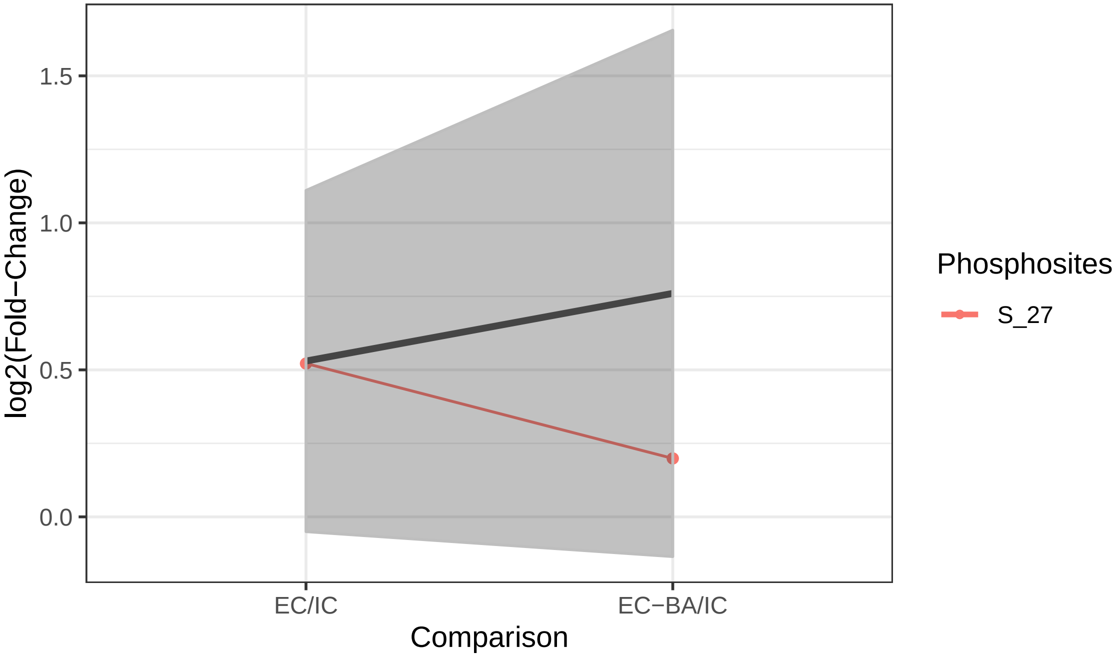

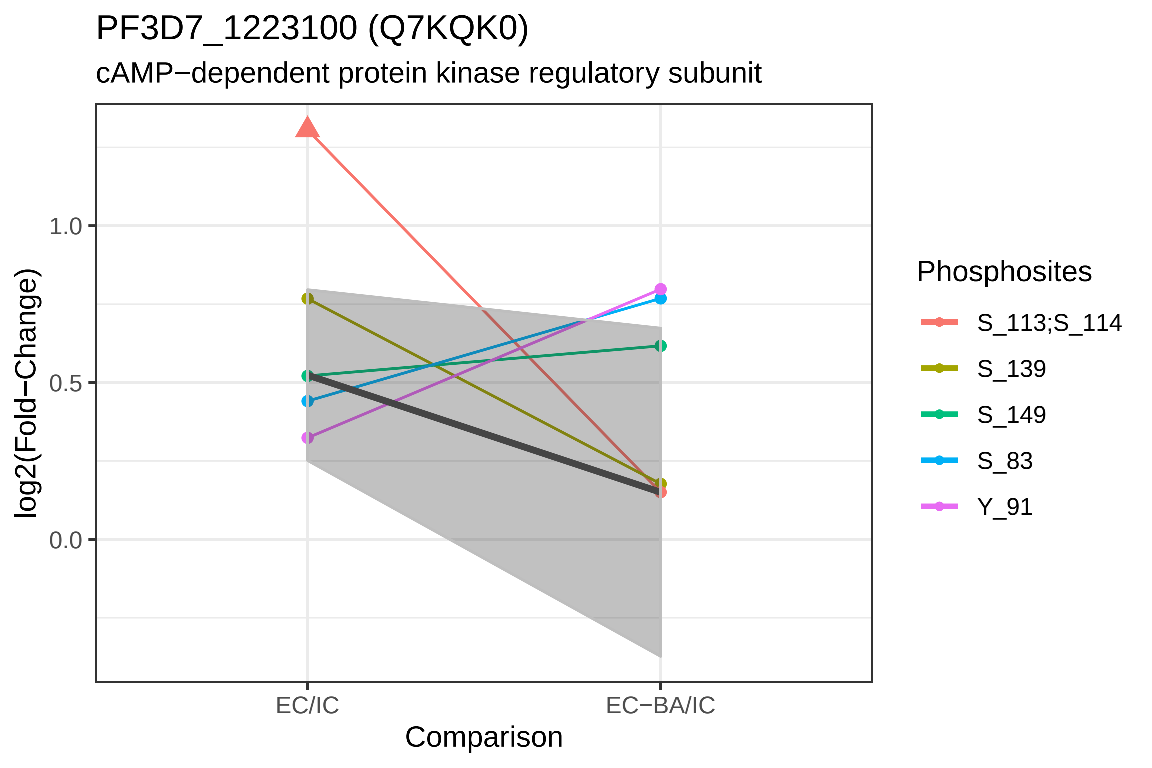

PF3D7_1223100 (Q7KQK0)
cAMP−dependent protein kinase regulatory subunit
log2(Fold−Change)
1.0
0.5
0.0
EC/IC
EC−BA/IC
Comparison
Phosphosites
S_113;S_114
S_139
S_149
S_83
Y_91

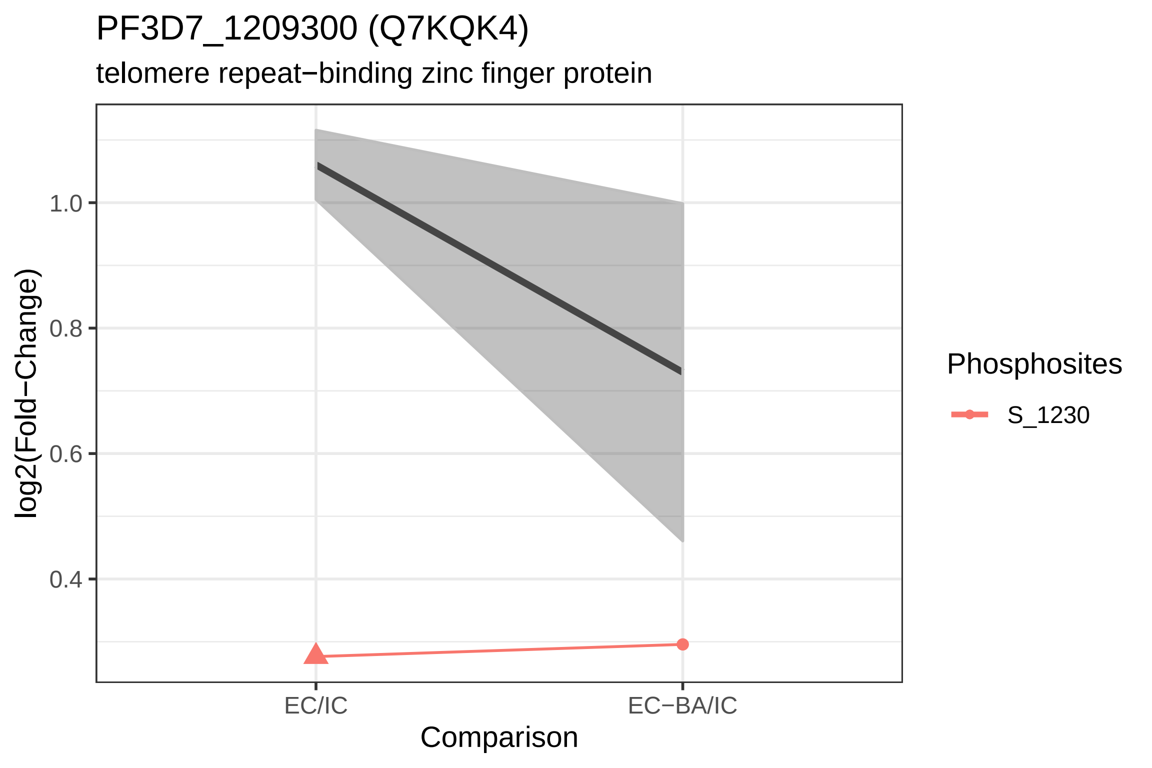

PF3D7_1209300 (Q7KQK4)
telomere repeat−binding zinc finger protein
log2(Fold−Change)
1.0
0.8
0.6
0.4
EC/IC
EC−BA/IC
Comparison
Phosphosites
S_1230

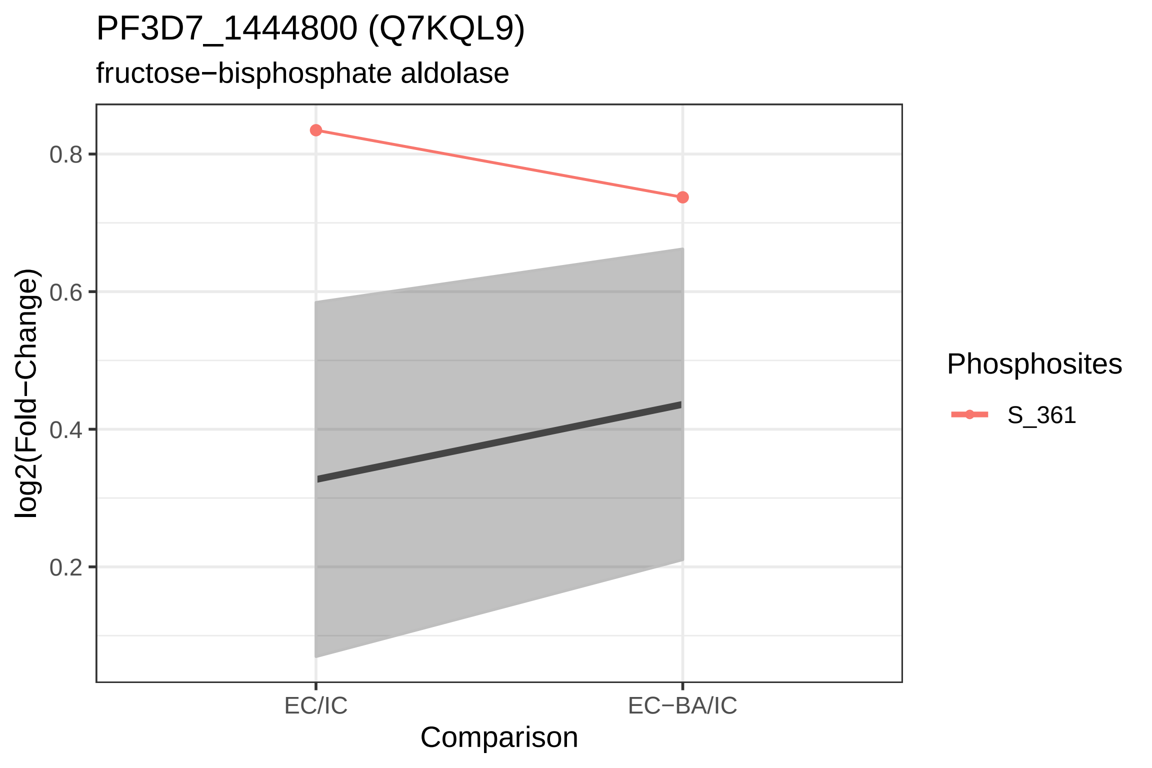

PF3D7_1444800 (Q7KQL9)
fructose−bisphosphate aldolase
log2(Fold−Change)
0.8
0.6
0.4
0.2
EC/IC
EC−BA/IC
Comparison
Phosphosites
S_361

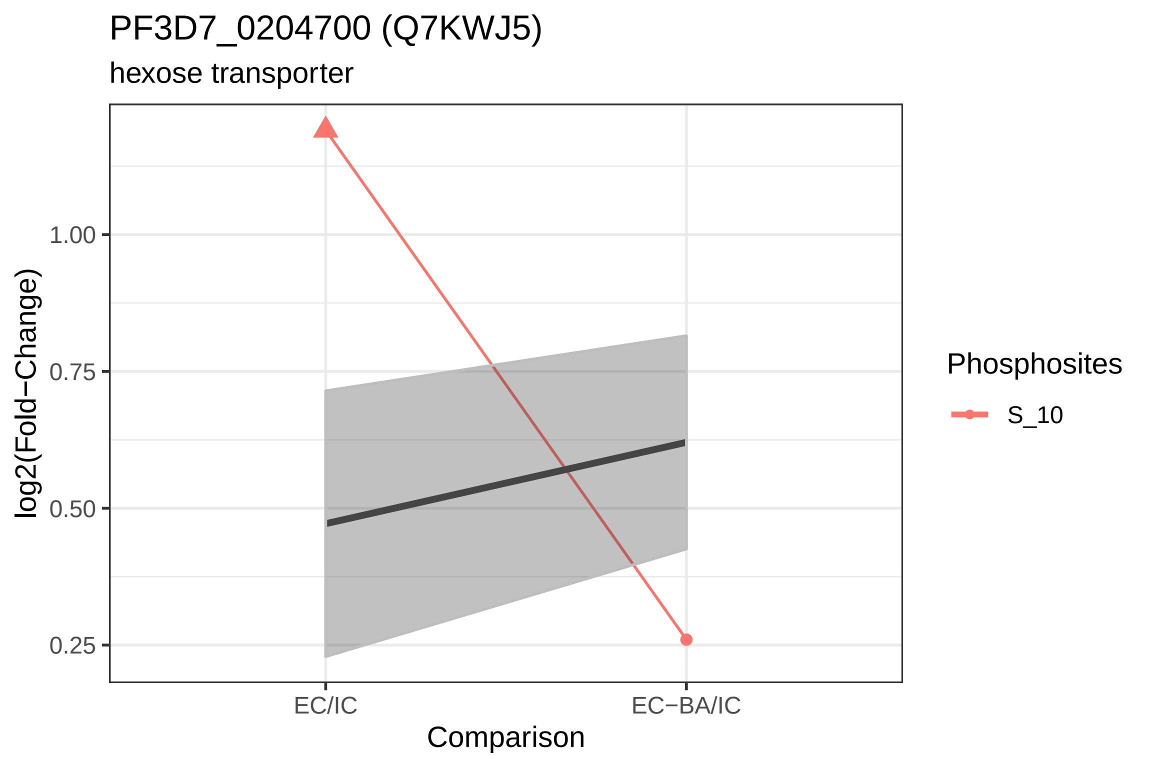
PF3D7_0204700 (Q7KWJ5)
hexose transporter
log2(Fold−Change)
1.00
0.75
0.50
0.25
EC/IC
EC−BA/IC
Comparison
Phosphosites
S_10

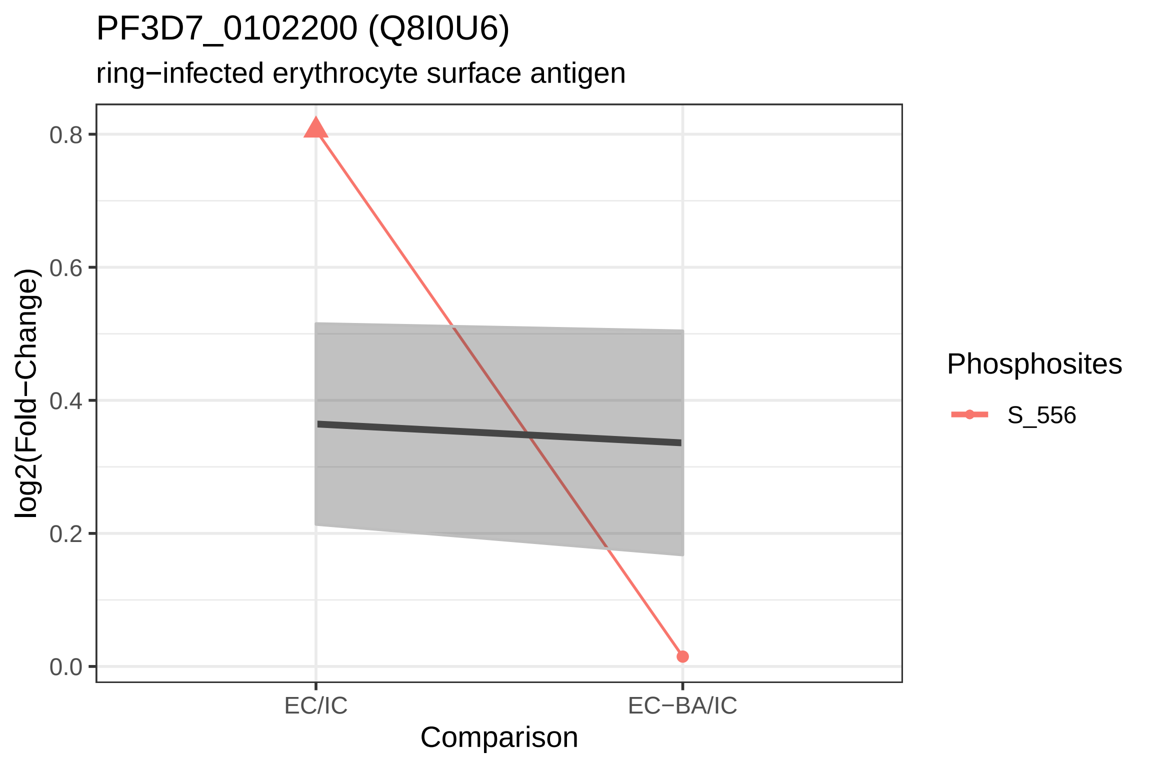
PF3D7_0102200 (Q8I0U6)
ring−infected erythrocyte surface antigen
log2(Fold−Change)
0.8
0.6
0.4
0.2
0.0
EC/IC
EC−BA/IC
Comparison
Phosphosites
S_556

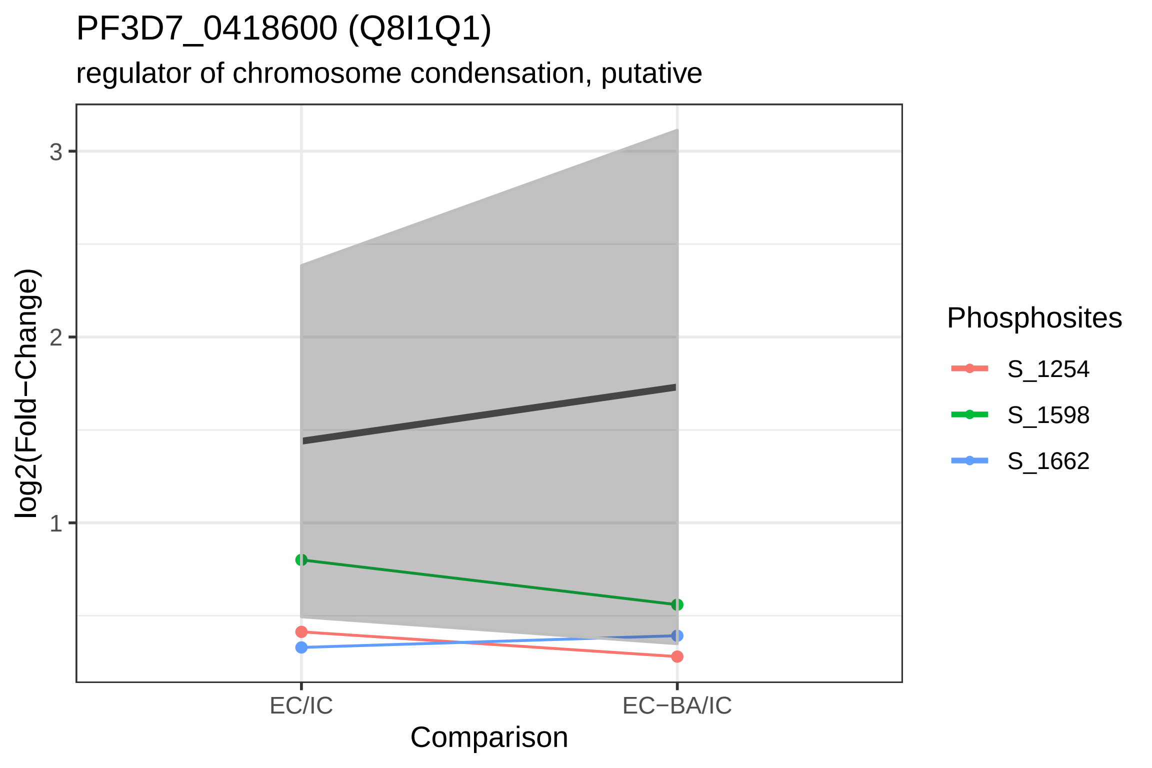
PF3D7_0418600 (Q8I1Q1)
regulator of chromosome condensation, putative
log2(Fold−Change)
3
2
1
EC/IC
EC−BA/IC
Comparison
Phosphosites
S_1254
S_1598
S_1662

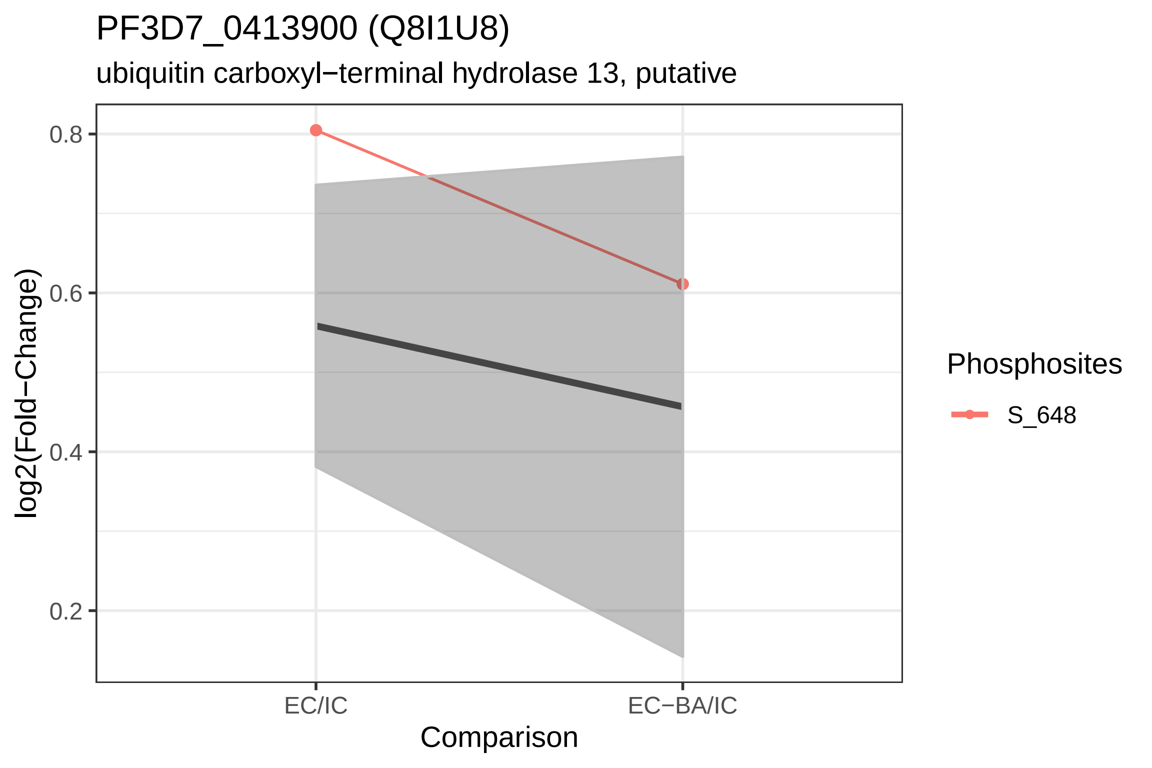
PF3D7_0413900 (Q8I1U8)
ubiquitin carboxyl−terminal hydrolase 13, putative
log2(Fold−Change)
0.8
0.6
0.4
0.2
EC/IC
EC−BA/IC
Comparison
Phosphosites
S_648

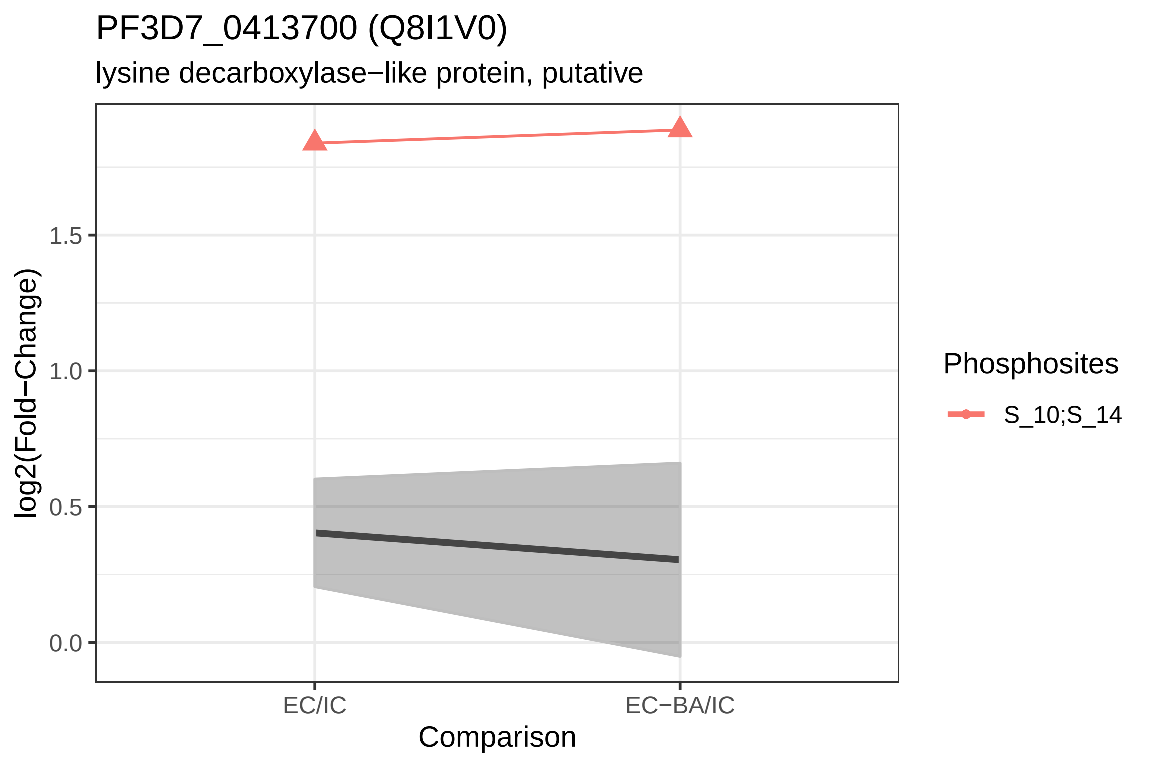

PF3D7_0413700 (Q8I1V0)
lysine decarboxylase−like protein, putative
log2(Fold−Change)
0.0
0.5
1.0
1.5
EC/IC
EC−BA/IC
Comparison
Phosphosites
S_10;S_14

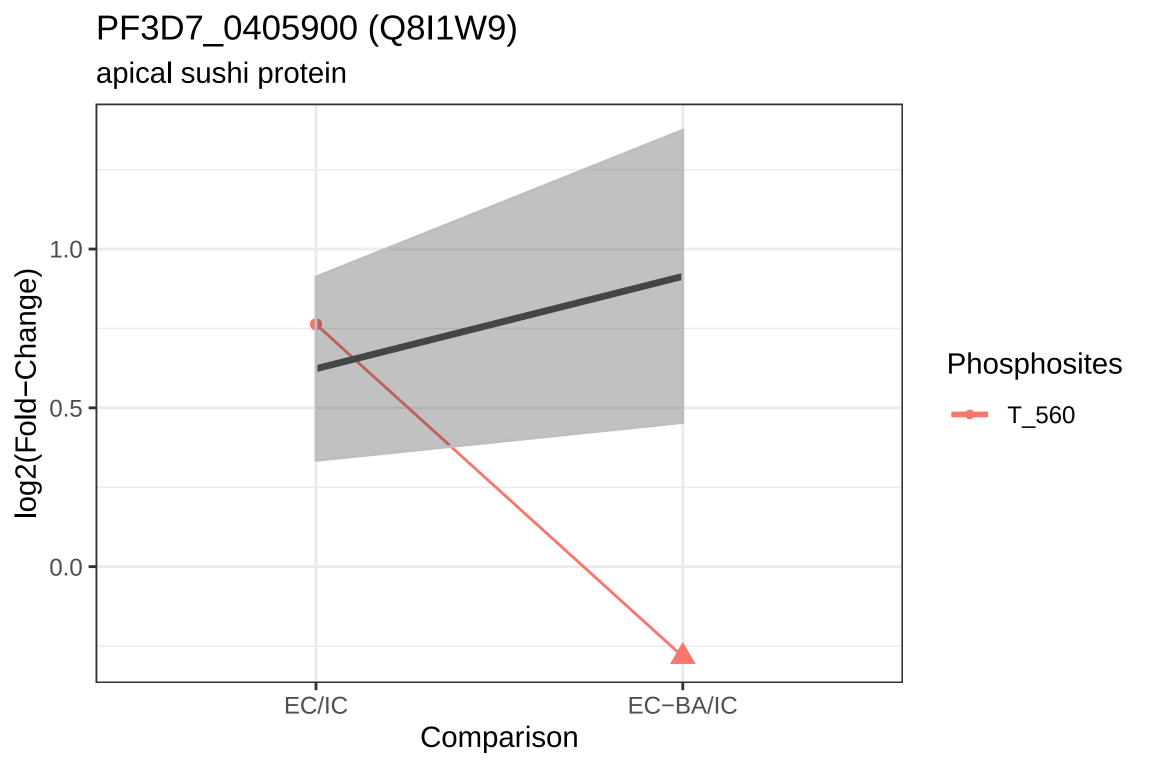
PF3D7_0405900 (Q8I1W9)
apical sushi protein
1.0
0.5
0.0
log2(Fold−Change)
EC/IC
EC−BA/IC
Comparison
Phosphosites
T_560

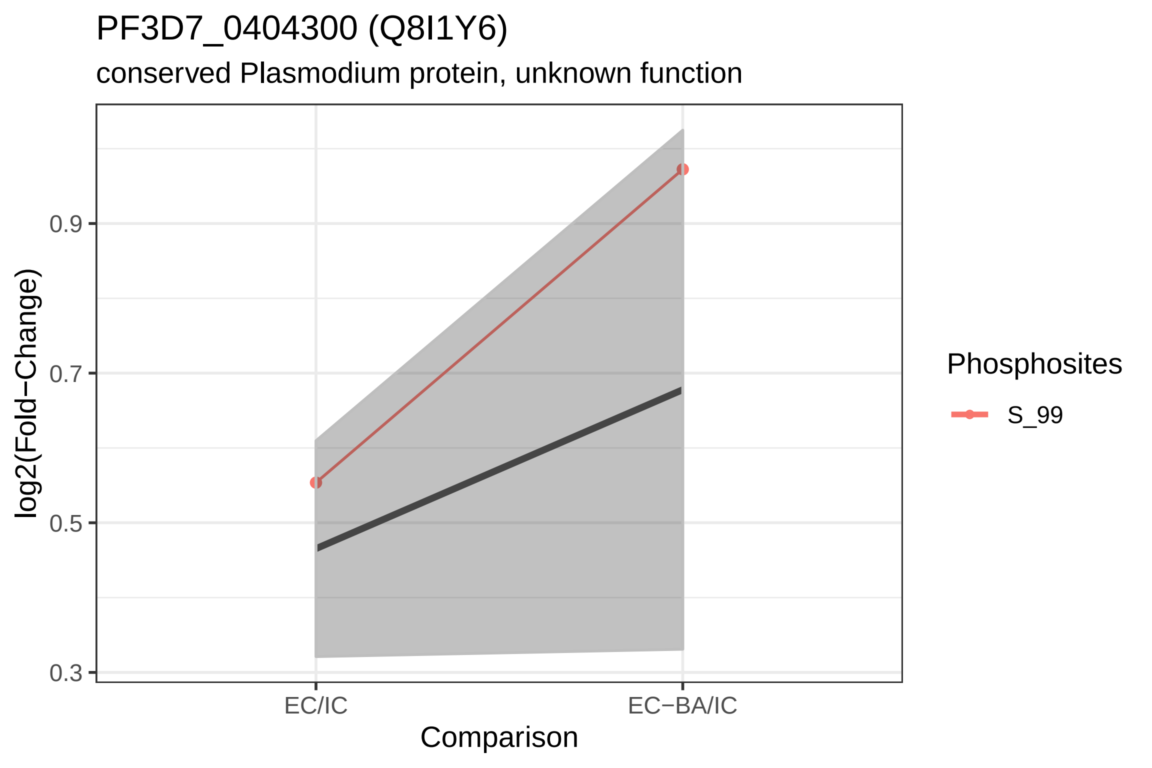
PF3D7_0404300 (Q8I1Y6)
conserved Plasmodium protein, unknown function
log2(Fold−Change)
0.9
0.7
0.5
0.3
EC/IC
EC−BA/IC
Comparison
Phosphosites
S_99

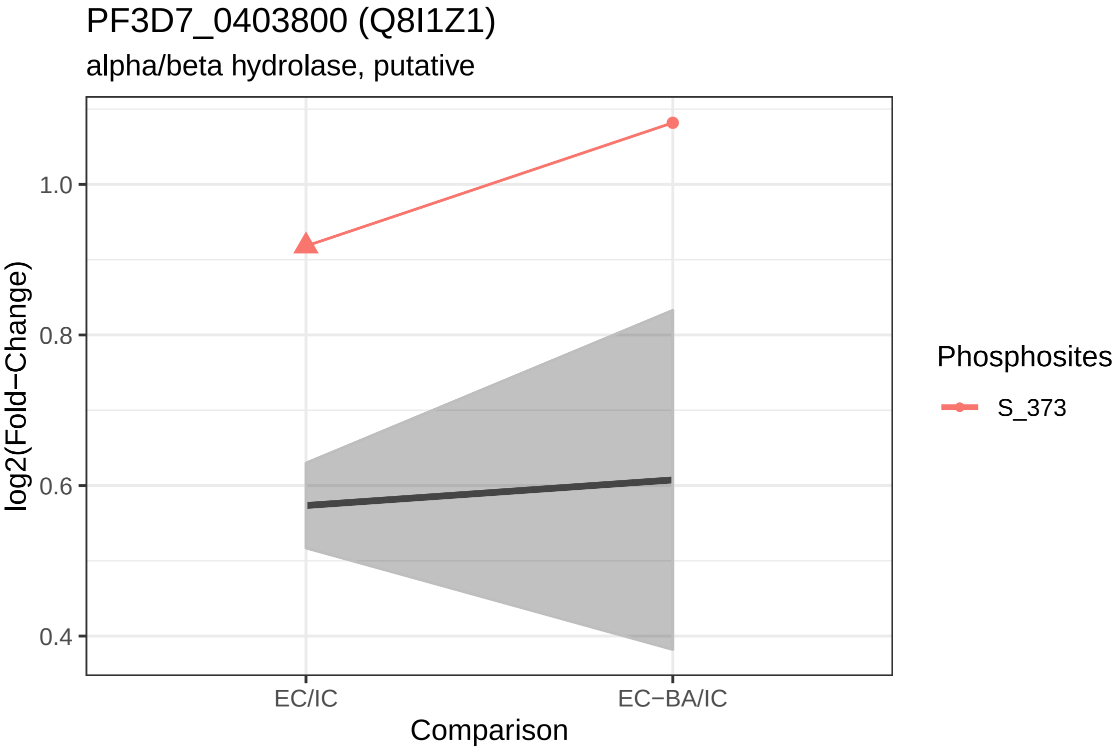
PF3D7_0403800 (Q8I1Z1)
alpha/beta hydrolase, putative
log2(Fold−Change)
1.0
0.8
0.6
0.4
EC/IC
EC−BA/IC
Comparison
Phosphosites
S_373

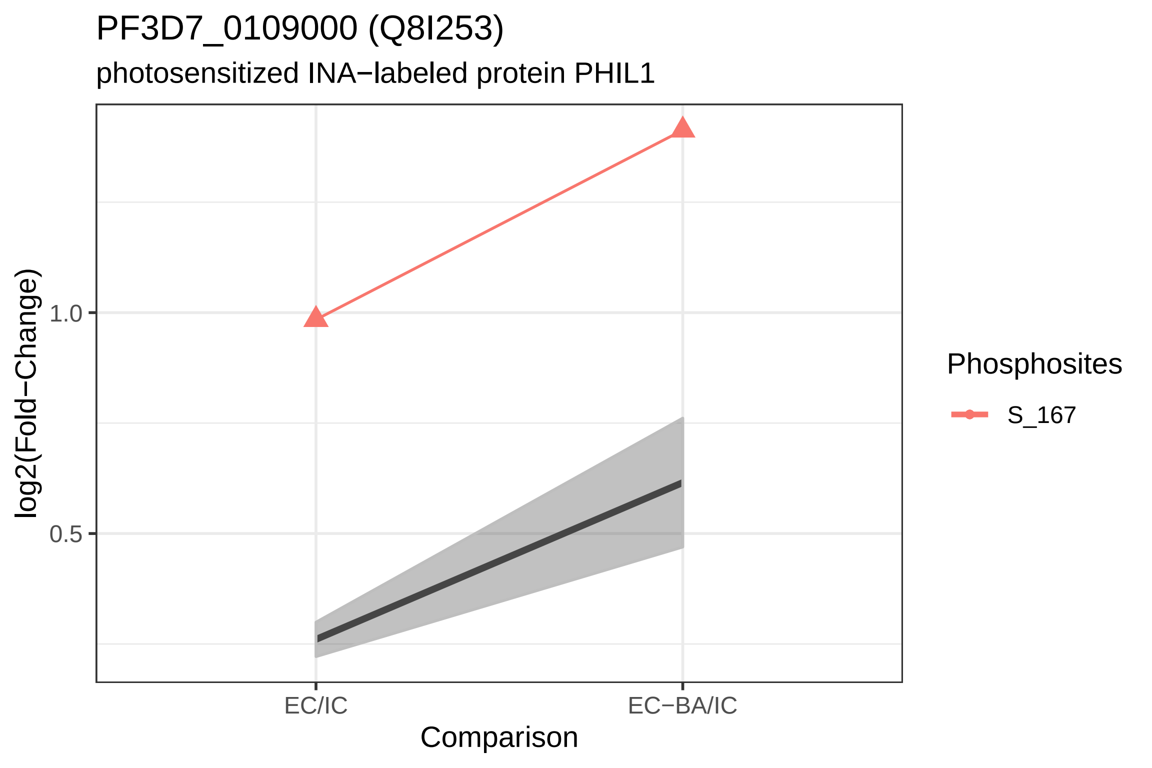

PF3D7_0109000 (Q8I253)
photosensitized INA−labeled protein PHIL1
log2(Fold−Change)
1.0
0.5
EC/IC
EC−BA/IC
Comparison
Phosphosites
S_167

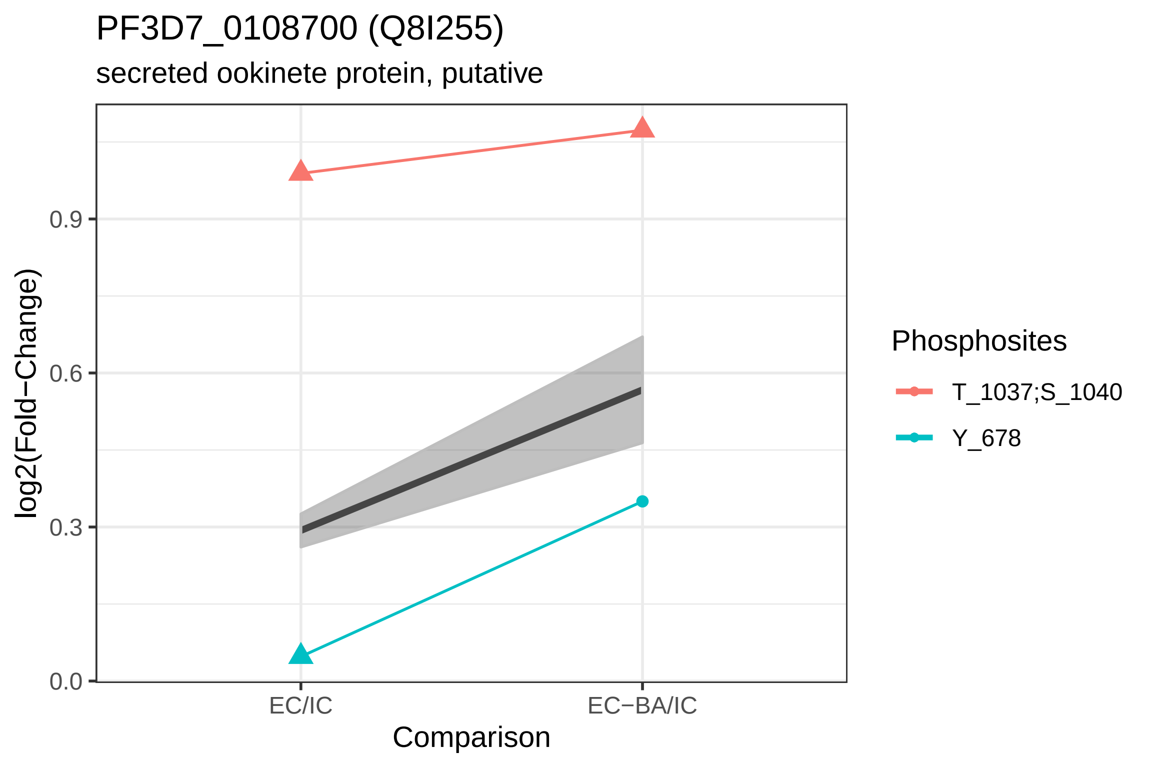
PF3D7_0108700 (Q8I255)
secreted ookinete protein, putative
log2(Fold−Change)
0.0
0.3
0.6
0.9
EC/IC
EC−BA/IC
Comparison
Phosphosites
T_1037;S_1040
Y_678

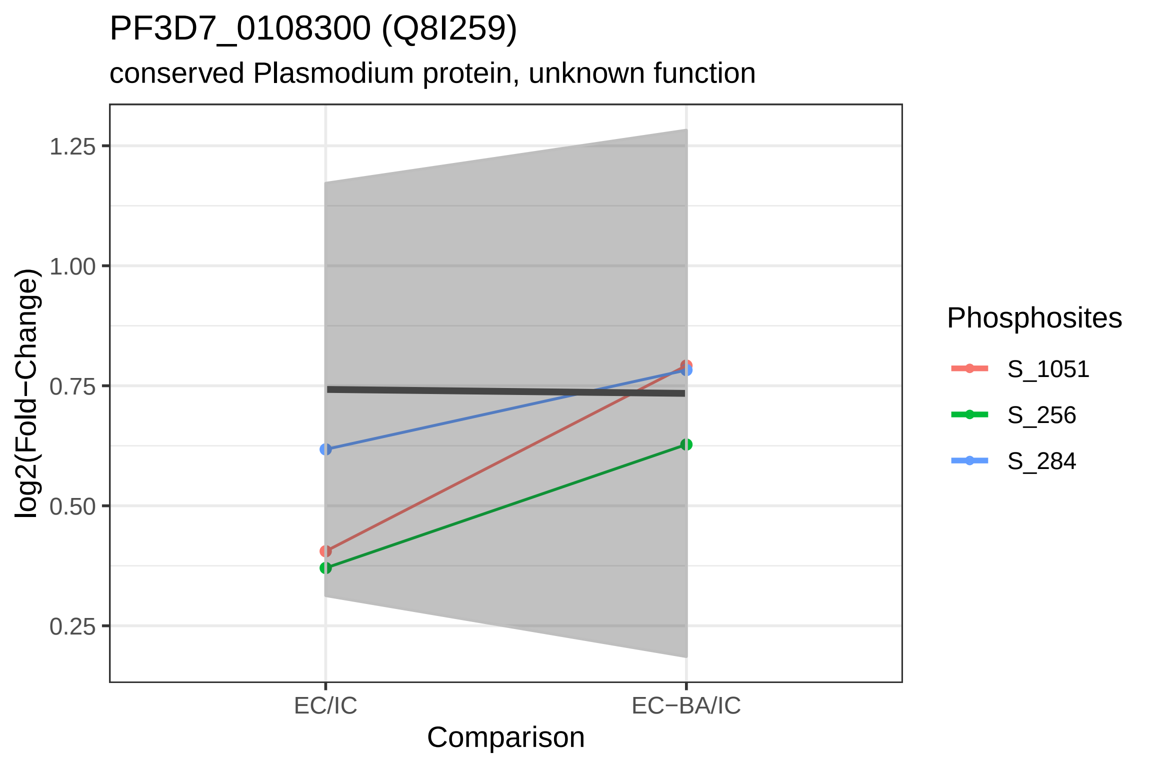
PF3D7_0108300 (Q8I259)
conserved Plasmodium protein, unknown function
log2(Fold−Change)
1.25
1.00
0.75
0.50
0.25
EC/IC
EC−BA/IC
Comparison
Phosphosites
S_1051
S_256
S_284

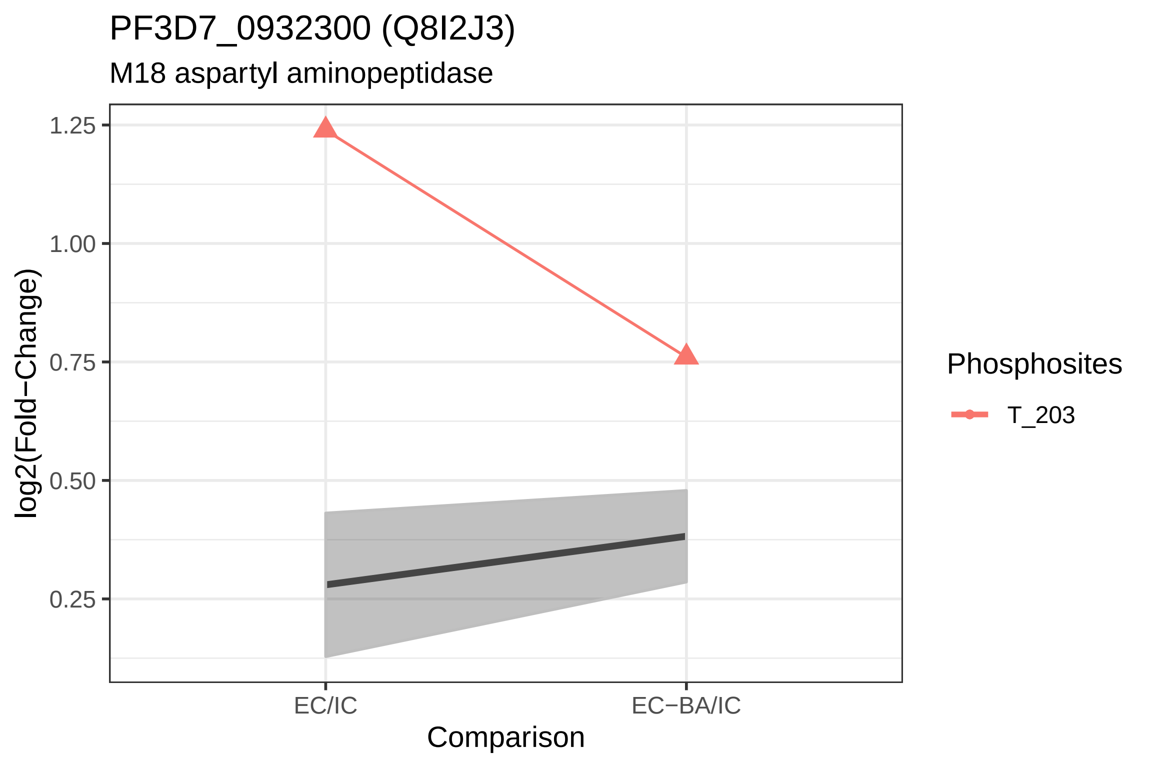

PF3D7_0932300 (Q8I2J3)
M18 aspartyl aminopeptidase
log2(Fold−Change)
1.25
1.00
0.75
0.50
0.25
EC/IC
EC−BA/IC
Comparison
Phosphosites
T_203

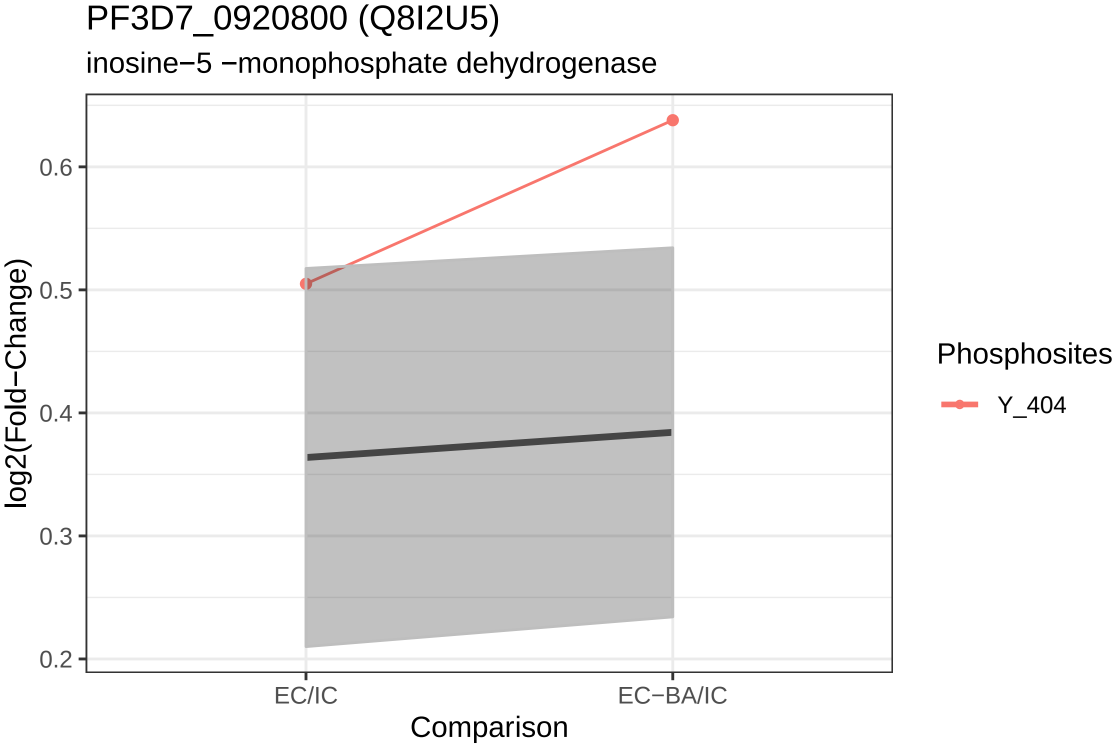

PF3D7_0920800 (Q8I2U5)
inosine−5 −monophosphate dehydrogenase
log2(Fold−Change)
0.6
0.5
0.4
0.3
0.2
EC/IC
EC−BA/IC
Comparison
Phosphosites
Y_404

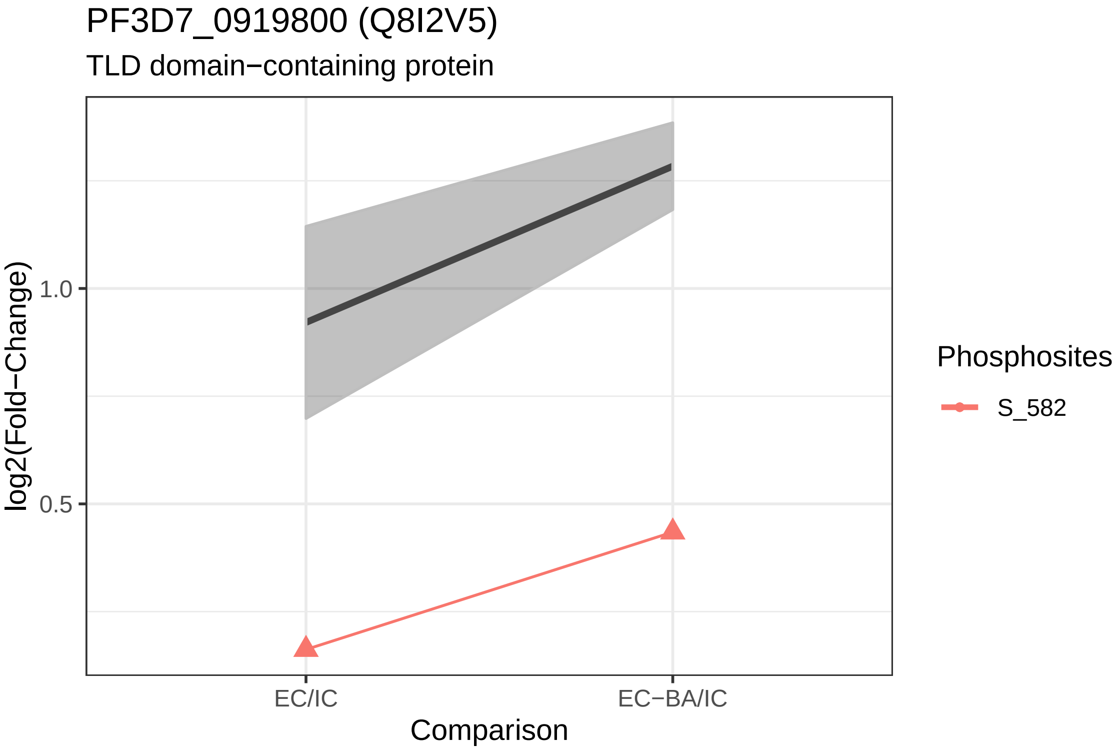

PF3D7_0919800 (Q8I2V5)
TLD domain−containing protein
log2(Fold−Change)
1.0
0.5
EC/IC
EC−BA/IC
Comparison
Phosphosites
S_582

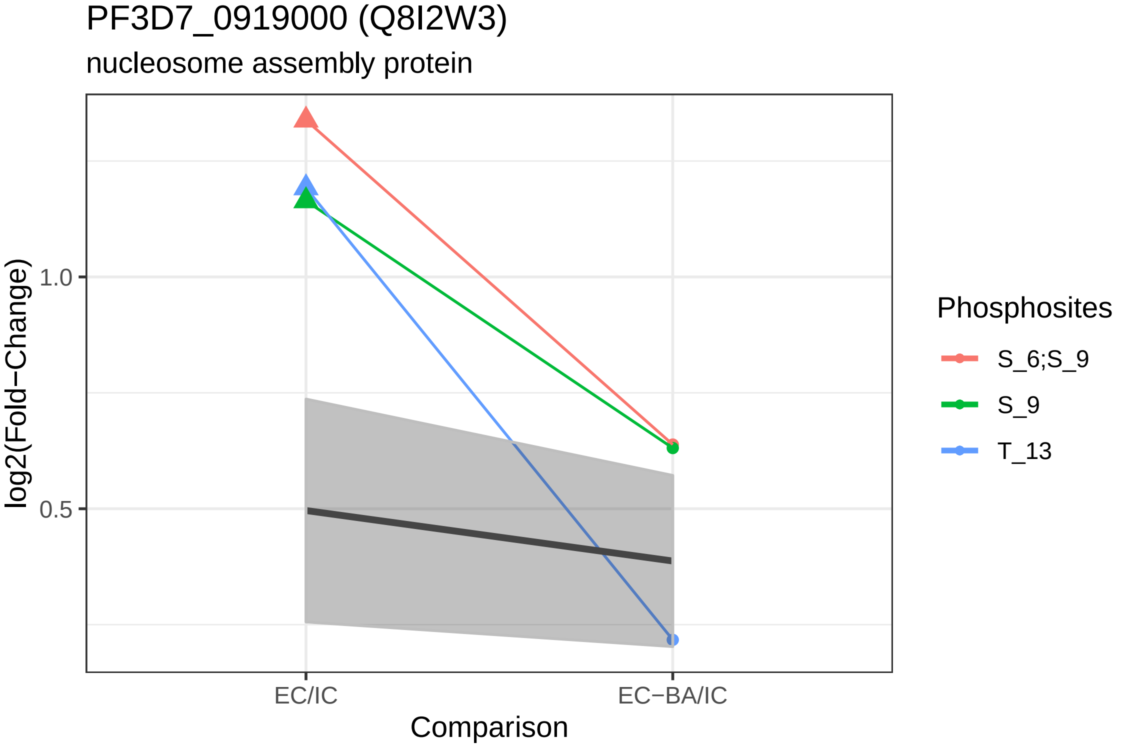
PF3D7_0919000 (Q8I2W3)
nucleosome assembly protein
log2(Fold−Change)
1.0
0.5
EC/IC
EC−BA/IC
Comparison
Phosphosites
S_6;S_9
S_9
T_13

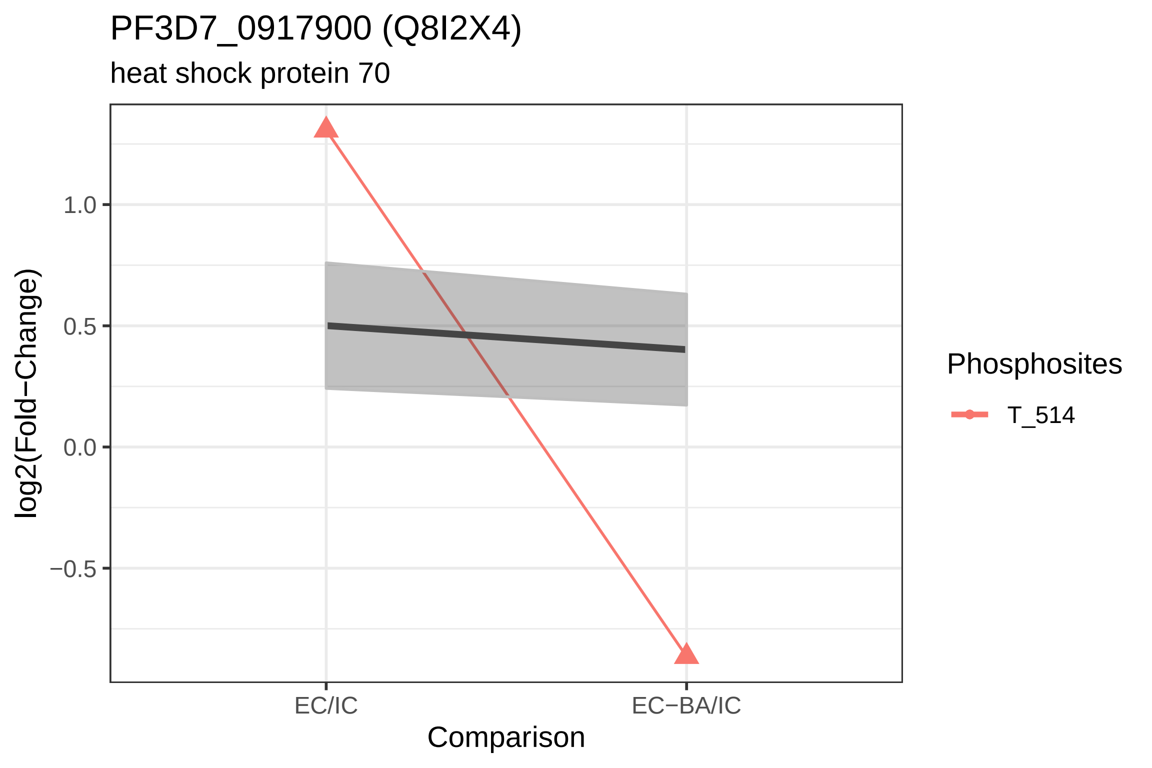

PF3D7_0917900 (Q8I2X4)
heat shock protein 70
log2(Fold−Change)
1.0
0.5
0.0
−0.5
EC/IC
EC−BA/IC
Comparison
Phosphosites
T_514

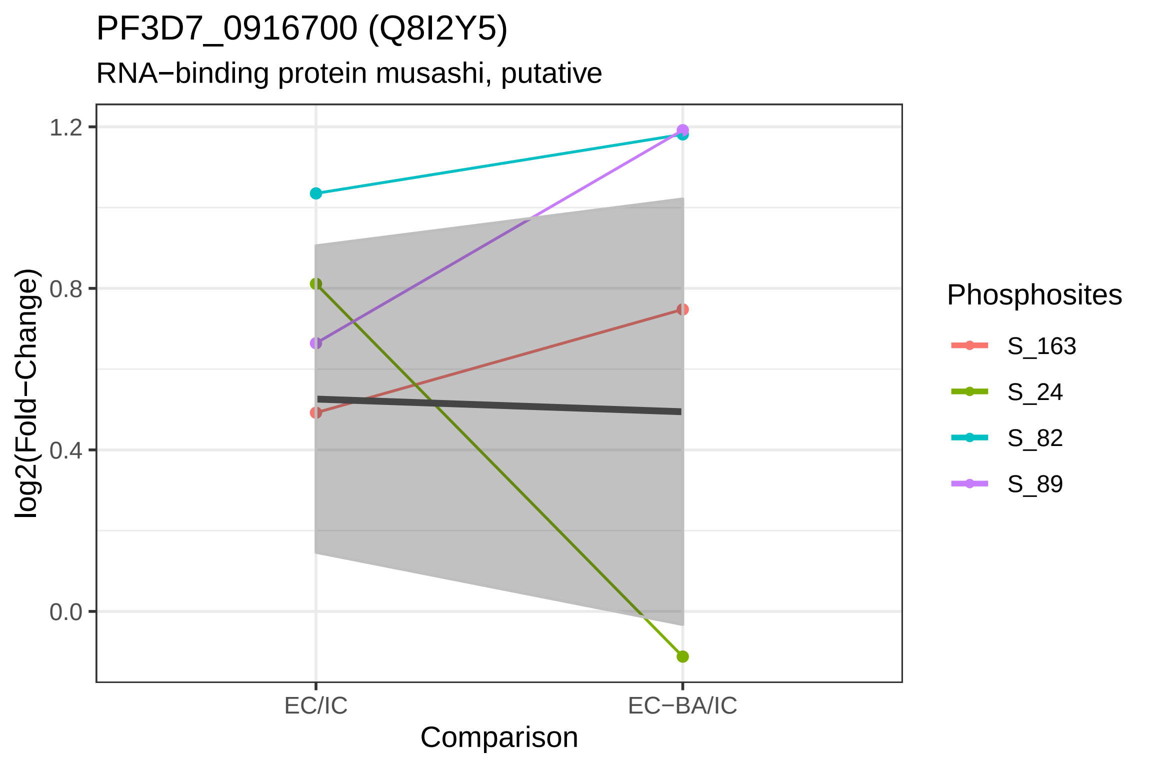
PF3D7_0916700 (Q8I2Y5)
RNA−binding protein musashi, putative
log2(Fold−Change)
1.2
0.8
0.4
0.0
EC/IC
EC−BA/IC
Comparison
Phosphosites
S_163
S_24
S_82
S_89

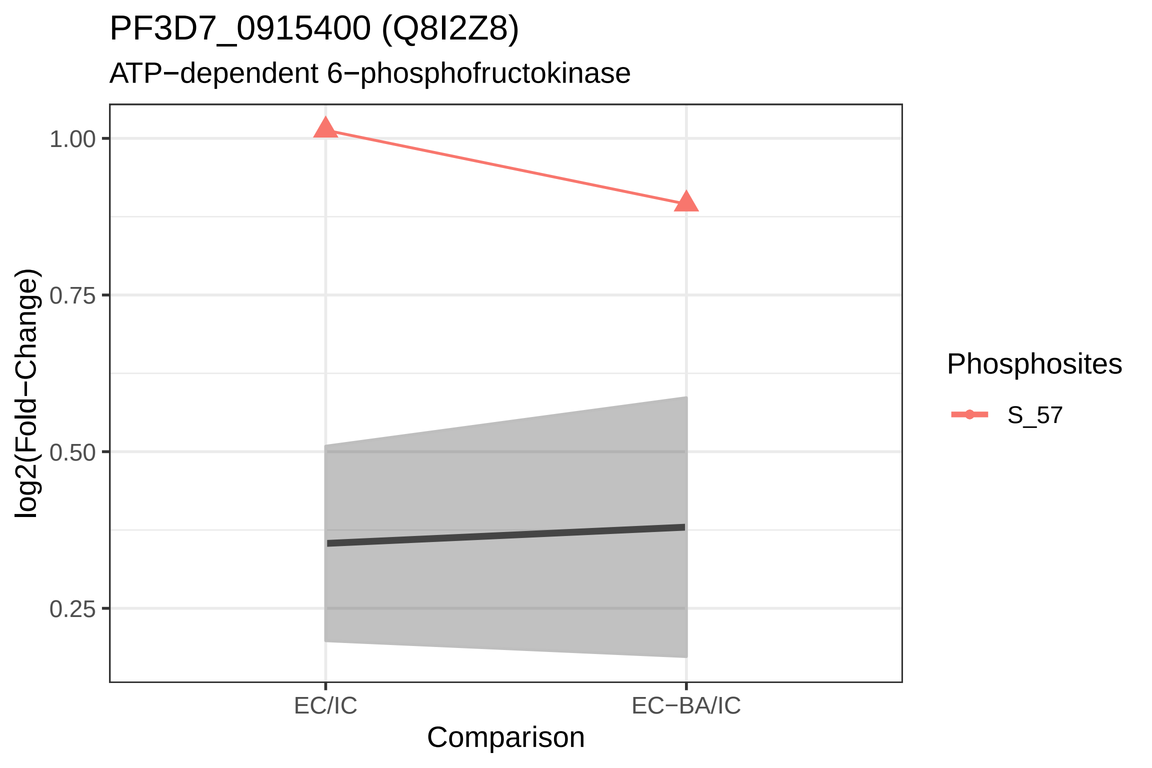
PF3D7_0915400 (Q8I2Z8)
ATP−dependent 6−phosphofructokinase
log2(Fold−Change)
1.00
0.75
0.50
0.25
EC/IC
EC−BA/IC
Comparison
Phosphosites
S_57

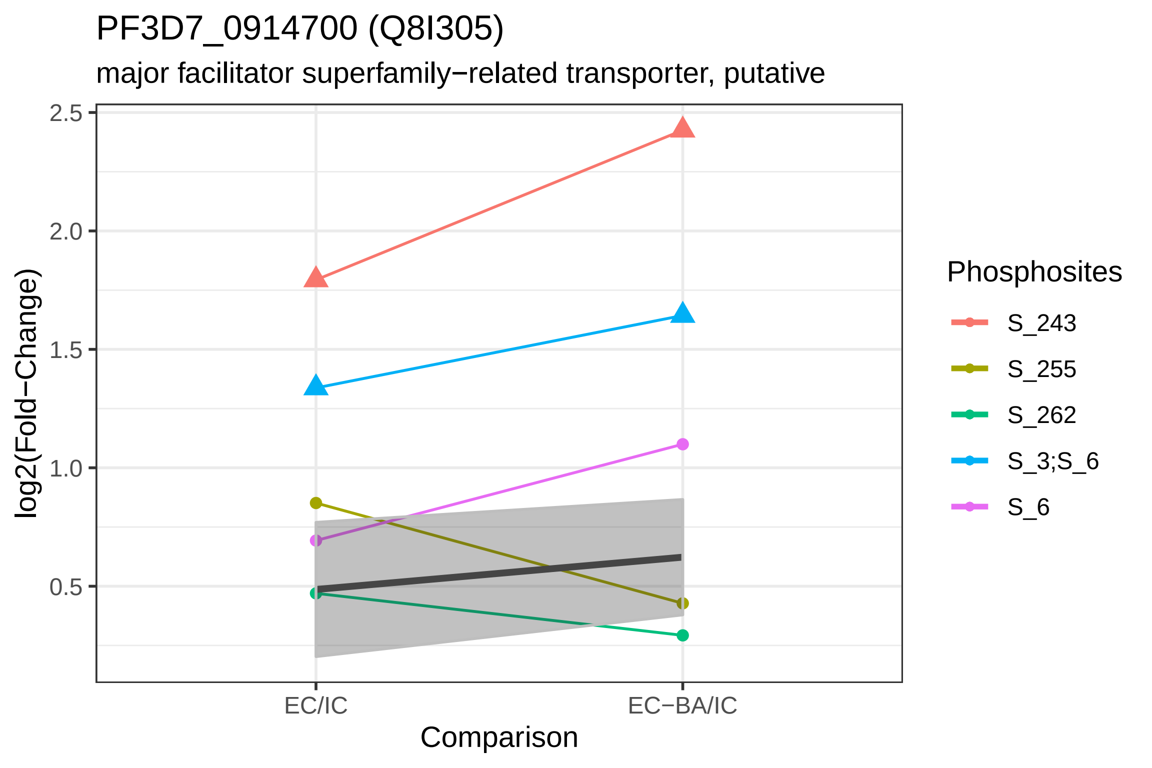

PF3D7_0914700 (Q8I305)
major facilitator superfamily−related transporter, putative
log2(Fold−Change)
2.5
2.0
1.5
1.0
0.5
EC/IC
EC−BA/IC
Comparison
Phosphosites
S_243
S_255
S_262
S_3;S_6
S_6

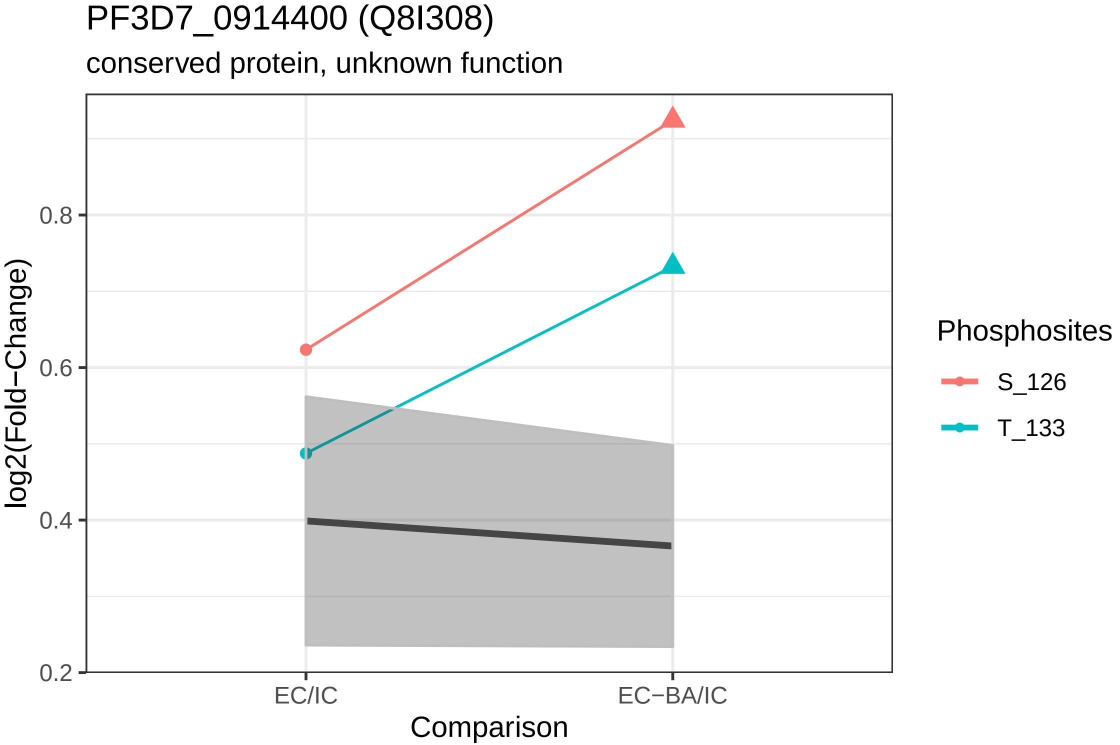
PF3D7_0914400 (Q8I308)
conserved protein, unknown function
log2(Fold−Change)
0.8
0.6
0.4
0.2
EC/IC
EC−BA/IC
Comparison
Phosphosites
S_126
T_133

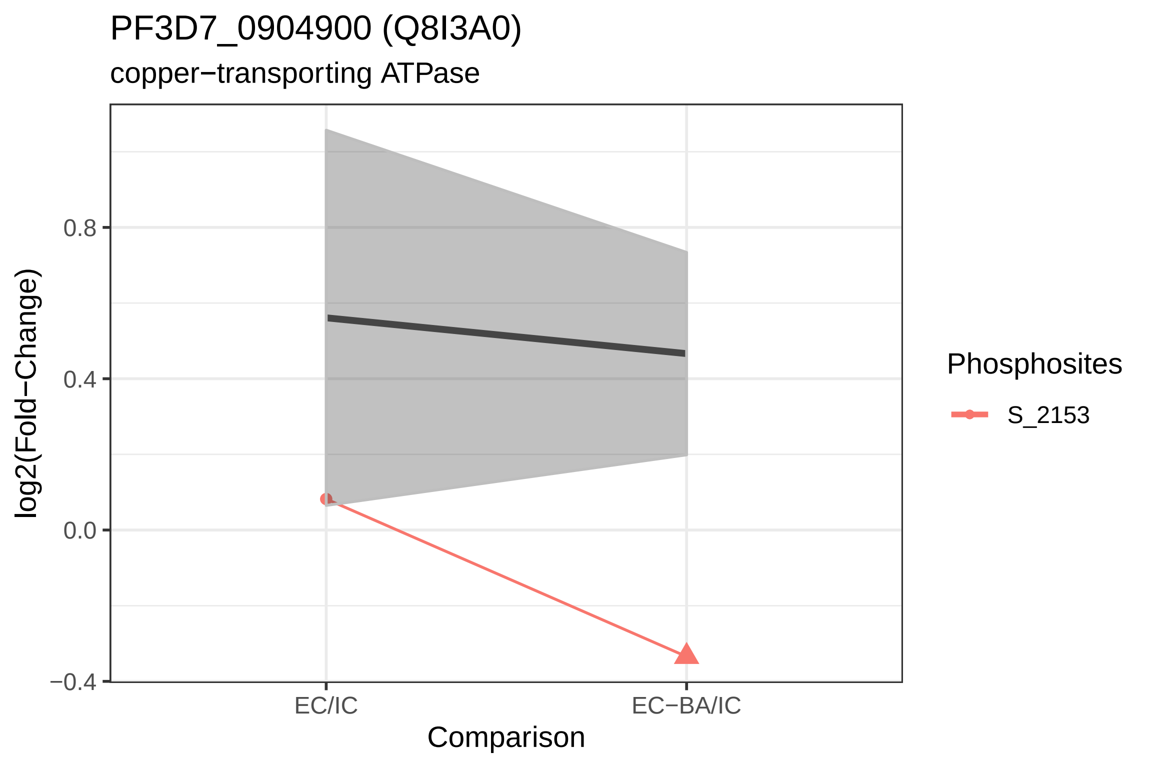
PF3D7_0904900 (Q8I3A0)
copper−transporting ATPase
log2(Fold−Change)
0.8
0.4
0.0
−0.4
EC/IC
EC−BA/IC
Comparison
Phosphosites
S_2153

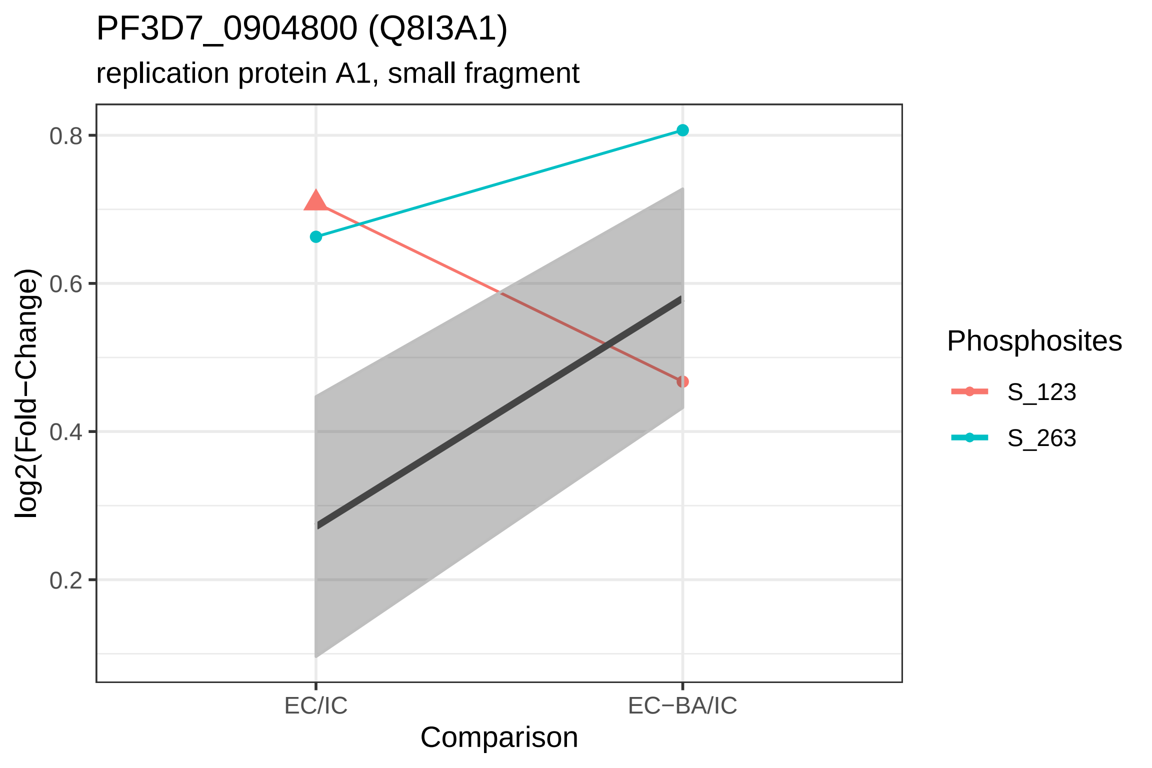

PF3D7_0904800 (Q8I3A1)
replication protein A1, small fragment
log2(Fold−Change)
0.8
0.6
0.4
0.2
EC/IC
EC−BA/IC
Comparison
Phosphosites
S_123
S_263

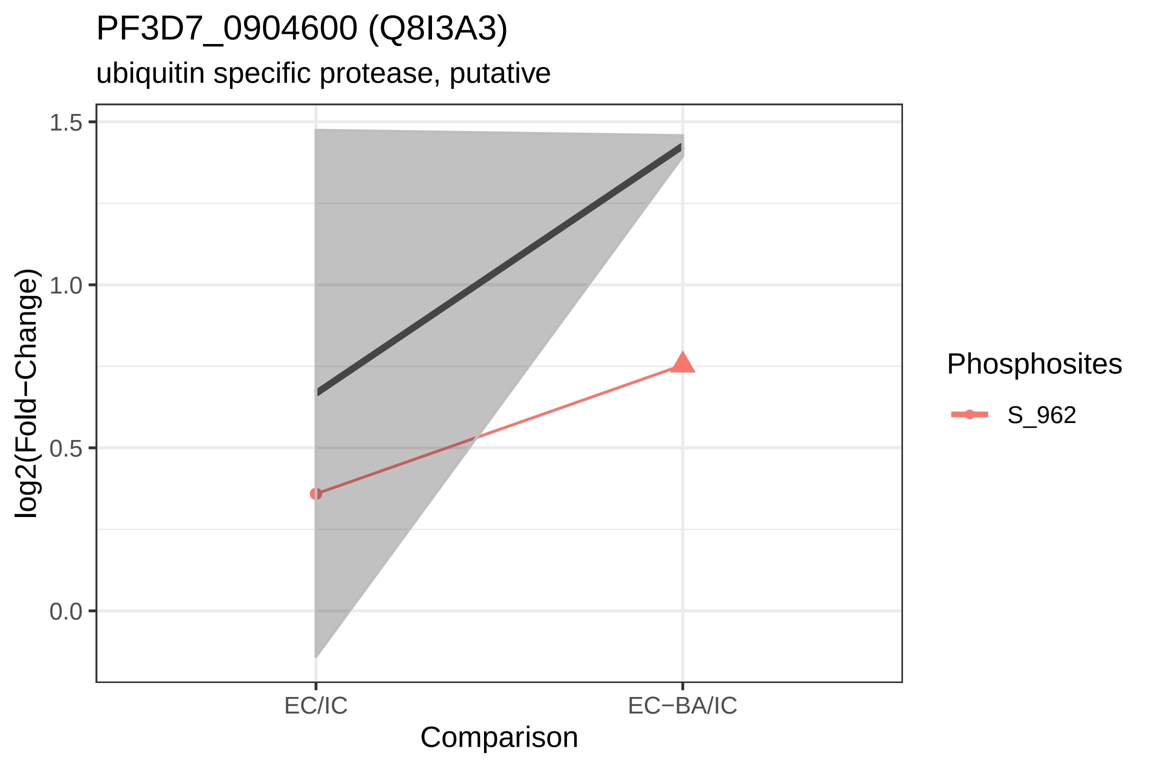

PF3D7_0904600 (Q8I3A3)
ubiquitin specific protease, putative
log2(Fold−Change)
1.5
1.0
0.5
0.0
EC/IC
EC−BA/IC
Comparison
Phosphosites
S_962

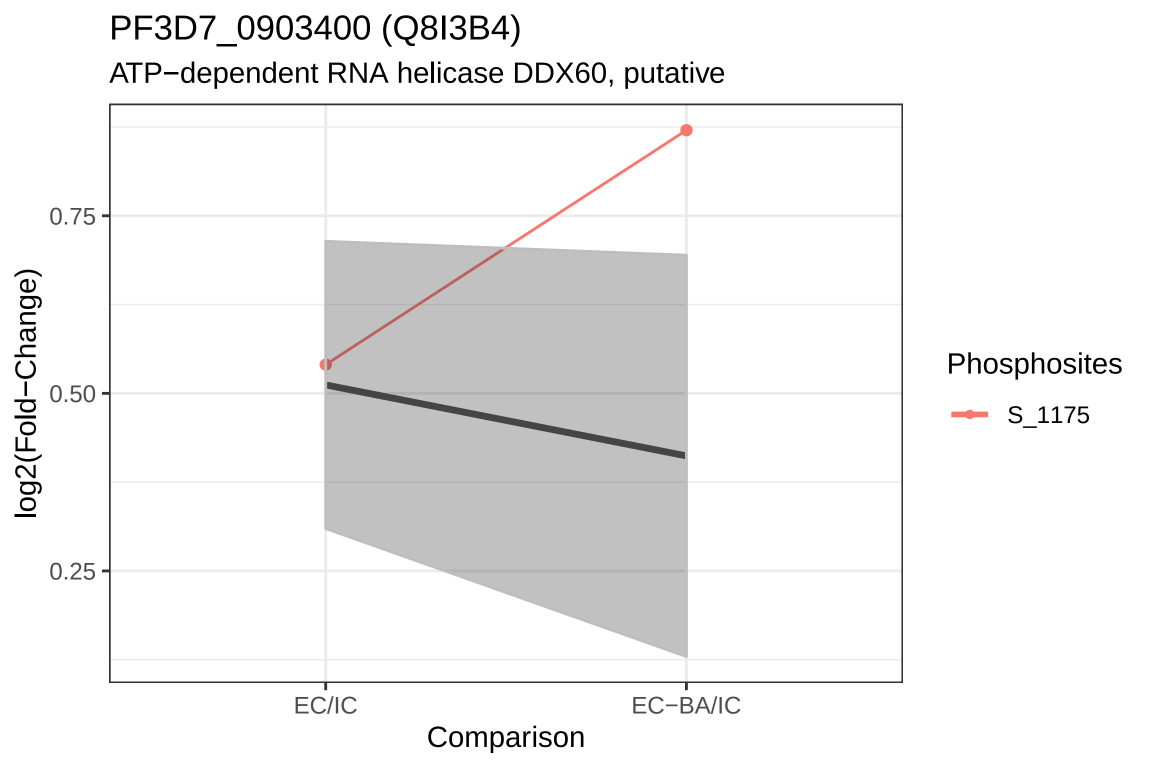
PF3D7_0903400 (Q8I3B4)
ATP−dependent RNA helicase DDX60, putative
log2(Fold−Change)
0.75
0.50
0.25
EC/IC
EC−BA/IC
Comparison
Phosphosites
S_1175

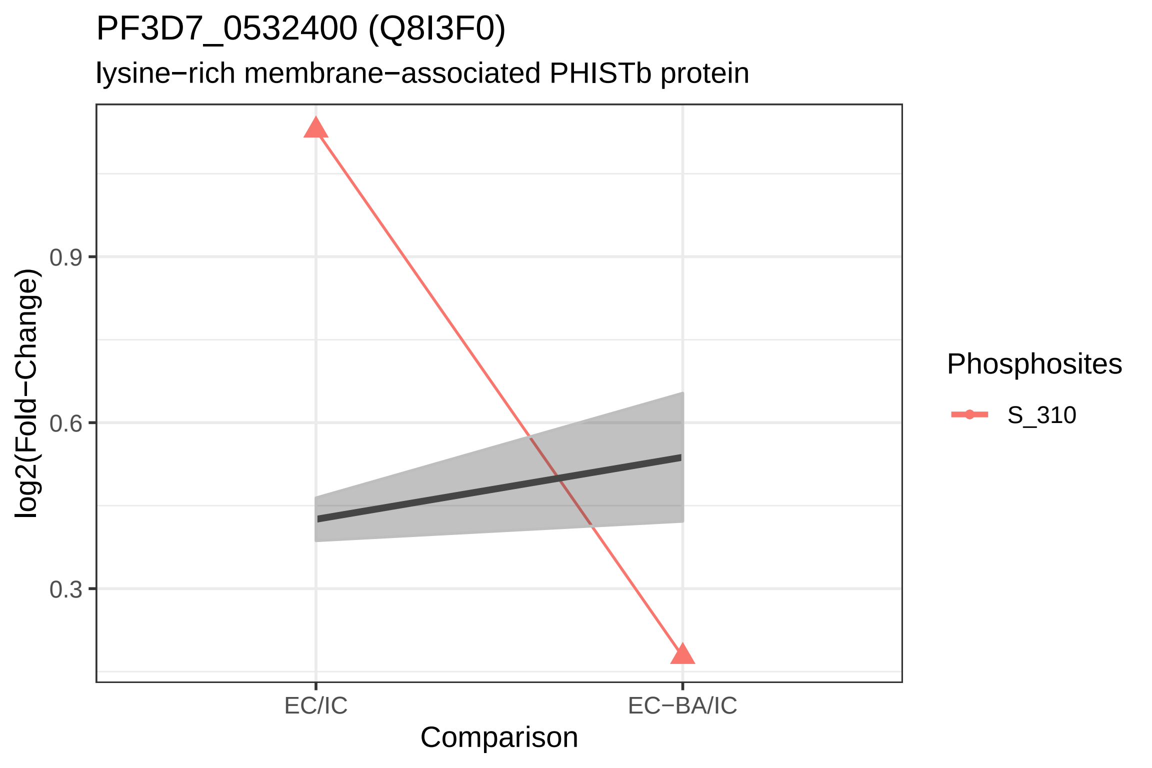
PF3D7_0532400 (Q8I3F0)
lysine−rich membrane−associated PHISTb protein
log2(Fold−Change)
0.9
0.6
0.3
EC/IC
EC−BA/IC
Comparison
Phosphosites
S_310

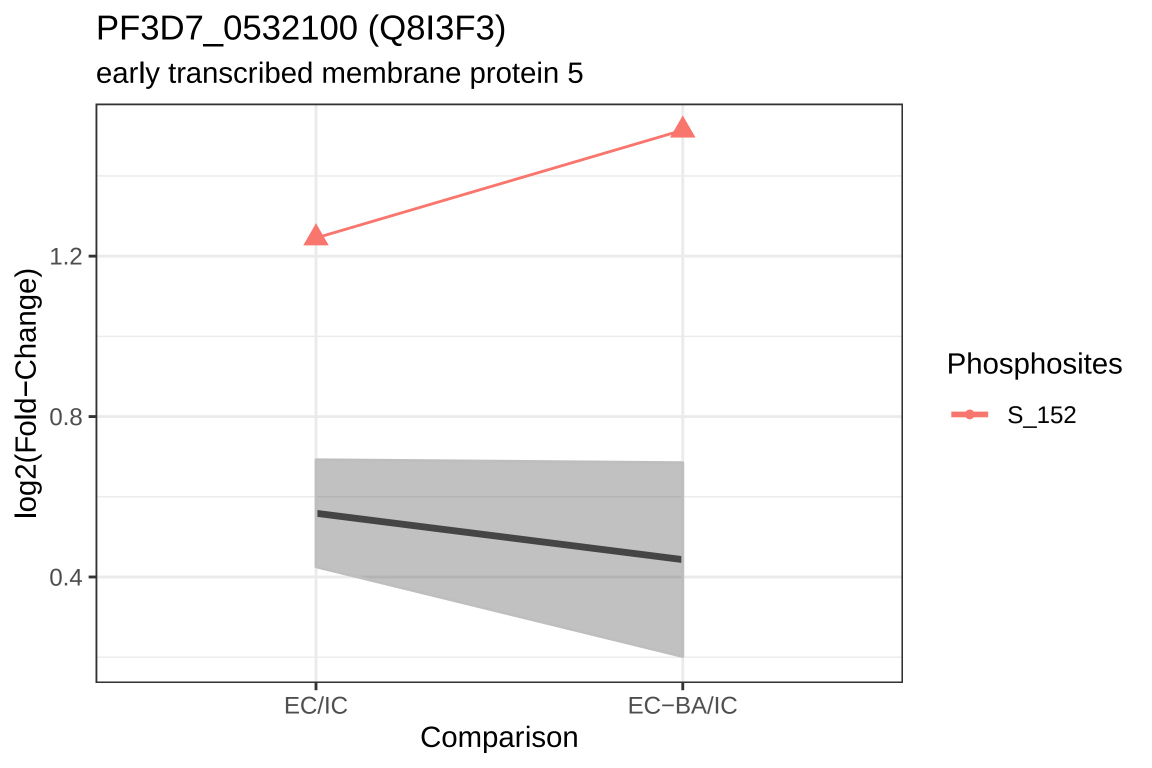

PF3D7_0532100 (Q8I3F3)
early transcribed membrane protein 5
log2(Fold−Change)
1.2
0.8
0.4
EC/IC
EC−BA/IC
Comparison
Phosphosites
S_152

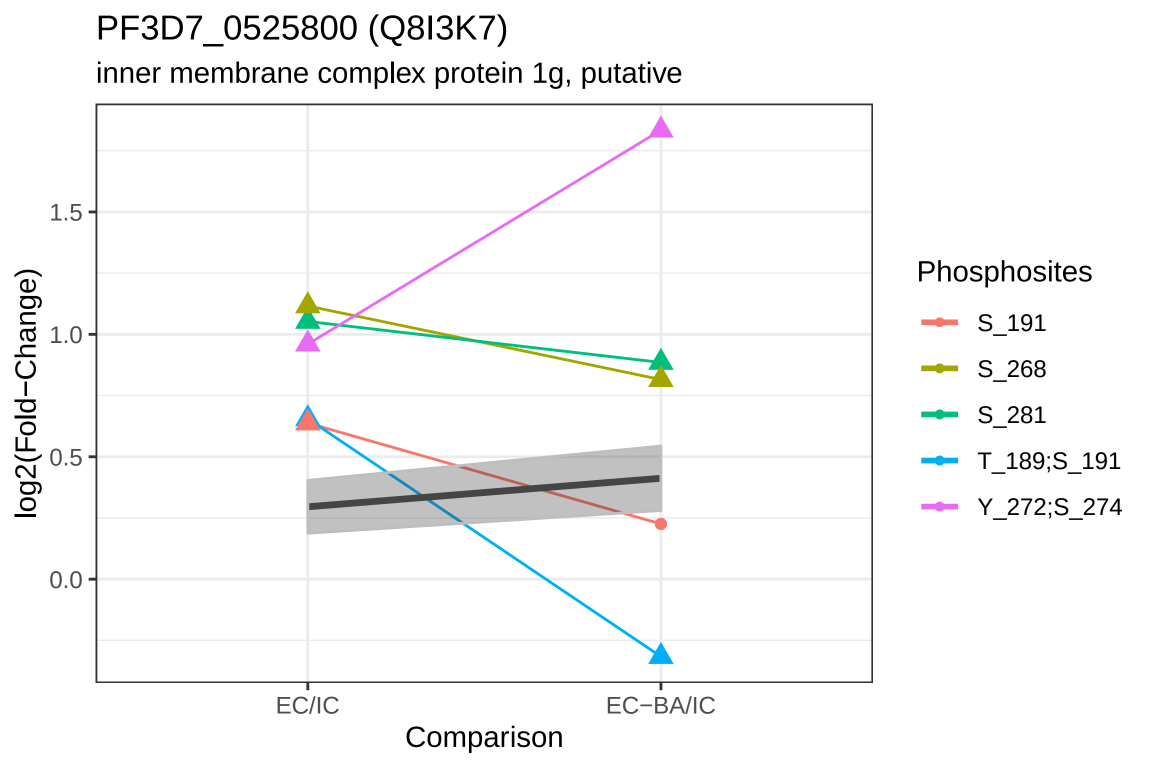
PF3D7_0525800 (Q8I3K7)
inner membrane complex protein 1g, putative
log2(Fold−Change)
1.5
1.0
0.5
0.0
EC/IC
EC−BA/IC
Comparison
Phosphosites
S_191
S_268
S_281
T_189;S_191
Y_272;S_274

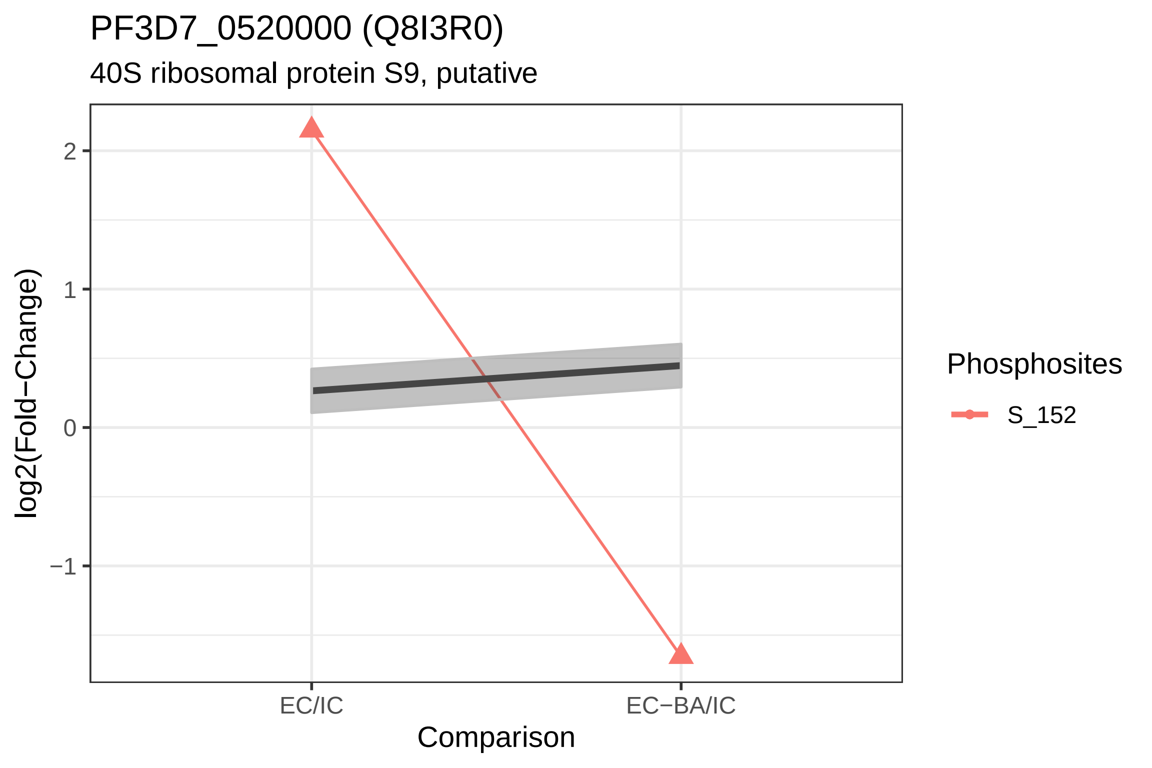

PF3D7_0520000 (Q8I3R0)
40S ribosomal protein S9, putative
log2(Fold−Change)
2
1
0
−1
EC/IC
EC−BA/IC
Comparison
Phosphosites
S_152

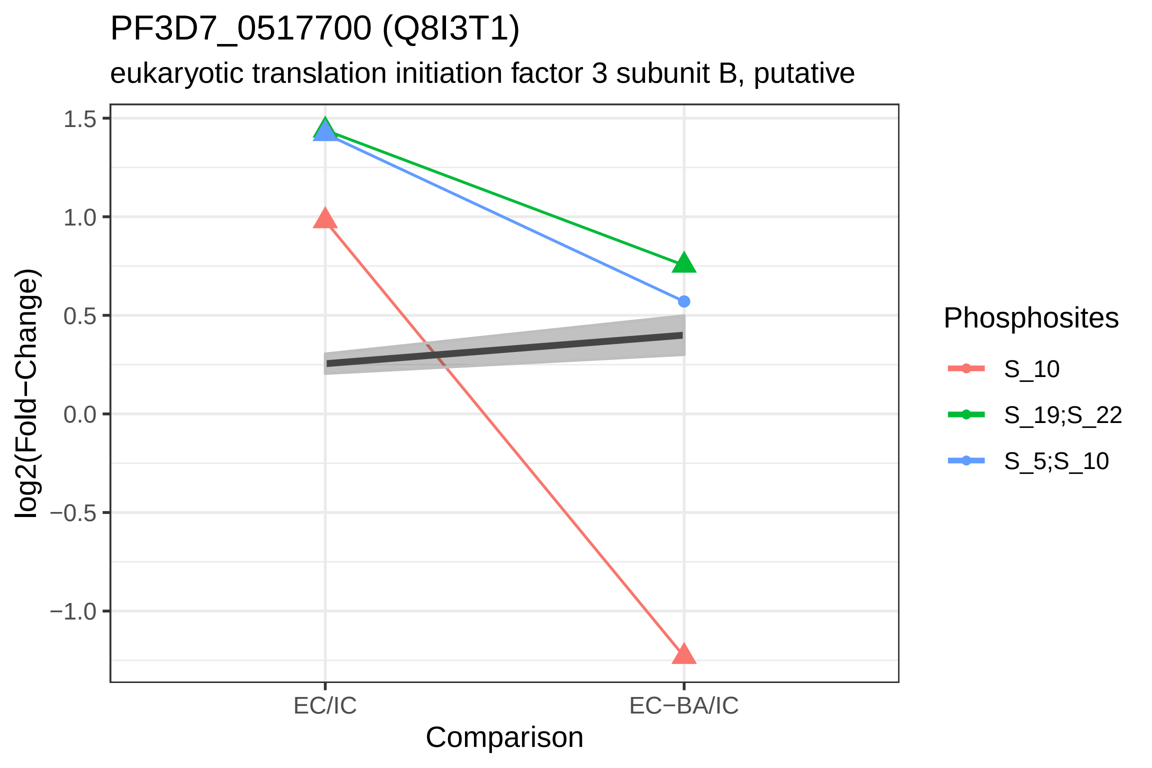

PF3D7_0517700 (Q8I3T1)
eukaryotic translation initiation factor 3 subunit B, putative
log2(Fold−Change)
1.5
1.0
0.5
0.0
−0.5
−1.0
EC/IC
EC−BA/IC
Comparison
Phosphosites
S_10
S_19;S_22
S_5;S_10

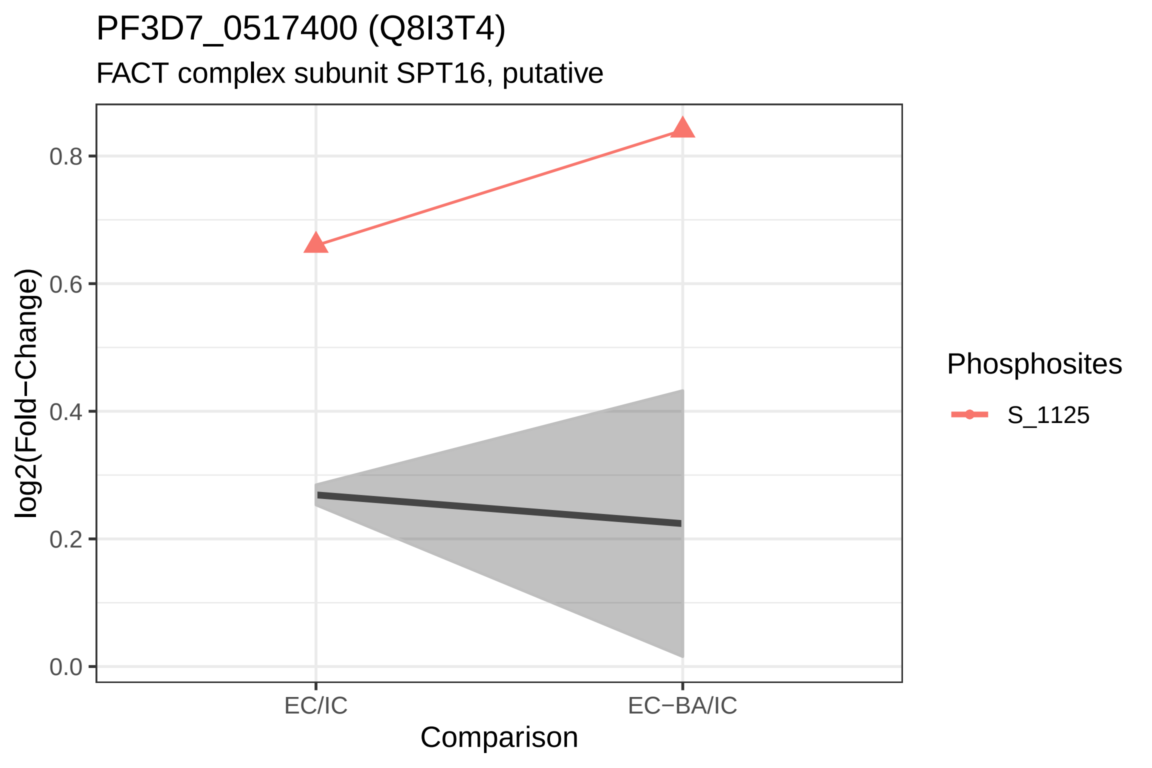
PF3D7_0517400 (Q8I3T4)
FACT complex subunit SPT16, putative
log2(Fold−Change)
0.0
0.2
0.4
0.6
0.8
EC/IC
EC−BA/IC
Comparison
Phosphosites
S_1125

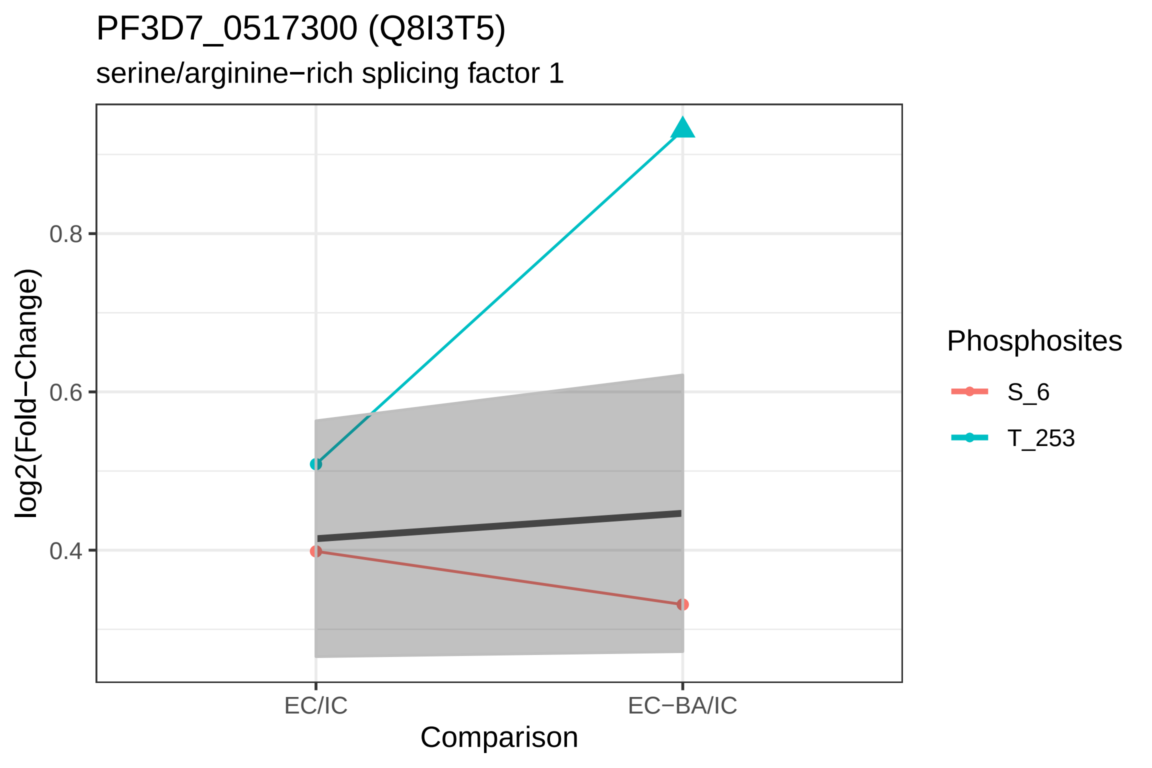
PF3D7_0517300 (Q8I3T5)
serine/arginine−rich splicing factor 1
log2(Fold−Change)
0.8
0.6
0.4
EC/IC
EC−BA/IC
Comparison
Phosphosites
S_6
T_253

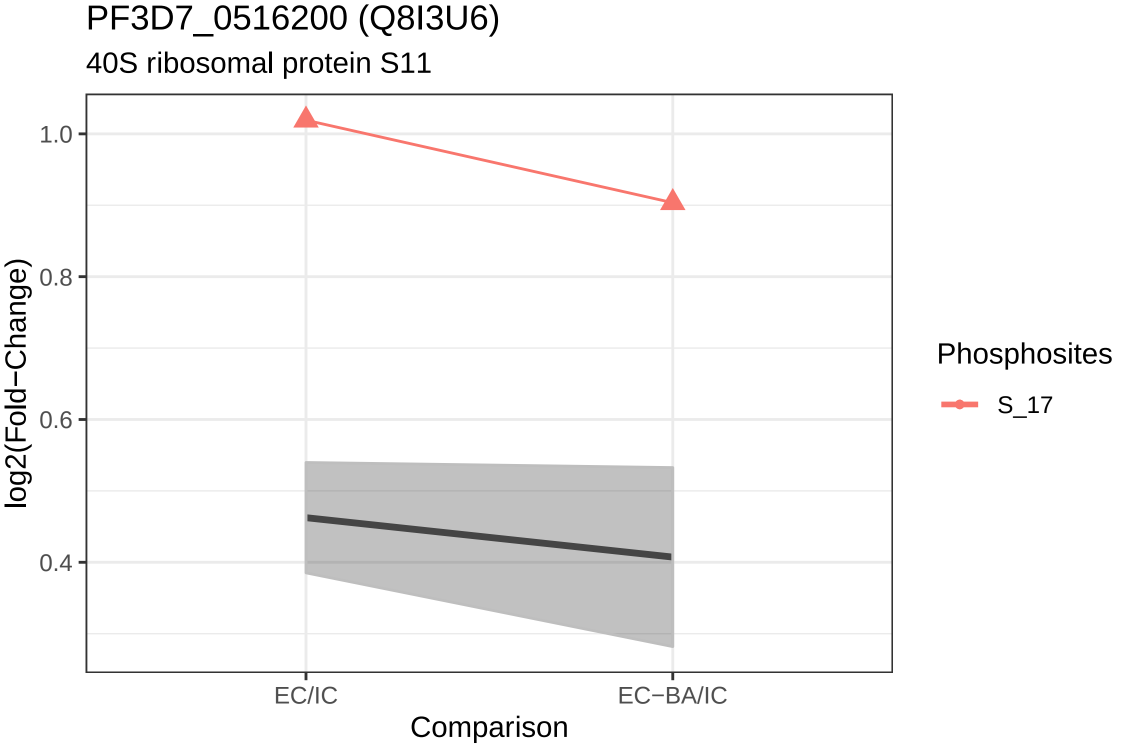

PF3D7_0516200 (Q8I3U6)
40S ribosomal protein S11
log2(Fold−Change)
1.0
0.8
0.6
0.4
EC/IC
EC−BA/IC
Comparison
Phosphosites
S_17

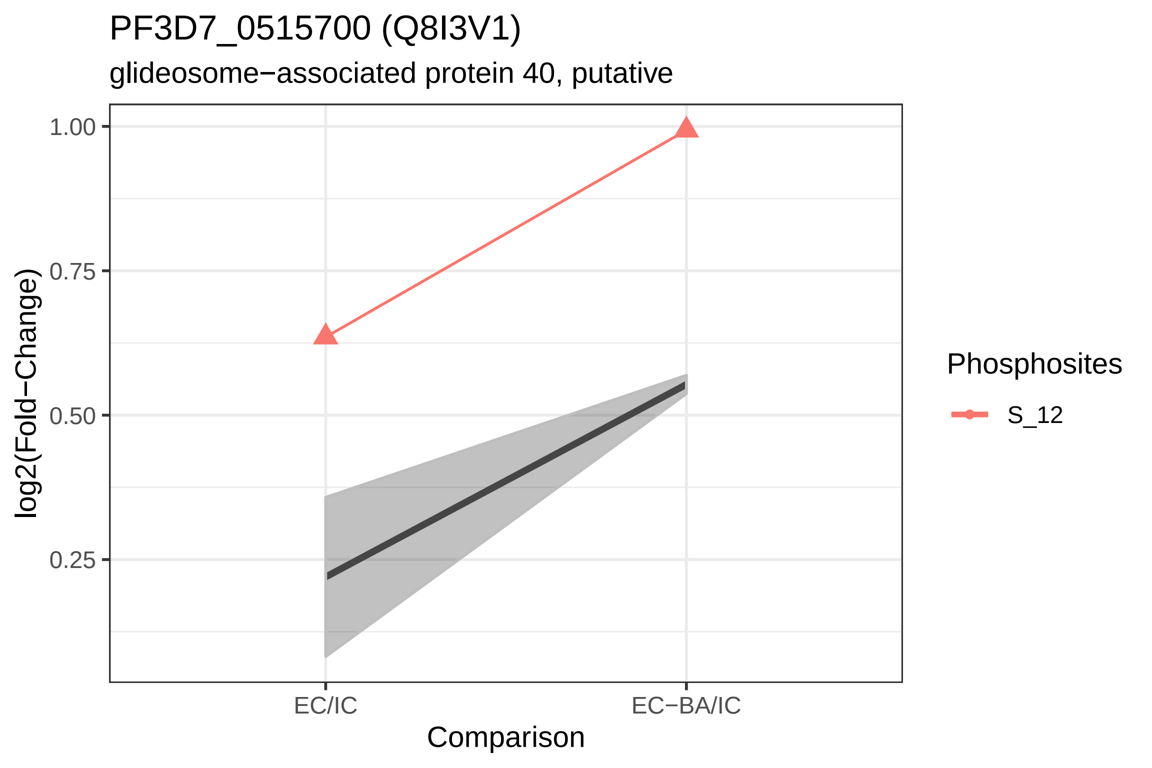
PF3D7_0515700 (Q8I3V1)
glideosome−associated protein 40, putative
log2(Fold−Change)
1.00
0.75
0.50
0.25
EC/IC
EC−BA/IC
Comparison
Phosphosites
S_12

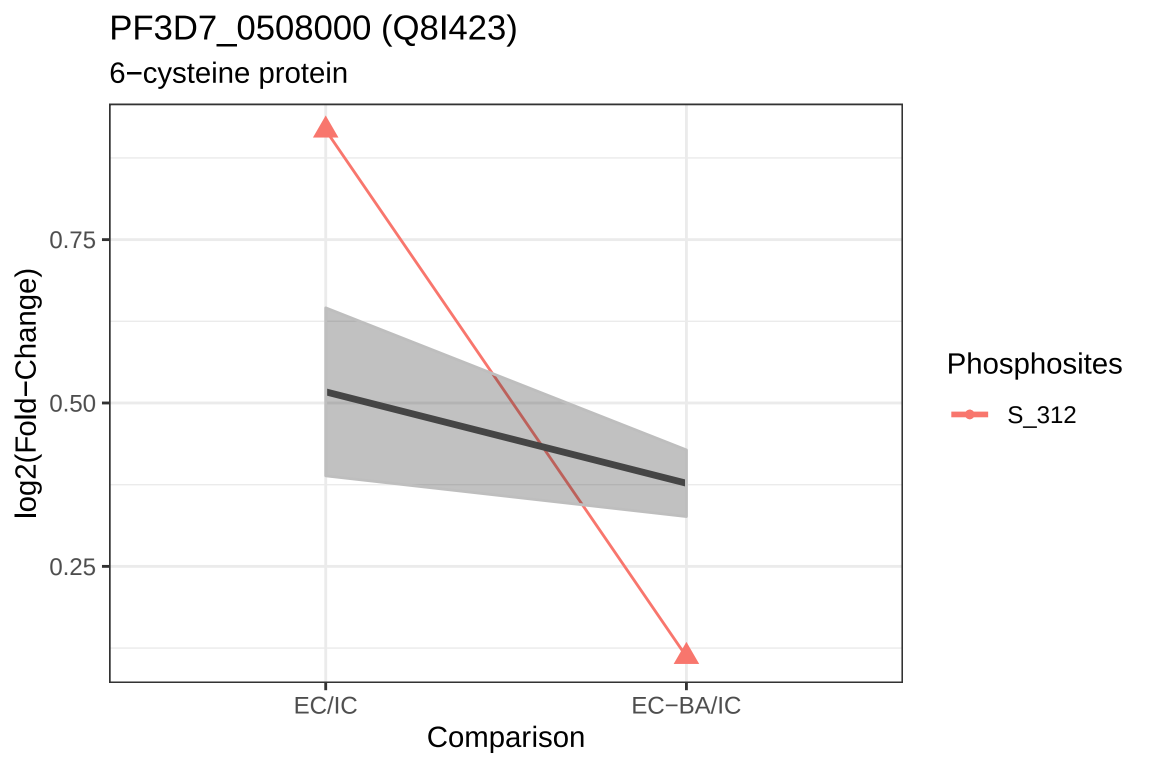

PF3D7_0508000 (Q8I423)
6−cysteine protein
log2(Fold−Change)
0.75
0.50
0.25
EC/IC
EC−BA/IC
Comparison
Phosphosites
S_312

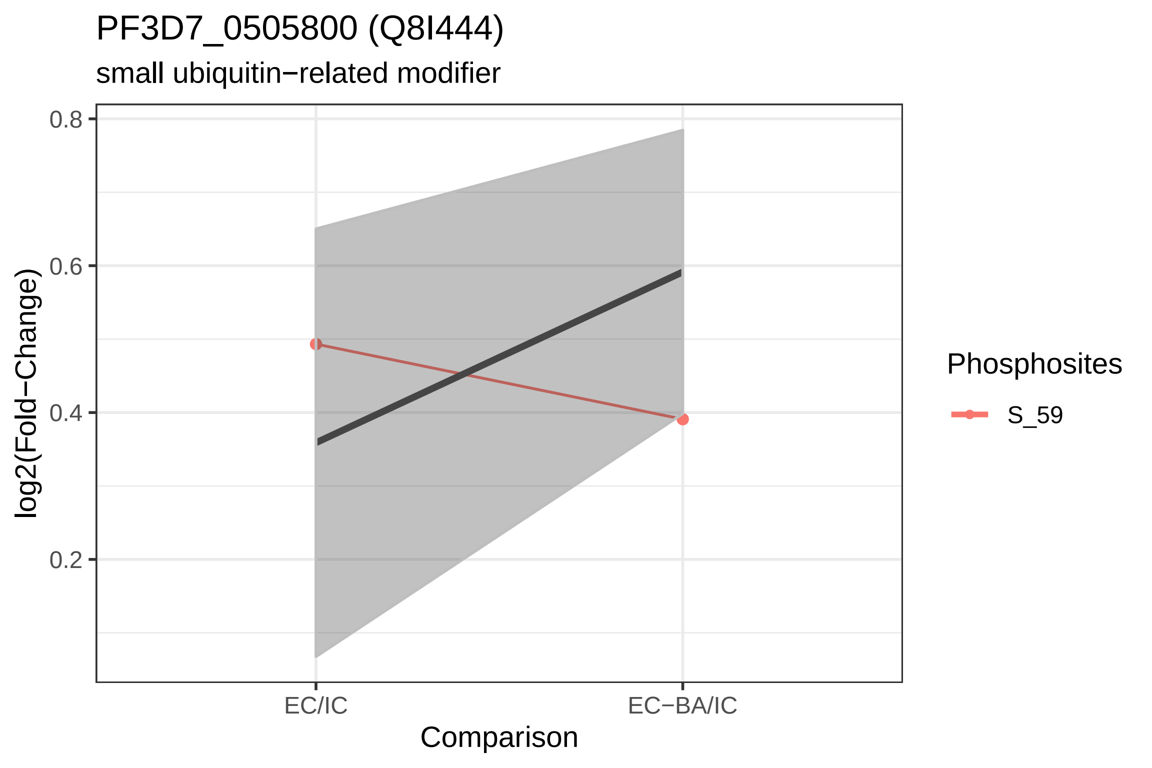
PF3D7_0505800 (Q8I444)
small ubiquitin−related modifier
0.8
0.6
0.4
0.2
log2(Fold−Change)
EC/IC
EC−BA/IC
Comparison
Phosphosites
S_59

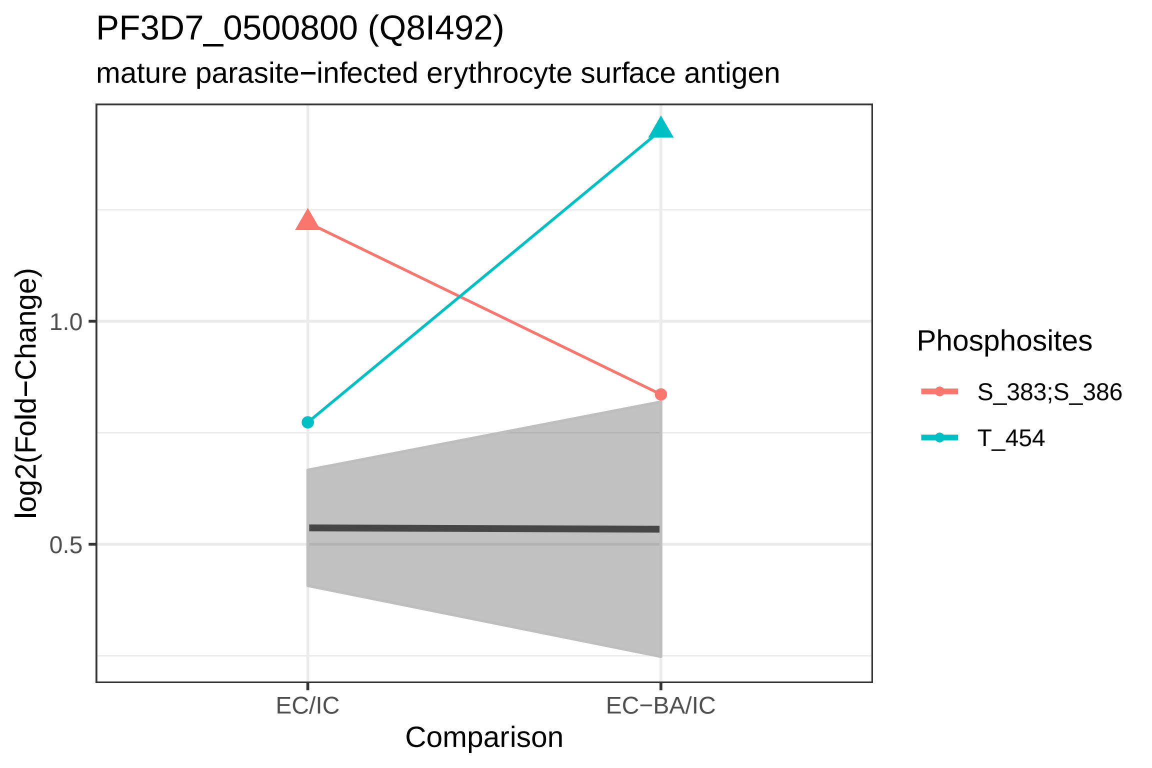
PF3D7_0500800 (Q8I492)
mature parasite−infected erythrocyte surface antigen
log2(Fold−Change)
1.0
0.5
EC/IC
EC−BA/IC
Comparison
Phosphosites
S_383;S_386
T_454

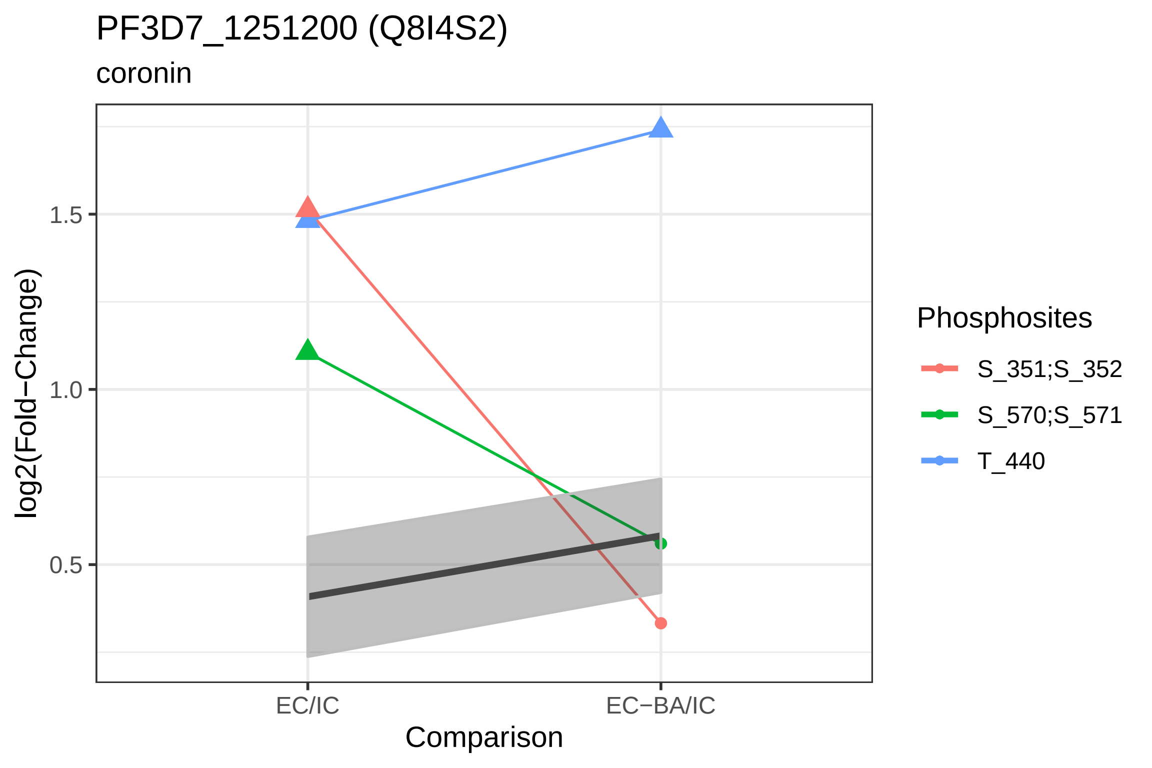

PF3D7_1251200 (Q8I4S2)
coronin
log2(Fold−Change)
1.5
1.0
0.5
EC/IC
EC−BA/IC
Comparison
Phosphosites
S_351;S_352
S_570;S_571
T_440

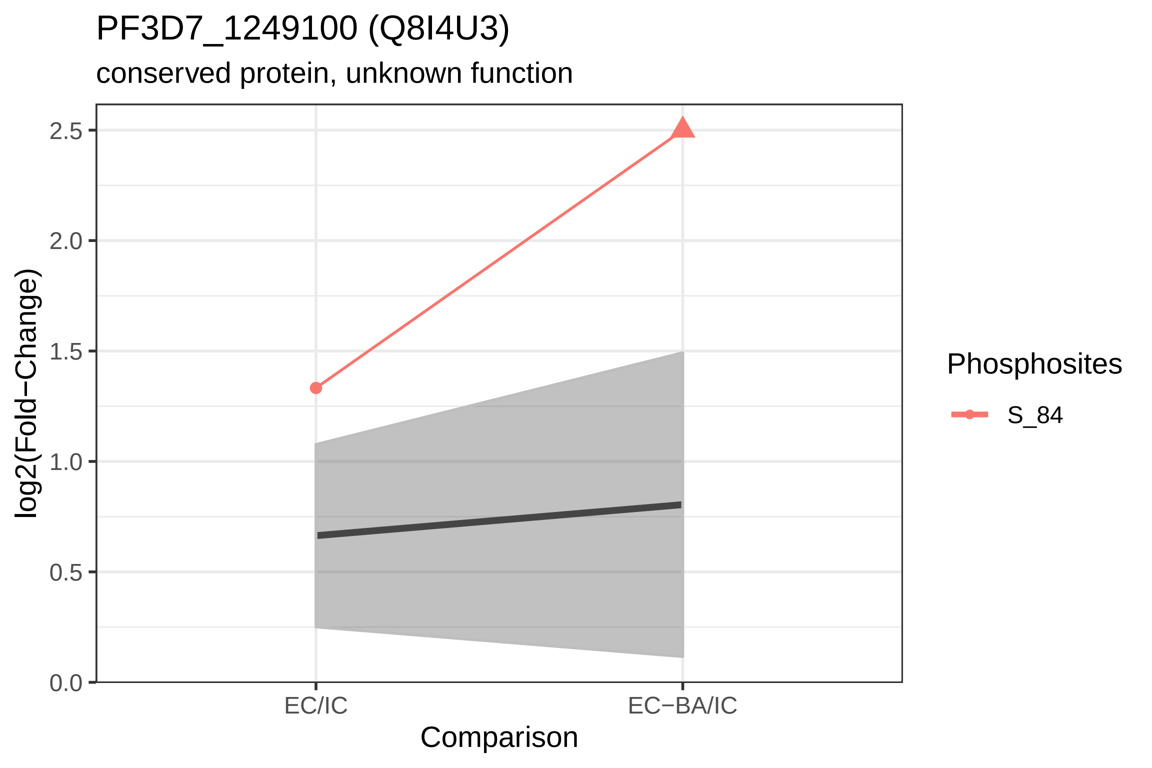
PF3D7_1249100 (Q8I4U3)
conserved protein, unknown function
log2(Fold−Change)
2.5
2.0
1.5
1.0
0.5
0.0
EC/IC
EC−BA/IC
Comparison
Phosphosites
S_84

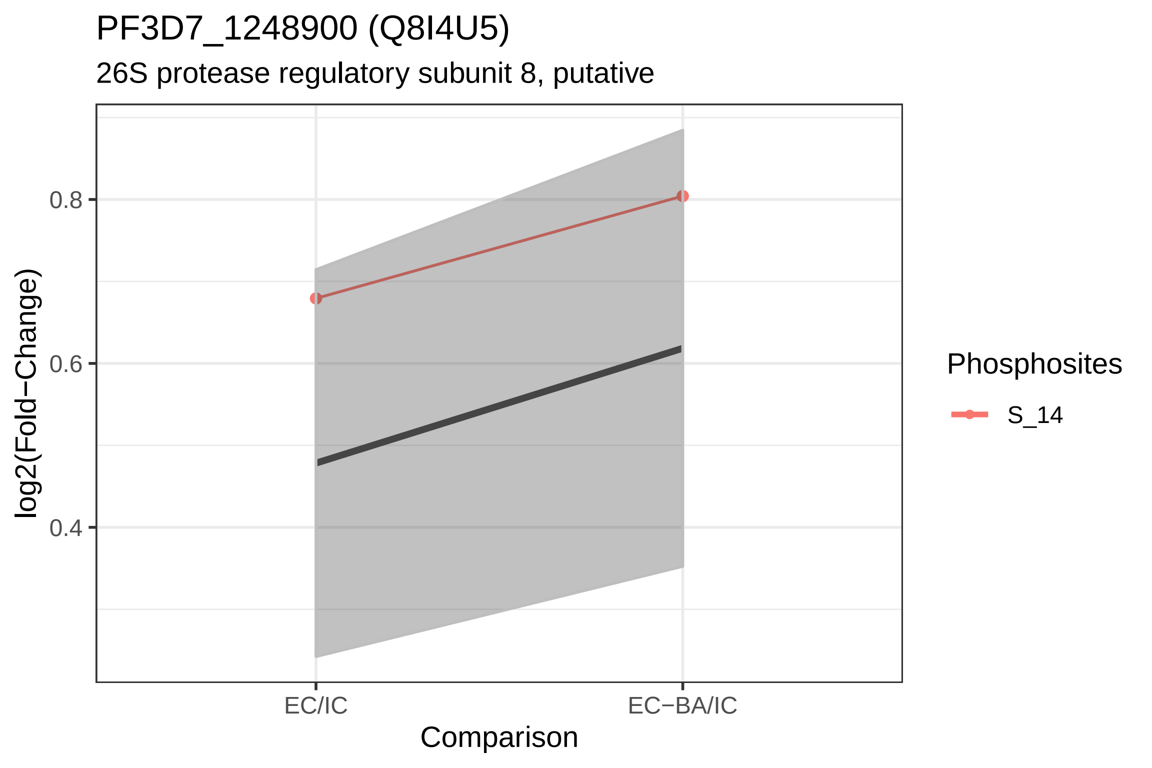

PF3D7_1248900 (Q8I4U5)
26S protease regulatory subunit 8, putative
log2(Fold−Change)
0.8
0.6
0.4
EC/IC
EC−BA/IC
Comparison
Phosphosites
S_14

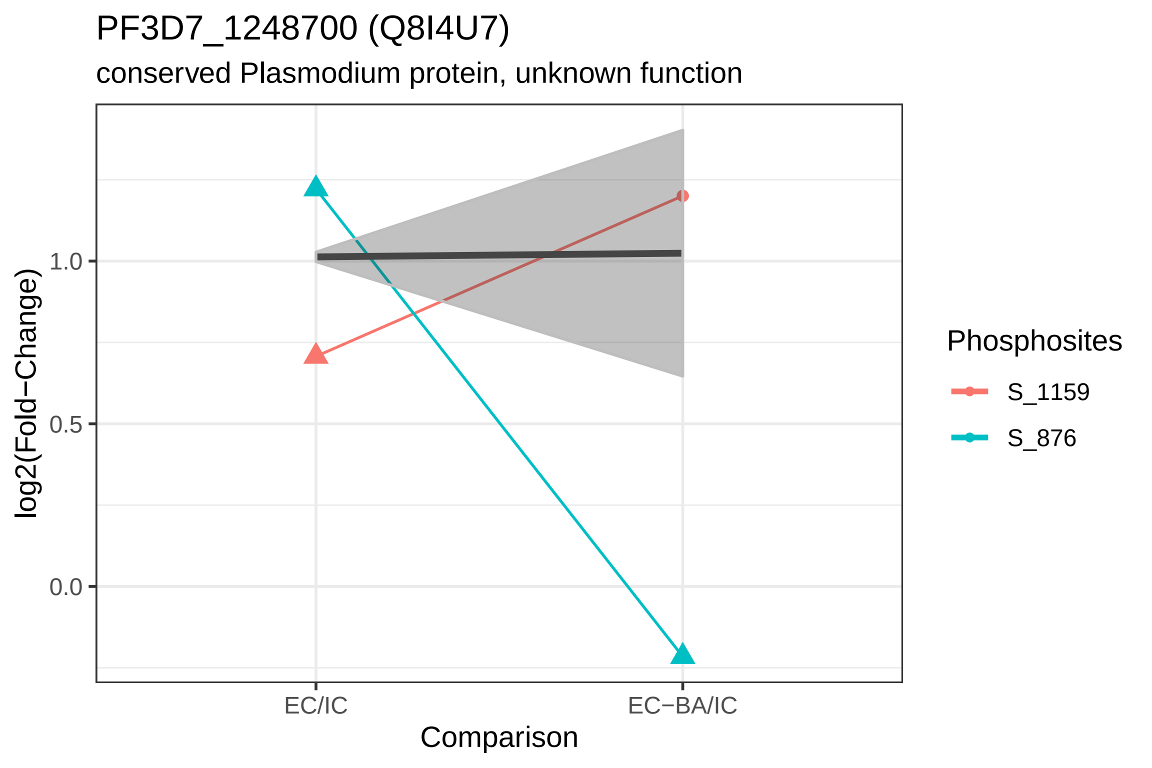
PF3D7_1248700 (Q8I4U7)
conserved Plasmodium protein, unknown function
log2(Fold−Change)
1.0
0.5
0.0
EC/IC
EC−BA/IC
Comparison
Phosphosites
S_1159
S_876

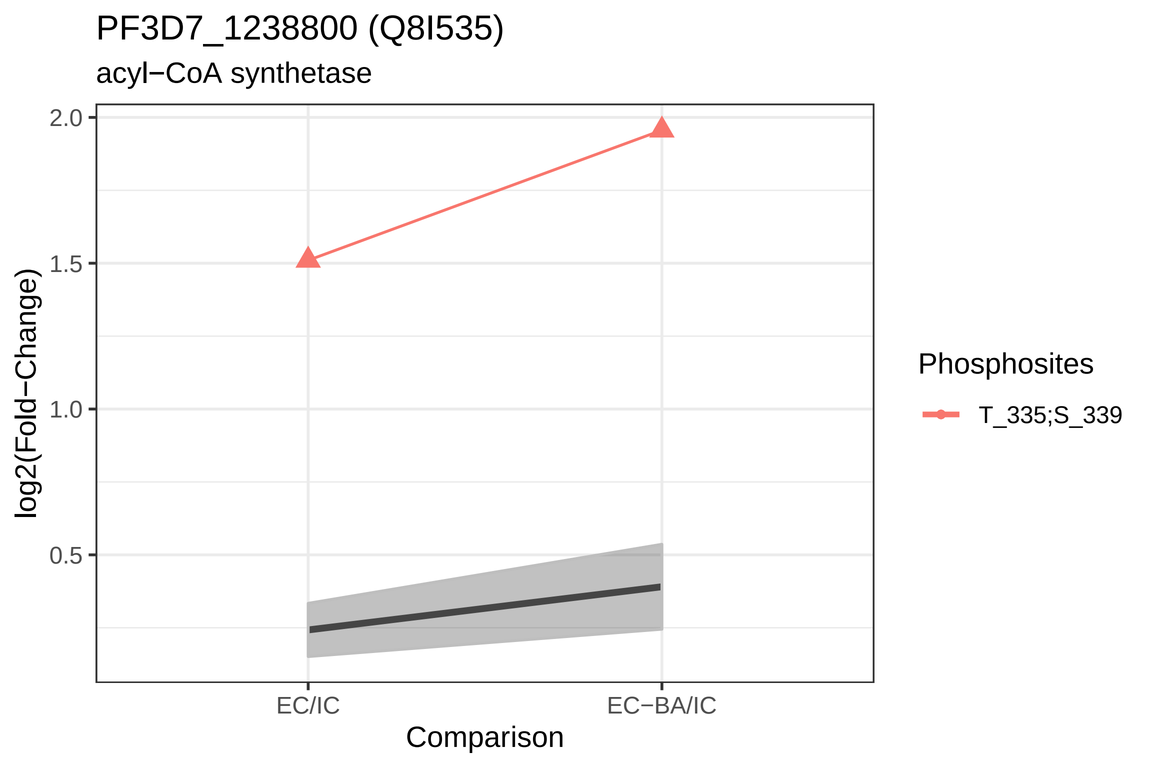

PF3D7_1238800 (Q8I535)
acyl−CoA synthetase
log2(Fold−Change)
2.0
1.5
1.0
0.5
EC/IC
EC−BA/IC
Comparison
Phosphosites
T_335;S_339

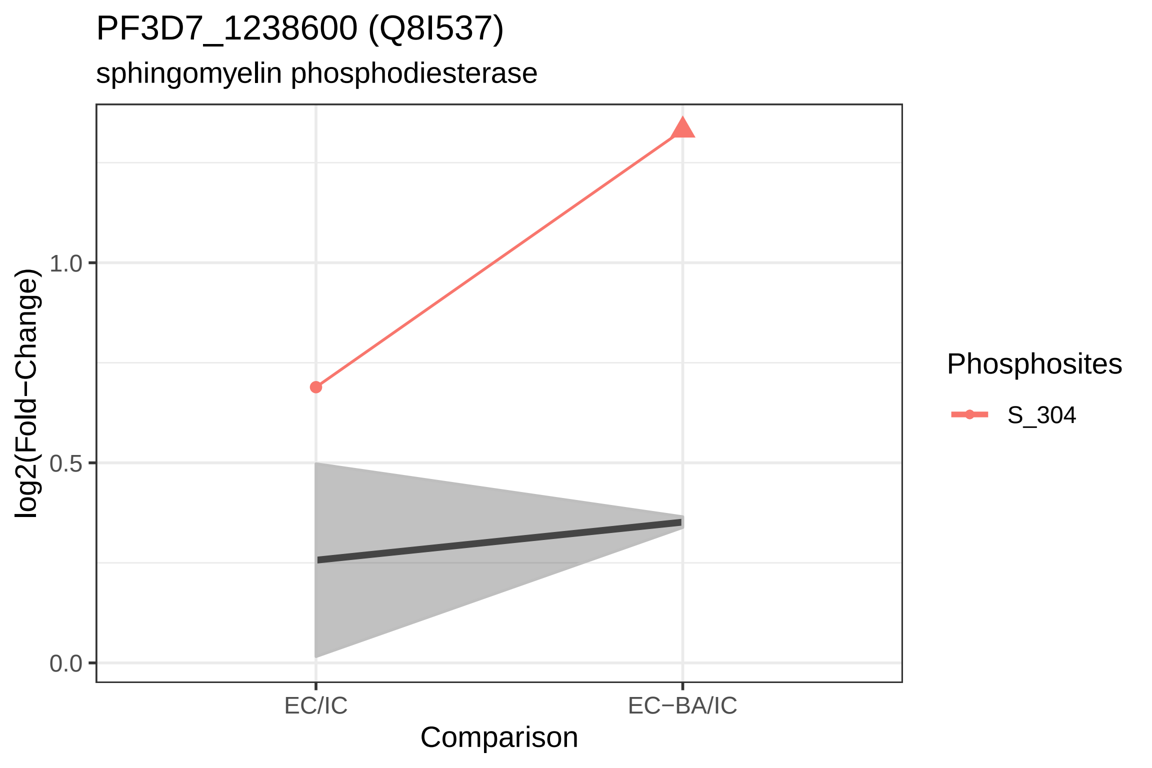

PF3D7_1238600 (Q8I537)
sphingomyelin phosphodiesterase
log2(Fold−Change)
1.0
0.5
0.0
EC/IC
EC−BA/IC
Comparison
Phosphosites
S_304

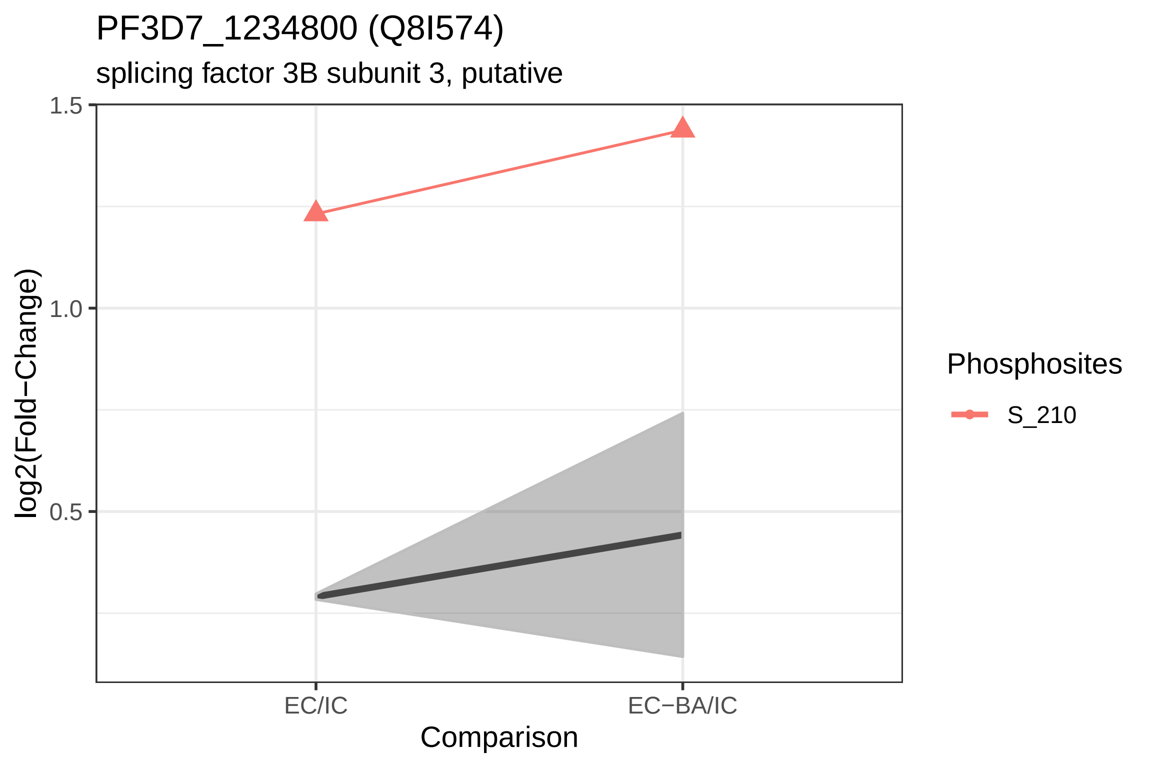

PF3D7_1234800 (Q8I574)
splicing factor 3B subunit 3, putative
1.5
1.0
0.5
log2(Fold−Change)
EC/IC
EC−BA/IC
Comparison
Phosphosites
S_210

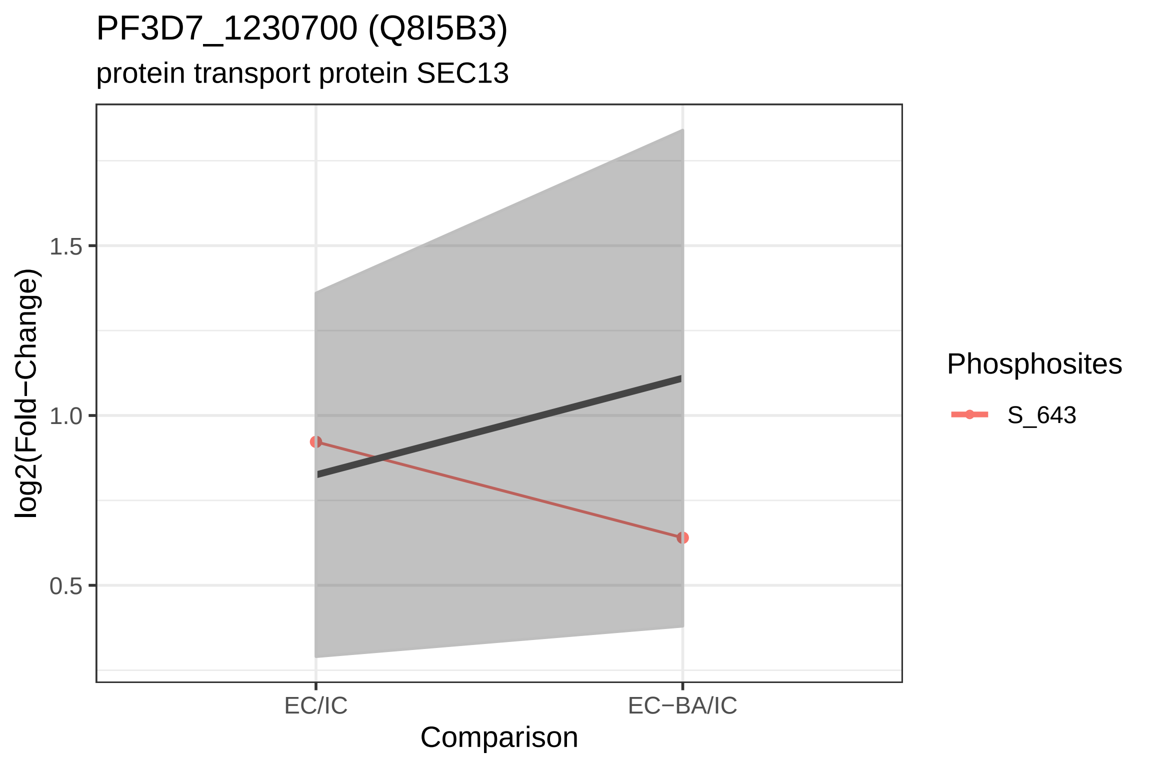
PF3D7_1230700 (Q8I5B3)
protein transport protein SEC13
log2(Fold−Change)
1.5
1.0
0.5
EC/IC
EC−BA/IC
Comparison
Phosphosites
S_643

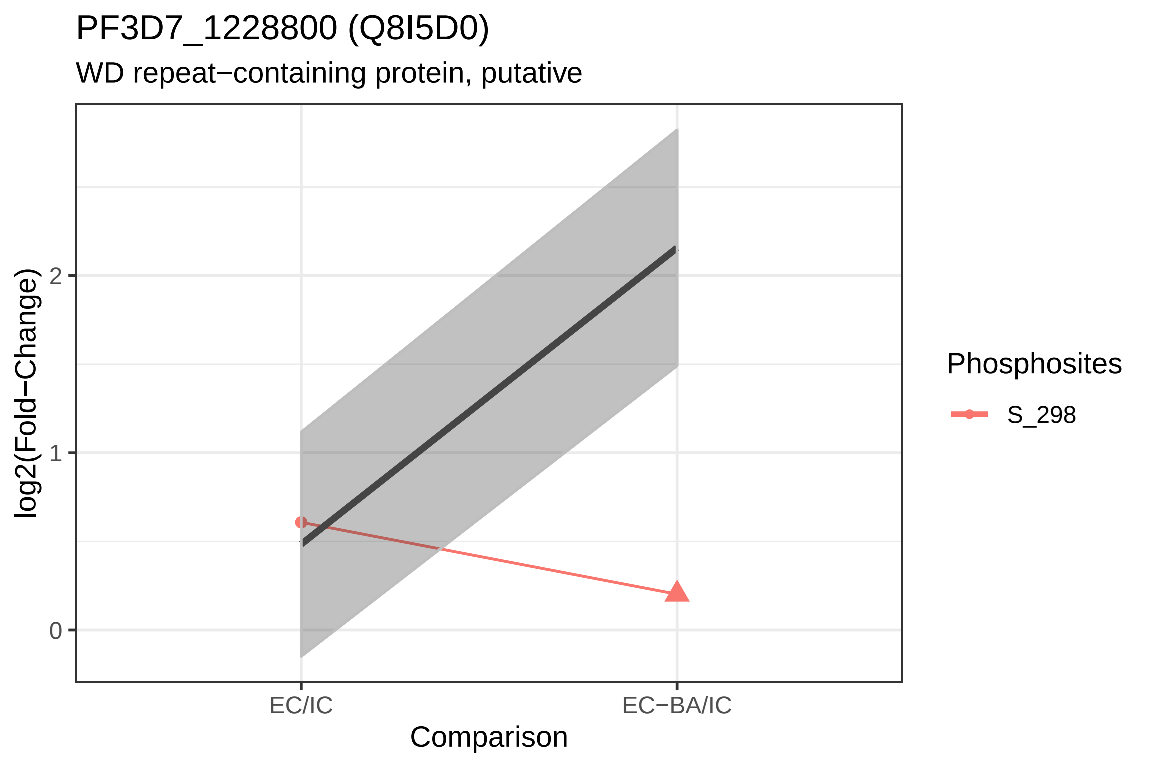
PF3D7_1228800 (Q8I5D0)
WD repeat−containing protein, putative
log2(Fold−Change)
2
1
0
EC/IC
EC−BA/IC
Comparison
Phosphosites
S_298

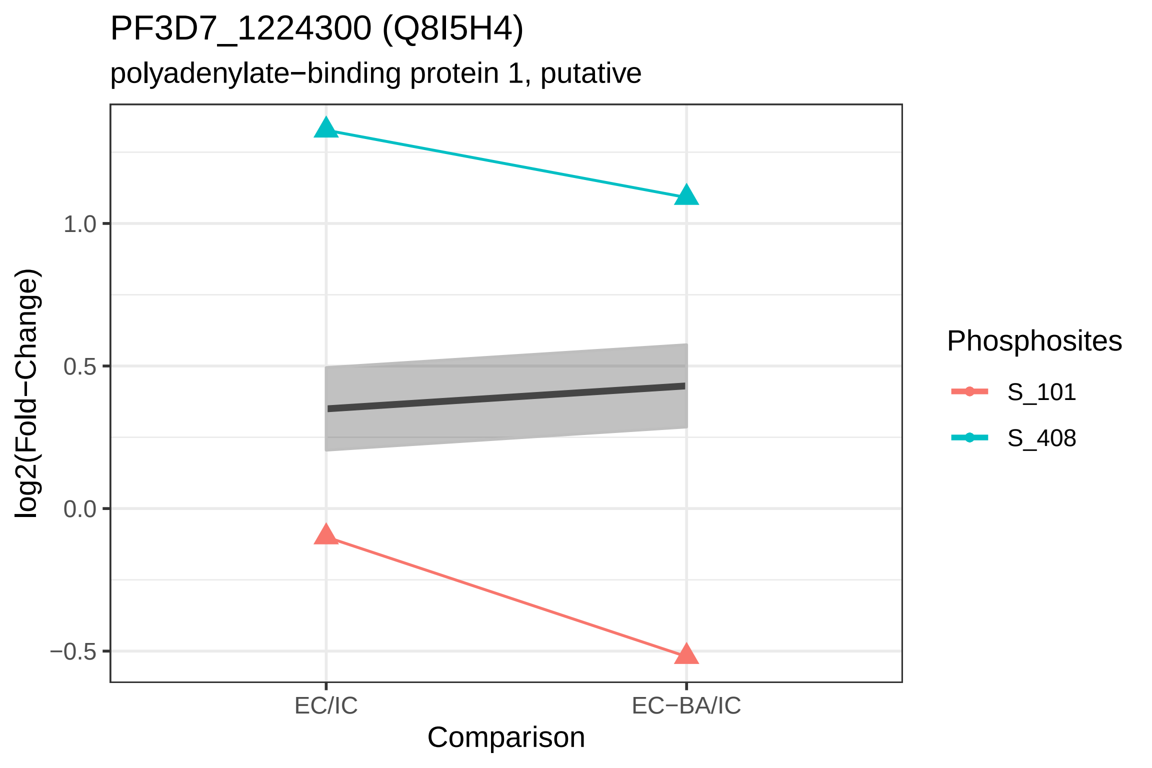
PF3D7_1224300 (Q8I5H4)
polyadenylate−binding protein 1, putative
log2(Fold−Change)
1.0
0.5
0.0
−0.5
EC/IC
EC−BA/IC
Comparison
Phosphosites
S_101
S_408

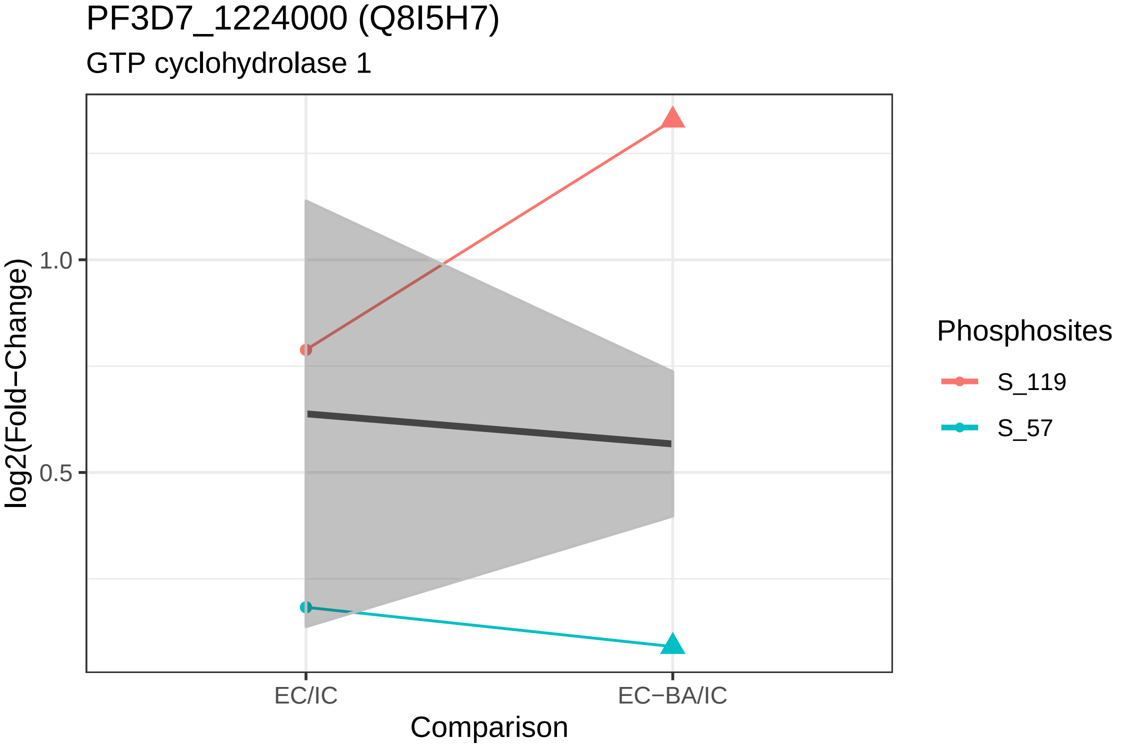

PF3D7_1224000 (Q8I5H7)
GTP cyclohydrolase 1
log2(Fold−Change)
1.0
0.5
EC/IC
EC−BA/IC
Comparison
Phosphosites
S_119
S_57

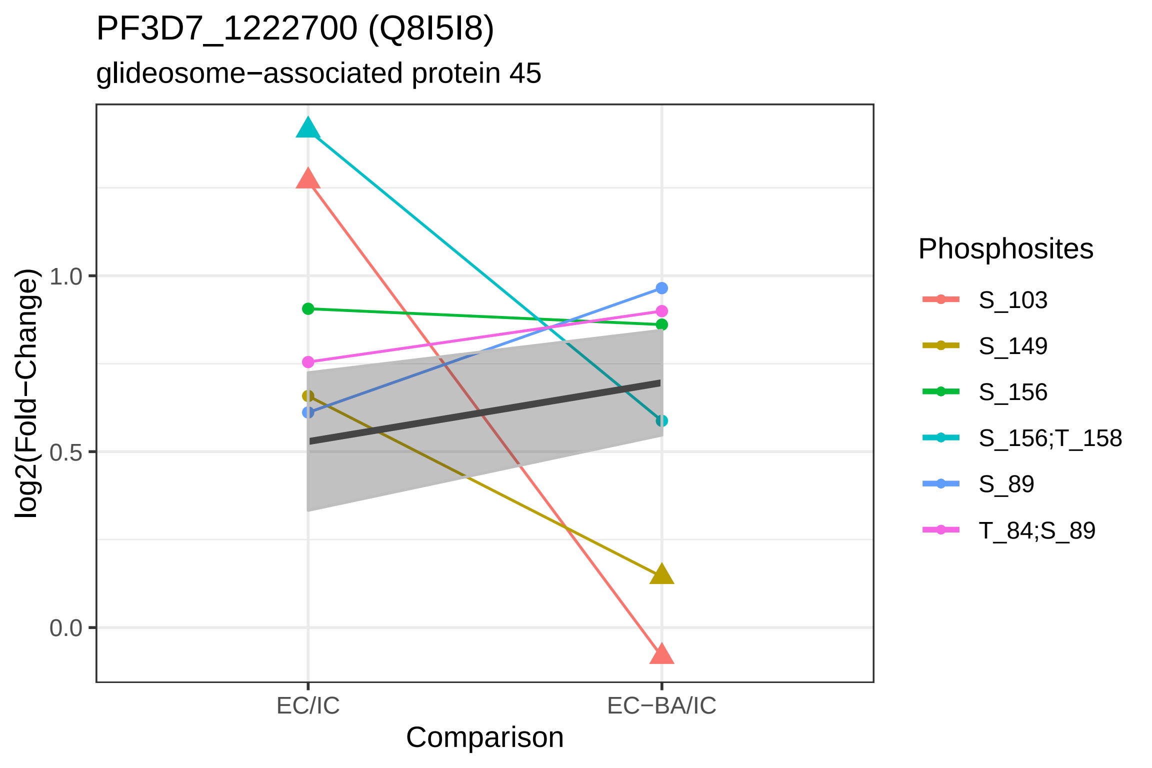

PF3D7_1222700 (Q8I5I8)
glideosome−associated protein 45
log2(Fold−Change)
1.0
0.5
0.0
EC/IC
EC−BA/IC
Comparison
Phosphosites
S_103
S_149
S_156
S_156;T_158
S_89
T_84;S_89

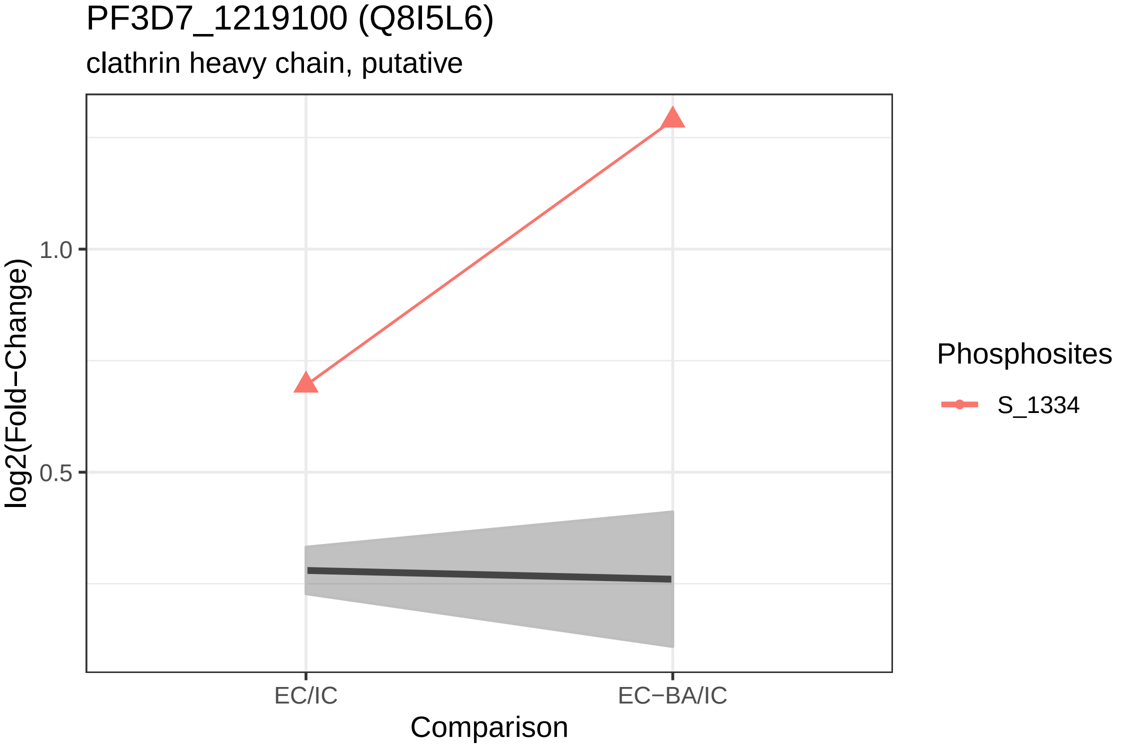

PF3D7_1219100 (Q8I5L6)
clathrin heavy chain, putative
log2(Fold−Change)
1.0
0.5
EC/IC
EC−BA/IC
Comparison
Phosphosites
S_1334

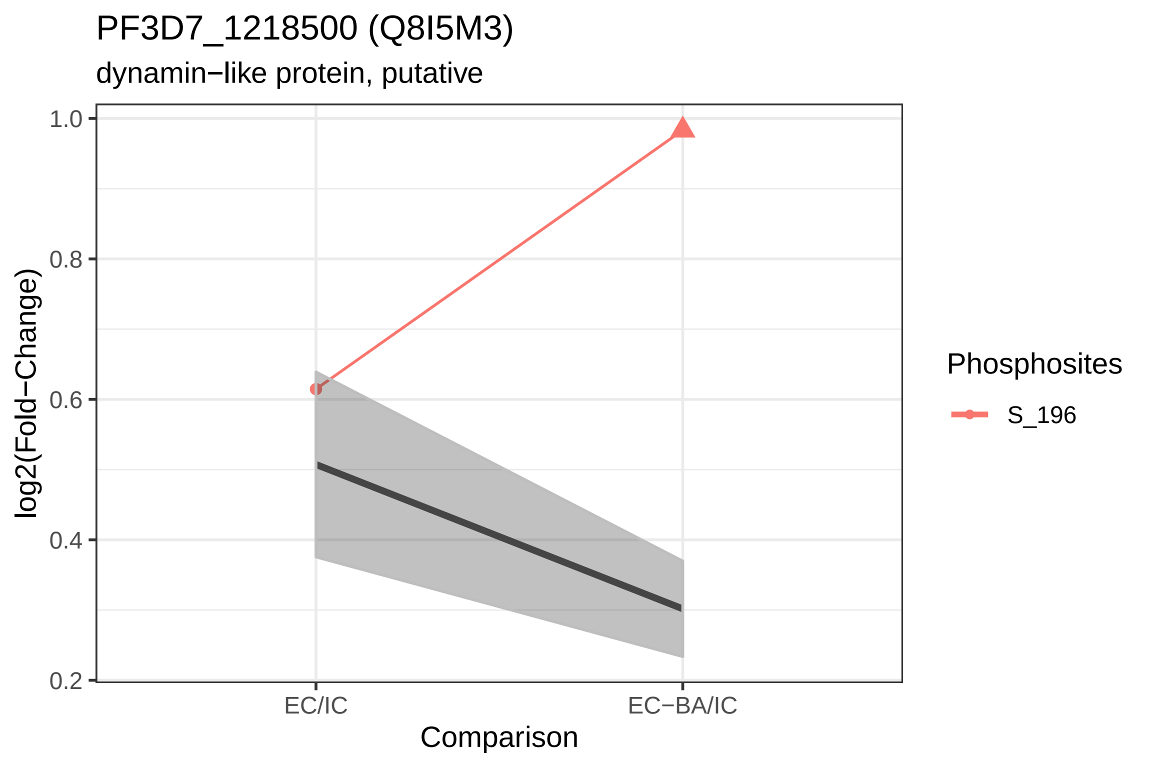
PF3D7_1218500 (Q8I5M3)
dynamin−like protein, putative
log2(Fold−Change)
1.0
0.8
0.6
0.4
0.2
EC/IC
EC−BA/IC
Comparison
Phosphosites
S_196

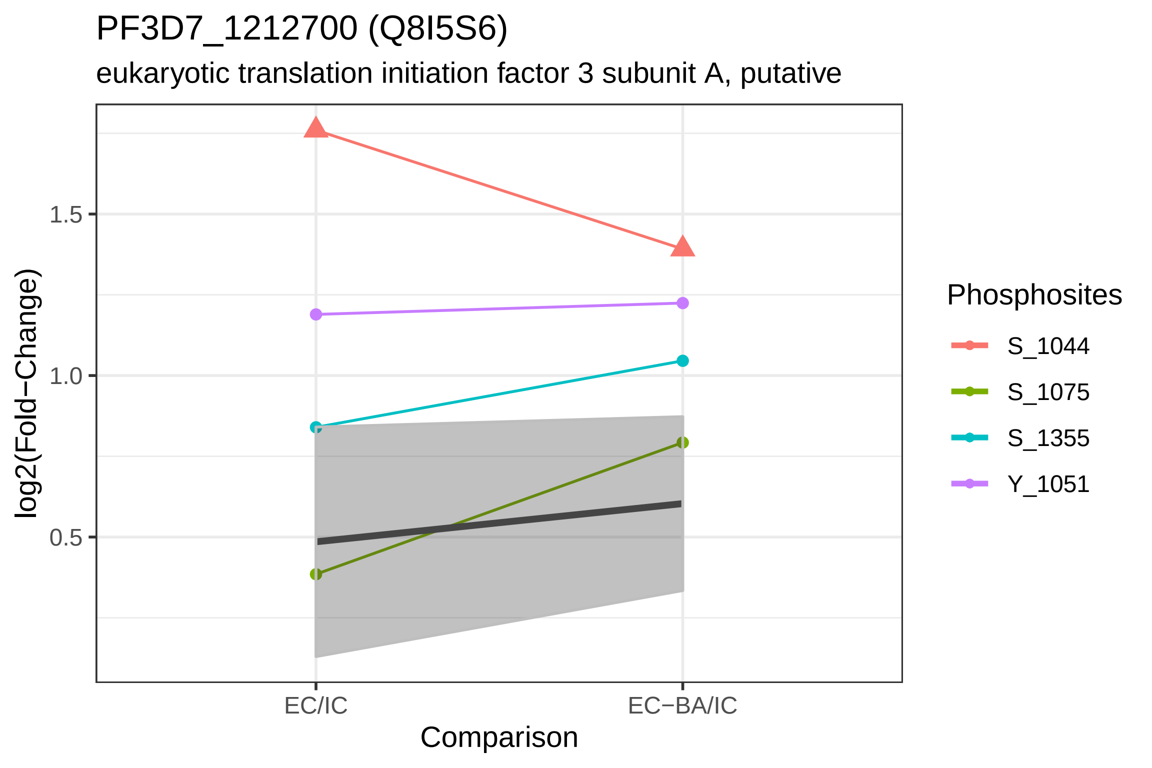

PF3D7_1212700 (Q8I5S6)
eukaryotic translation initiation factor 3 subunit A, putative
log2(Fold−Change)
1.5
1.0
0.5
EC/IC
EC−BA/IC
Comparison
Phosphosites
S_1044
S_1075
S_1355
Y_1051

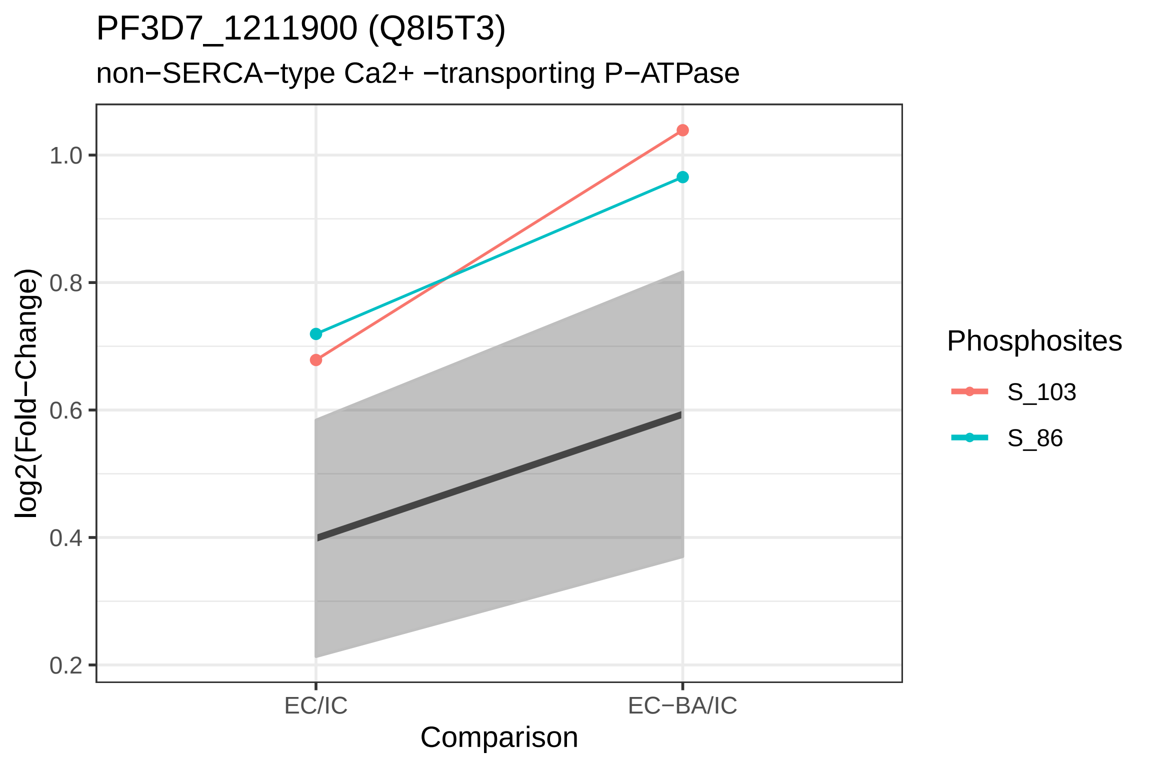
PF3D7_1211900 (Q8I5T3)
non−SERCA−type Ca2+ −transporting P−ATPase
log2(Fold−Change)
1.0
0.8
0.6
0.4
0.2
EC/IC
EC−BA/IC
Comparison
Phosphosites
S_103
S_86

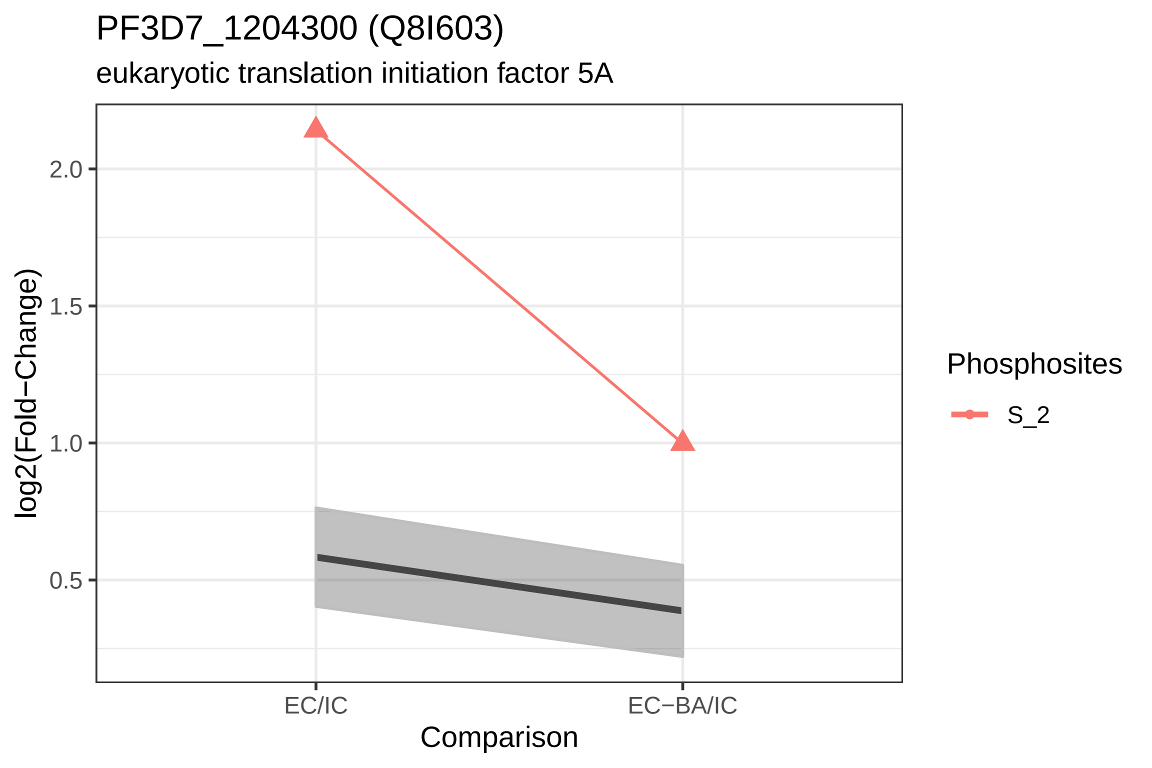

PF3D7_1204300 (Q8I603)
eukaryotic translation initiation factor 5A
log2(Fold−Change)
2.0
1.5
1.0
0.5
EC/IC
EC−BA/IC
Comparison
Phosphosites
S_2

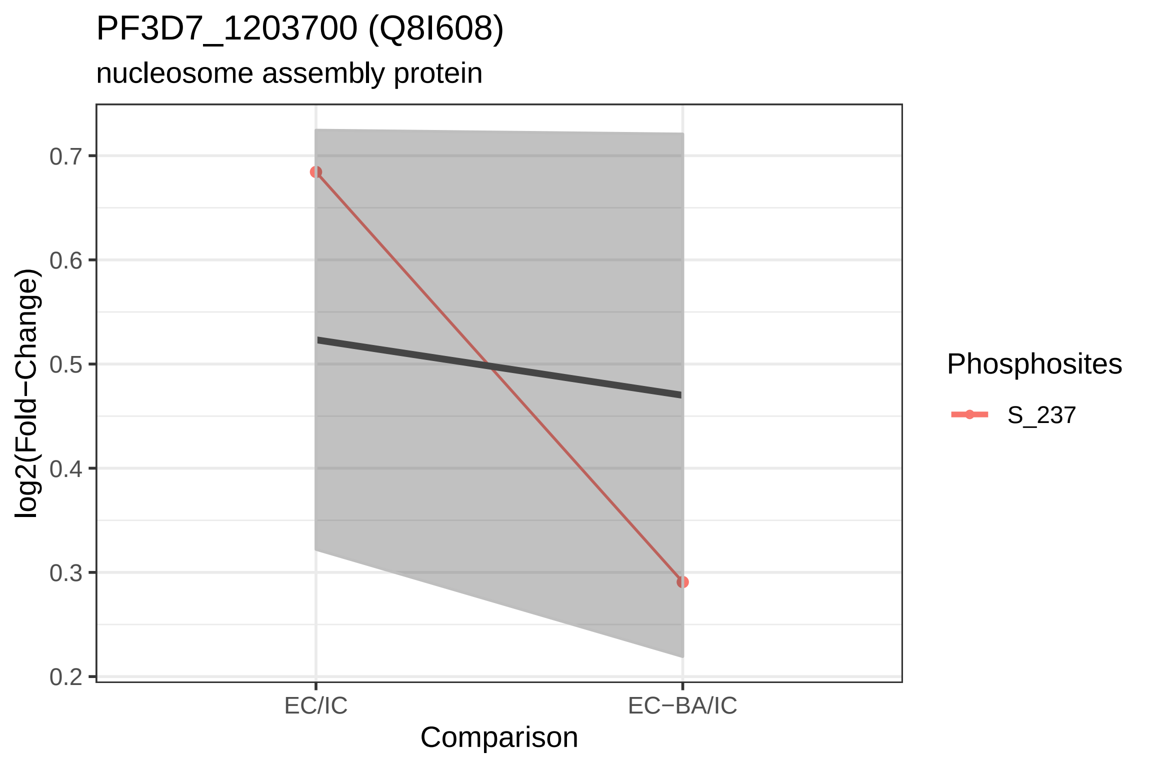

PF3D7_1203700 (Q8I608)
nucleosome assembly protein
log2(Fold−Change)
0.7
0.6
0.5
0.4
0.3
0.2
EC/IC
EC−BA/IC
Comparison
Phosphosites
S_237

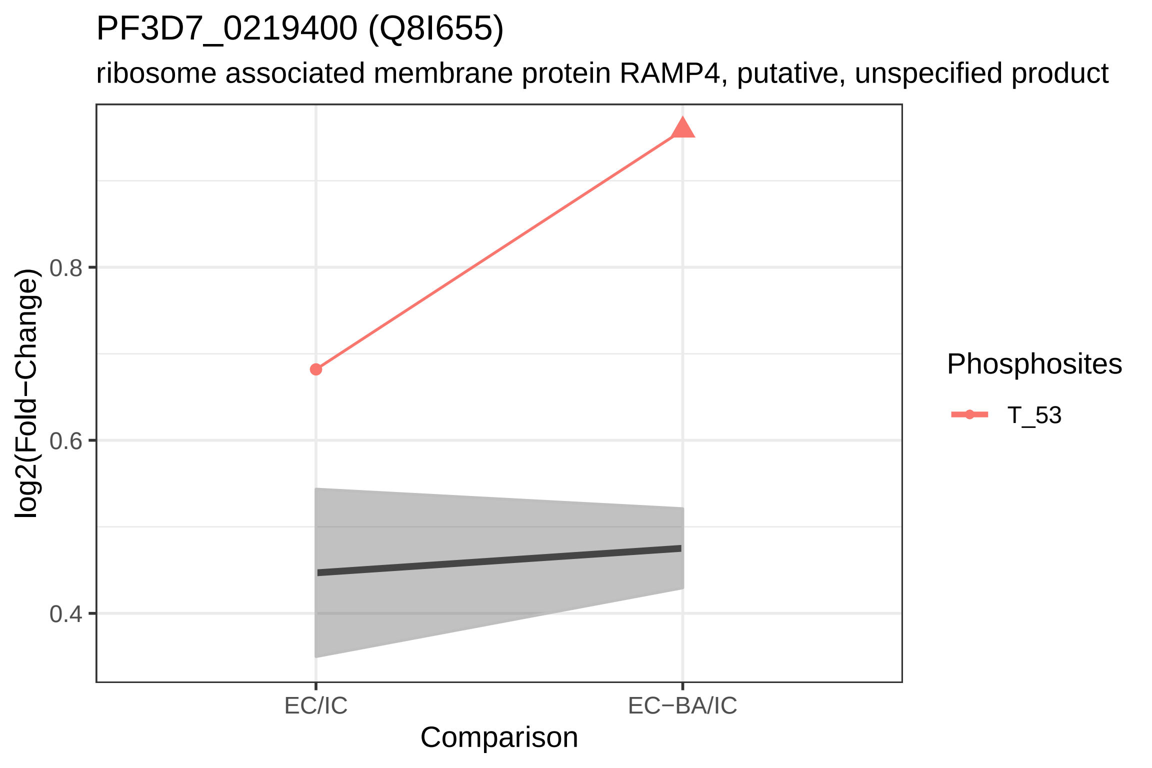
PF3D7_0219400 (Q8I655)
ribosome associated membrane protein RAMP4, putative, unspecified product
log2(Fold−Change)
0.8
0.6
0.4
EC/IC
EC−BA/IC
Comparison
Phosphosites
T_53

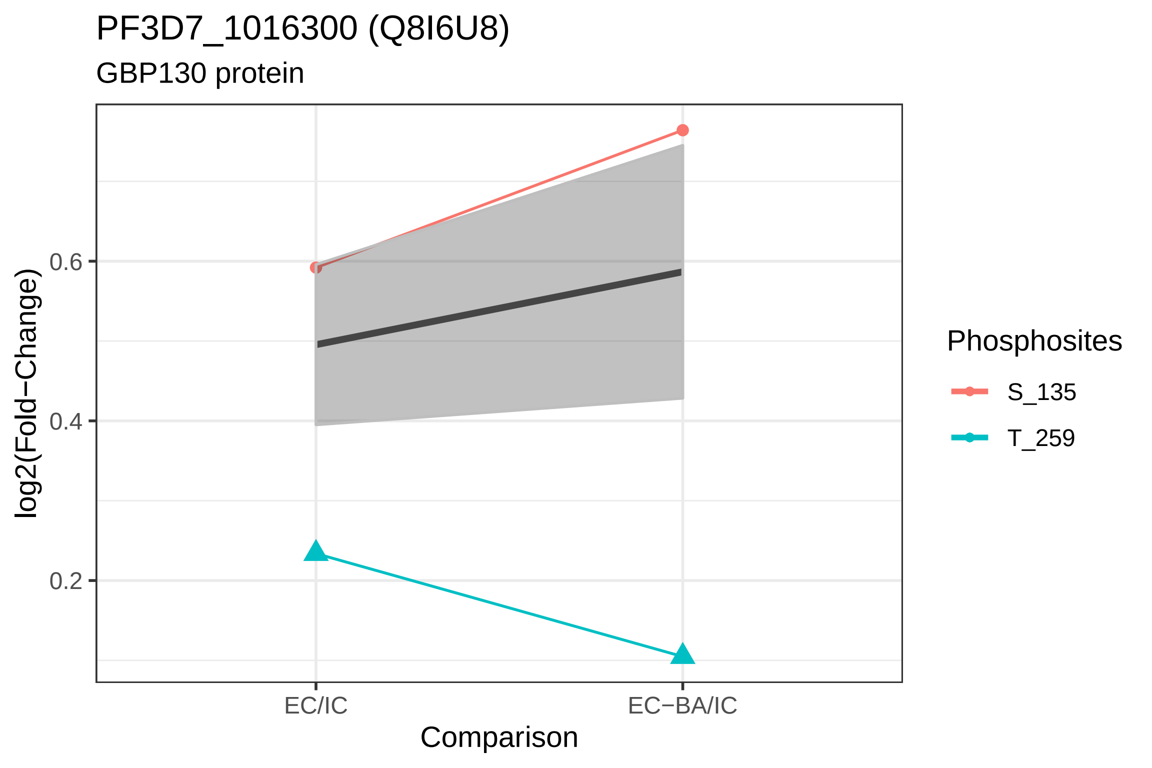

PF3D7_1016300 (Q8I6U8)
GBP130 protein
log2(Fold−Change)
0.6
0.4
0.2
EC/IC
EC−BA/IC
Comparison
Phosphosites
S_135
T_259

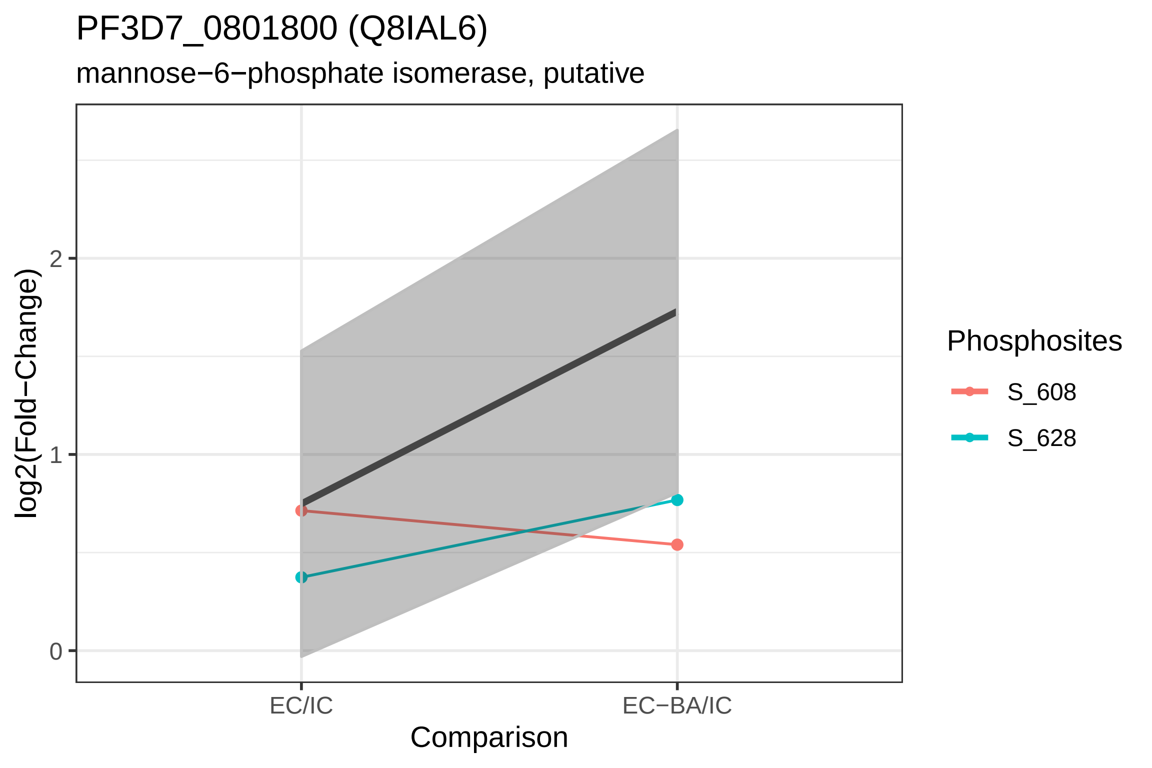

PF3D7_0801800 (Q8IAL6)
mannose−6−phosphate isomerase, putative
log2(Fold−Change)
2
1
0
EC/IC
EC−BA/IC
Comparison
Phosphosites
S_608
S_628

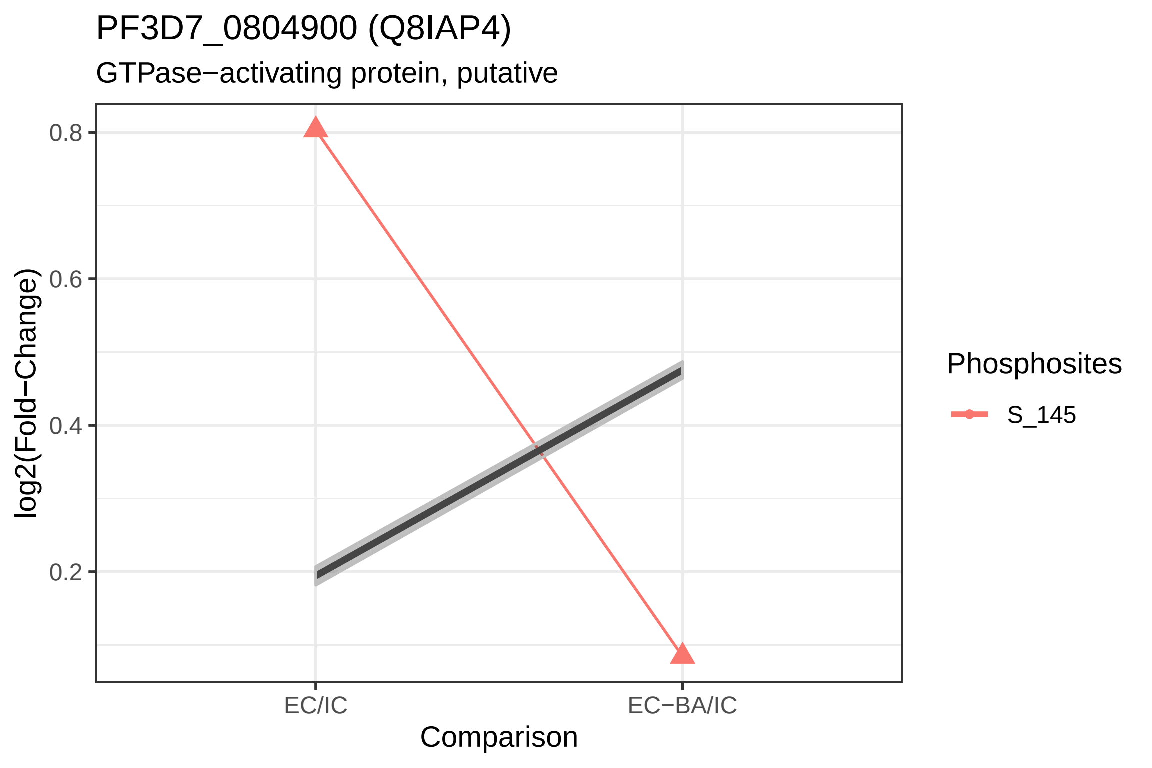
PF3D7_0804900 (Q8IAP4)
GTPase−activating protein, putative
log2(Fold−Change)
0.8
0.6
0.4
0.2
EC/IC
EC−BA/IC
Comparison
Phosphosites
S_145

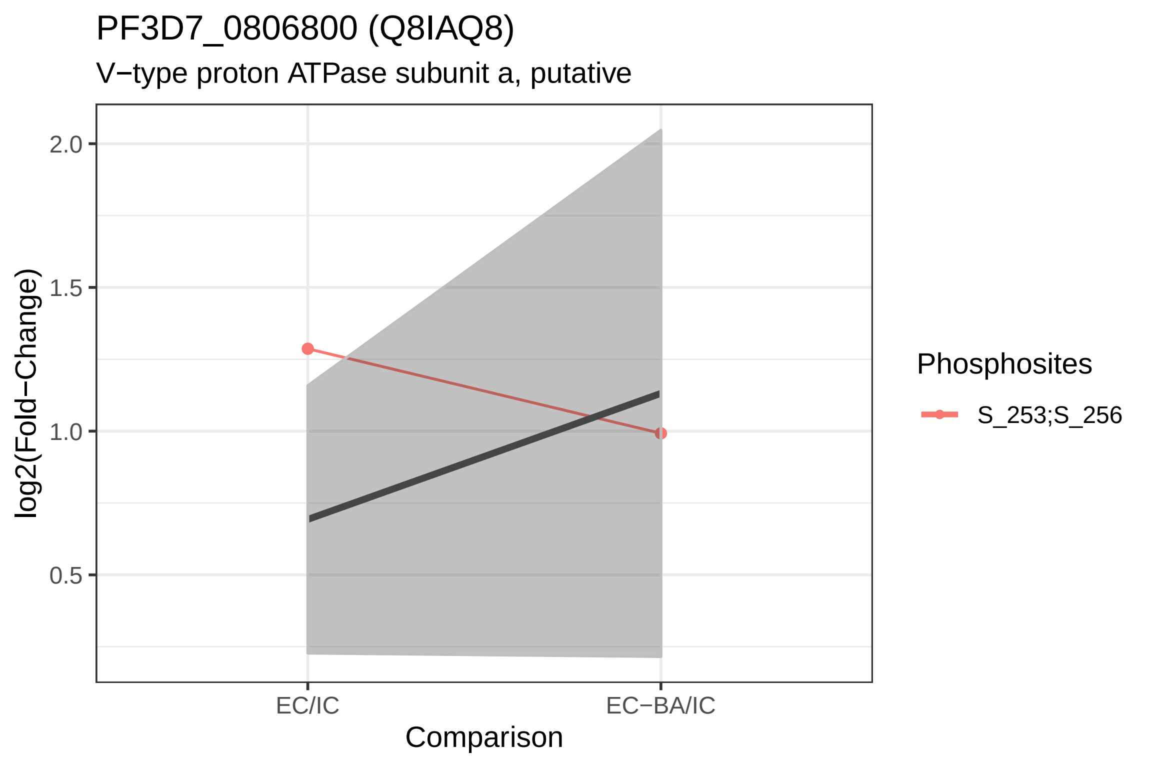

PF3D7_0806800 (Q8IAQ8)
V−type proton ATPase subunit a, putative
log2(Fold−Change)
2.0
1.5
1.0
0.5
EC/IC
EC−BA/IC
Comparison
Phosphosites
S_253;S_256

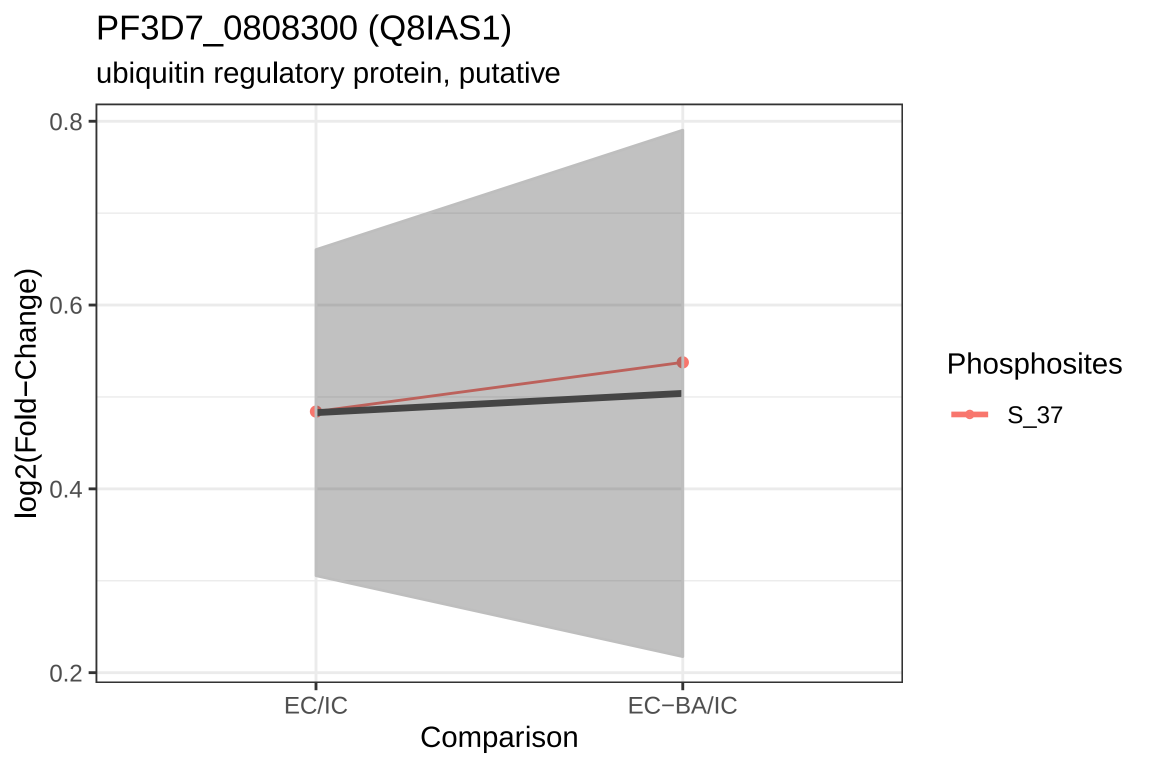

PF3D7_0808300 (Q8IAS1)
ubiquitin regulatory protein, putative
log2(Fold−Change)
0.8
0.6
0.4
0.2
EC/IC
EC−BA/IC
Comparison
Phosphosites
S_37

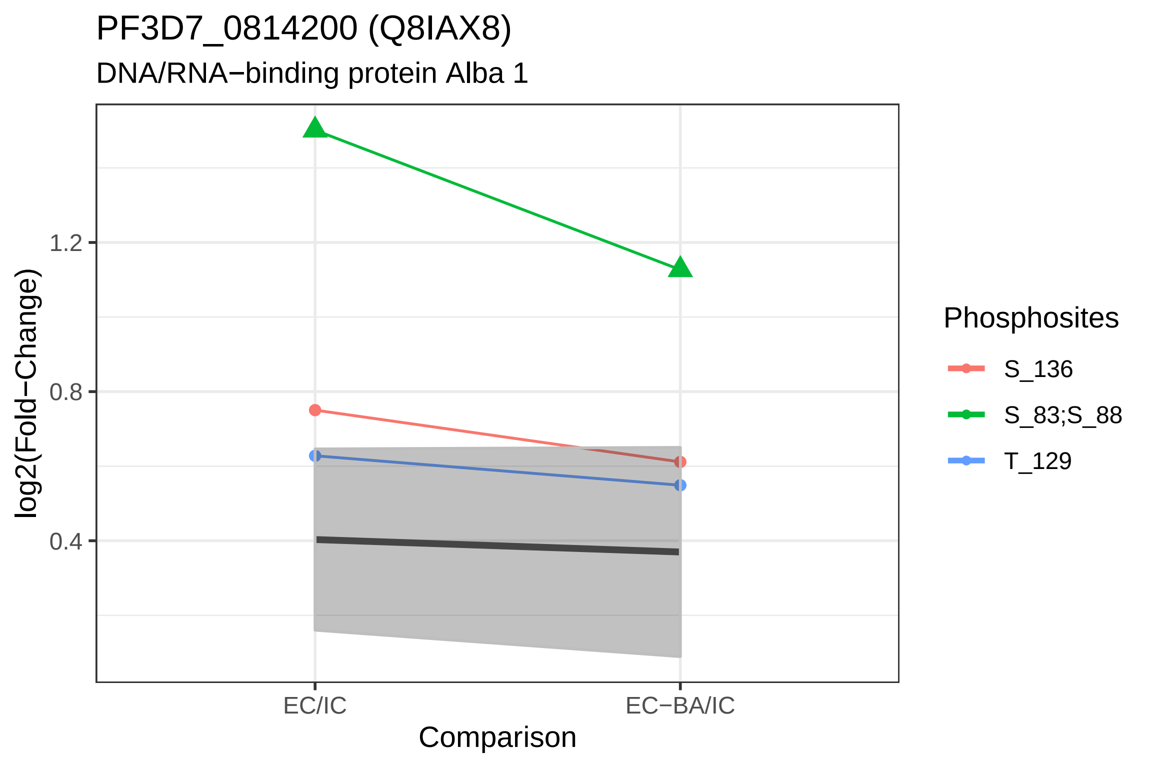

PF3D7_0814200 (Q8IAX8)
DNA/RNA−binding protein Alba 1
log2(Fold−Change)
1.2
0.8
0.4
EC/IC
EC−BA/IC
Comparison
Phosphosites
S_136
S_83;S_88
T_129

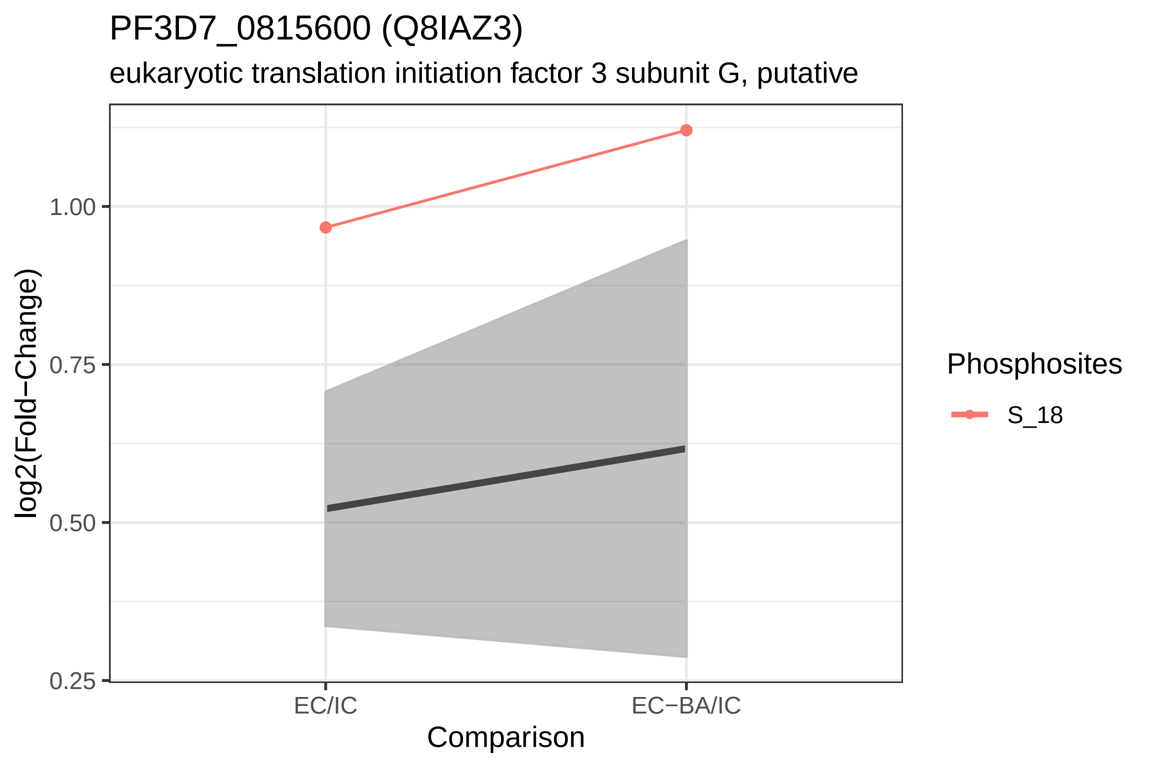
PF3D7_0815600 (Q8IAZ3)
eukaryotic translation initiation factor 3 subunit G, putative
log2(Fold−Change)
1.00
0.75
0.50
0.25
EC/IC
EC−BA/IC
Comparison
Phosphosites
S_18

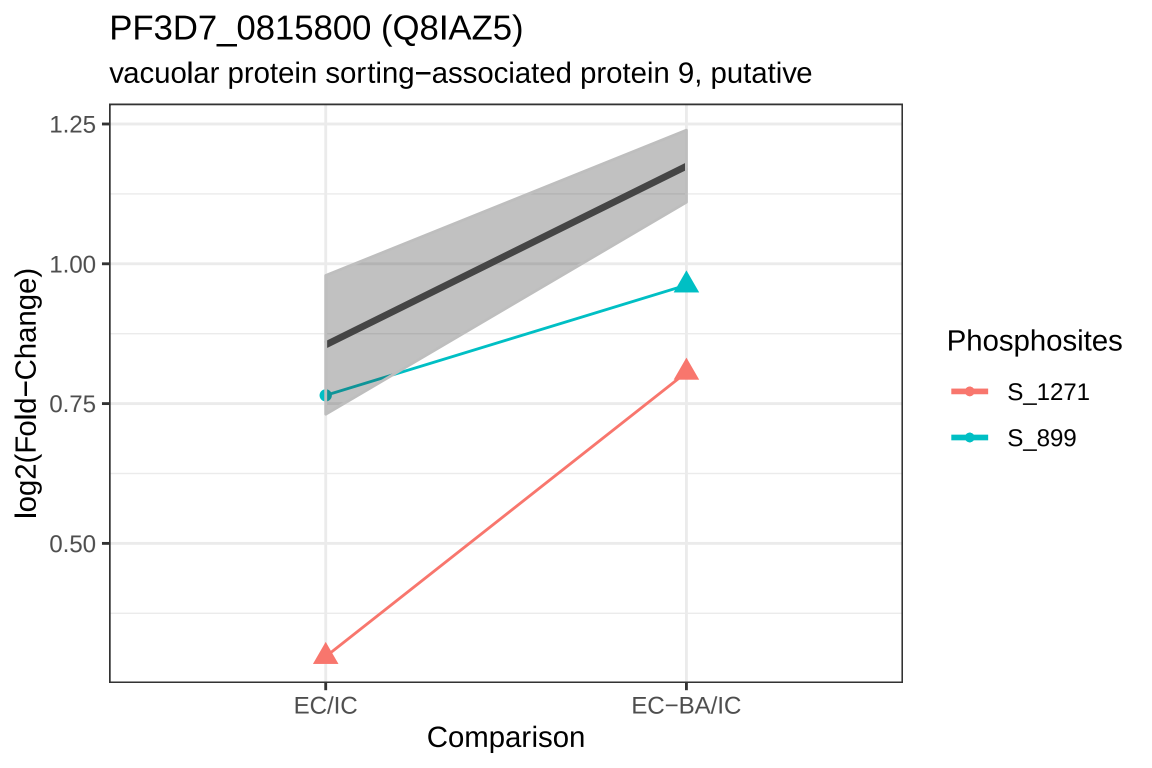
PF3D7_0815800 (Q8IAZ5)
vacuolar protein sorting−associated protein 9, putative
log2(Fold−Change)
1.25
1.00
0.75
0.50
EC/IC
EC−BA/IC
Comparison
Phosphosites
S_1271
S_899

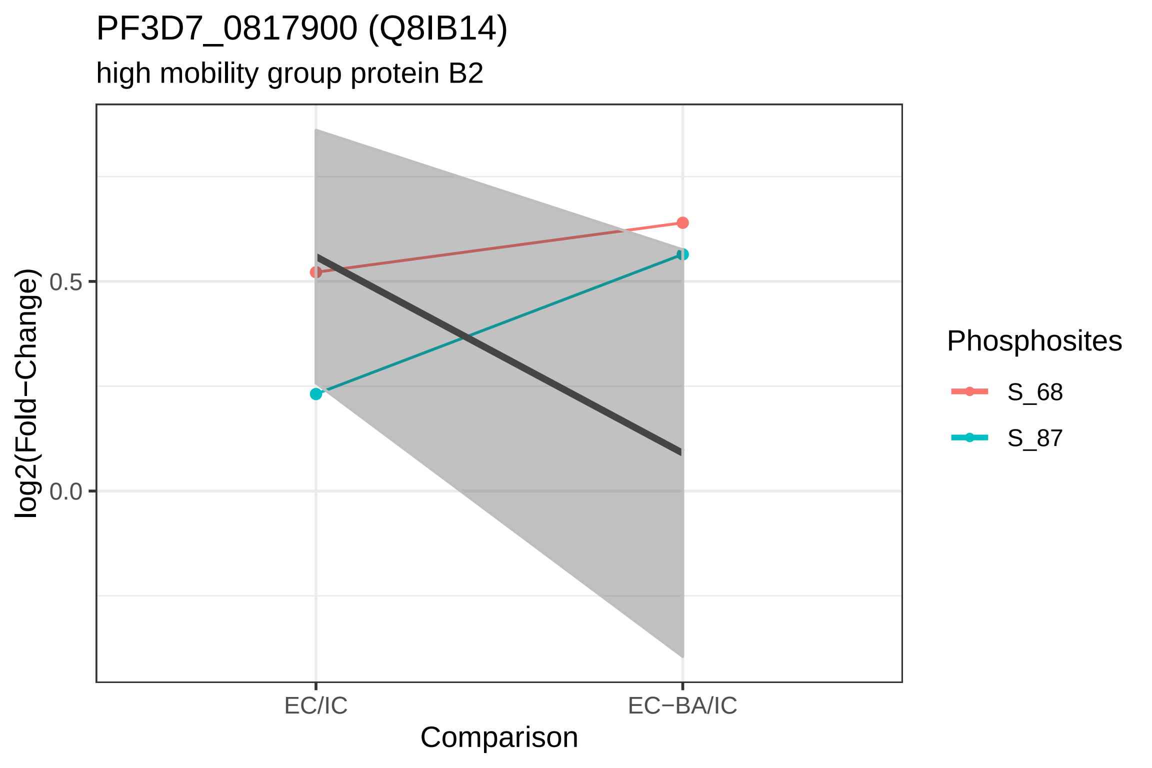

PF3D7_0817900 (Q8IB14)
high mobility group protein B2
log2(Fold−Change)
0.5
0.0
EC/IC
EC−BA/IC
Comparison
Phosphosites
S_68
S_87

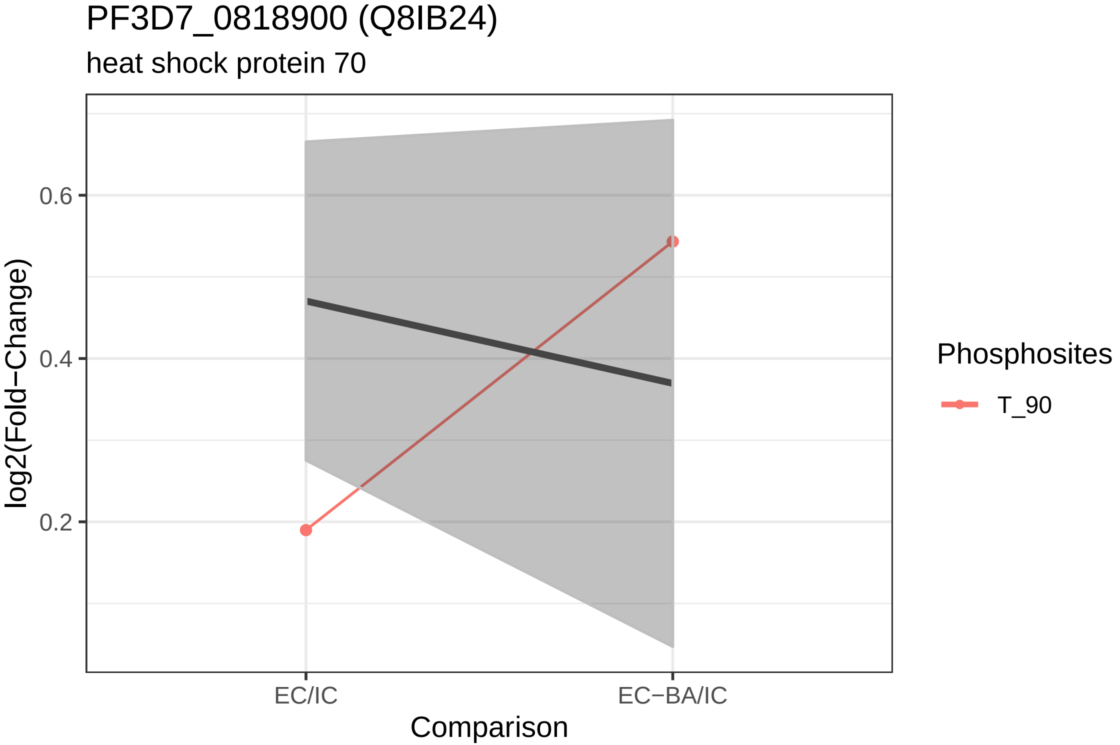

PF3D7_0818900 (Q8IB24)
heat shock protein 70
log2(Fold−Change)
0.6
0.4
0.2
EC/IC
EC−BA/IC
Comparison
Phosphosites
T_90

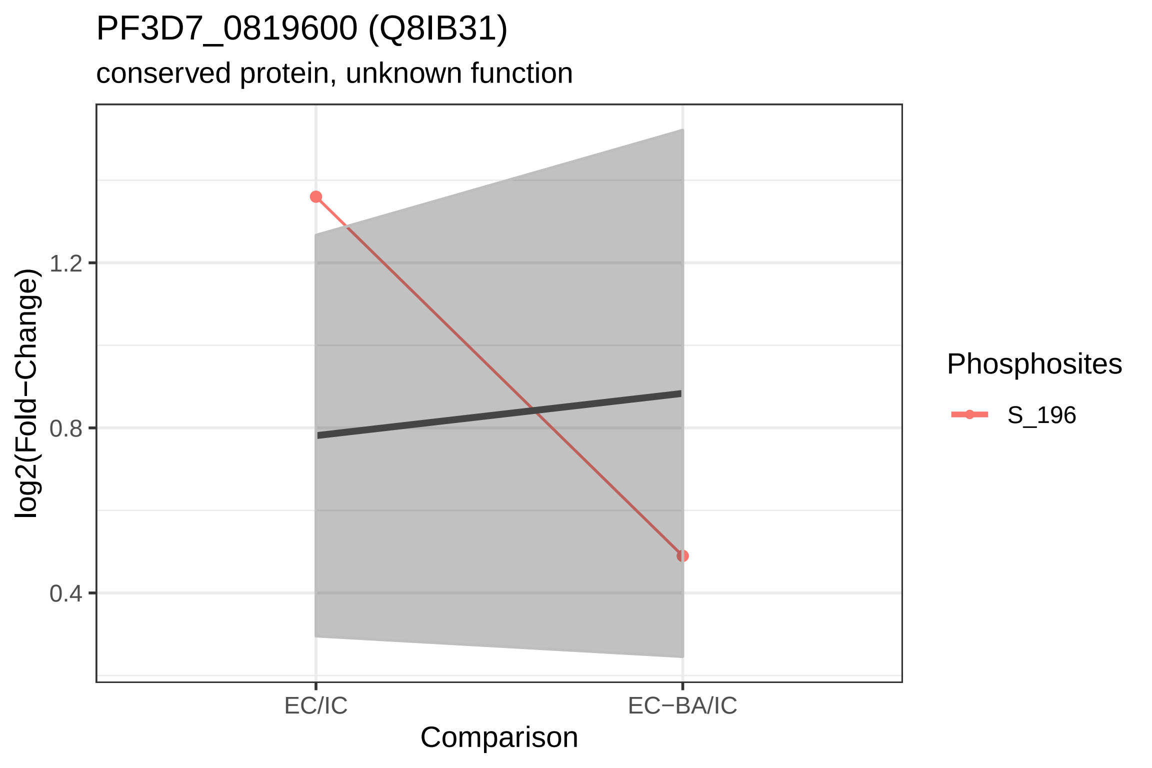

PF3D7_0819600 (Q8IB31)
conserved protein, unknown function
log2(Fold−Change)
1.2
0.8
0.4
EC/IC
EC−BA/IC
Comparison
Phosphosites
S_196

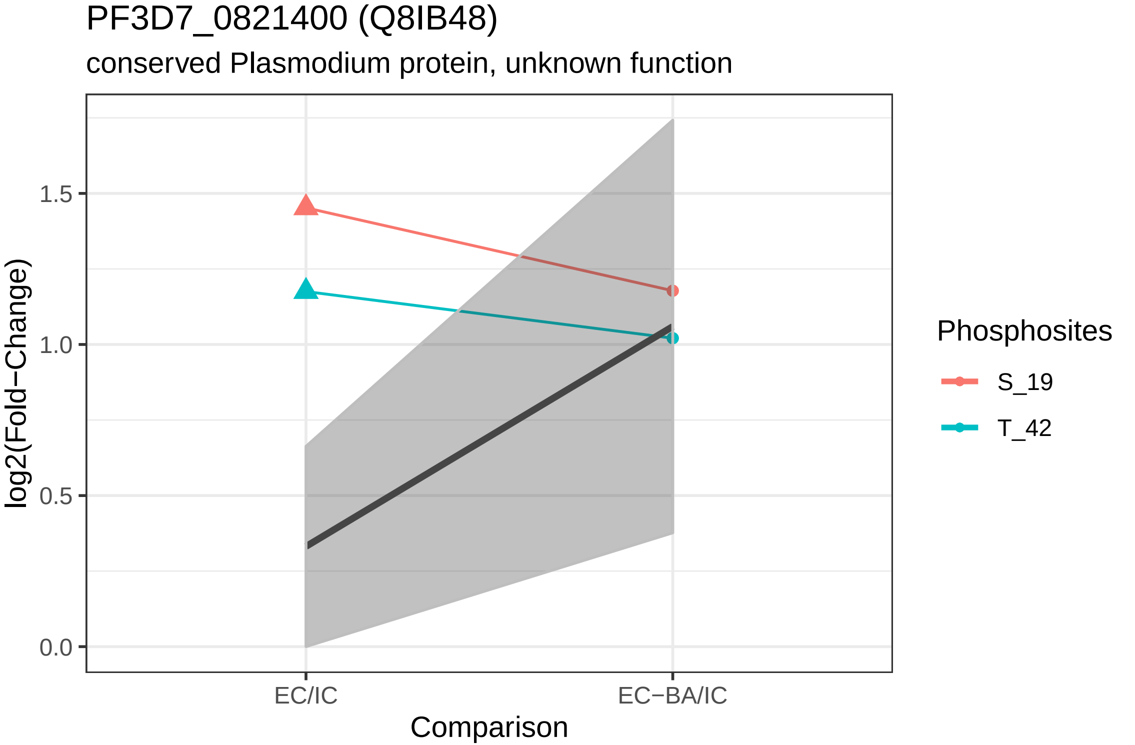
PF3D7_0821400 (Q8IB48)
conserved Plasmodium protein, unknown function
log2(Fold−Change)
0.0
0.5
1.0
1.5
EC/IC
EC−BA/IC
Comparison
Phosphosites
S_19
T_42

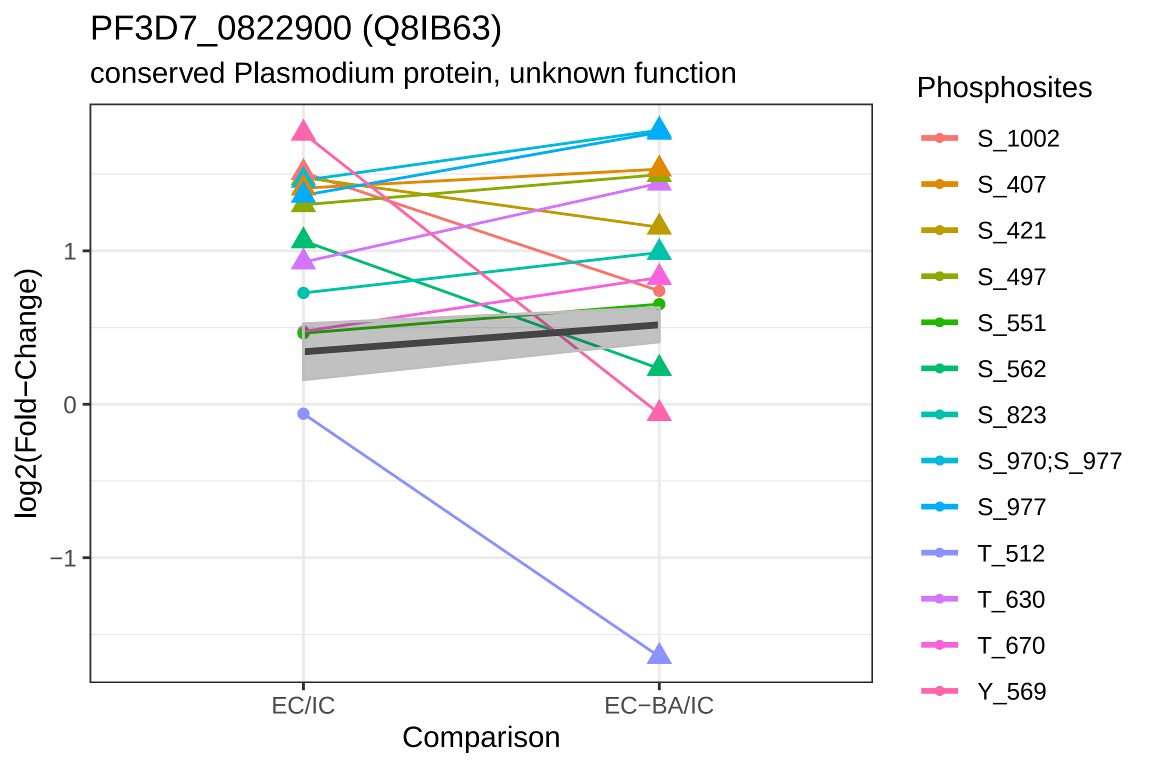
PF3D7_0822900 (Q8IB63)
conserved Plasmodium protein, unknown function
log2(Fold−Change)
1
0
−1
EC/IC
EC−BA/IC
Comparison
Phosphosites
S_1002
S_407
S_421
S_497
S_551
S_562
S_823
S_970;S_977
S_977
T_512
T_630
T_670
Y_569

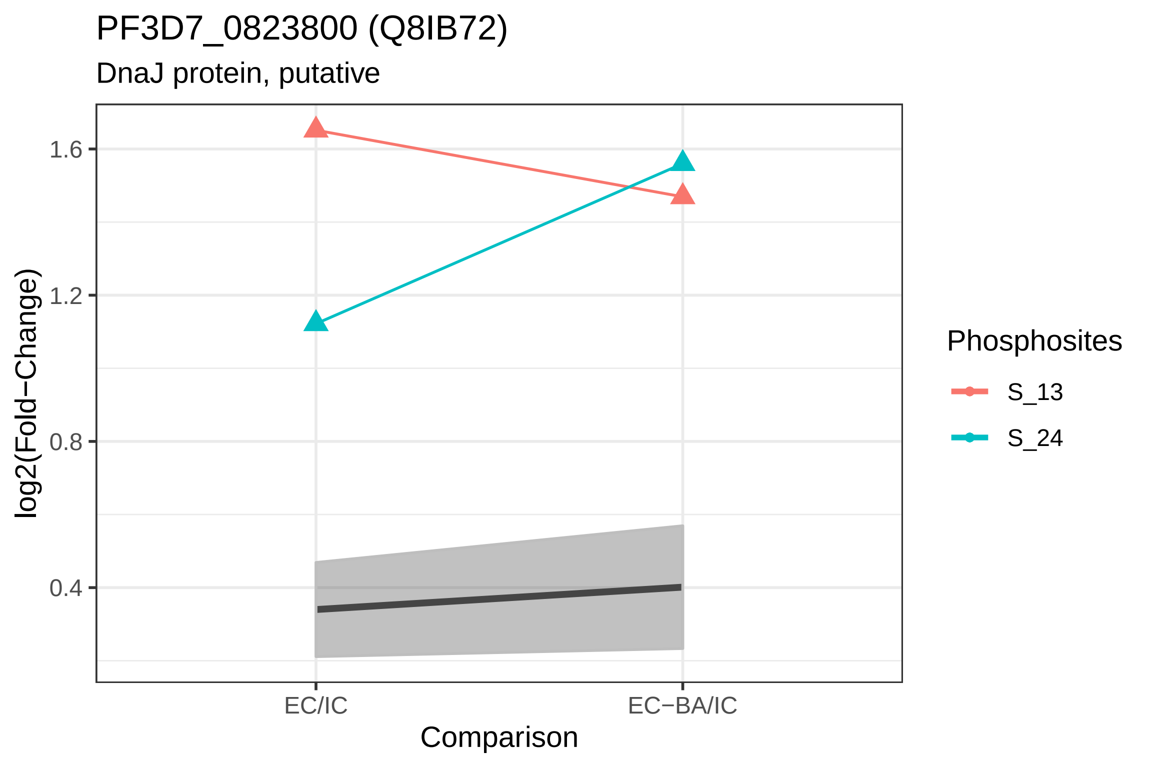

PF3D7_0823800 (Q8IB72)
DnaJ protein, putative
log2(Fold−Change)
1.6
1.2
0.8
0.4
EC/IC
EC−BA/IC
Comparison
Phosphosites
S_13
S_24

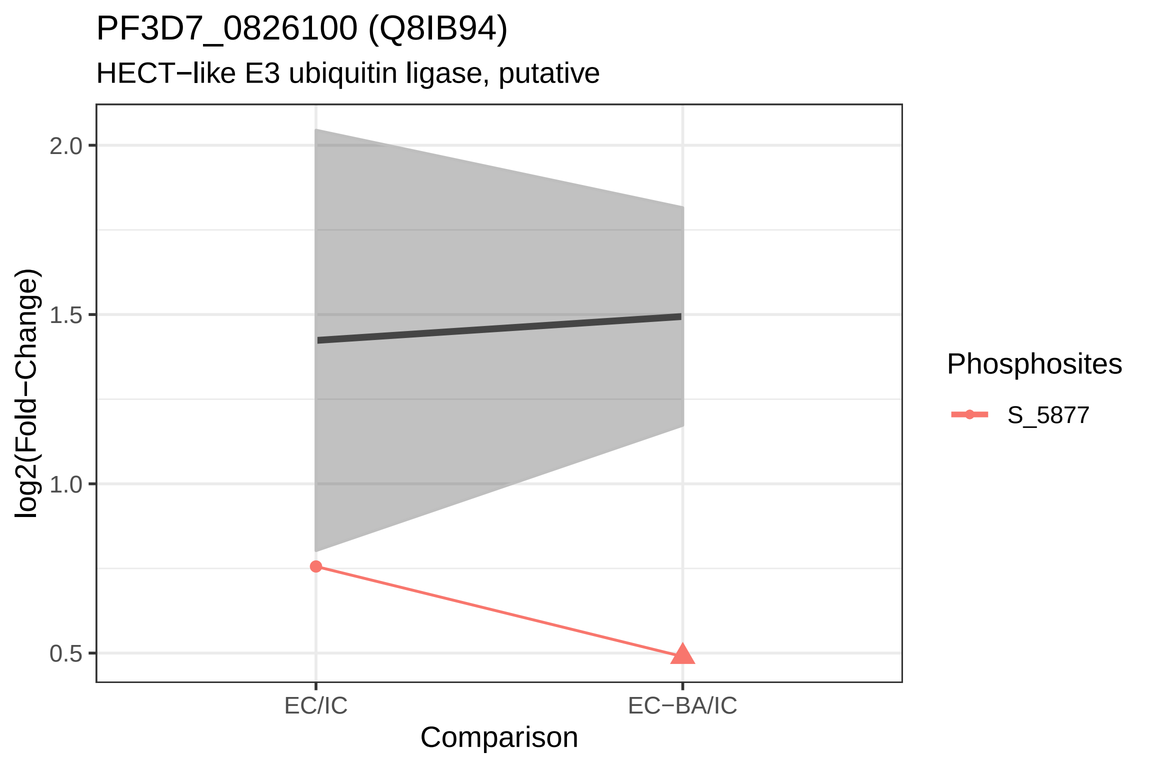

PF3D7_0826100 (Q8IB94)
HECT−like E3 ubiquitin ligase, putative
log2(Fold−Change)
2.0
1.5
1.0
0.5
EC/IC
EC−BA/IC
Comparison
Phosphosites
S_5877

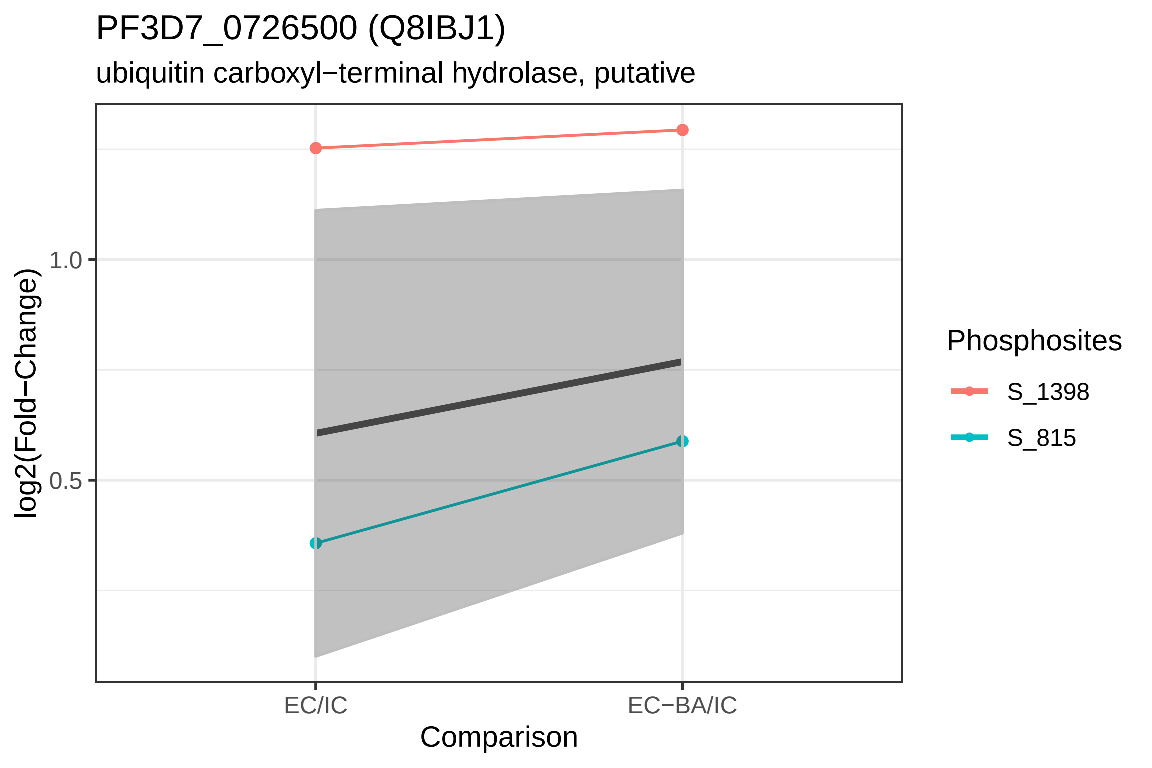

PF3D7_0726500 (Q8IBJ1)
ubiquitin carboxyl−terminal hydrolase, putative
log2(Fold−Change)
1.0
0.5
EC/IC
EC−BA/IC
Comparison
Phosphosites
S_1398
S_815

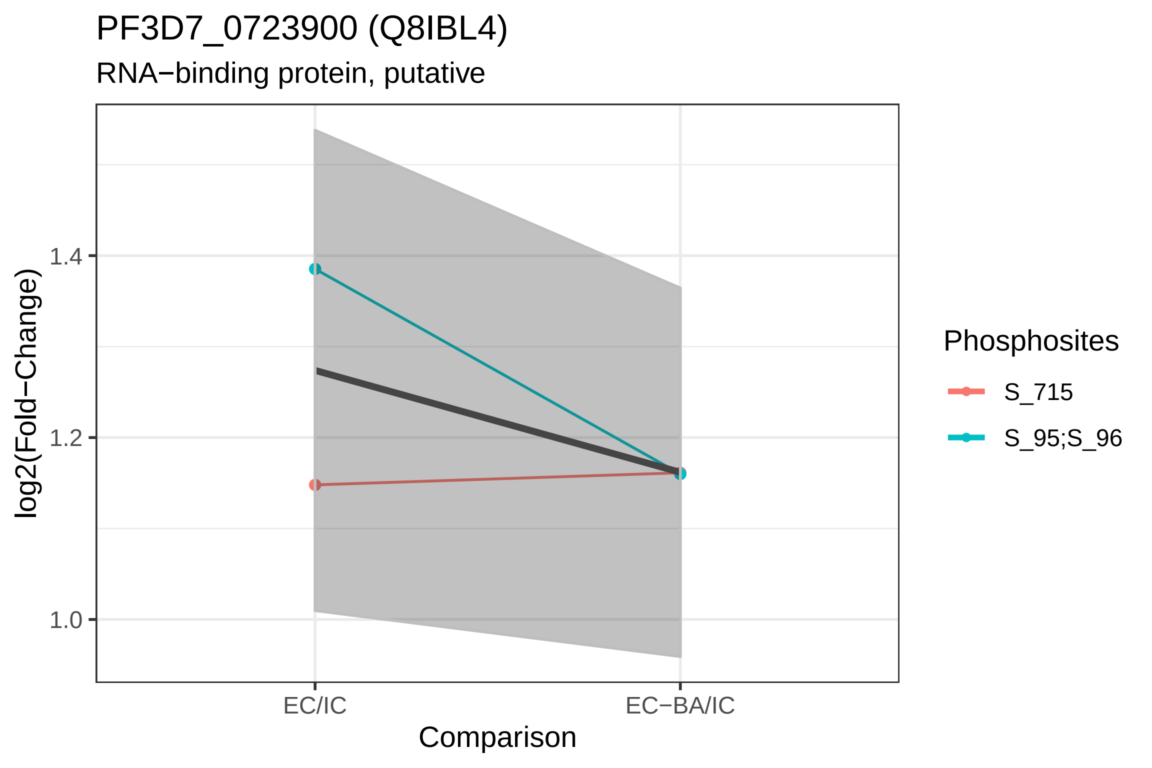

PF3D7_0723900 (Q8IBL4)
RNA−binding protein, putative
log2(Fold−Change)
1.4
1.2
1.0
EC/IC
EC−BA/IC
Comparison
Phosphosites
S_715
S_95;S_96

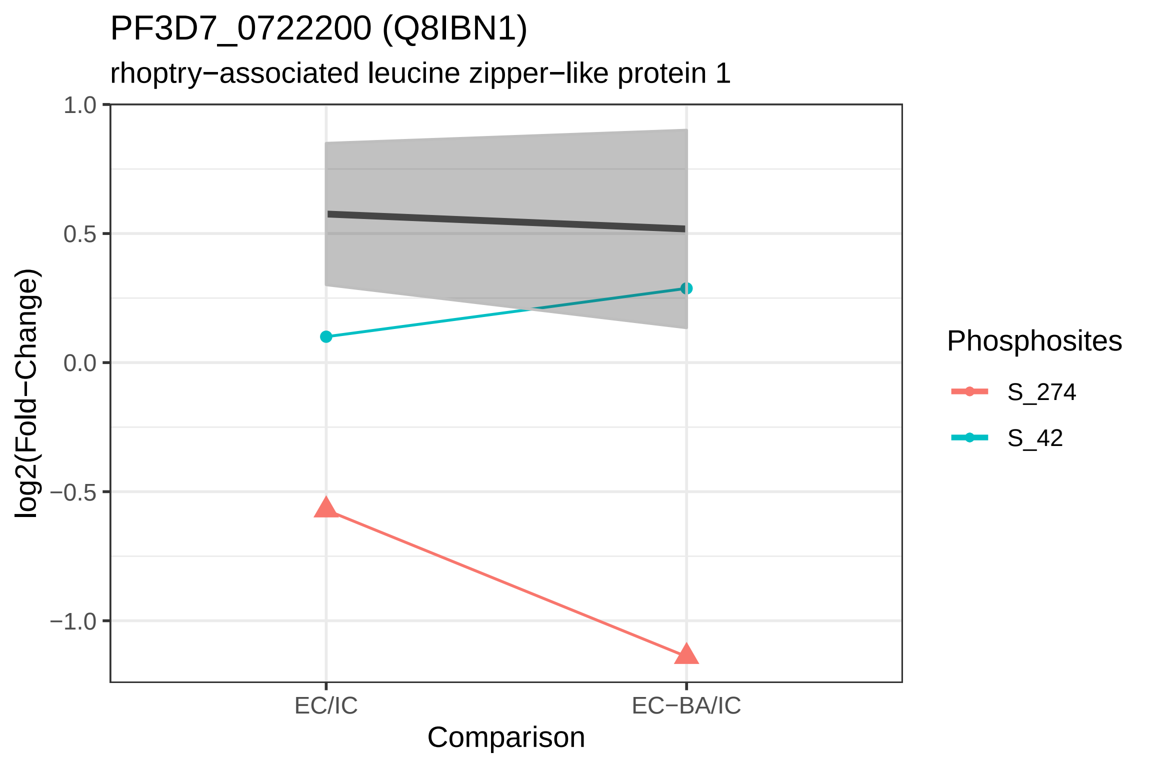
PF3D7_0722200 (Q8IBN1)
rhoptry−associated leucine zipper−like protein 1
1.0
0.5
0.0
−0.5
−1.0
log2(Fold−Change)
EC/IC
EC−BA/IC
Comparison
Phosphosites
S_274
S_42

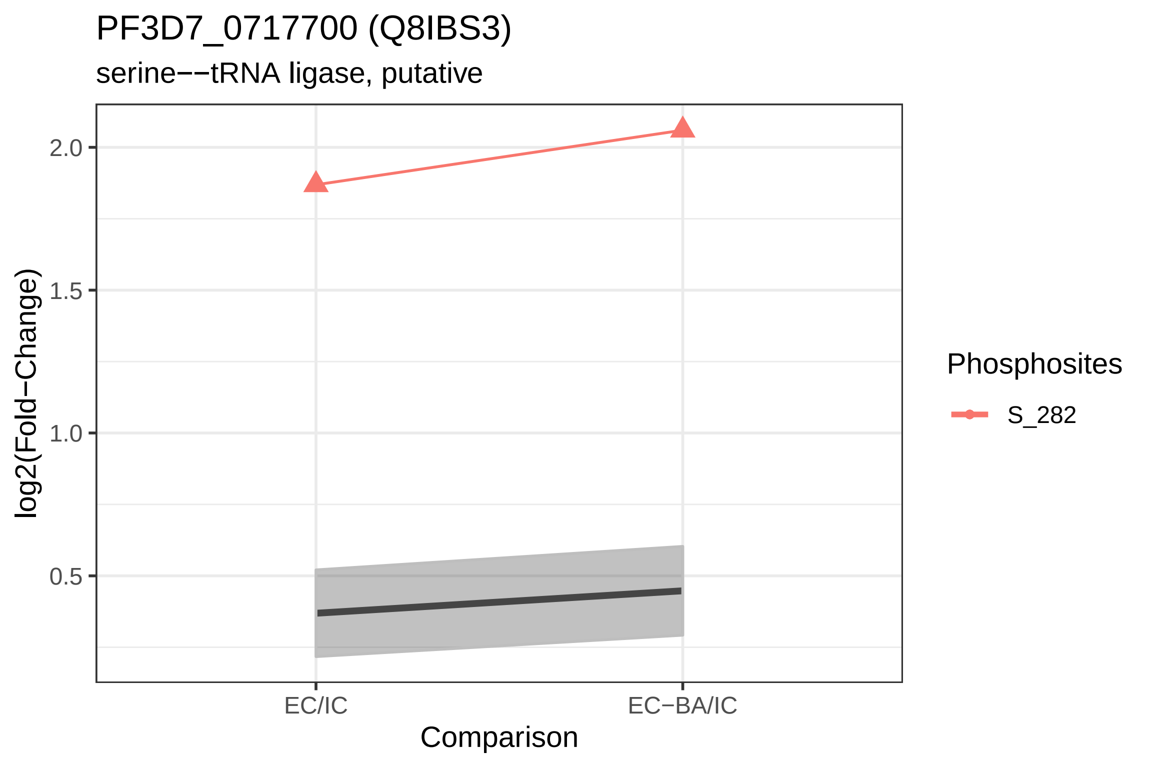
PF3D7_0717700 (Q8IBS3)
serine−−tRNA ligase, putative
log2(Fold−Change)
2.0
1.5
1.0
0.5
EC/IC
EC−BA/IC
Comparison
Phosphosites
S_282

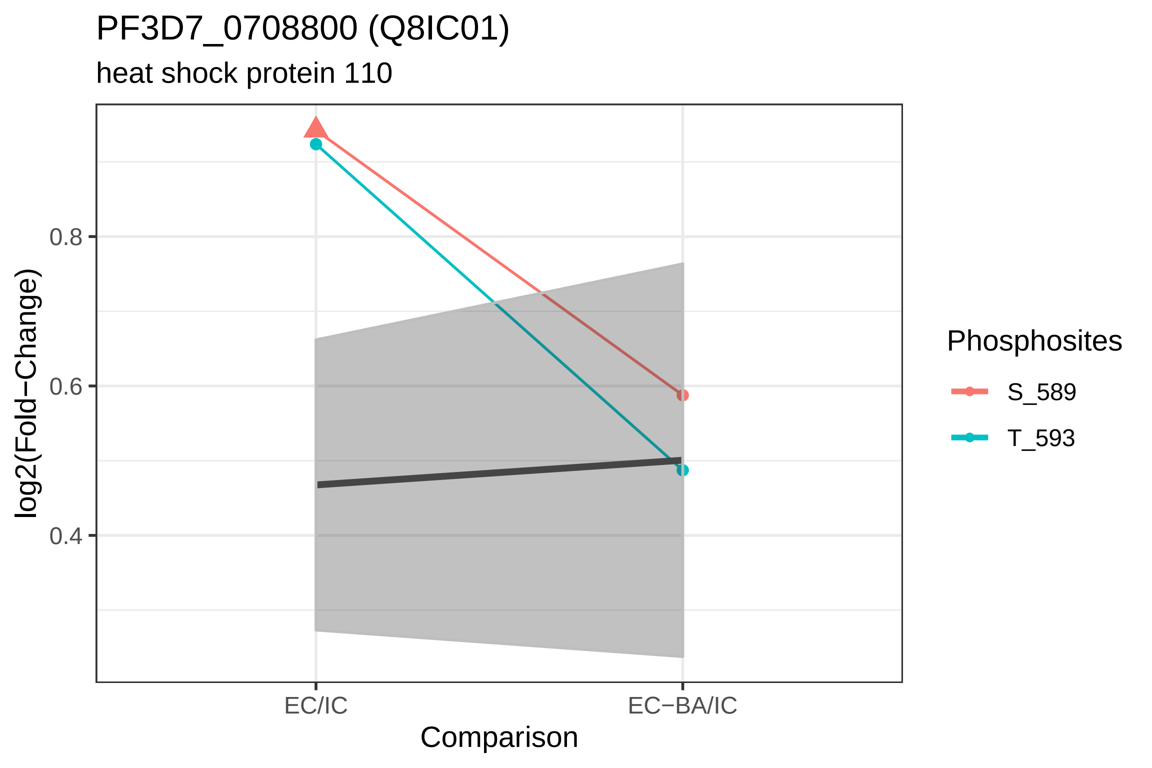
PF3D7_0708800 (Q8IC01)
heat shock protein 110
log2(Fold−Change)
0.8
0.6
0.4
EC/IC
EC−BA/IC
Comparison
Phosphosites
S_589
T_593

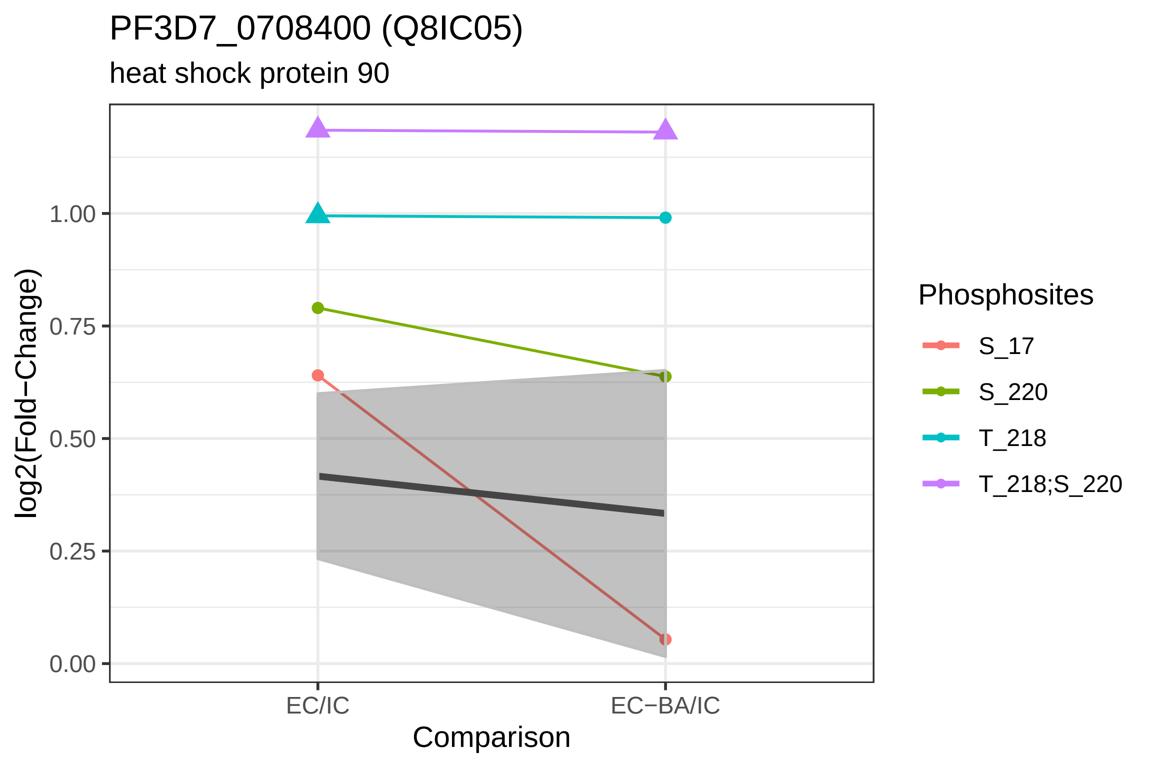

PF3D7_0708400 (Q8IC05)
heat shock protein 90
log2(Fold−Change)
1.00
0.75
0.50
0.25
0.00
EC/IC
EC−BA/IC
Comparison
Phosphosites
S_17
S_220
T_218
T_218;S_220

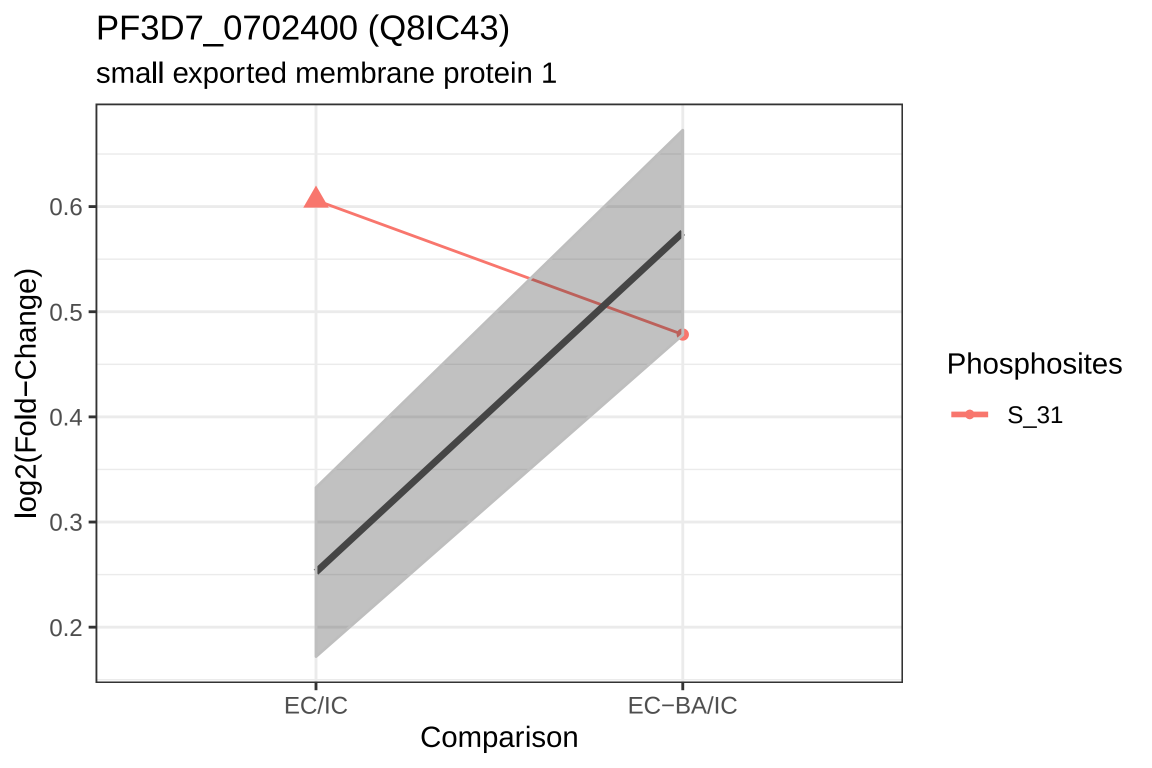

PF3D7_0702400 (Q8IC43)
small exported membrane protein 1
0.6
0.5
0.4
0.3
0.2
log2(Fold−Change)
EC/IC
EC−BA/IC
Comparison
Phosphosites
S_31

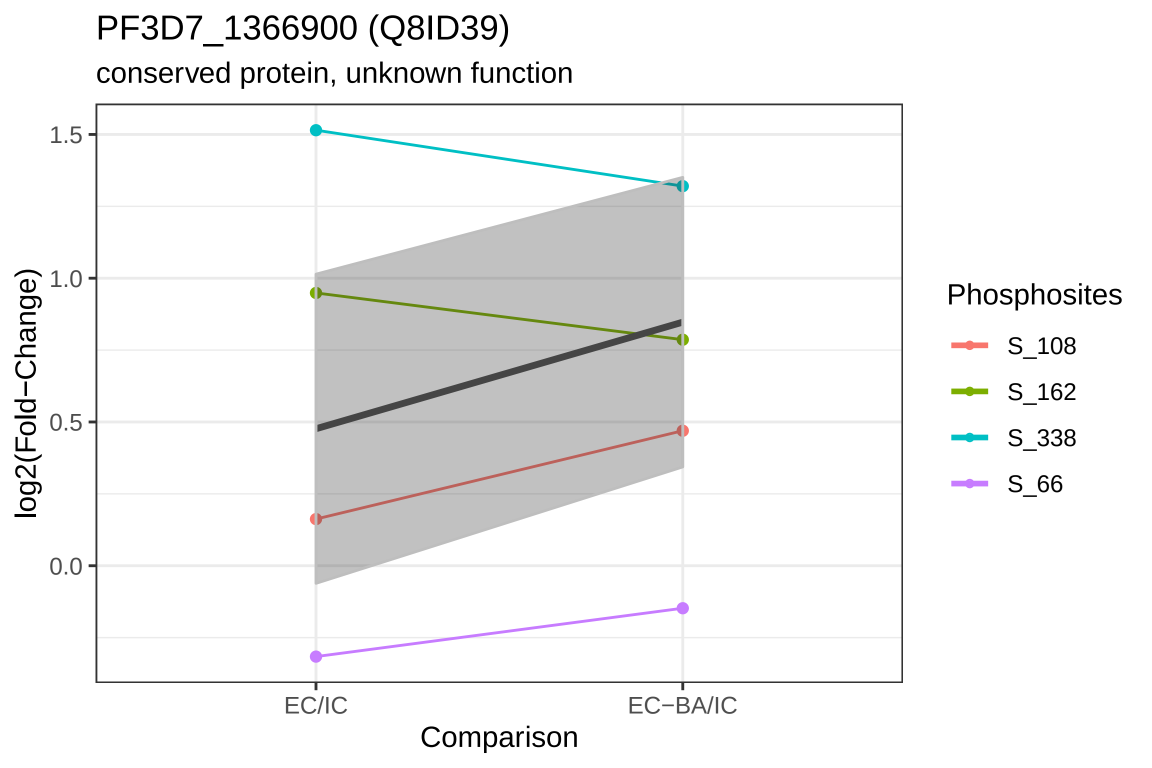

PF3D7_1366900 (Q8ID39)
conserved protein, unknown function
log2(Fold−Change)
1.5
1.0
0.5
0.0
EC/IC
EC−BA/IC
Comparison
Phosphosites
S_108
S_162
S_338
S_66

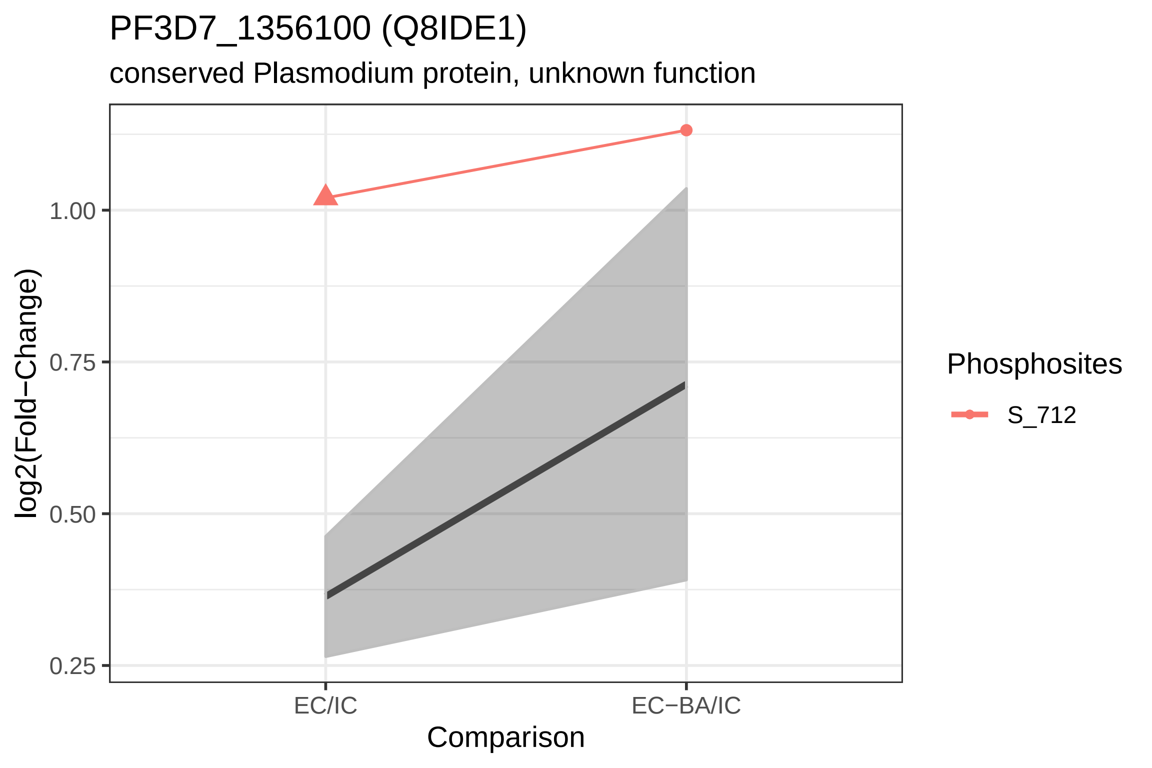

PF3D7_1356100 (Q8IDE1)
conserved Plasmodium protein, unknown function
log2(Fold−Change)
1.00
0.75
0.50
0.25
EC/IC
EC−BA/IC
Comparison
Phosphosites
S_712

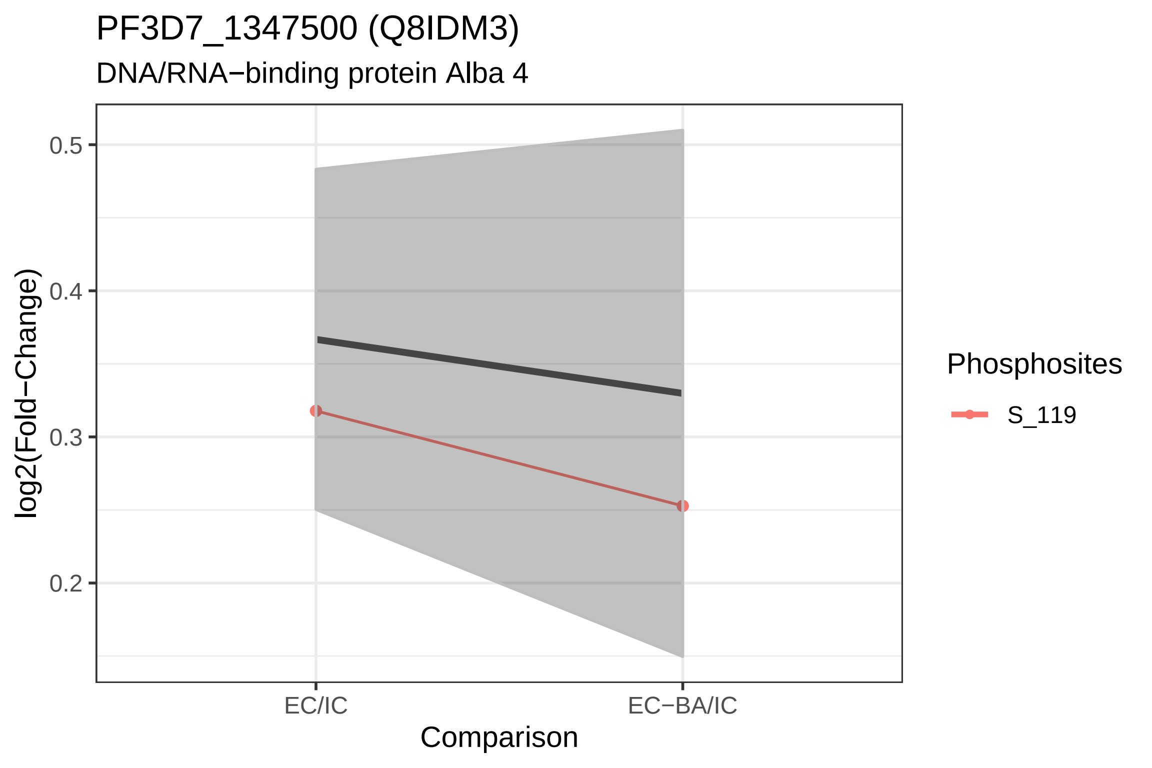
PF3D7_1347500 (Q8IDM3)
DNA/RNA−binding protein Alba 4
log2(Fold−Change)
0.5
0.4
0.3
0.2
EC/IC
EC−BA/IC
Comparison
Phosphosites
S_119

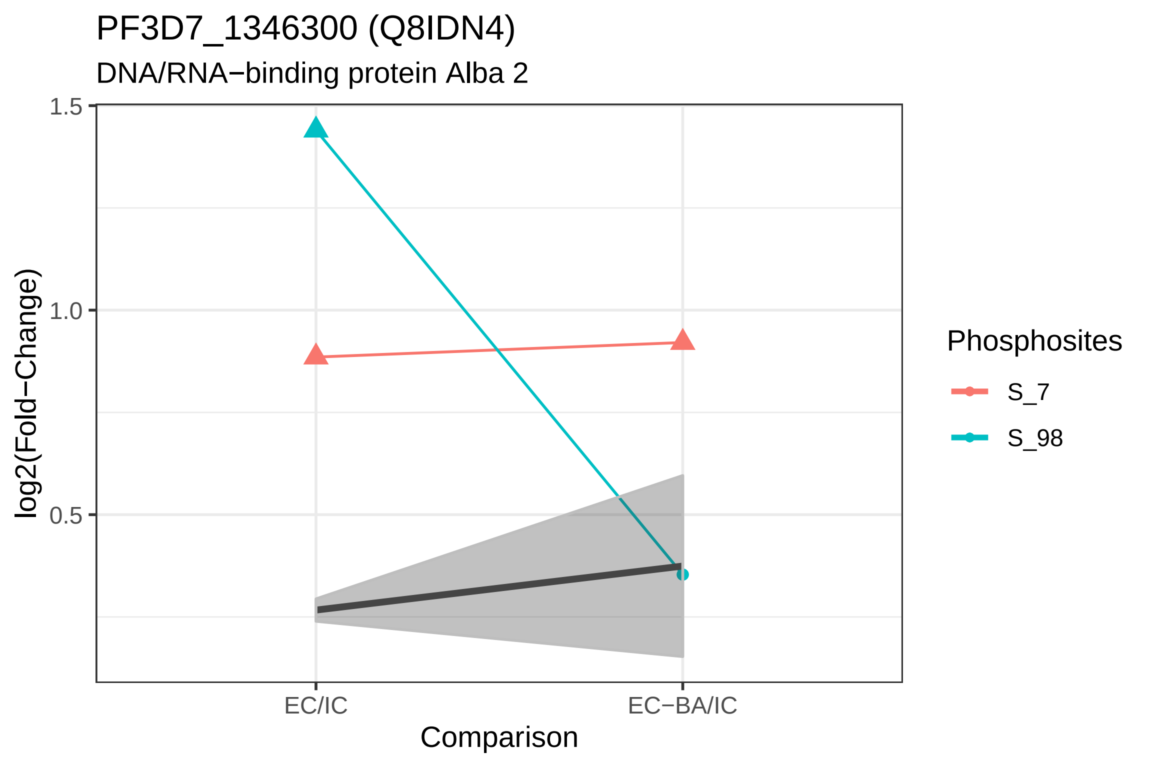
PF3D7_1346300 (Q8IDN4)
DNA/RNA−binding protein Alba 2
log2(Fold−Change)
1.5
1.0
0.5
EC/IC
EC−BA/IC
Comparison
Phosphosites
S_7
S_98

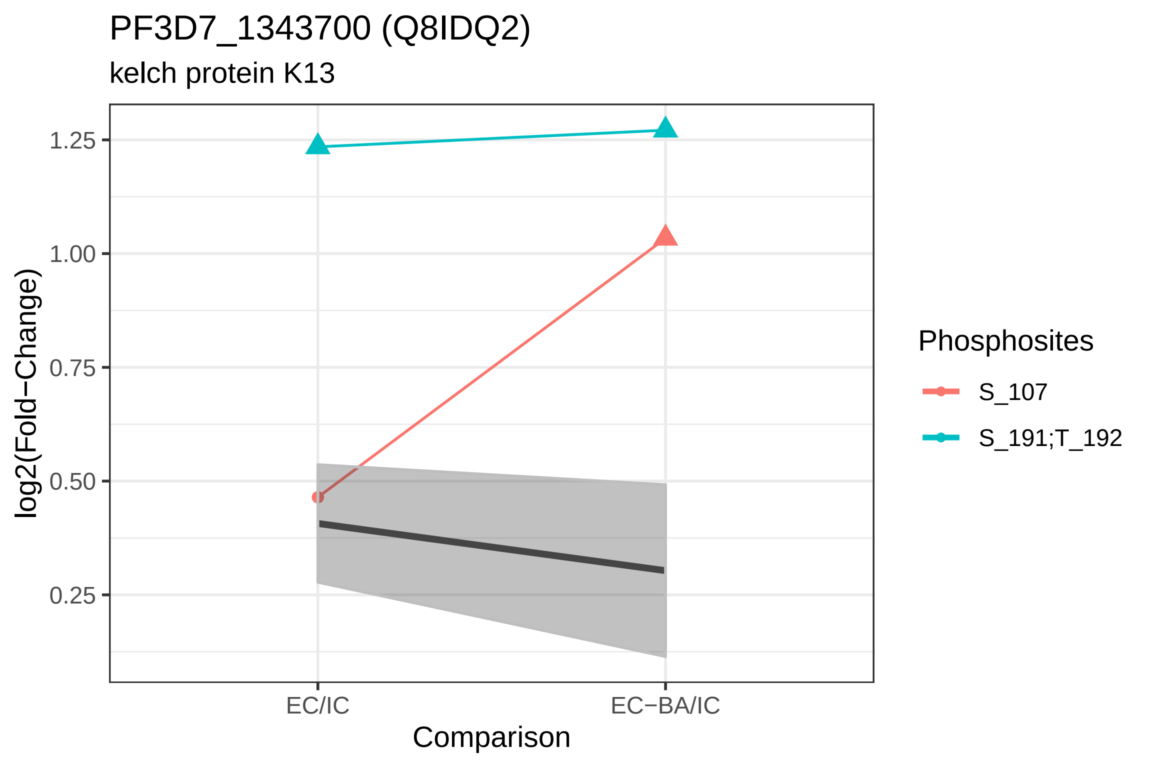

PF3D7_1343700 (Q8IDQ2)
kelch protein K13
log2(Fold−Change)
1.25
1.00
0.75
0.50
0.25
EC/IC
EC−BA/IC
Comparison
Phosphosites
S_107
S_191;T_192

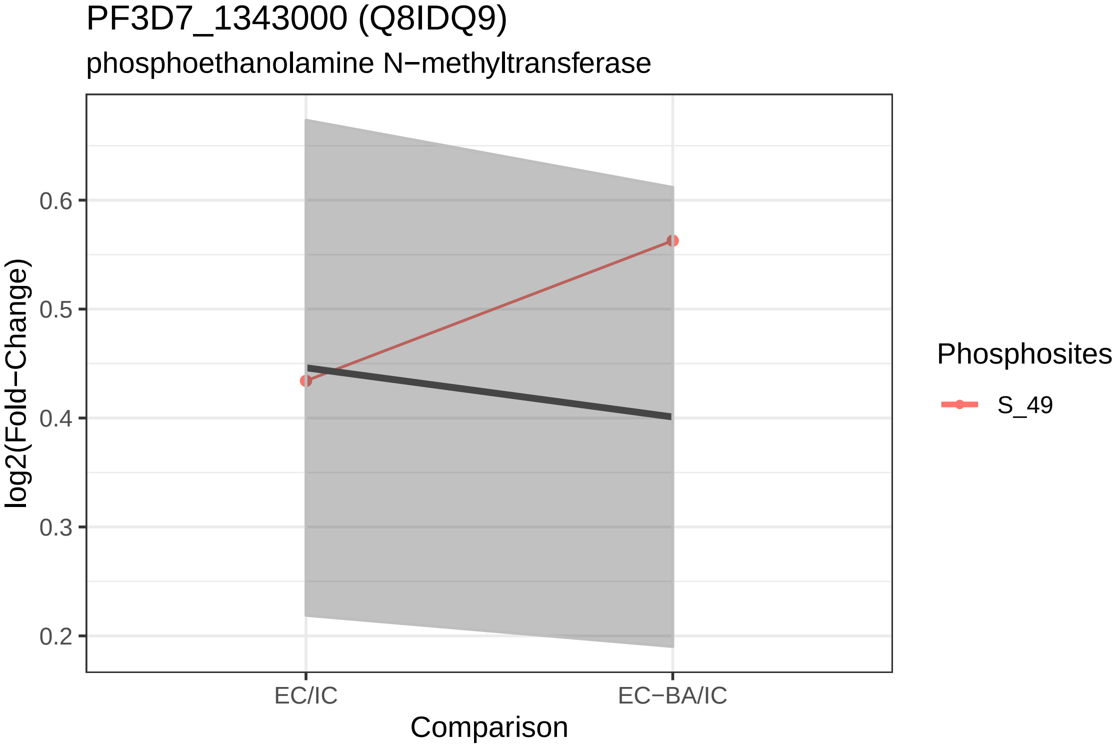
PF3D7_1343000 (Q8IDQ9)
phosphoethanolamine N−methyltransferase
log2(Fold−Change)
0.6
0.5
0.4
0.3
0.2
EC/IC
EC−BA/IC
Comparison
Phosphosites
S_49

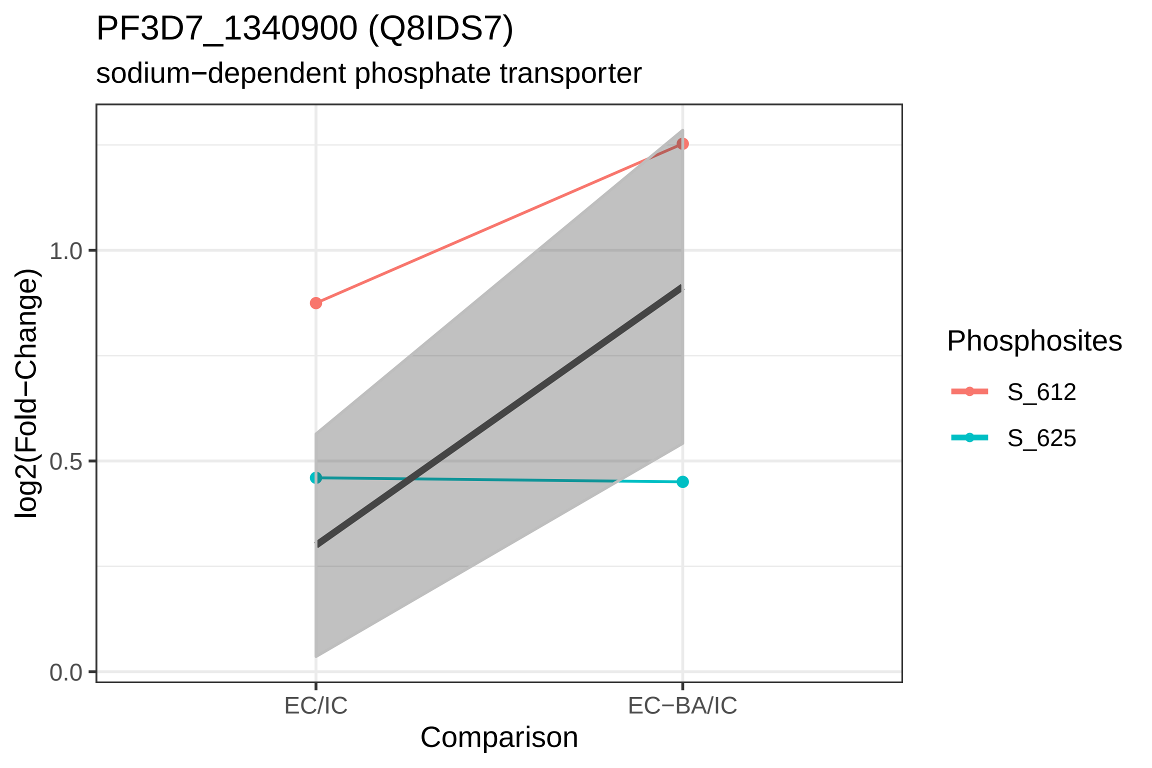

PF3D7_1340900 (Q8IDS7)
sodium−dependent phosphate transporter
log2(Fold−Change)
1.0
0.5
0.0
EC/IC
EC−BA/IC
Comparison
Phosphosites
S_612
S_625

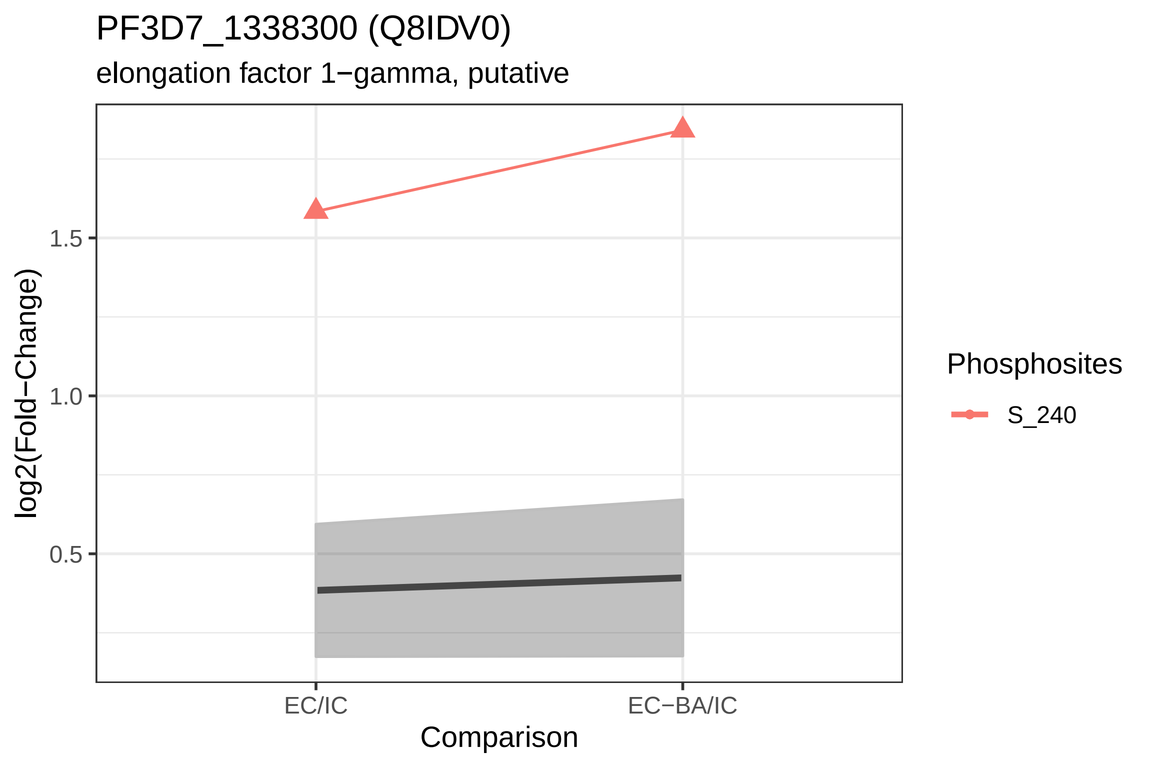
PF3D7_1338300 (Q8IDV0)
elongation factor 1−gamma, putative
log2(Fold−Change)
1.5
1.0
0.5
EC/IC
EC−BA/IC
Comparison
Phosphosites
S_240

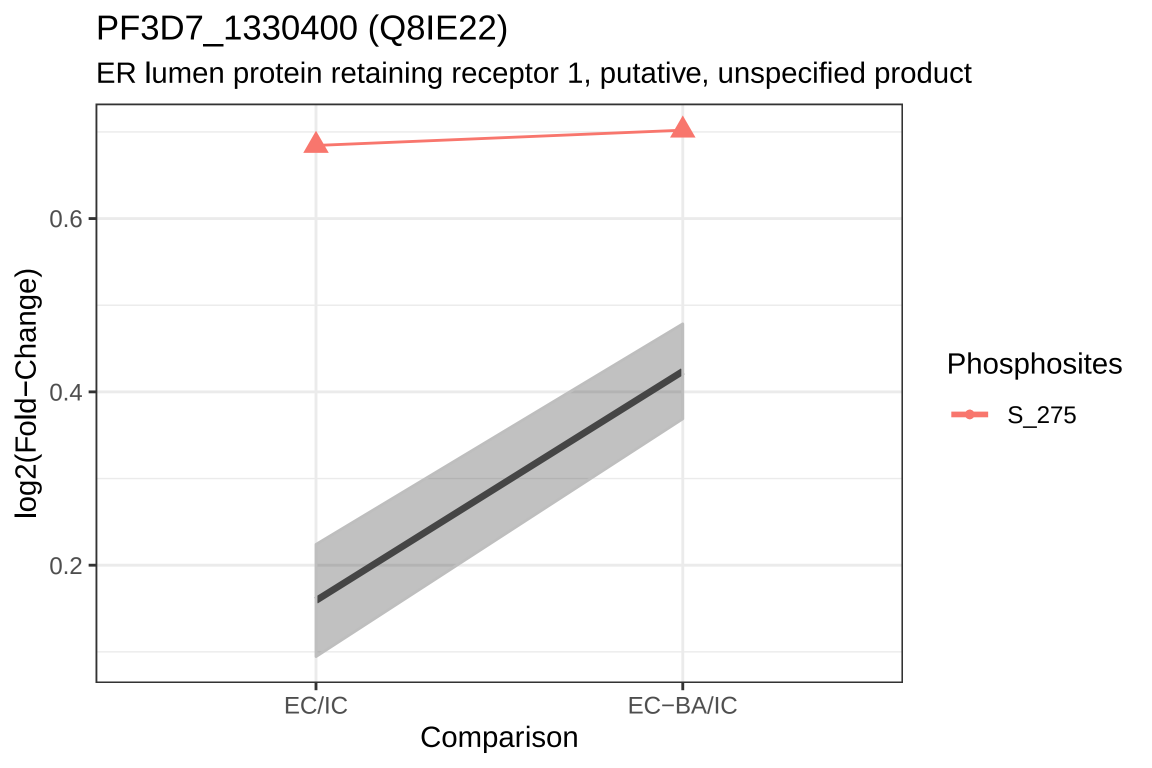
PF3D7_1330400 (Q8IE22)
ER lumen protein retaining receptor 1, putative, unspecified product
log2(Fold−Change)
0.6
0.4
0.2
EC/IC
EC−BA/IC
Comparison
Phosphosites
S_275

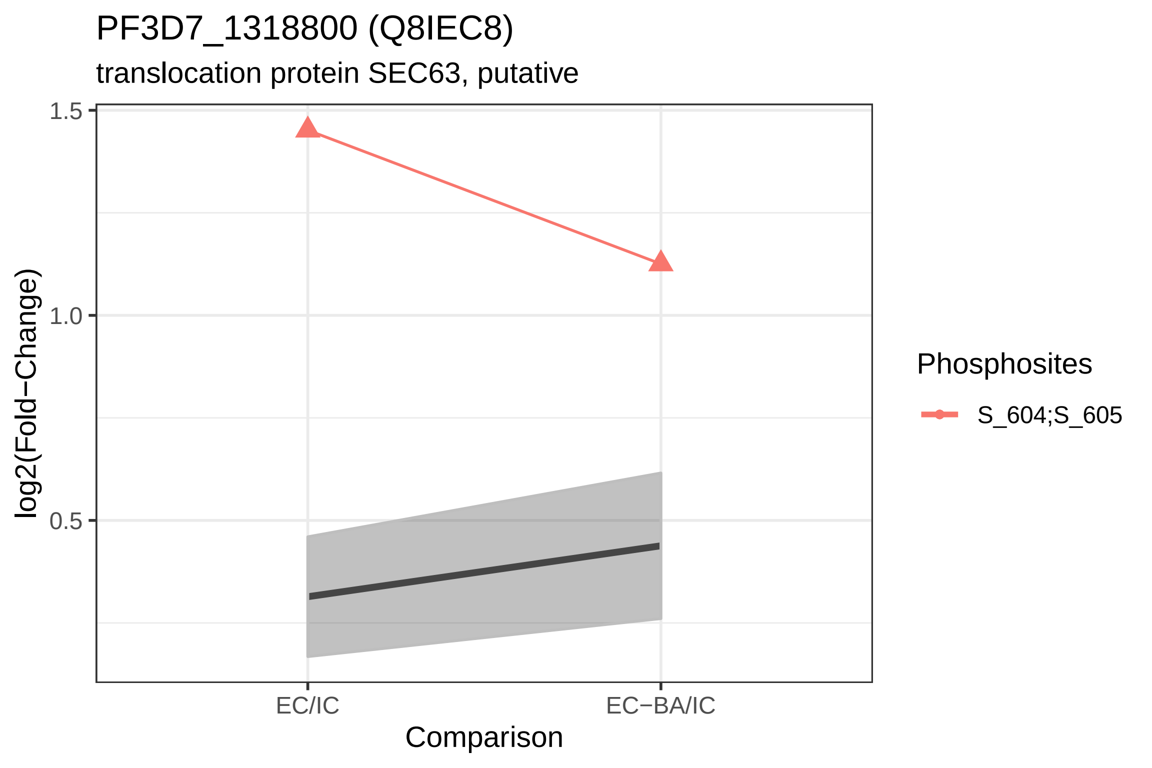

PF3D7_1318800 (Q8IEC8)
translocation protein SEC63, putative
1.5
1.0
0.5
log2(Fold−Change)
EC/IC
EC−BA/IC
Comparison
Phosphosites
S_604;S_605

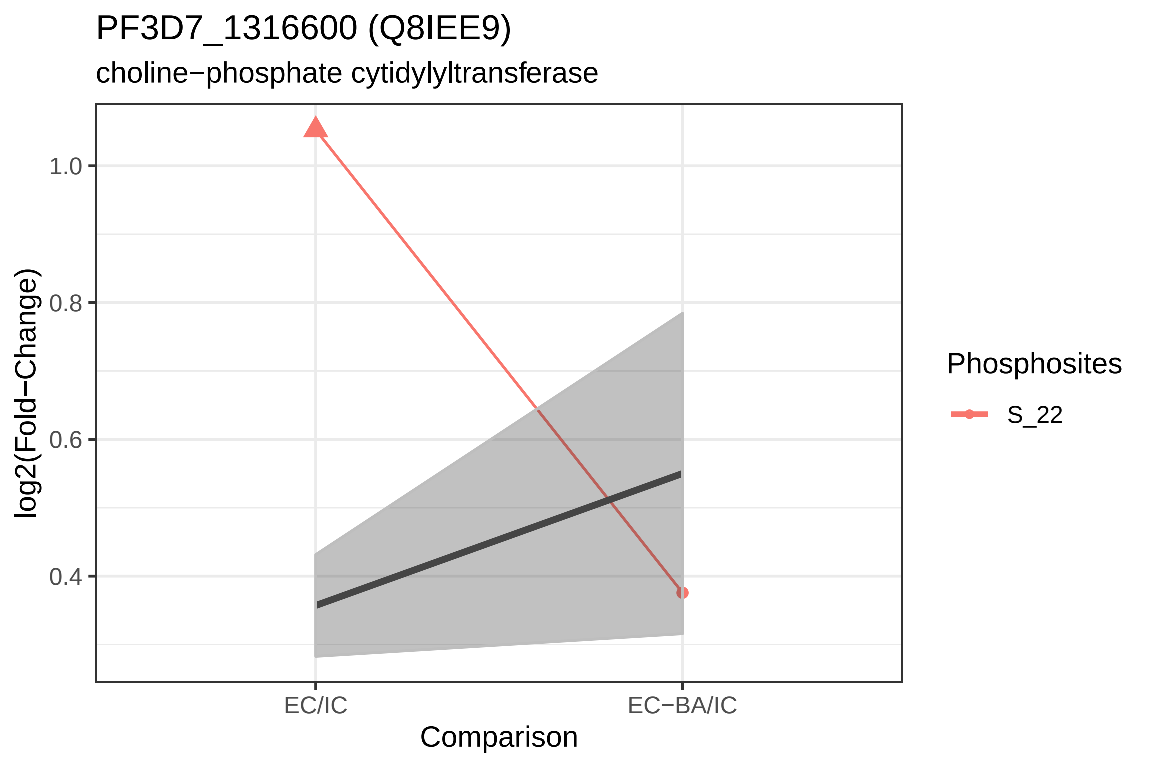

PF3D7_1316600 (Q8IEE9)
choline−phosphate cytidylyltransferase
log2(Fold−Change)
1.0
0.8
0.6
0.4
EC/IC
EC−BA/IC
Comparison
Phosphosites
S_22

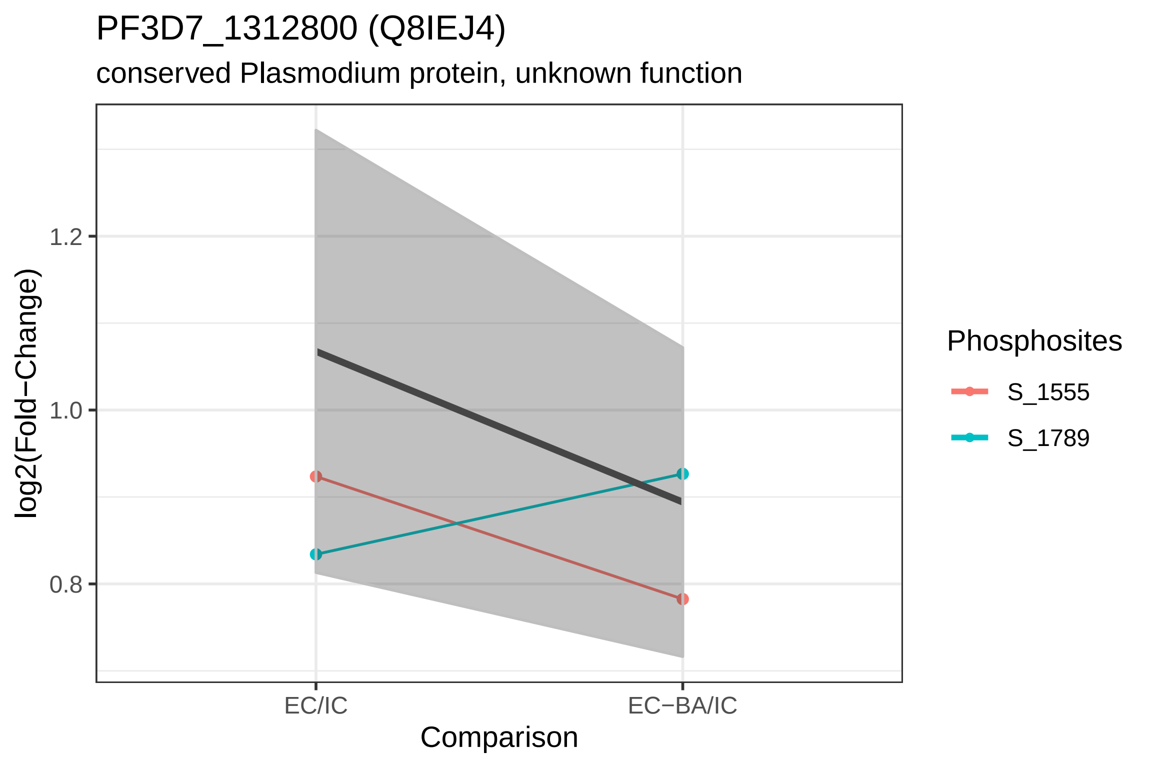
PF3D7_1312800 (Q8IEJ4)
conserved Plasmodium protein, unknown function
log2(Fold−Change)
1.2
1.0
0.8
EC/IC
EC−BA/IC
Comparison
Phosphosites
S_1555
S_1789

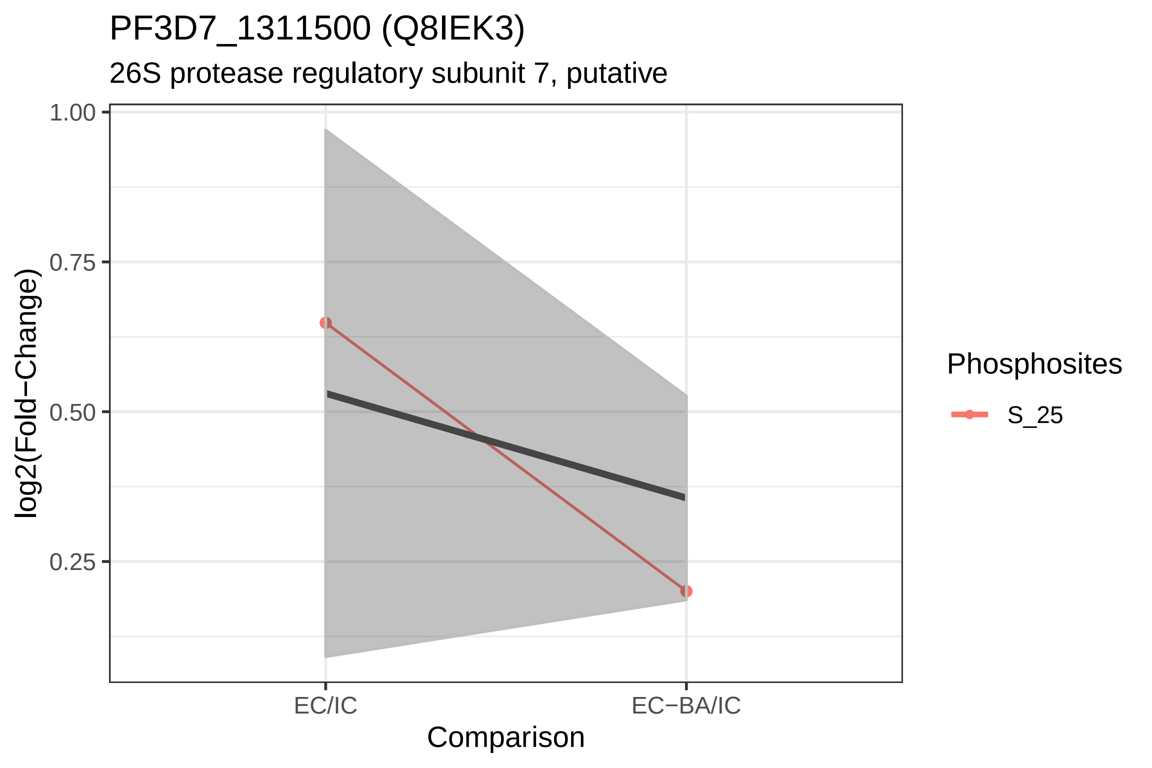
PF3D7_1311500 (Q8IEK3)
26S protease regulatory subunit 7, putative
log2(Fold−Change)
1.00
0.75
0.50
0.25
EC/IC
EC−BA/IC
Comparison
Phosphosites
S_25

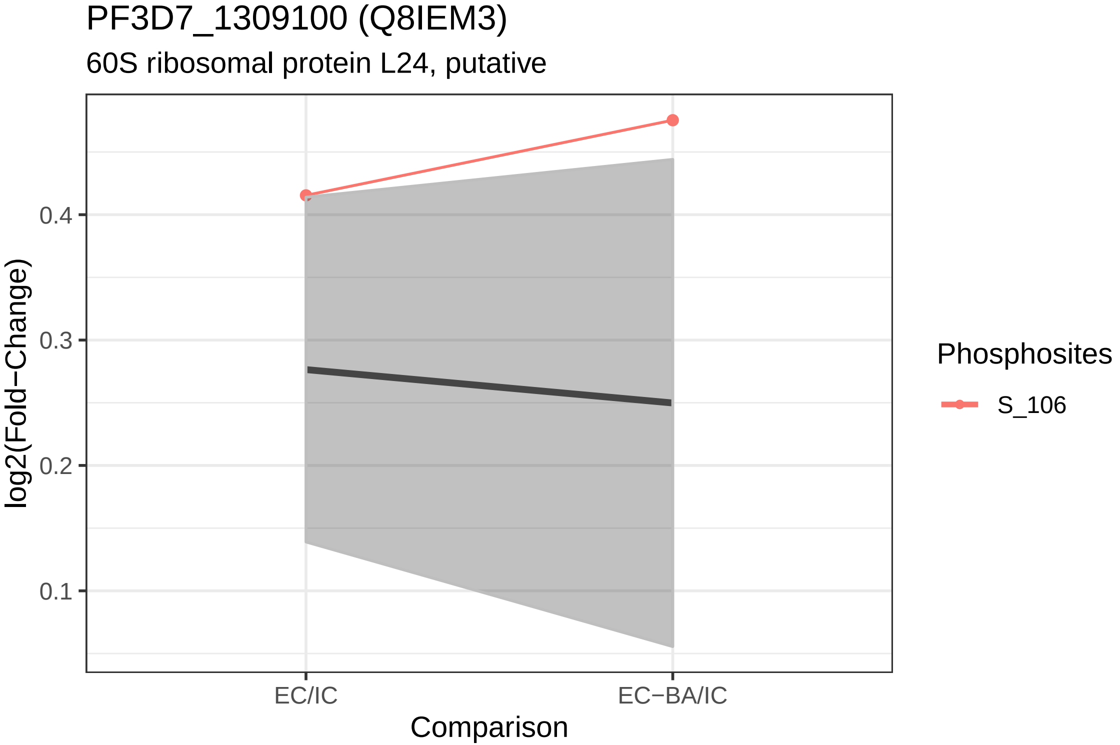

PF3D7_1309100 (Q8IEM3)
60S ribosomal protein L24, putative
log2(Fold−Change)
0.4
0.3
0.2
0.1
EC/IC
EC−BA/IC
Comparison
Phosphosites
S_106

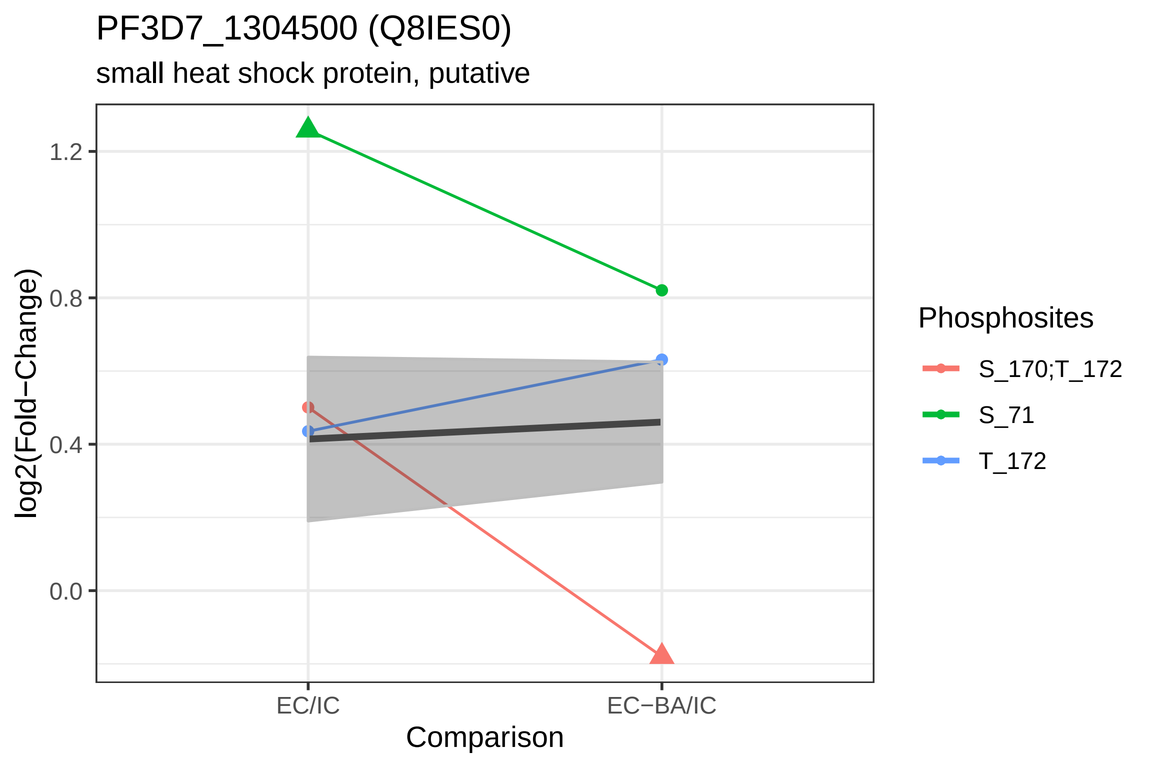

PF3D7_1304500 (Q8IES0)
small heat shock protein, putative
log2(Fold−Change)
1.2
0.8
0.4
0.0
EC/IC
EC−BA/IC
Comparison
Phosphosites
S_170;T_172
S_71
T_172

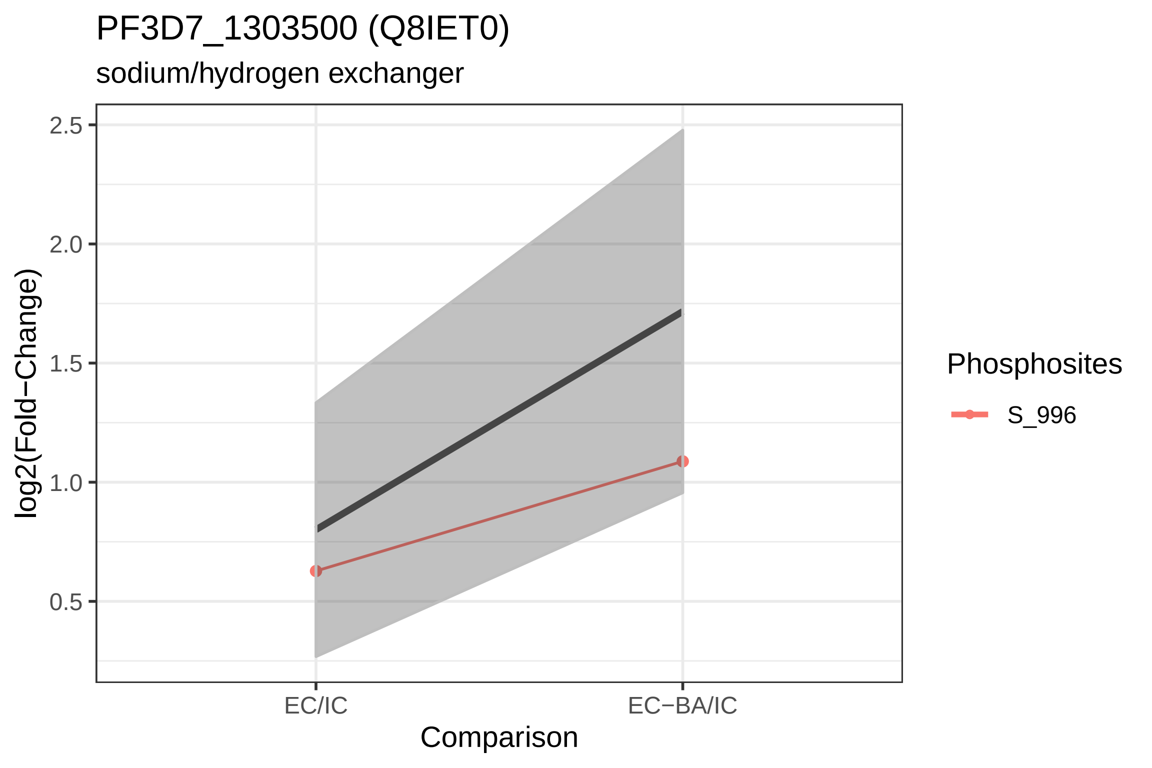

PF3D7_1303500 (Q8IET0)
sodium/hydrogen exchanger
log2(Fold−Change)
2.5
2.0
1.5
1.0
0.5
EC/IC
EC−BA/IC
Comparison
Phosphosites
S_996

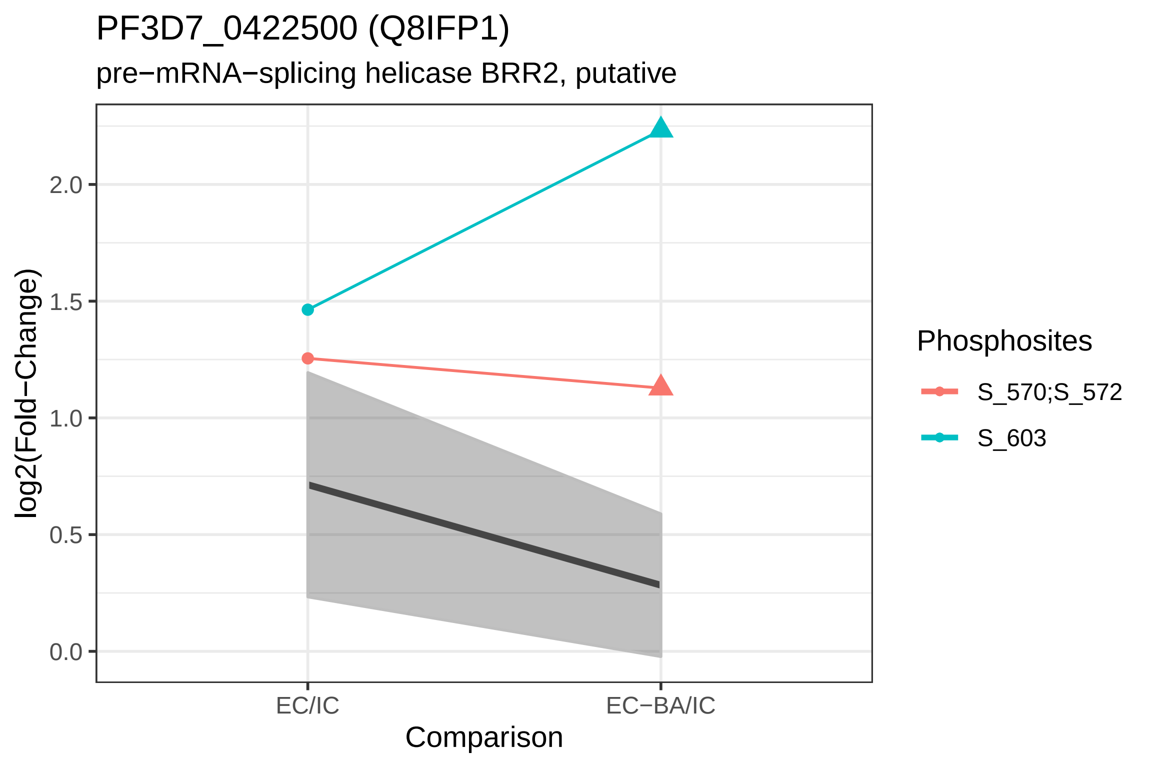
PF3D7_0422500 (Q8IFP1)
pre−mRNA−splicing helicase BRR2, putative
log2(Fold−Change)
2.0
1.5
1.0
0.5
0.0
EC/IC
EC−BA/IC
Comparison
Phosphosites
S_570;S_572
S_603

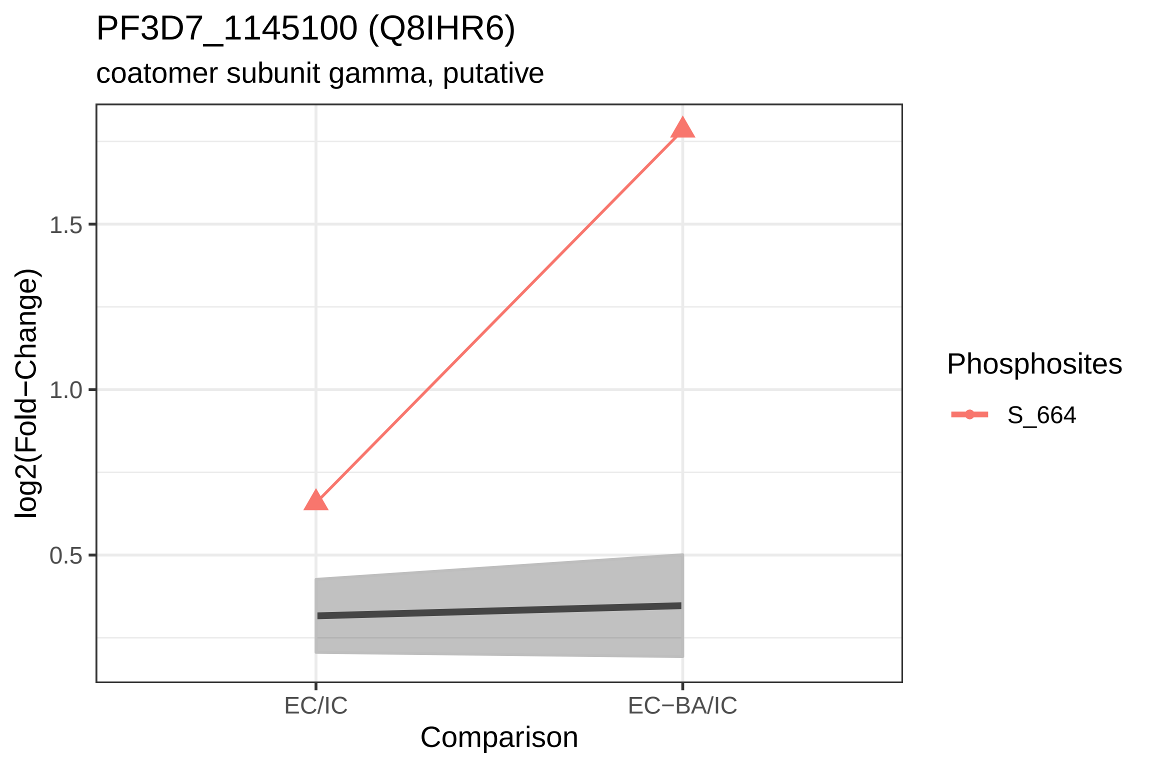
PF3D7_1145100 (Q8IHR6)
coatomer subunit gamma, putative
log2(Fold−Change)
1.5
1.0
0.5
EC/IC
EC−BA/IC
Comparison
Phosphosites
S_664

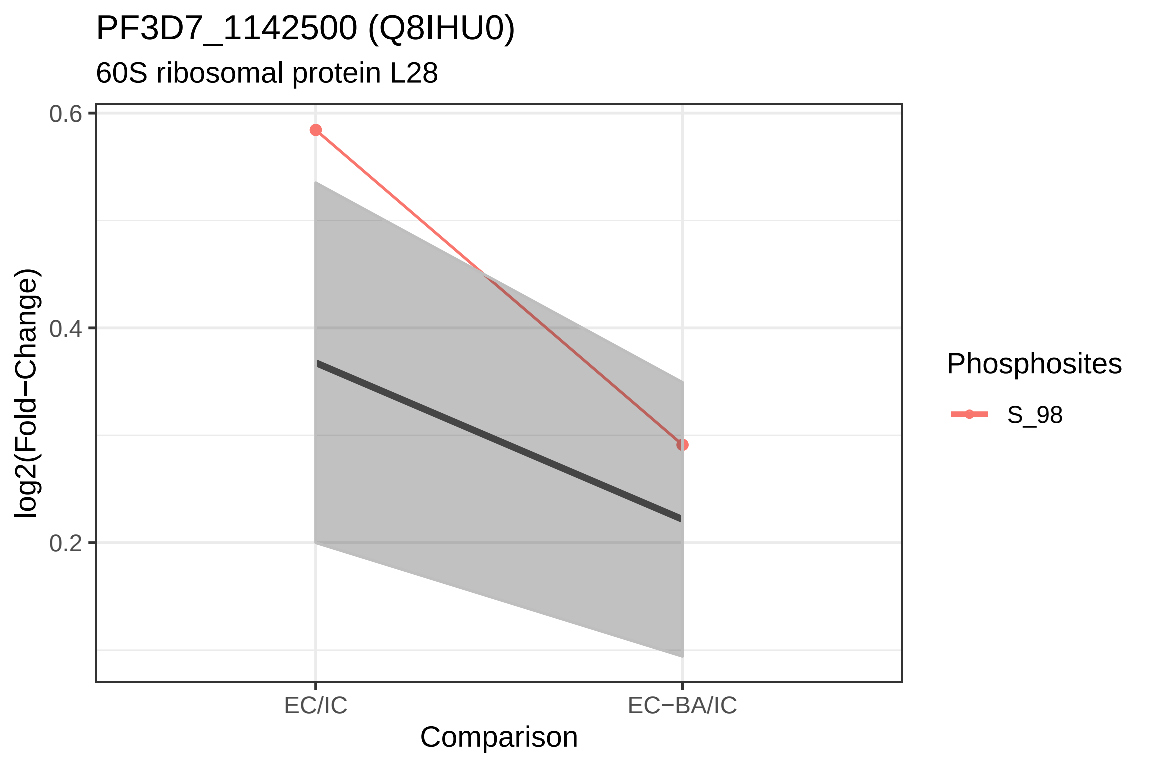
PF3D7_1142500 (Q8IHU0)
60S ribosomal protein L28
log2(Fold−Change)
0.6
0.4
0.2
EC/IC
EC−BA/IC
Comparison
Phosphosites
S_98

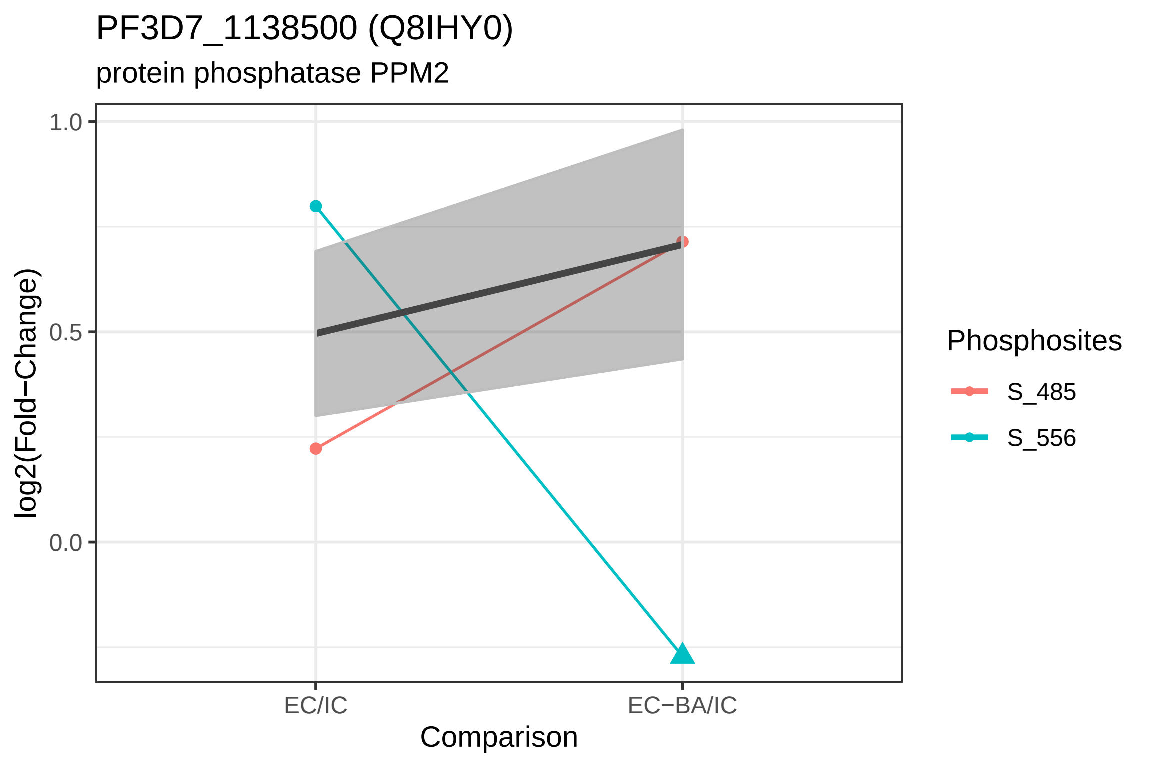
PF3D7_1138500 (Q8IHY0)
protein phosphatase PPM2
1.0
0.5
0.0
log2(Fold−Change)
EC/IC
EC−BA/IC
Comparison
Phosphosites
S_485
S_556

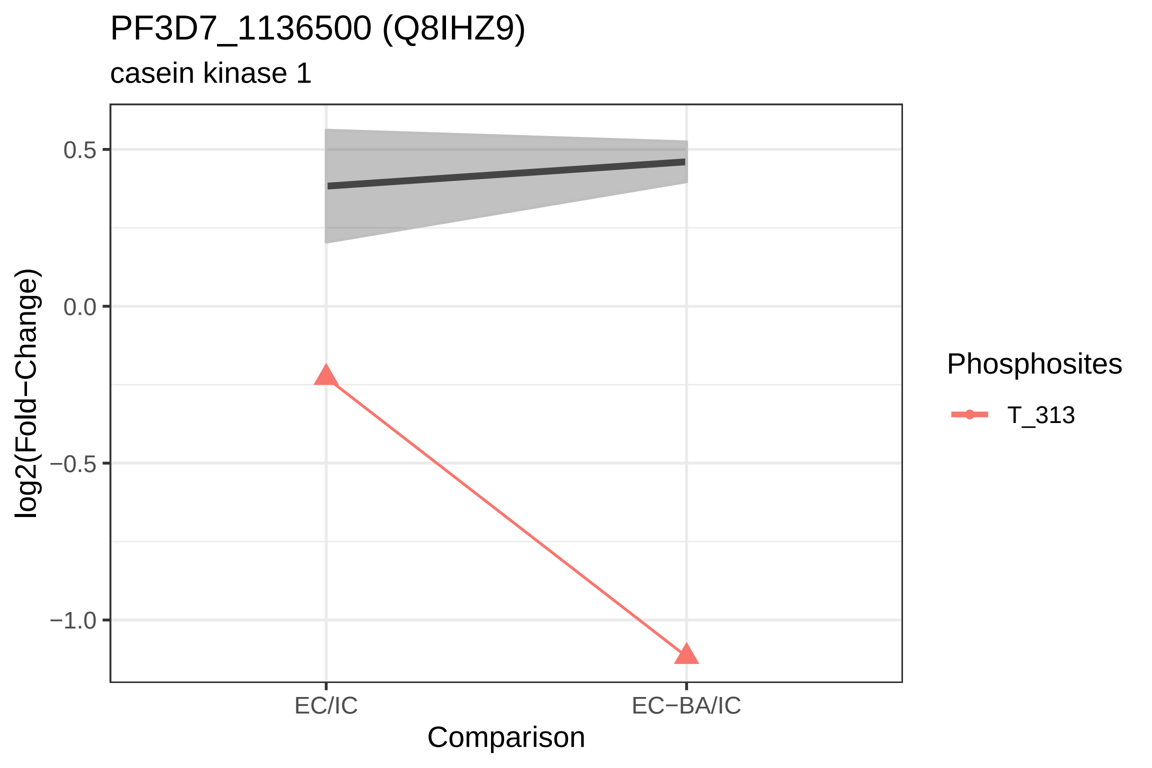

PF3D7_1136500 (Q8IHZ9)
casein kinase 1
0.5
0.0
−0.5
−1.0
log2(Fold−Change)
EC/IC
EC−BA/IC
Comparison
Phosphosites
T_313

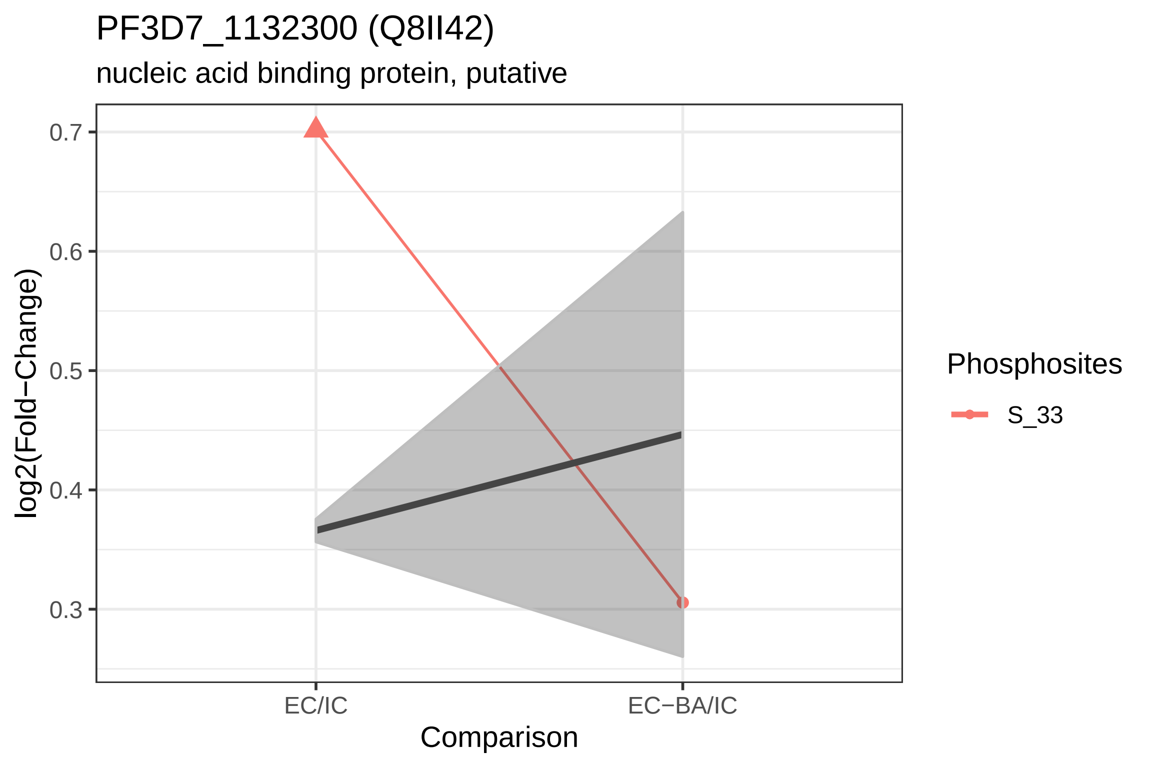
PF3D7_1132300 (Q8II42)
nucleic acid binding protein, putative
log2(Fold−Change)
0.7
0.6
0.5
0.4
0.3
EC/IC
EC−BA/IC
Comparison
Phosphosites
S_33

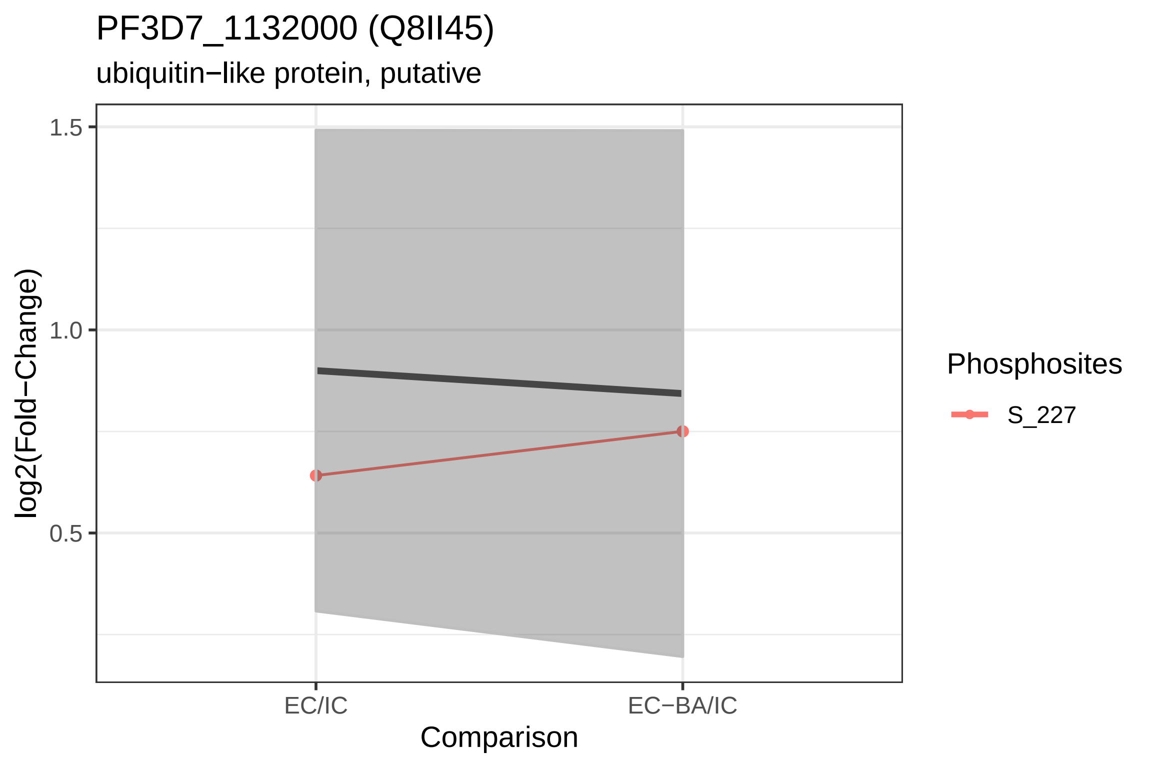

PF3D7_1132000 (Q8II45)
ubiquitin−like protein, putative
log2(Fold−Change)
1.5
1.0
0.5
EC/IC
EC−BA/IC
Comparison
Phosphosites
S_227

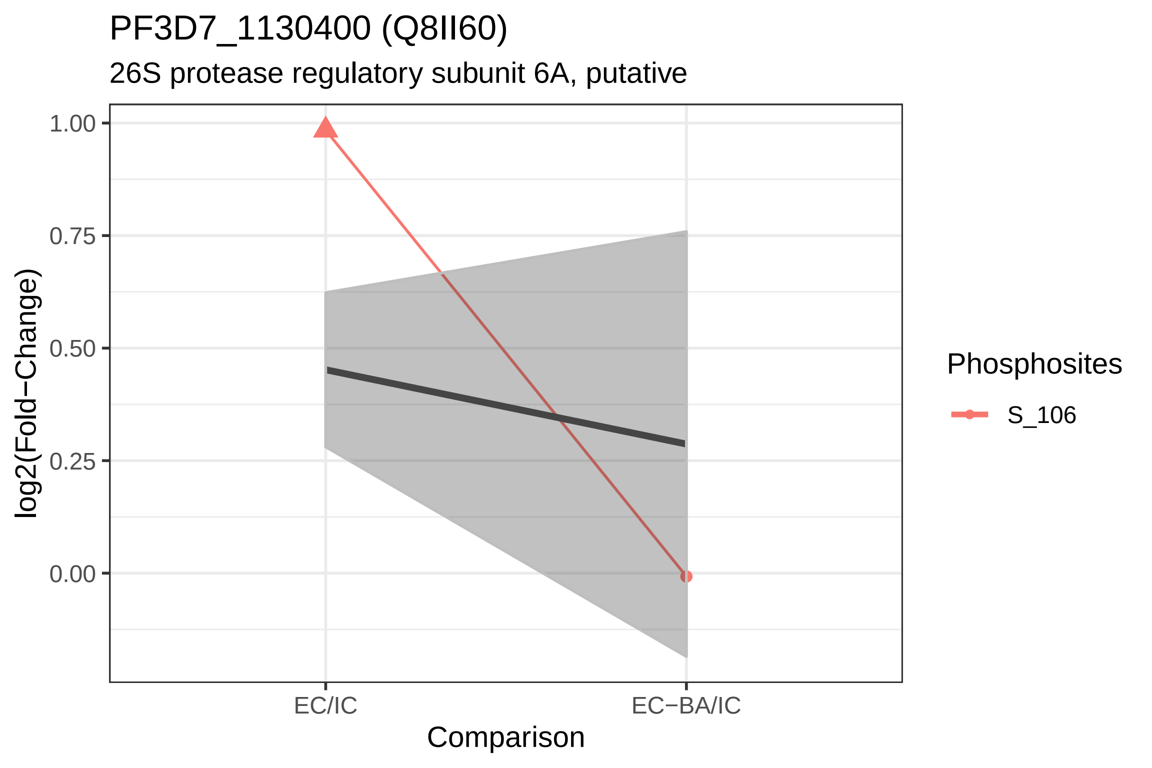
PF3D7_1130400 (Q8II60)
26S protease regulatory subunit 6A, putative
1.00
0.75
0.50
0.25
0.00
log2(Fold−Change)
EC/IC
EC−BA/IC
Comparison
Phosphosites
S_106

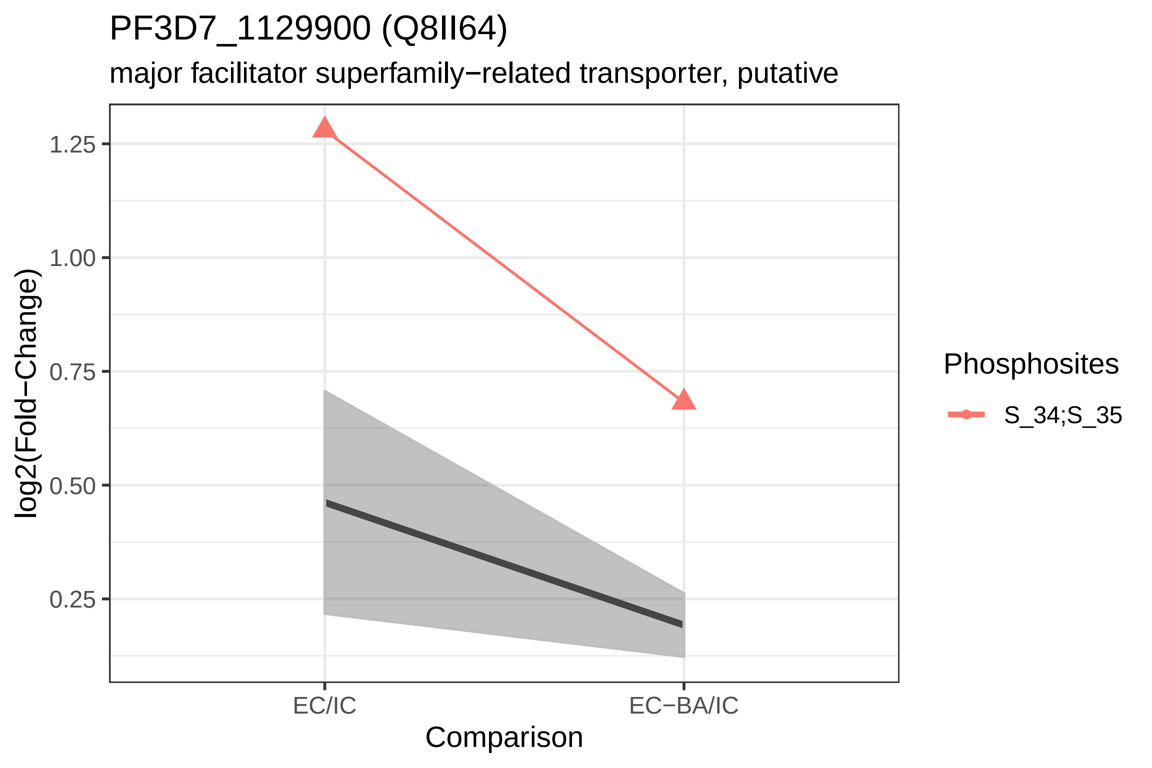
PF3D7_1129900 (Q8II64)
major facilitator superfamily−related transporter, putative
log2(Fold−Change)
1.25
1.00
0.75
0.50
0.25
EC/IC
EC−BA/IC
Comparison
Phosphosites
S_34;S_35

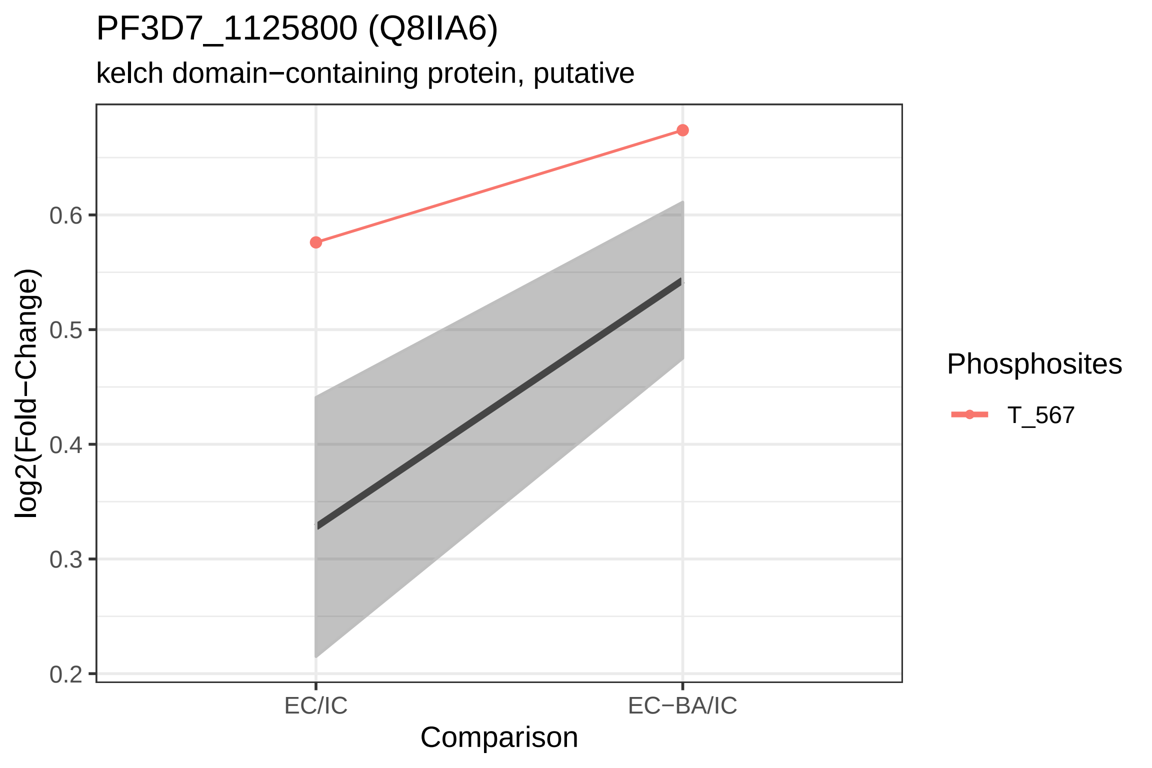
PF3D7_1125800 (Q8IIA6)
kelch domain−containing protein, putative
log2(Fold−Change)
0.6
0.5
0.4
0.3
0.2
EC/IC
EC−BA/IC
Comparison
Phosphosites
T_567

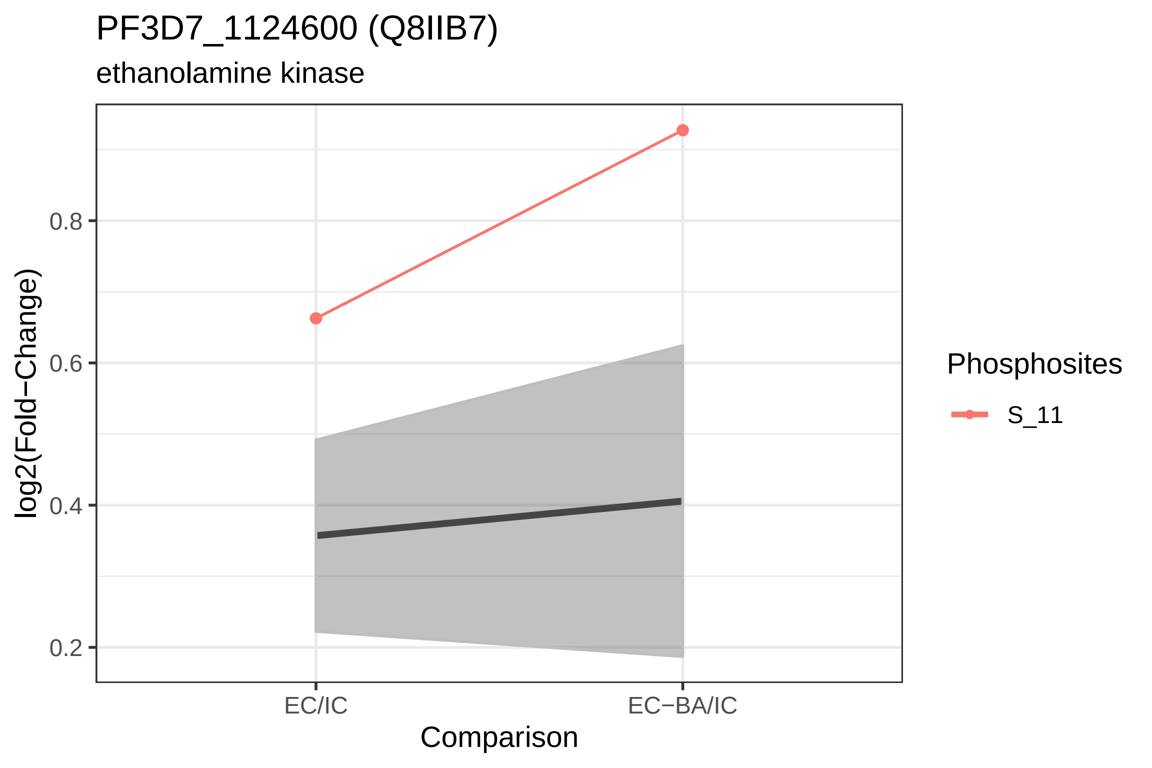
PF3D7_1124600 (Q8IIB7)
ethanolamine kinase
log2(Fold−Change)
0.8
0.6
0.4
0.2
EC/IC
EC−BA/IC
Comparison
Phosphosites
S_11

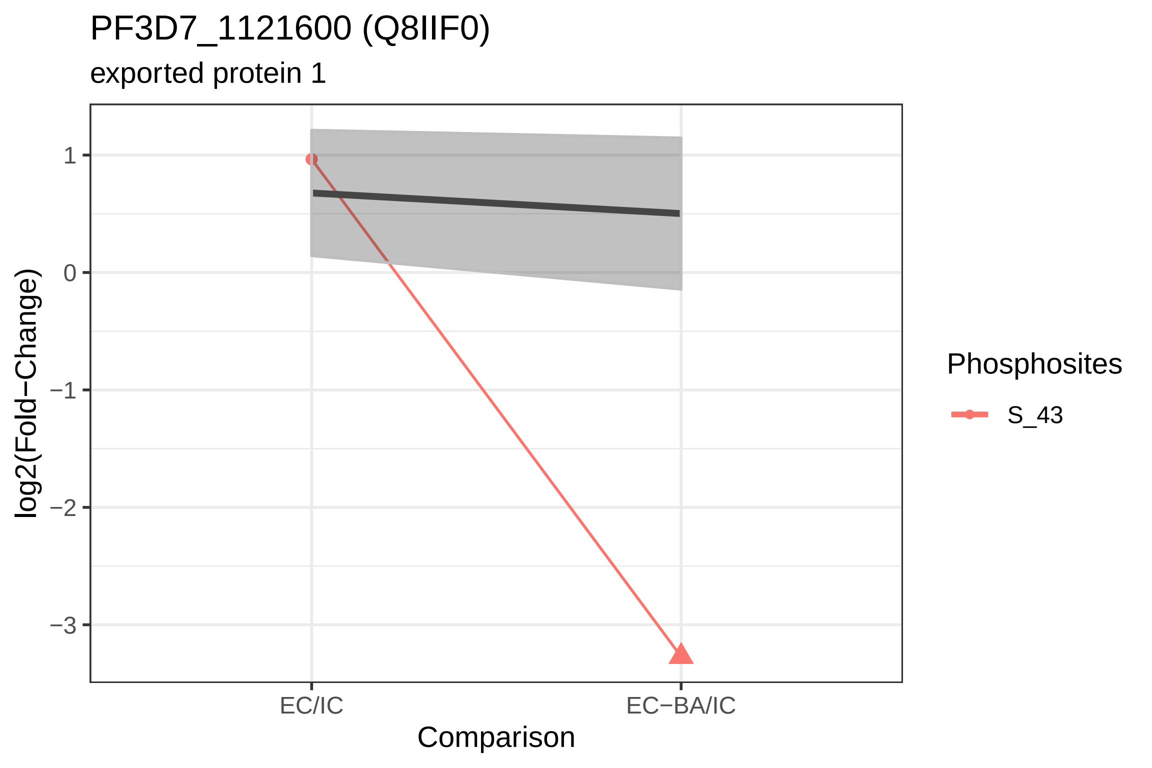

PF3D7_1121600 (Q8IIF0)
exported protein 1
log2(Fold−Change)
1
0
−1
−2
−3
EC/IC
EC−BA/IC
Comparison
Phosphosites
S_43

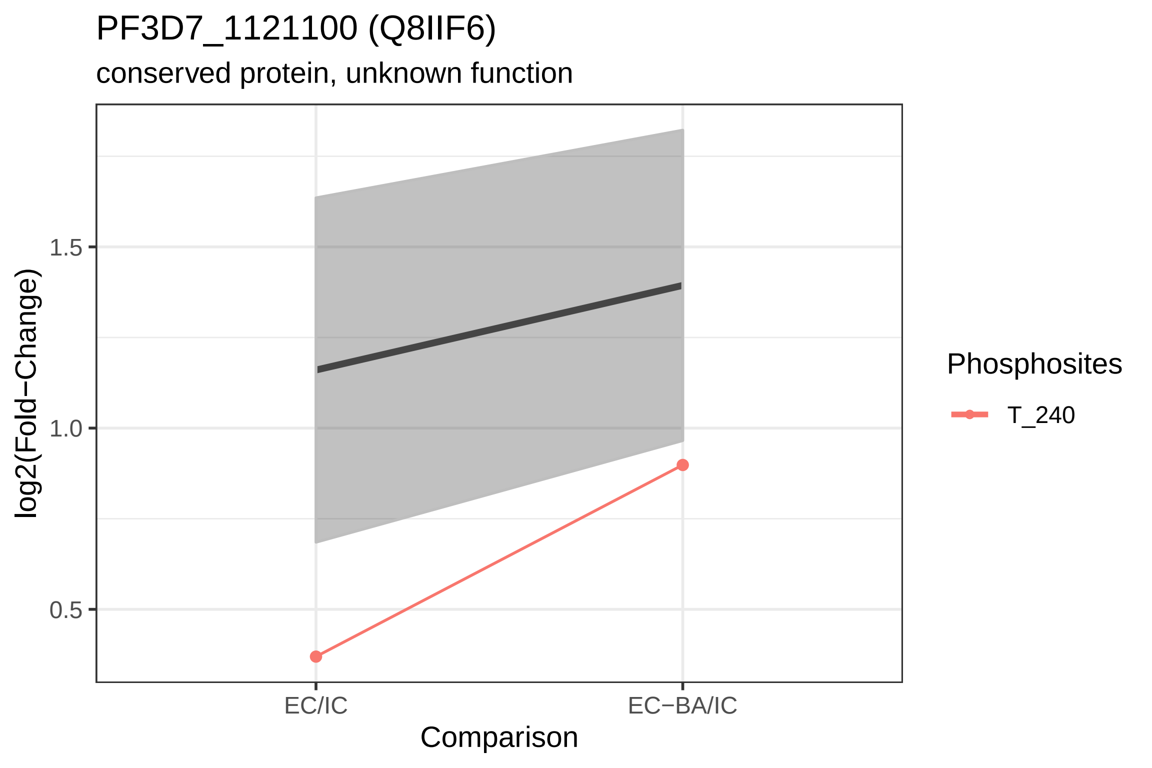
PF3D7_1121100 (Q8IIF6)
conserved protein, unknown function
log2(Fold−Change)
1.5
1.0
0.5
EC/IC
EC−BA/IC
Comparison
Phosphosites
T_240

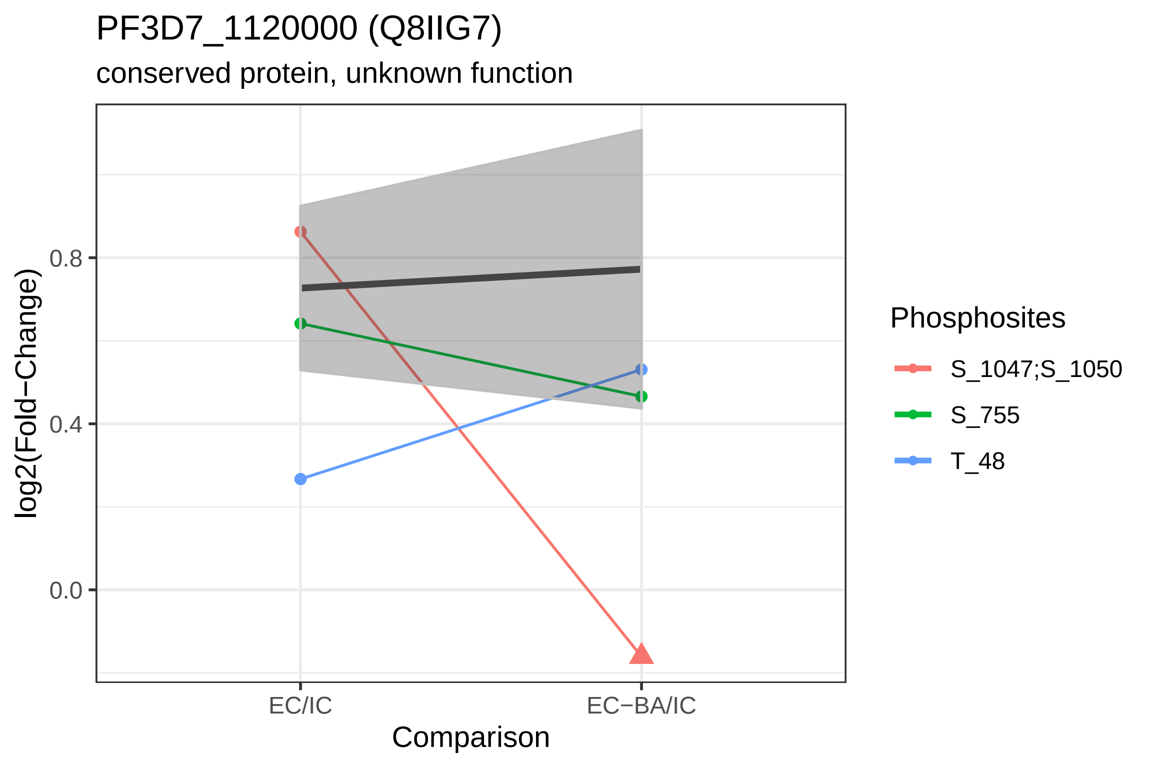

PF3D7_1120000 (Q8IIG7)
conserved protein, unknown function
log2(Fold−Change)
0.8
0.4
0.0
EC/IC
EC−BA/IC
Comparison
Phosphosites
S_1047;S_1050
S_755
T_48

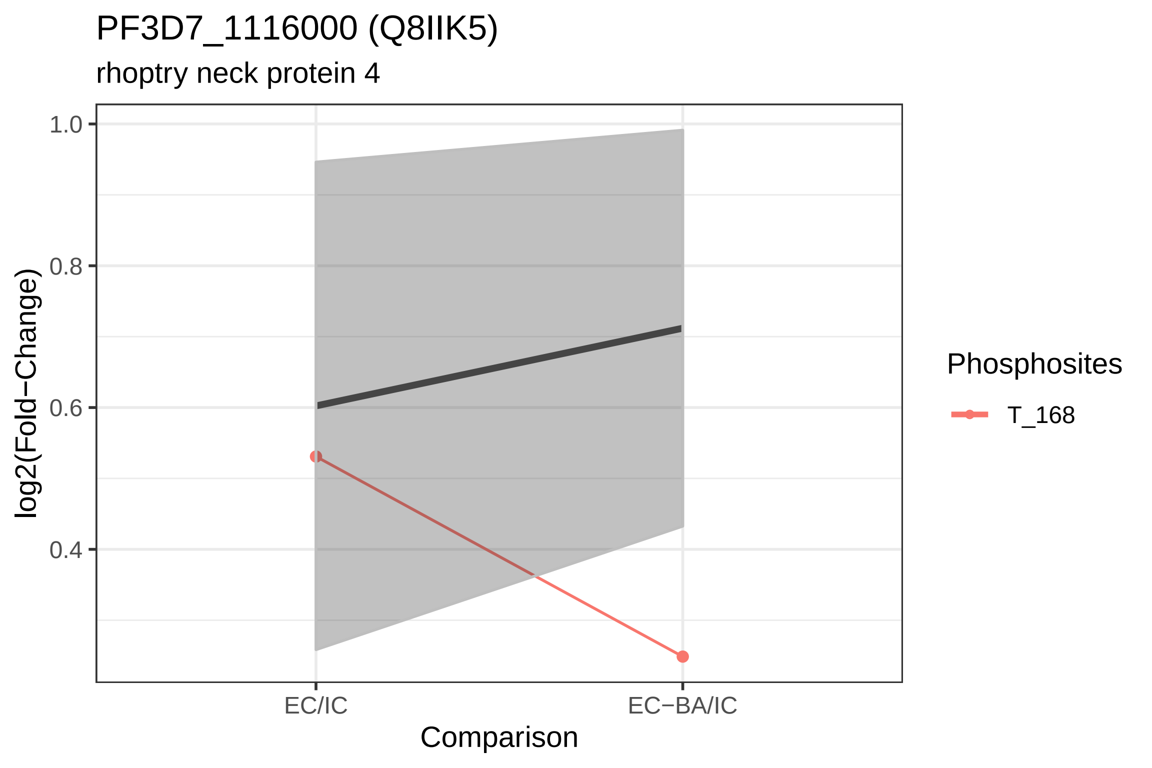

PF3D7_1116000 (Q8IIK5)
rhoptry neck protein 4
1.0
0.8
0.6
0.4
log2(Fold−Change)
EC/IC
EC−BA/IC
Comparison
Phosphosites
T_168

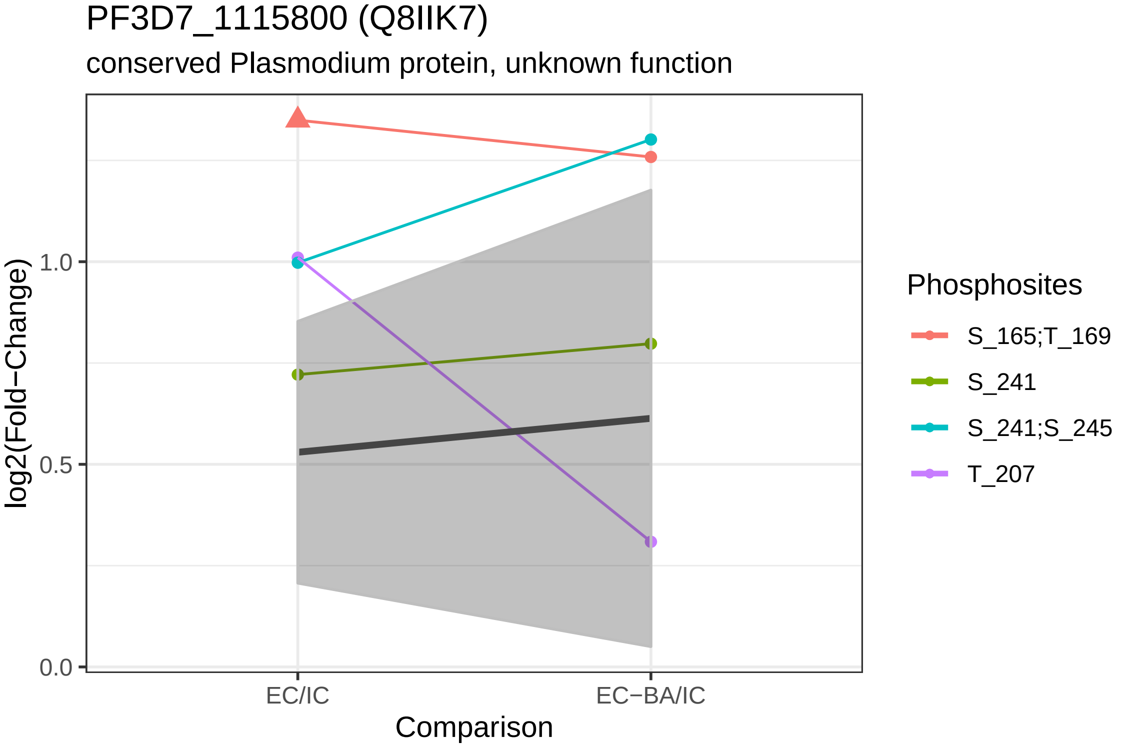
PF3D7_1115800 (Q8IIK7)
conserved Plasmodium protein, unknown function
log2(Fold−Change)
1.0
0.5
0.0
EC/IC
EC−BA/IC
Comparison
Phosphosites
S_165;T_169
S_241
S_241;S_245
T_207

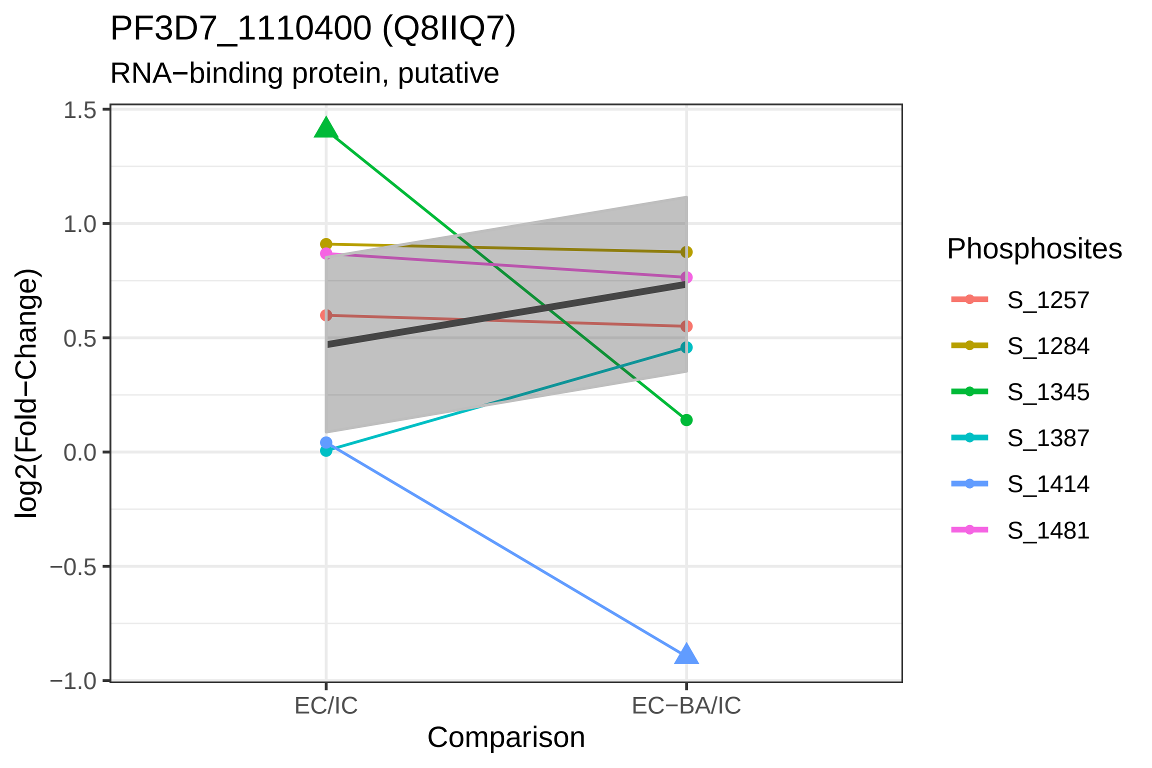

PF3D7_1110400 (Q8IIQ7)
RNA−binding protein, putative
log2(Fold−Change)
1.5
1.0
0.5
0.0
−0.5
−1.0
EC/IC
EC−BA/IC
Comparison
Phosphosites
S_1257
S_1284
S_1345
S_1387
S_1414
S_1481

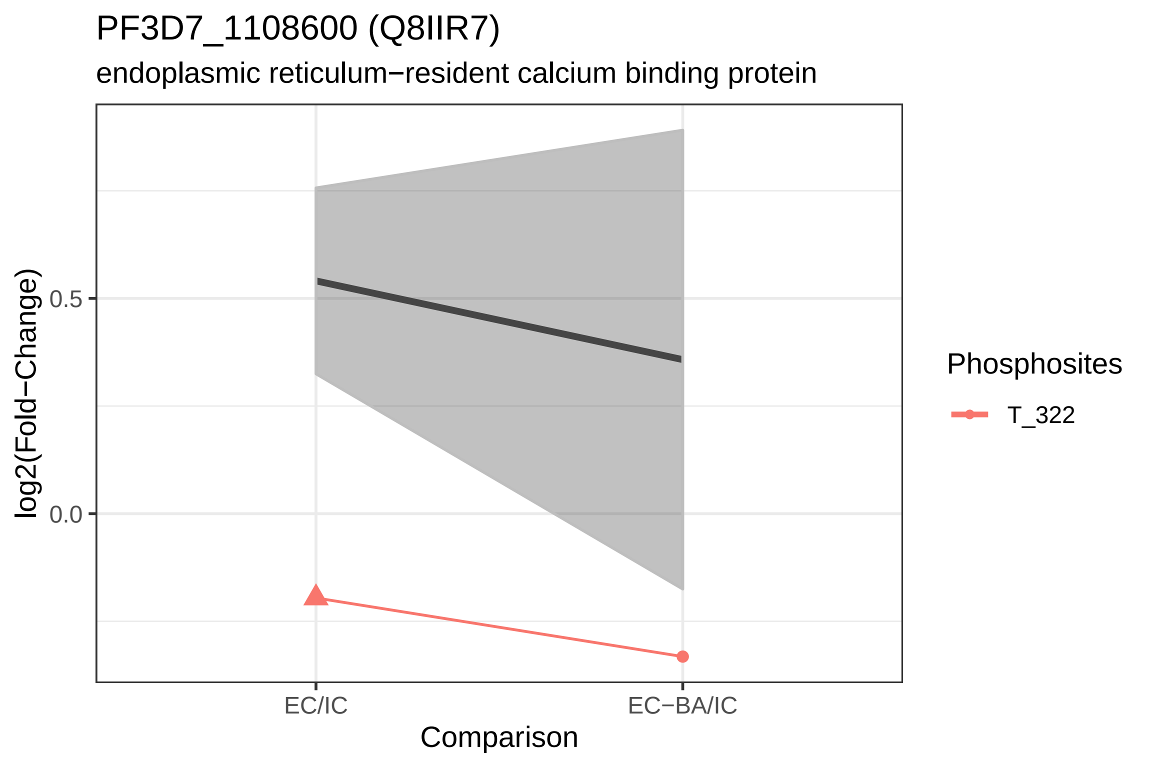
PF3D7_1108600 (Q8IIR7)
endoplasmic reticulum−resident calcium binding protein
log2(Fold−Change)
0.5
0.0
EC/IC
EC−BA/IC
Comparison
Phosphosites
T_322

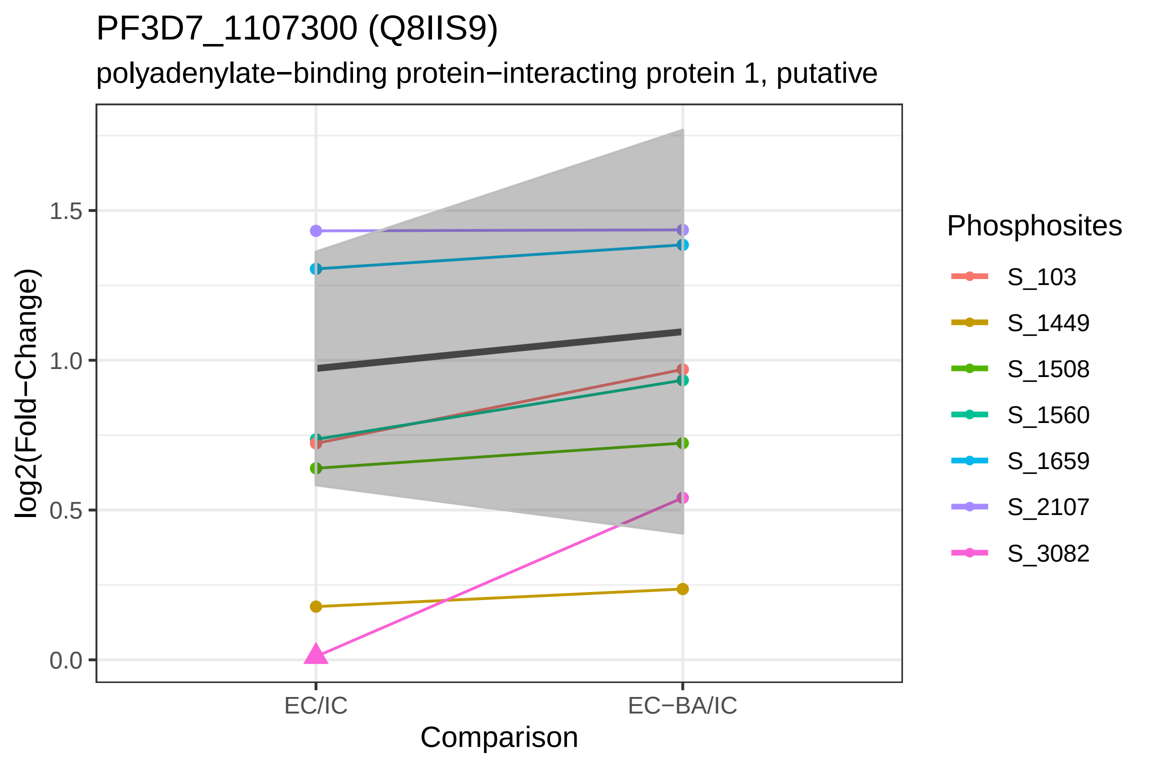
PF3D7_1107300 (Q8IIS9)
polyadenylate−binding protein−interacting protein 1, putative
log2(Fold−Change)
0.0
0.5
1.0
1.5
EC/IC
EC−BA/IC
Comparison
Phosphosites
S_103
S_1449
S_1508
S_1560
S_1659
S_2107
S_3082

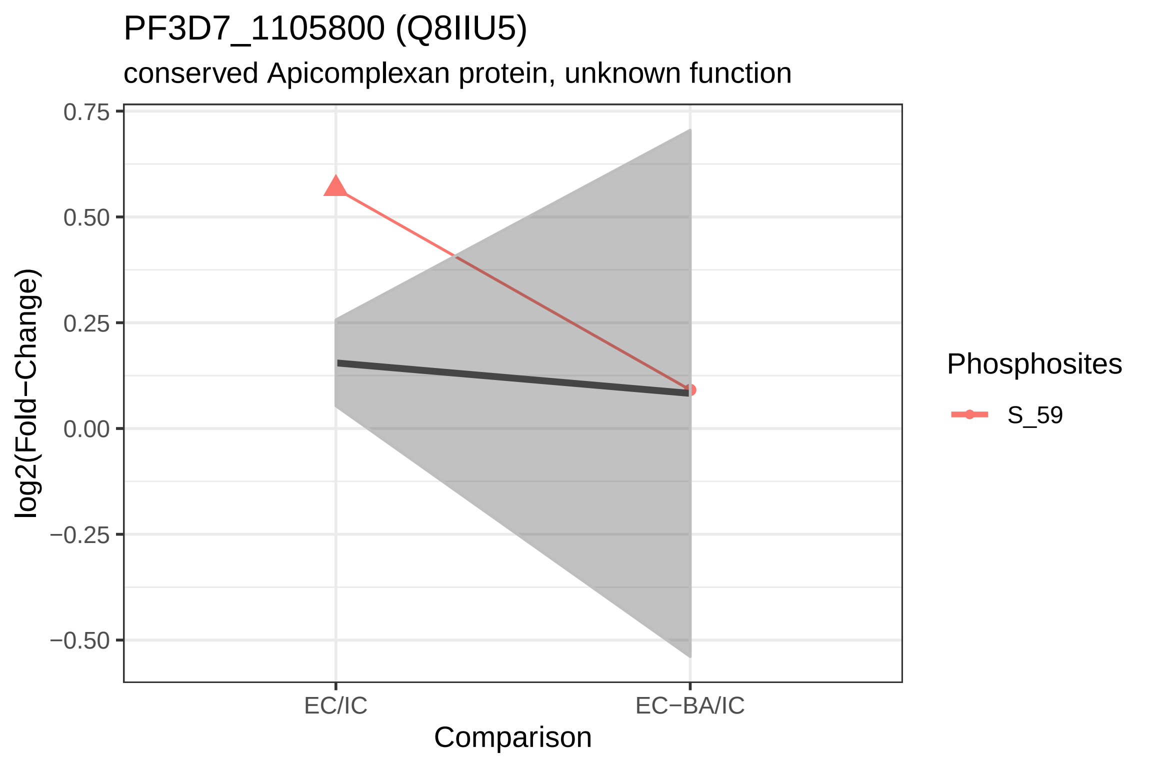

PF3D7_1105800 (Q8IIU5)
conserved Apicomplexan protein, unknown function
0.75
0.50
0.25
0.00
−0.25
−0.50
log2(Fold−Change)
EC/IC
EC−BA/IC
Comparison
Phosphosites
S_59

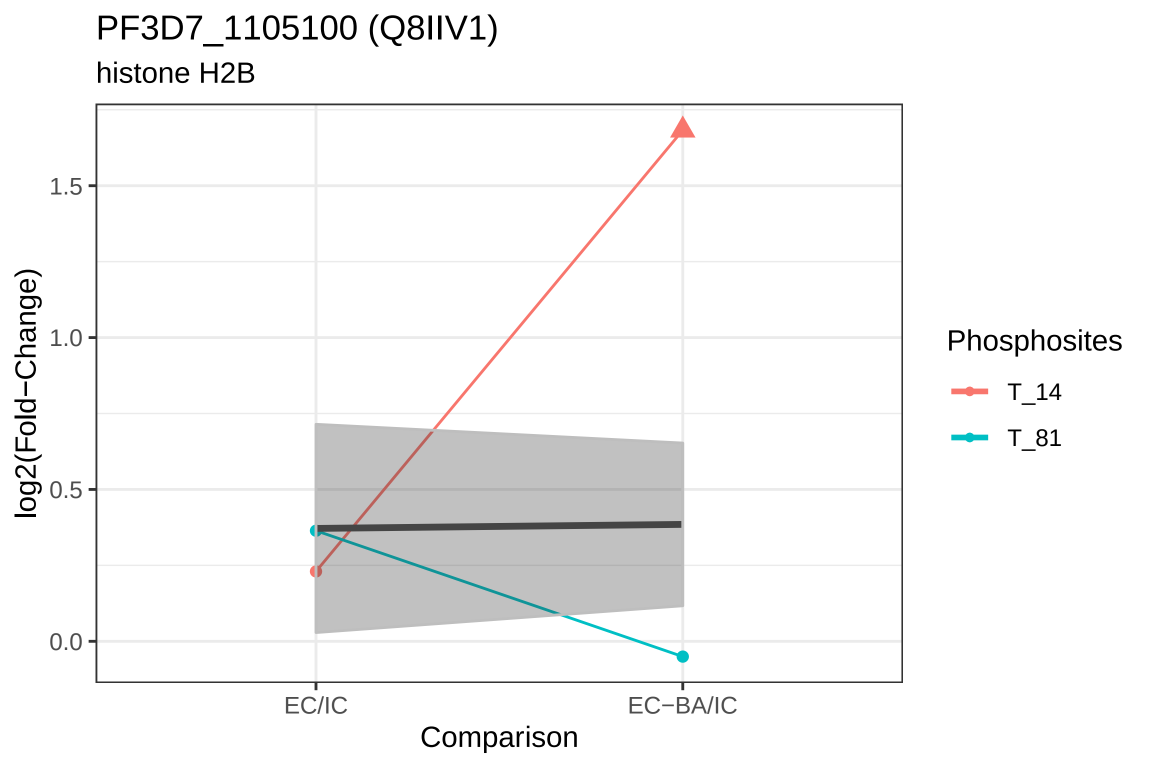

PF3D7_1105100 (Q8IIV1)
histone H2B
log2(Fold−Change)
1.5
1.0
0.5
0.0
EC/IC
EC−BA/IC
Comparison
Phosphosites
T_14
T_81

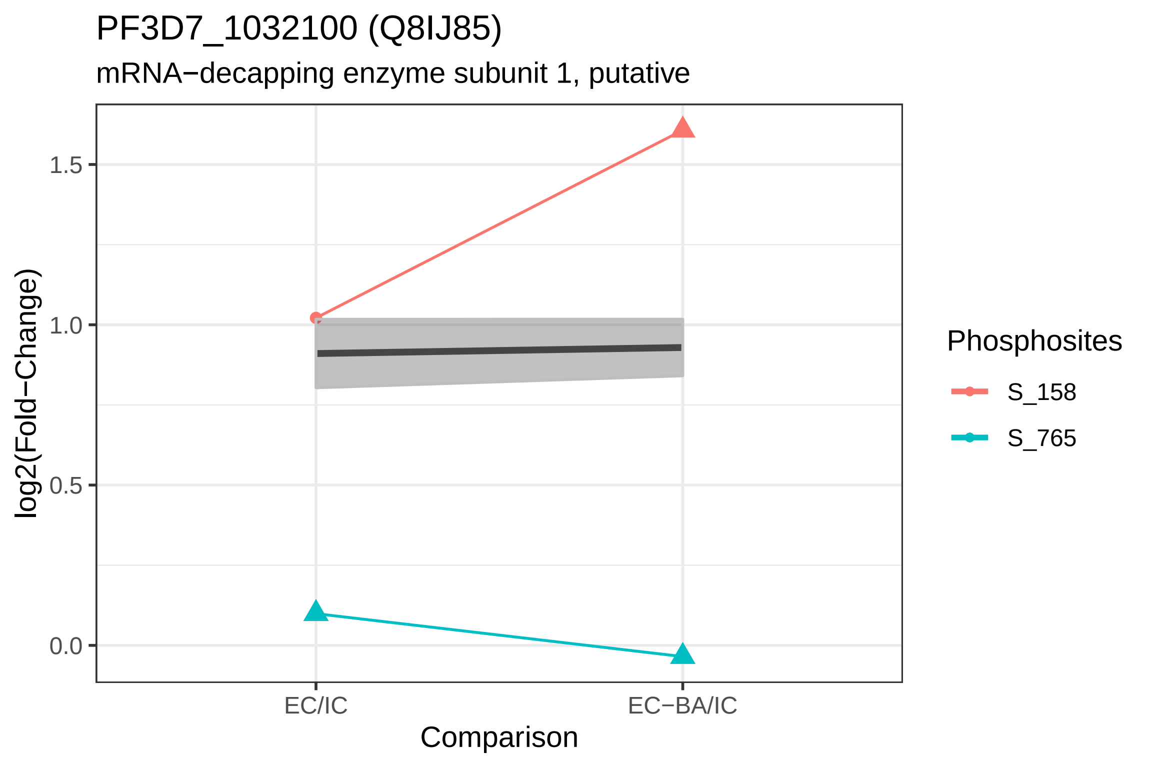
PF3D7_1032100 (Q8IJ85)
mRNA−decapping enzyme subunit 1, putative
log2(Fold−Change)
1.5
1.0
0.5
0.0
EC/IC
EC−BA/IC
Comparison
Phosphosites
S_158
S_765

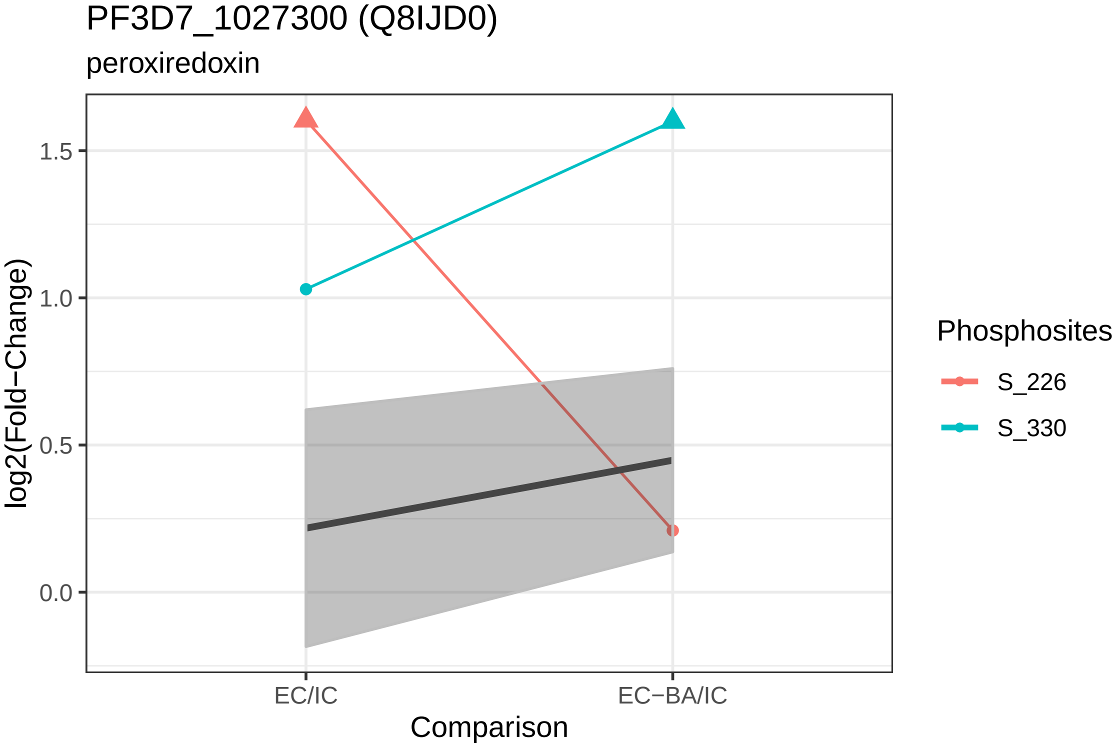

PF3D7_1027300 (Q8IJD0)
peroxiredoxin
log2(Fold−Change)
1.5
1.0
0.5
0.0
EC/IC
EC−BA/IC
Comparison
Phosphosites
S_226
S_330

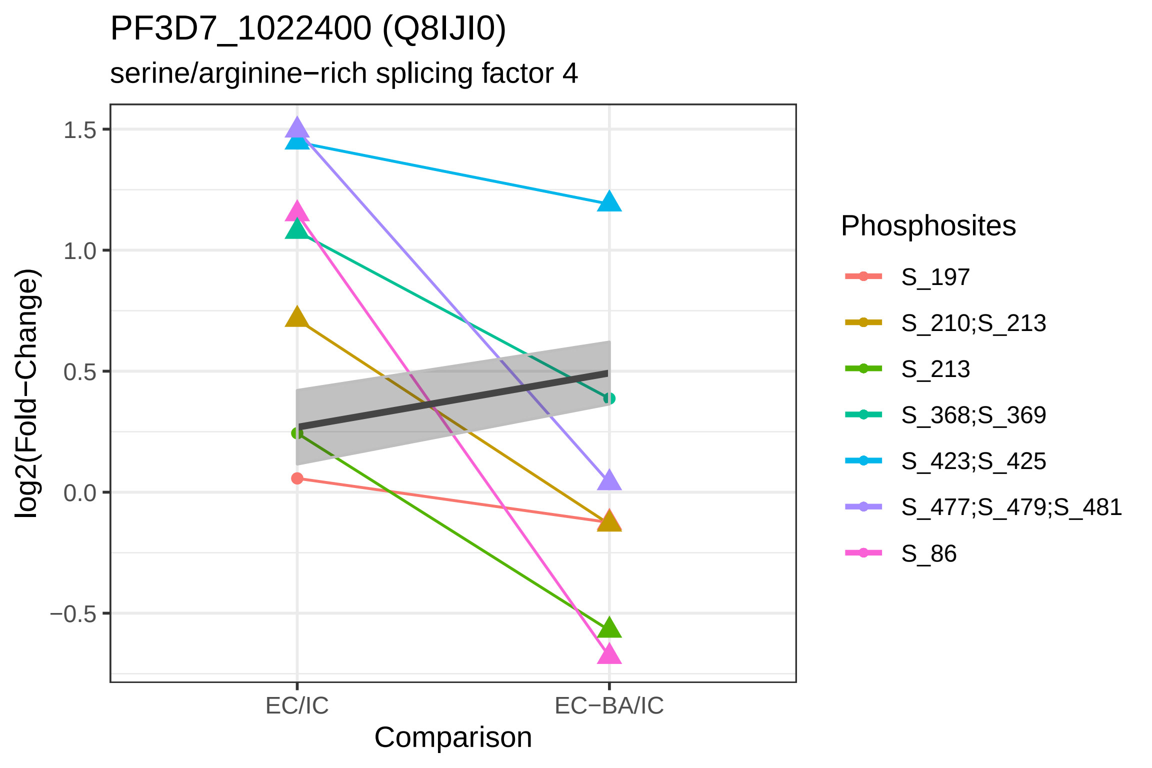

PF3D7_1022400 (Q8IJI0)
serine/arginine−rich splicing factor 4
log2(Fold−Change)
1.5
1.0
0.5
0.0
−0.5
EC/IC
EC−BA/IC
Comparison
Phosphosites
S_197
S_210;S_213
S_213
S_368;S_369
S_423;S_425
S_477;S_479;S_481
S_86

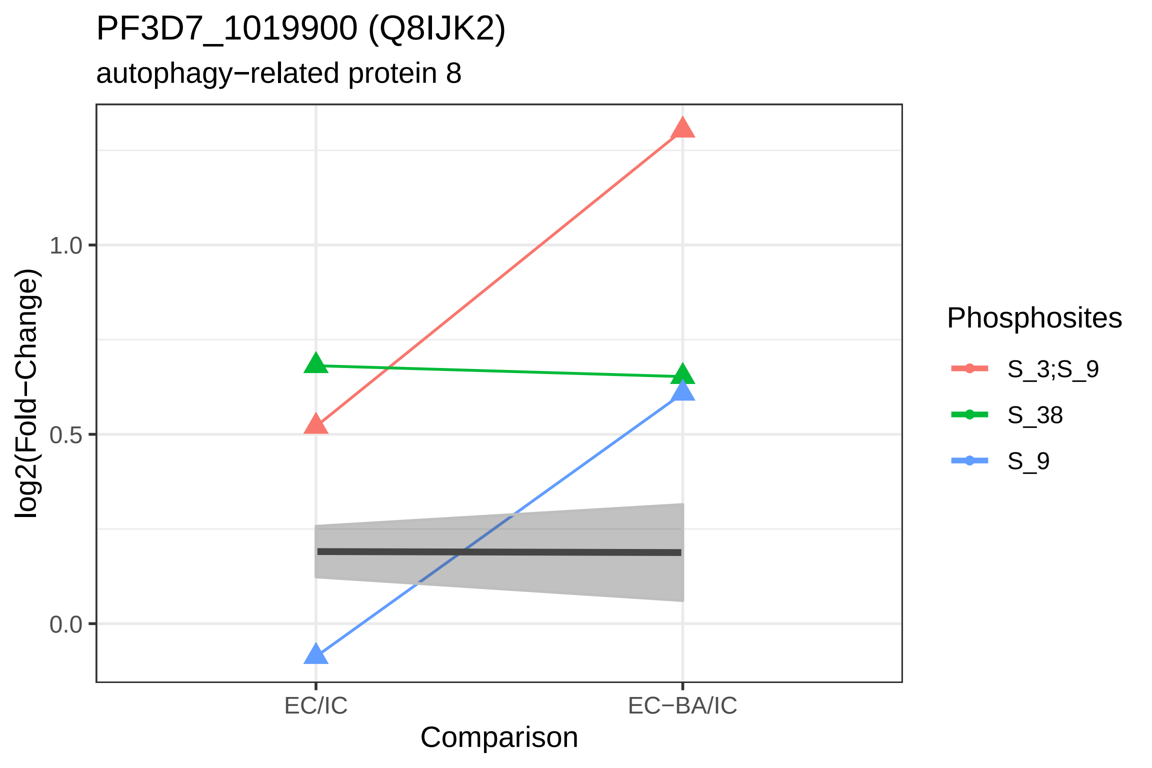
PF3D7_1019900 (Q8IJK2)
autophagy−related protein 8
log2(Fold−Change)
1.0
0.5
0.0
EC/IC
EC−BA/IC
Comparison
Phosphosites
S_3;S_9
S_38
S_9

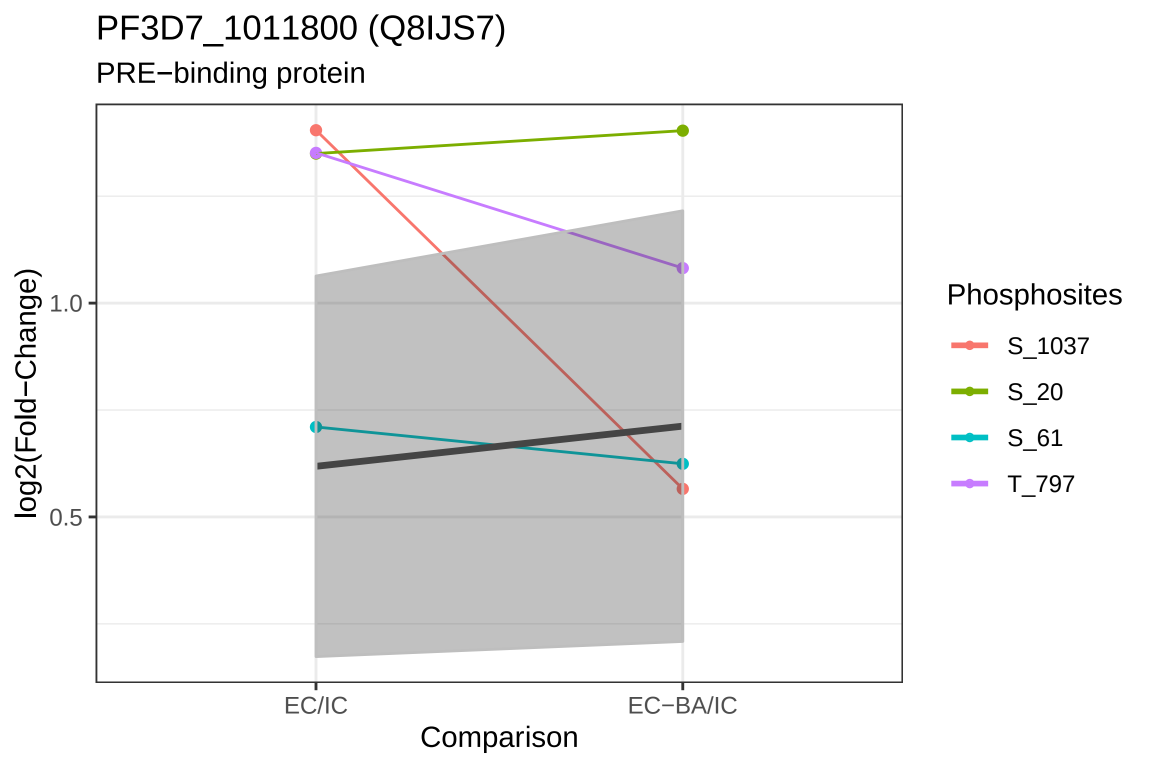
PF3D7_1011800 (Q8IJS7)
PRE−binding protein
log2(Fold−Change)
1.0
0.5
EC/IC
EC−BA/IC
Comparison
Phosphosites
S_1037
S_20
S_61
T_797

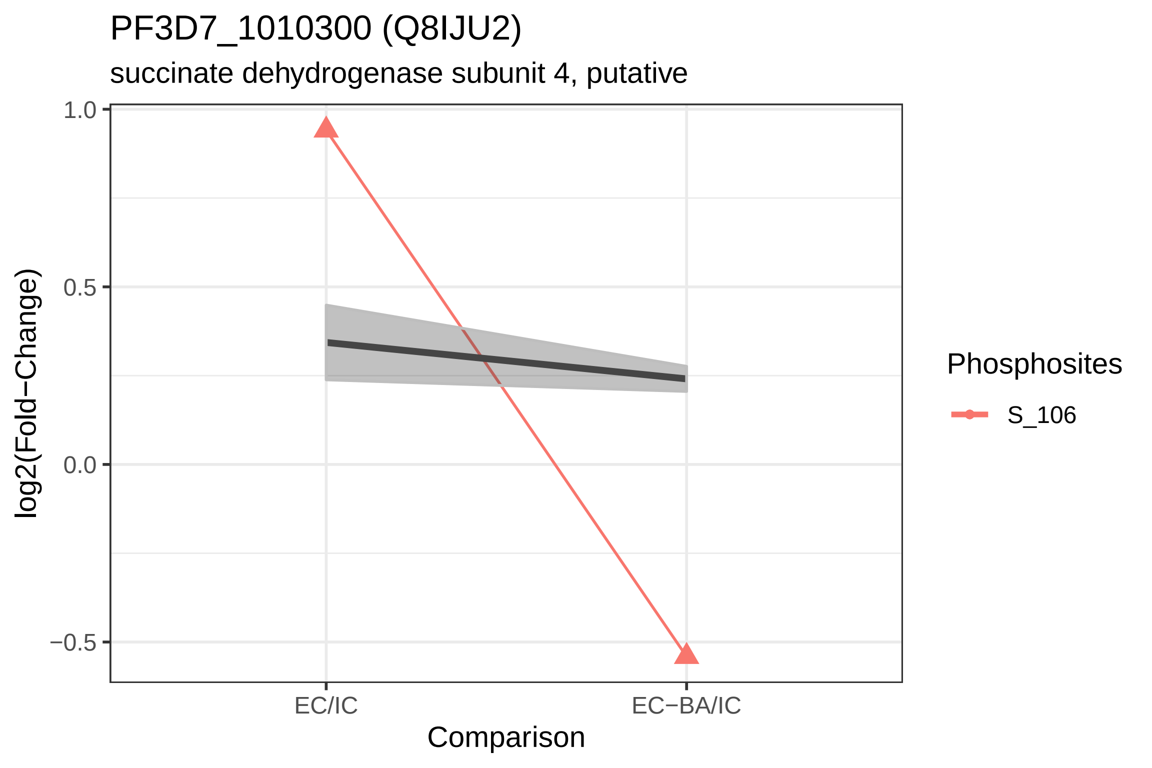

PF3D7_1010300 (Q8IJU2)
succinate dehydrogenase subunit 4, putative
1.0
0.5
0.0
−0.5
log2(Fold−Change)
EC/IC
EC−BA/IC
Comparison
Phosphosites
S_106

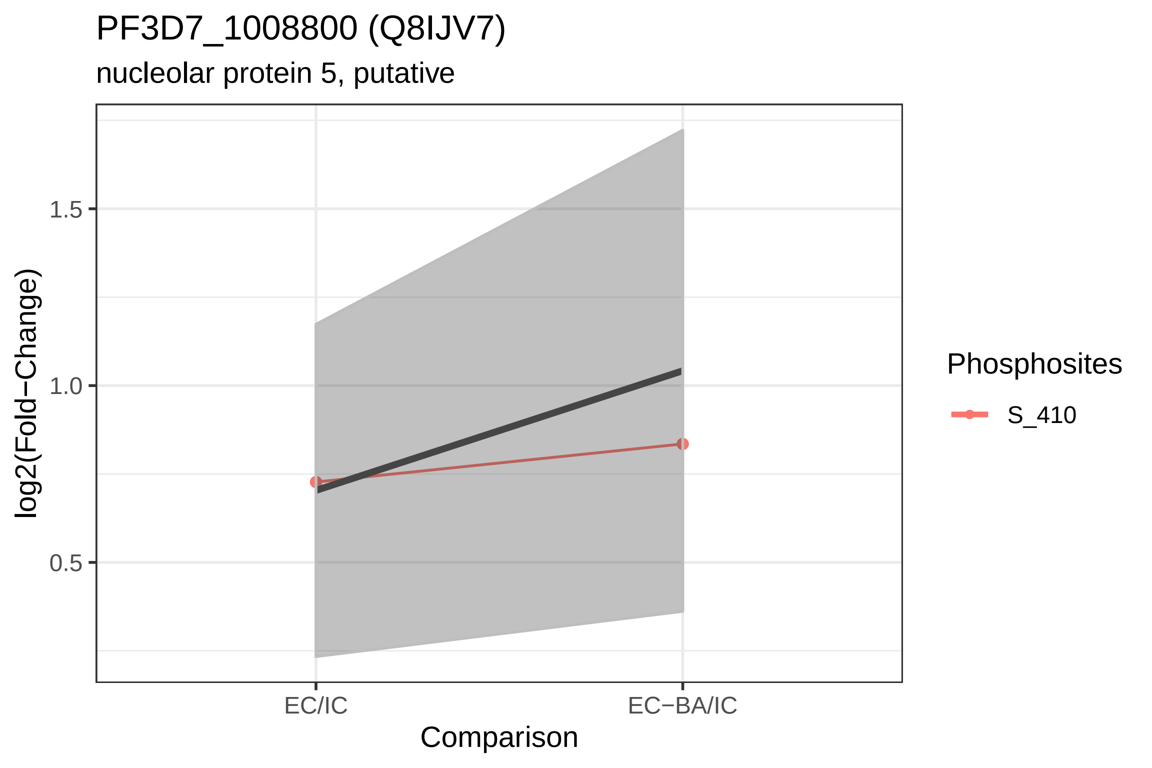

PF3D7_1008800 (Q8IJV7)
nucleolar protein 5, putative
log2(Fold−Change)
1.5
1.0
0.5
EC/IC
EC−BA/IC
Comparison
Phosphosites
S_410

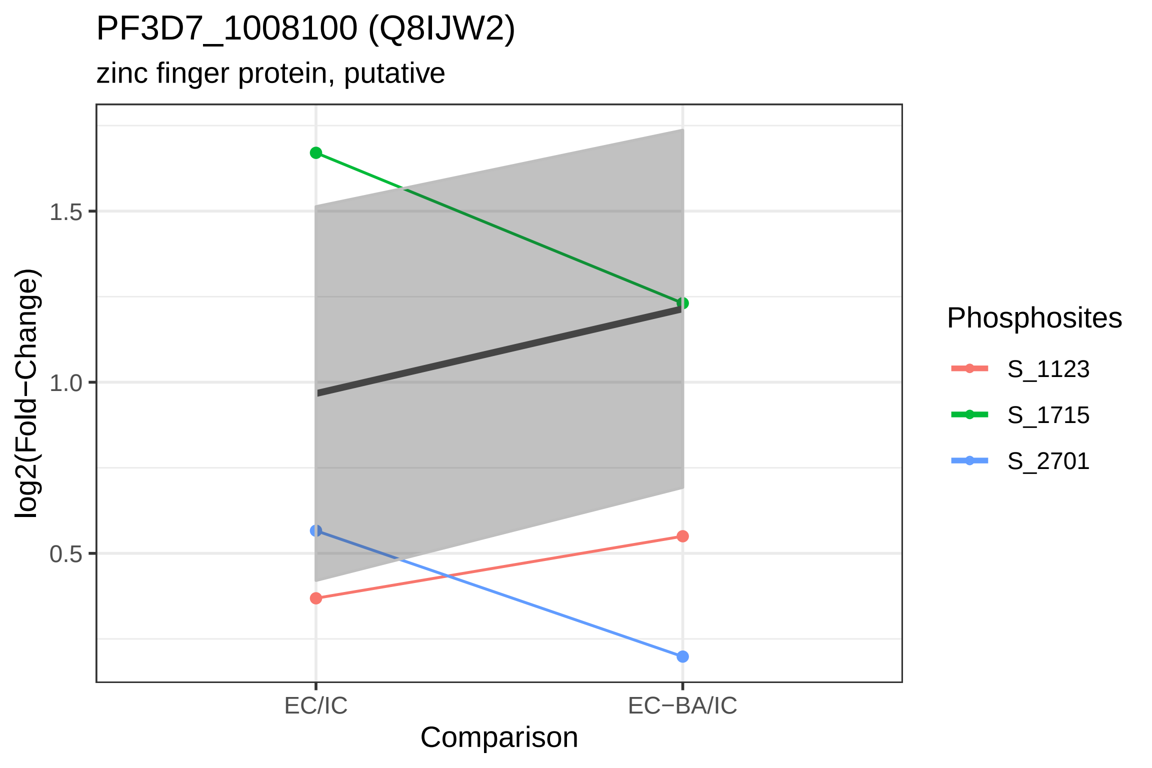
PF3D7_1008100 (Q8IJW2)
zinc finger protein, putative
log2(Fold−Change)
1.5
1.0
0.5
EC/IC
EC−BA/IC
Comparison
Phosphosites
S_1123
S_1715
S_2701

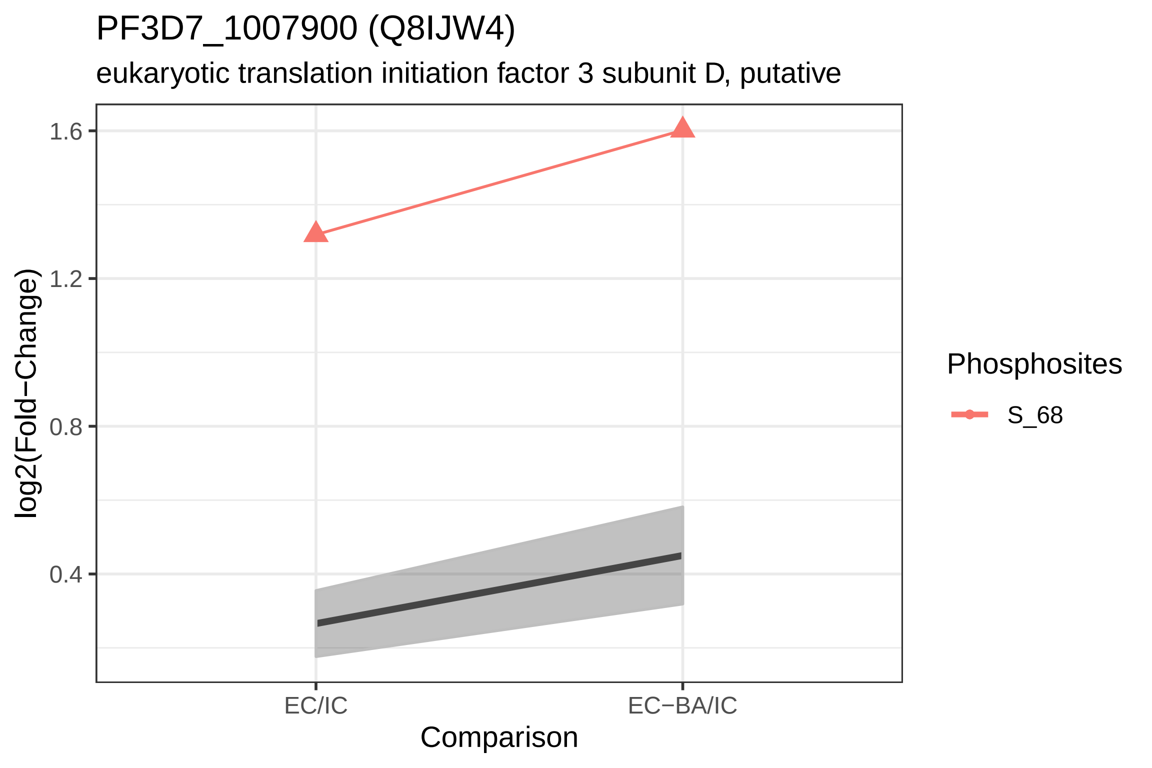
PF3D7_1007900 (Q8IJW4)
eukaryotic translation initiation factor 3 subunit D, putative
log2(Fold−Change)
1.6
1.2
0.8
0.4
EC/IC
EC−BA/IC
Comparison
Phosphosites
S_68

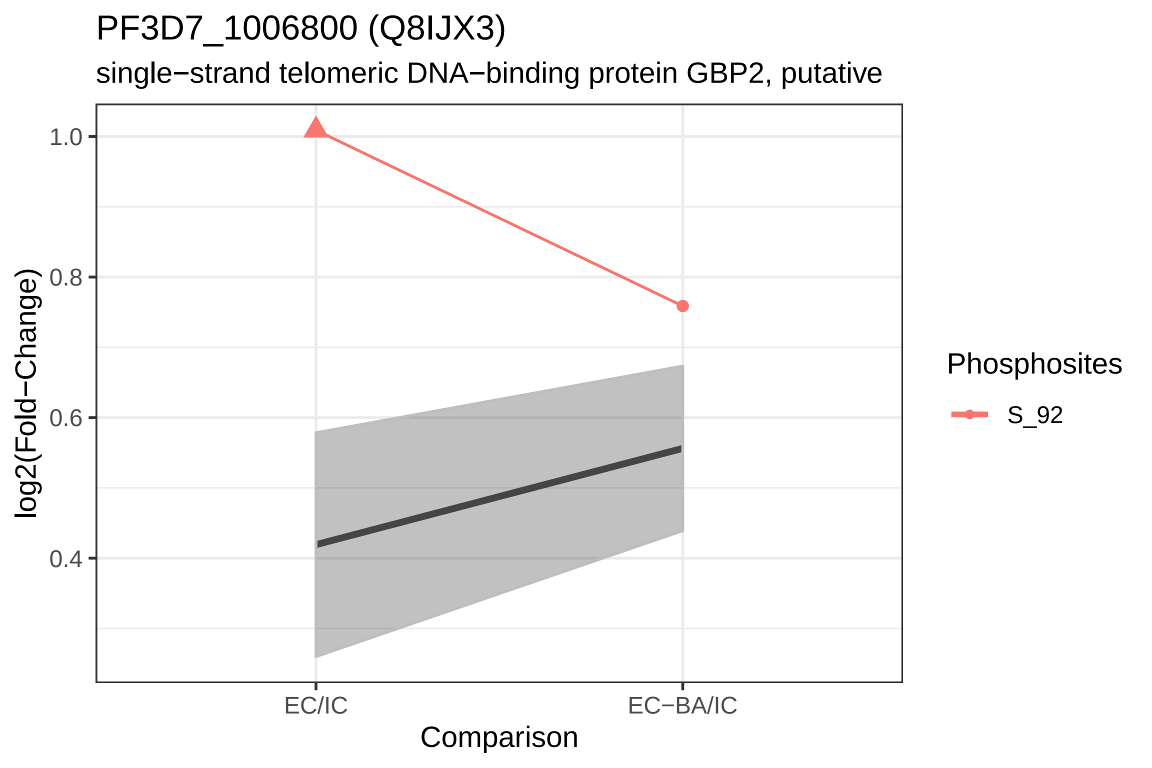
PF3D7_1006800 (Q8IJX3)
single−strand telomeric DNA−binding protein GBP2, putative
log2(Fold−Change)
1.0
0.8
0.6
0.4
EC/IC
EC−BA/IC
Comparison
Phosphosites
S_92

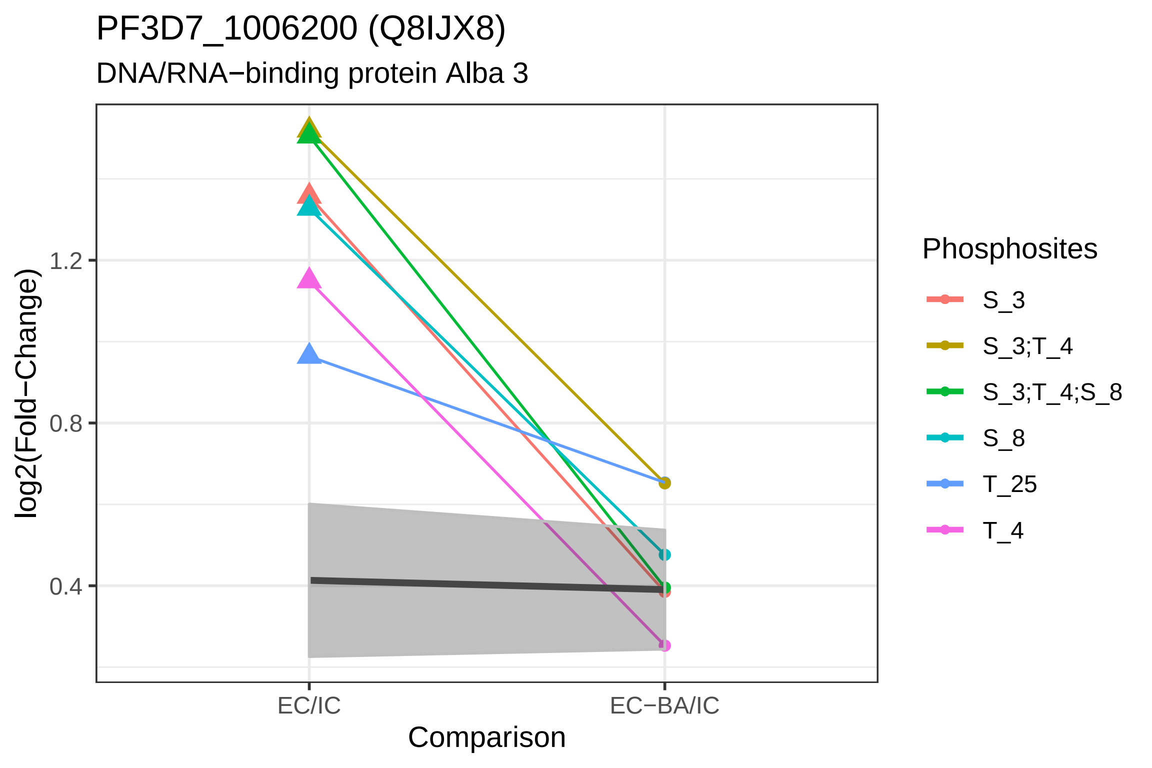

PF3D7_1006200 (Q8IJX8)
DNA/RNA−binding protein Alba 3
log2(Fold−Change)
1.2
0.8
0.4
EC/IC
EC−BA/IC
Comparison
Phosphosites
S_3
S_3;T_4
S_3;T_4;S_8
S_8
T_25
T_4

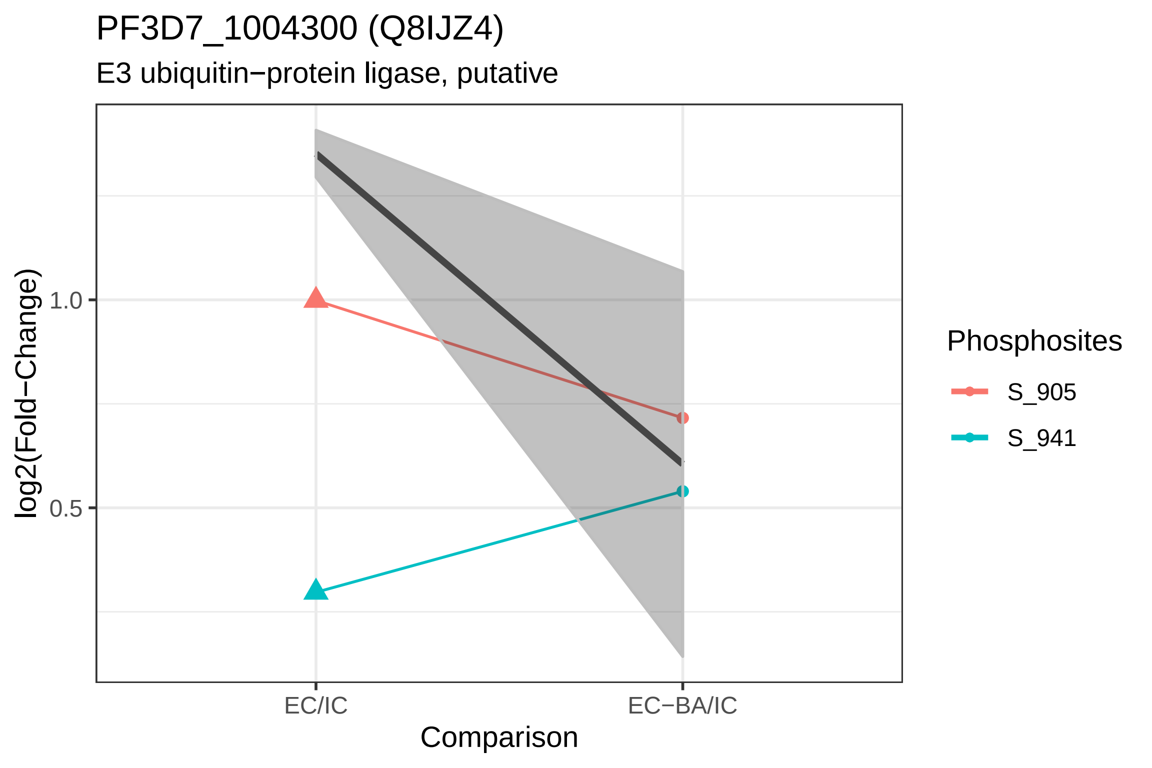

PF3D7_1004300 (Q8IJZ4)
E3 ubiquitin−protein ligase, putative
log2(Fold−Change)
1.0
0.5
EC/IC
EC−BA/IC
Comparison
Phosphosites
S_905
S_941

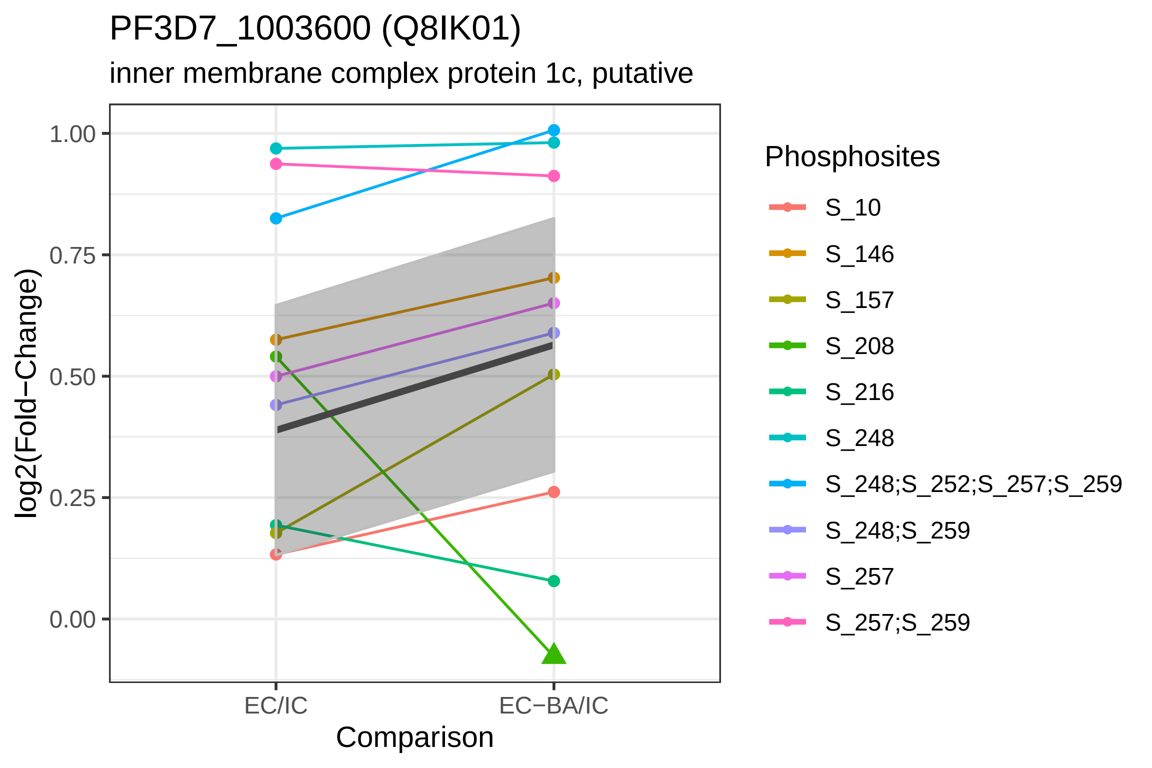
PF3D7_1003600 (Q8IK01)
inner membrane complex protein 1c, putative
log2(Fold−Change)
1.00
0.75
0.50
0.25
0.00
EC/IC
EC−BA/IC
Comparison
Phosphosites
S_10
S_146
S_157
S_208
S_216
S_248
S_248;S_252;S_257;S_259
S_248;S_259
S_257
S_257;S_259

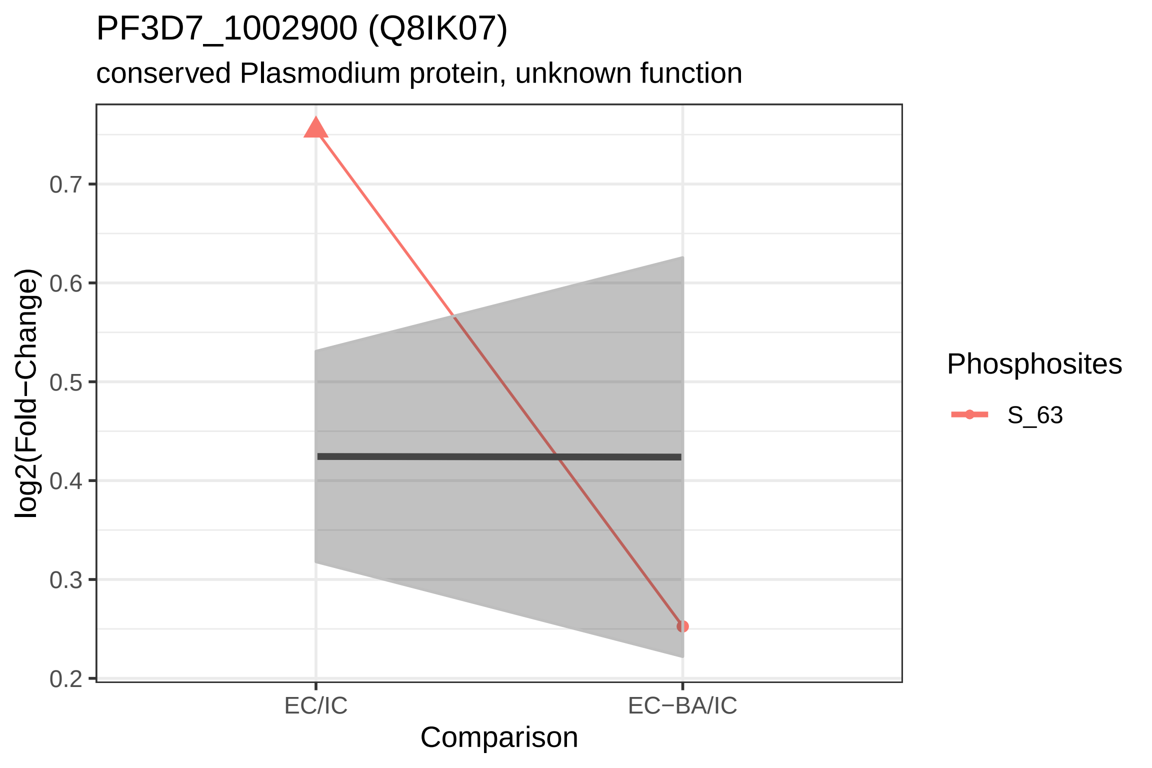

PF3D7_1002900 (Q8IK07)
conserved Plasmodium protein, unknown function
log2(Fold−Change)
0.7
0.6
0.5
0.4
0.3
0.2
EC/IC
EC−BA/IC
Comparison
Phosphosites
S_63

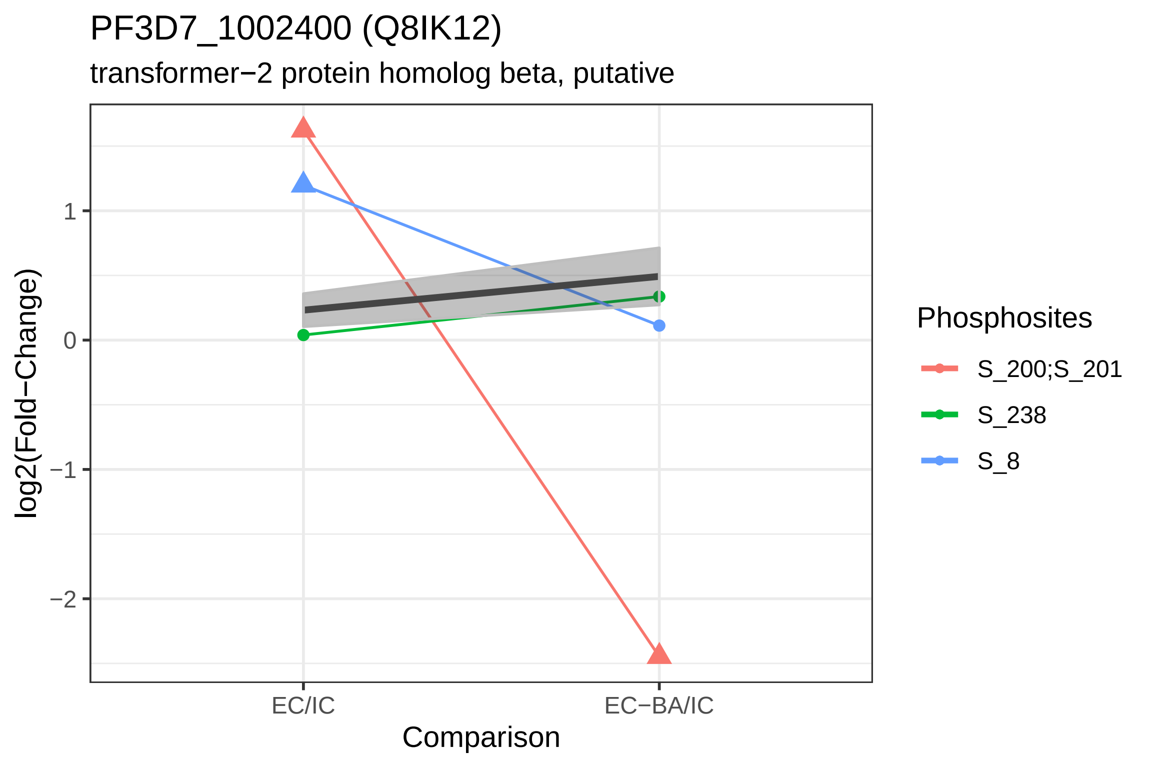

PF3D7_1002400 (Q8IK12)
transformer−2 protein homolog beta, putative
log2(Fold−Change)
1
0
−1
−2
EC/IC
EC−BA/IC
Comparison
Phosphosites
S_200;S_201
S_238
S_8

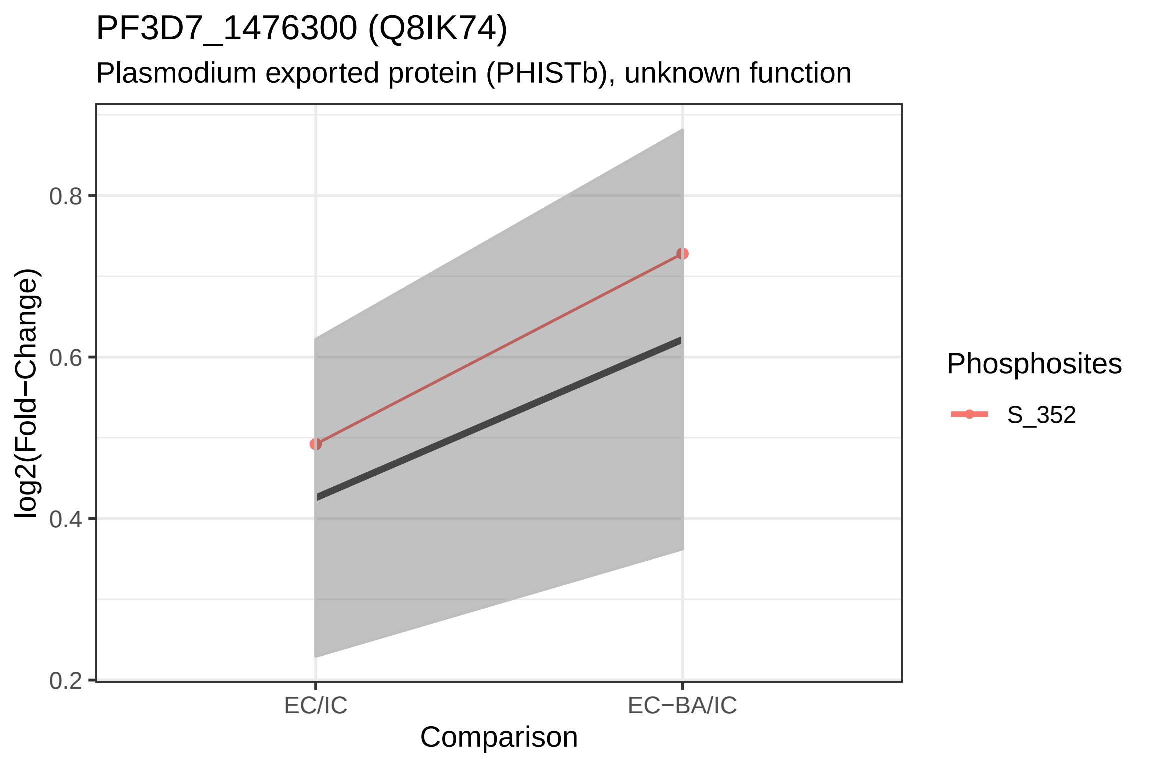

PF3D7_1476300 (Q8IK74)
Plasmodium exported protein (PHISTb), unknown function
log2(Fold−Change)
0.8
0.6
0.4
0.2
EC/IC
EC−BA/IC
Comparison
Phosphosites
S_352

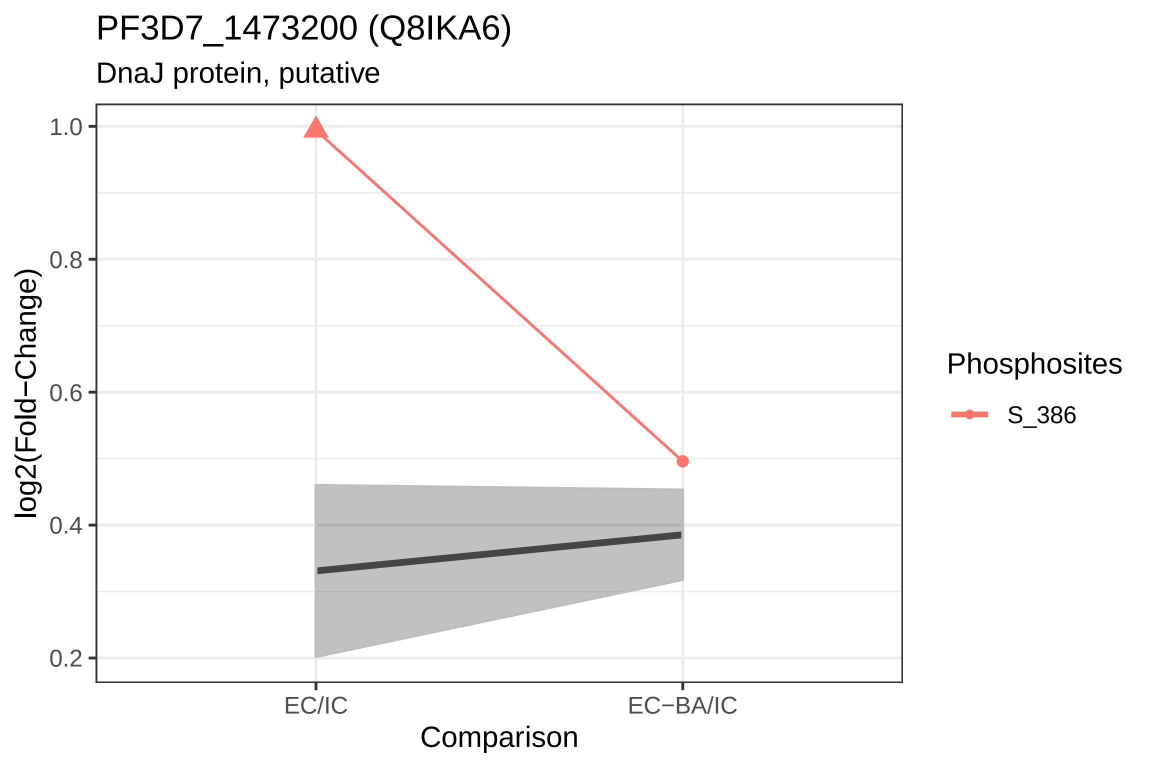
PF3D7_1473200 (Q8IKA6)
DnaJ protein, putative
log2(Fold−Change)
1.0
0.8
0.6
0.4
0.2
EC/IC
EC−BA/IC
Comparison
Phosphosites
S_386

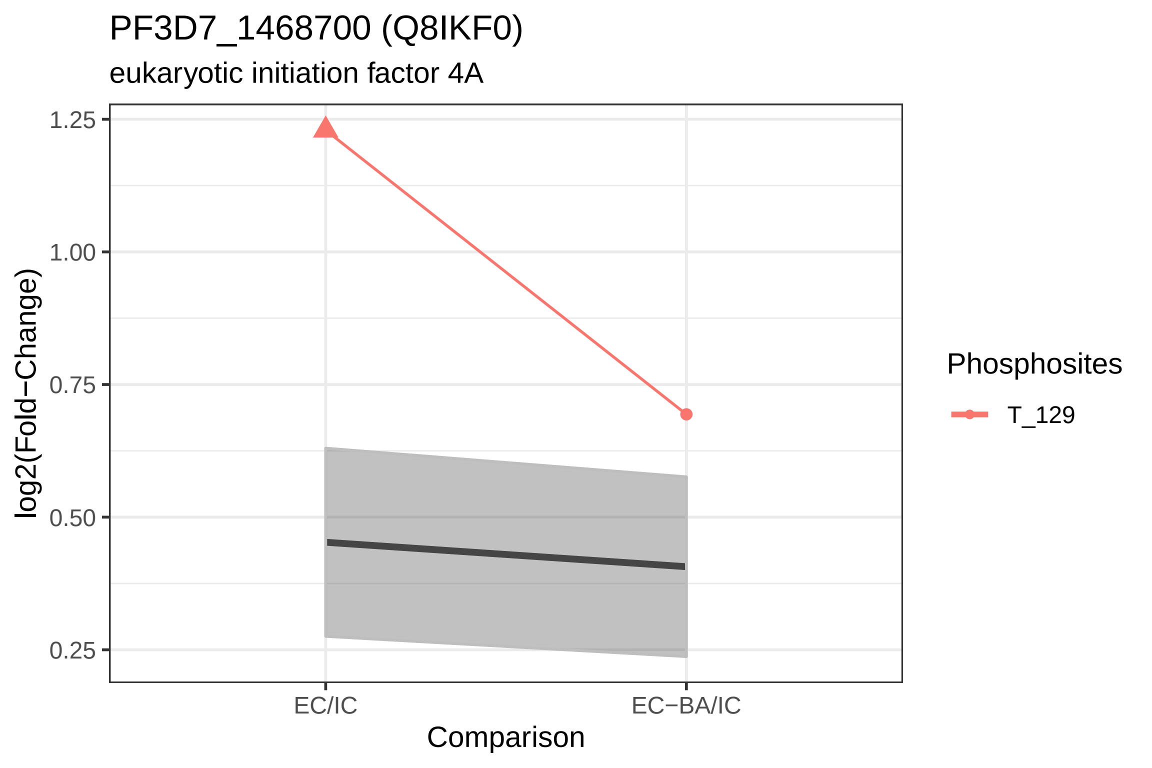

PF3D7_1468700 (Q8IKF0)
eukaryotic initiation factor 4A
log2(Fold−Change)
1.25
1.00
0.75
0.50
0.25
EC/IC
EC−BA/IC
Comparison
Phosphosites
T_129

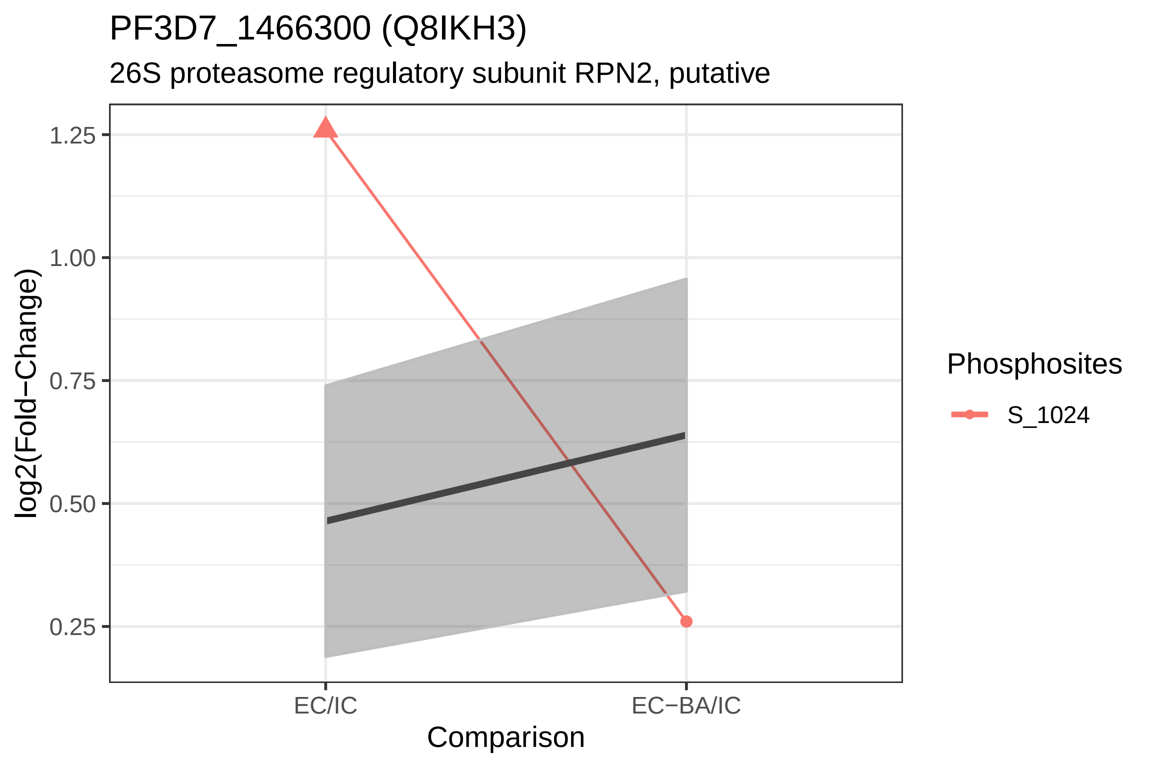
PF3D7_1466300 (Q8IKH3)
26S proteasome regulatory subunit RPN2, putative
1.25
1.00
0.75
0.50
0.25
log2(Fold−Change)
EC/IC
EC−BA/IC
Comparison
Phosphosites
S_1024

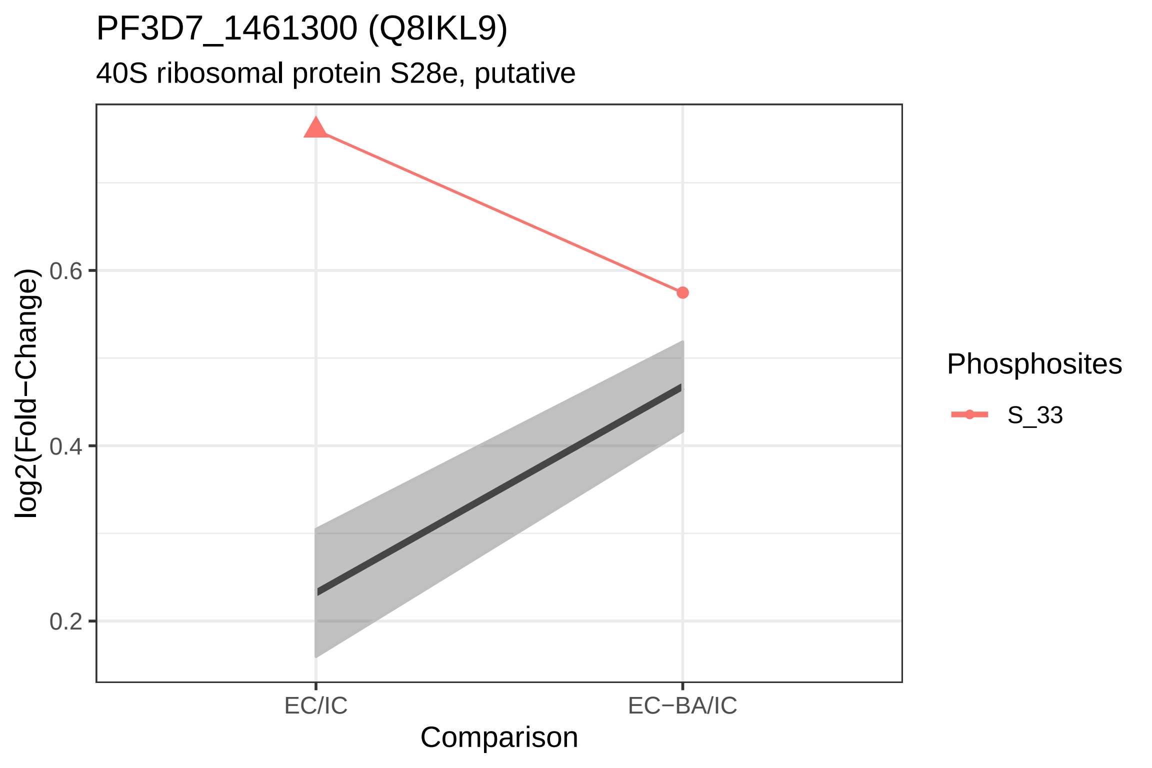
PF3D7_1461300 (Q8IKL9)
40S ribosomal protein S28e, putative
log2(Fold−Change)
0.6
0.4
0.2
EC/IC
EC−BA/IC
Comparison
Phosphosites
S_33

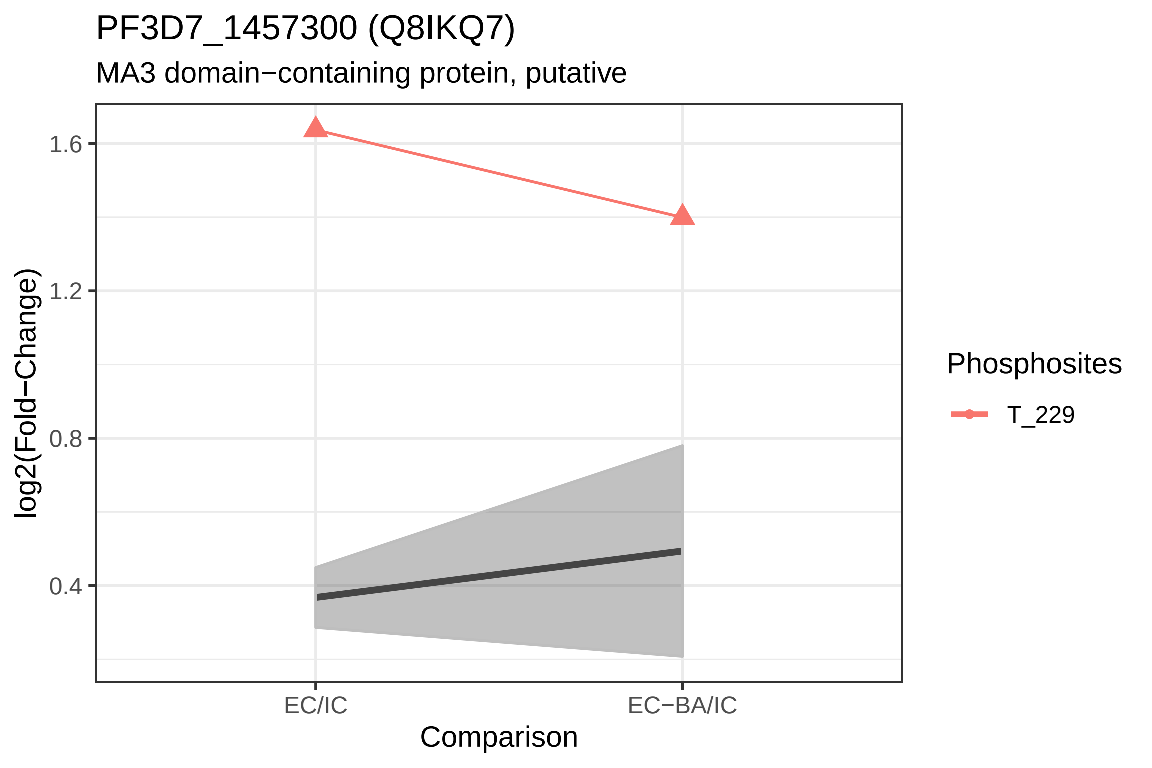

PF3D7_1457300 (Q8IKQ7)
MA3 domain−containing protein, putative
log2(Fold−Change)
1.6
1.2
0.8
0.4
EC/IC
EC−BA/IC
Comparison
Phosphosites
T_229

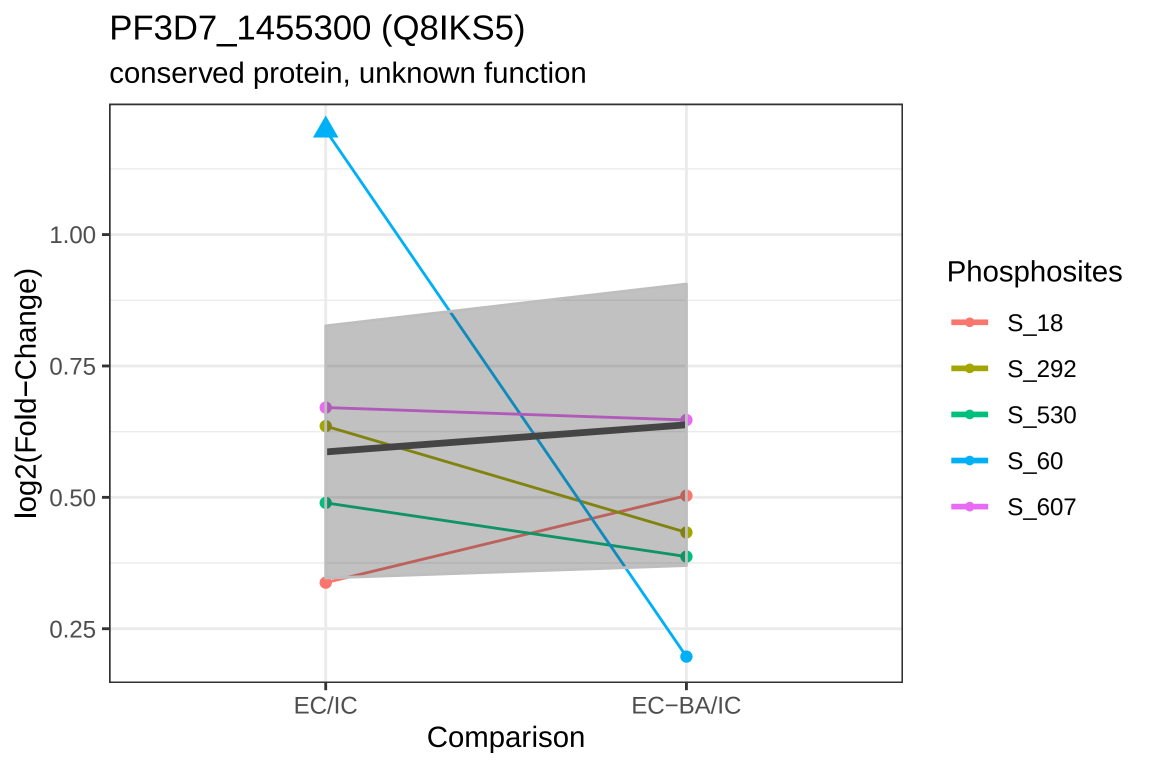
PF3D7_1455300 (Q8IKS5)
conserved protein, unknown function
log2(Fold−Change)
1.00
0.75
0.50
0.25
EC/IC
EC−BA/IC
Comparison
Phosphosites
S_18
S_292
S_530
S_60
S_607

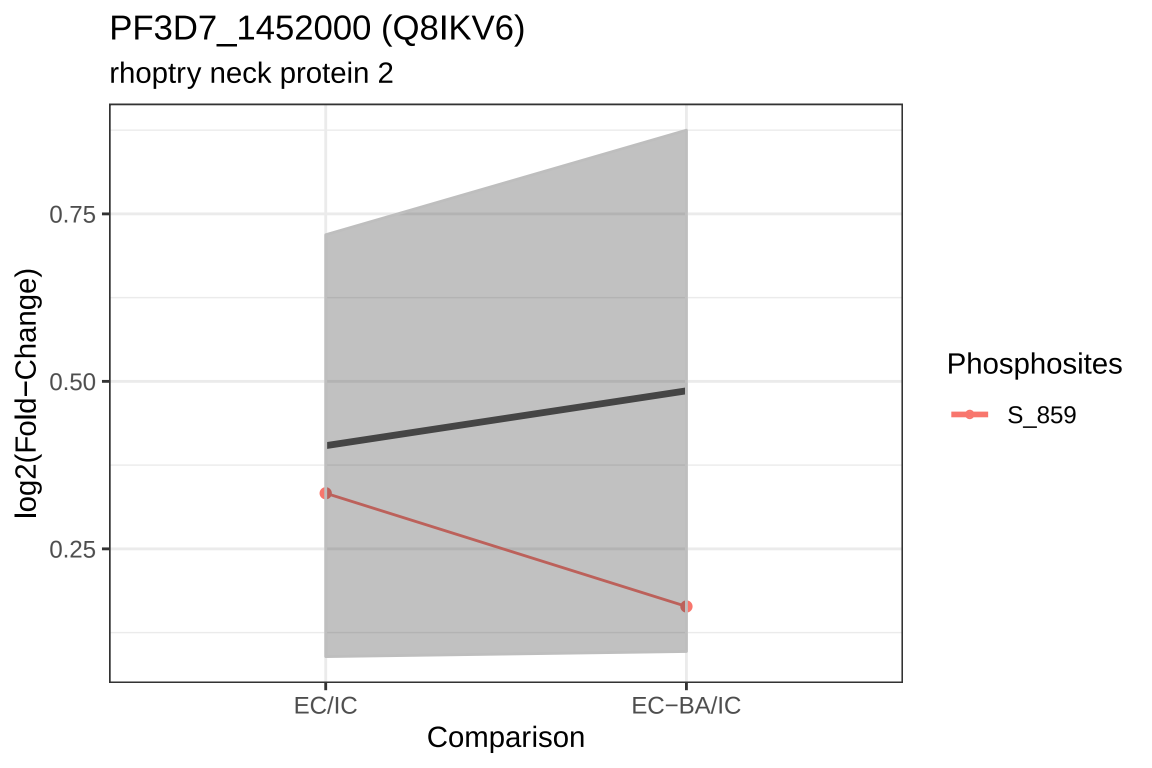

PF3D7_1452000 (Q8IKV6)
rhoptry neck protein 2
log2(Fold−Change)
0.75
0.50
0.25
EC/IC
EC−BA/IC
Comparison
Phosphosites
S_859

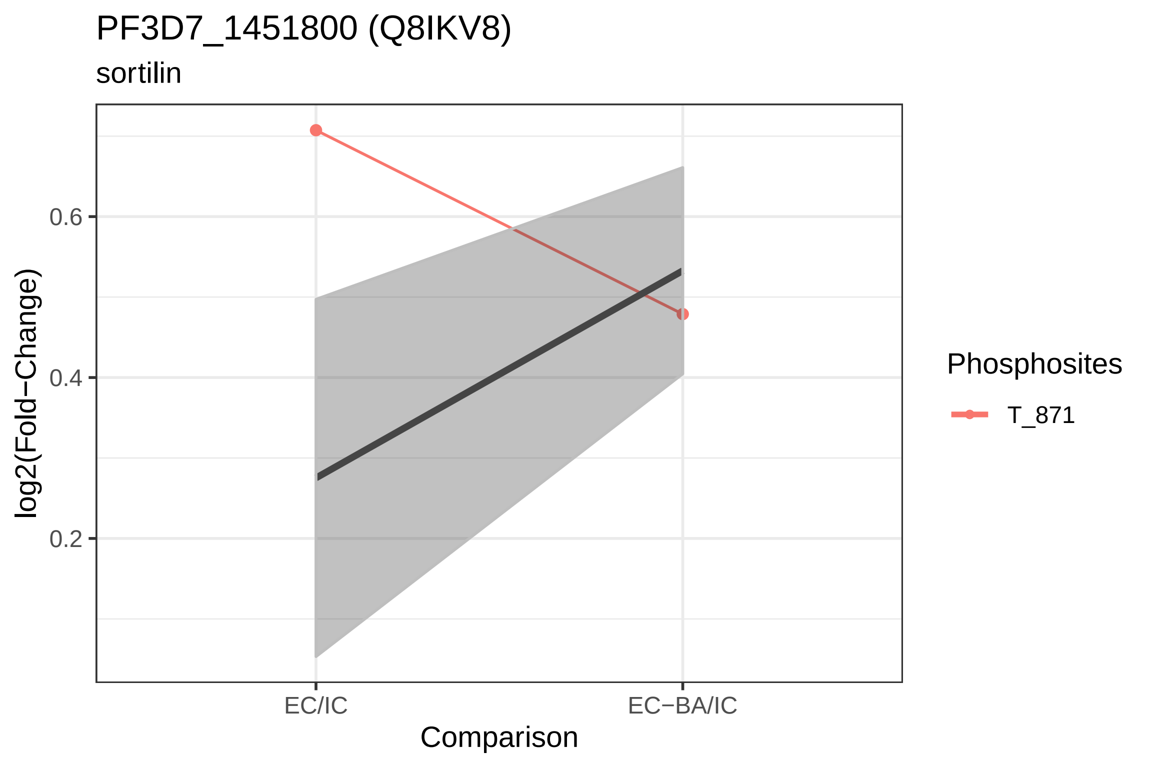

PF3D7_1451800 (Q8IKV8)
sortilin
log2(Fold−Change)
0.6
0.4
0.2
EC/IC
EC−BA/IC
Comparison
Phosphosites
T_871

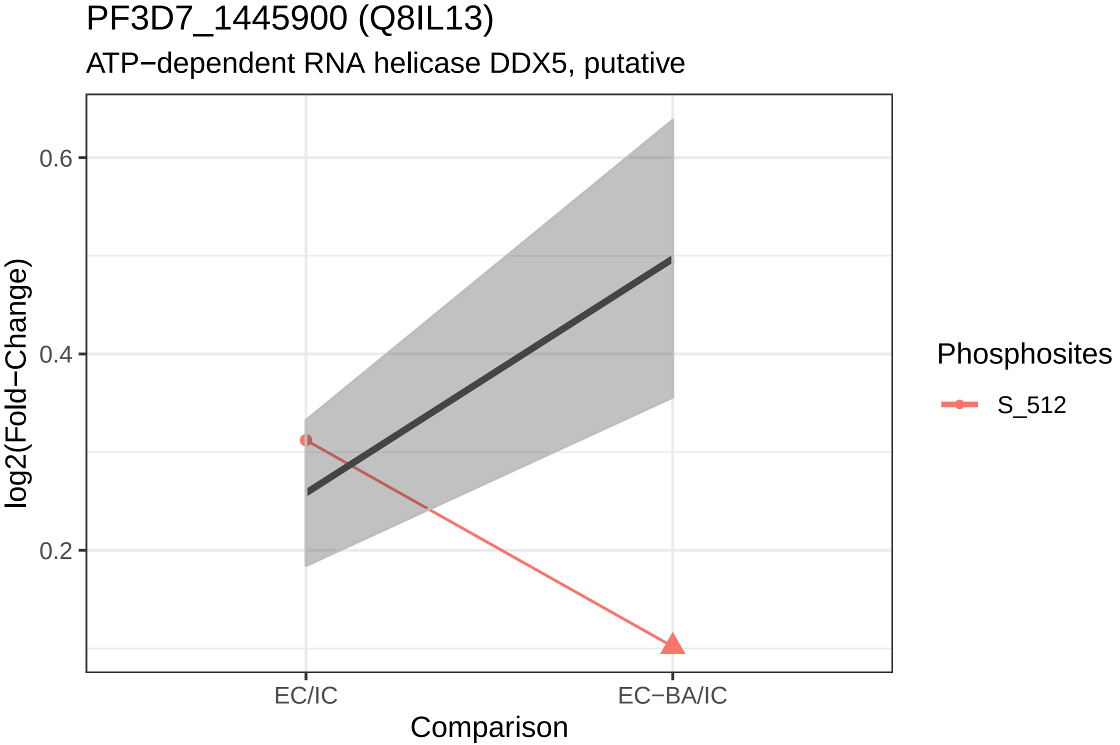
PF3D7_1445900 (Q8IL13)
ATP−dependent RNA helicase DDX5, putative
log2(Fold−Change)
0.6
0.4
0.2
EC/IC
EC−BA/IC
Comparison
Phosphosites
S_512

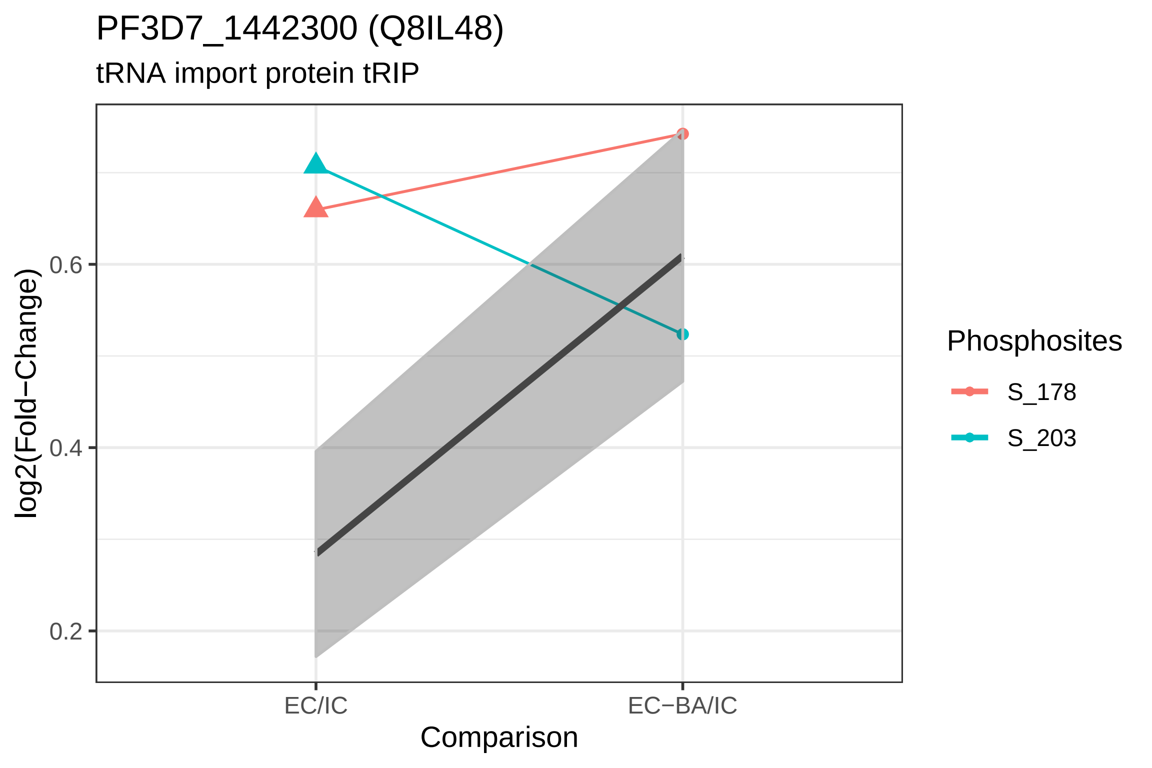

PF3D7_1442300 (Q8IL48)
tRNA import protein tRIP
log2(Fold−Change)
0.6
0.4
0.2
EC/IC
EC−BA/IC
Comparison
Phosphosites
S_178
S_203

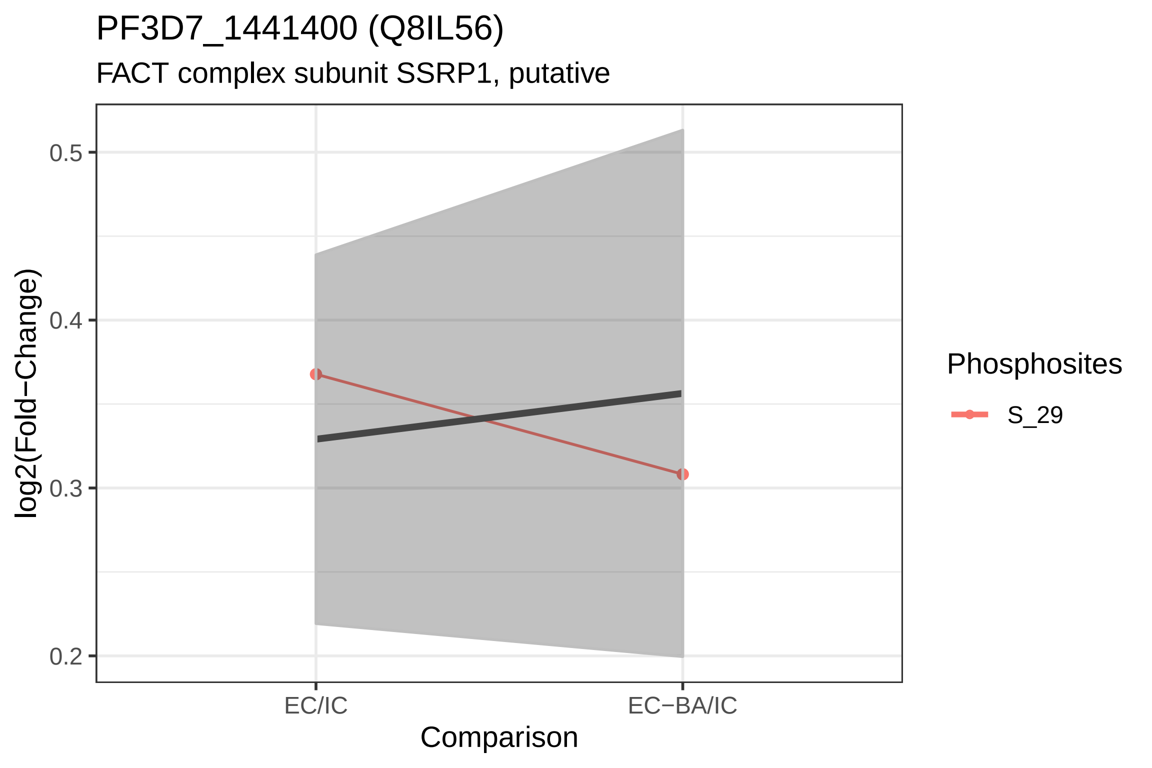
PF3D7_1441400 (Q8IL56)
FACT complex subunit SSRP1, putative
log2(Fold−Change)
0.5
0.4
0.3
0.2
EC/IC
EC−BA/IC
Comparison
Phosphosites
S_29

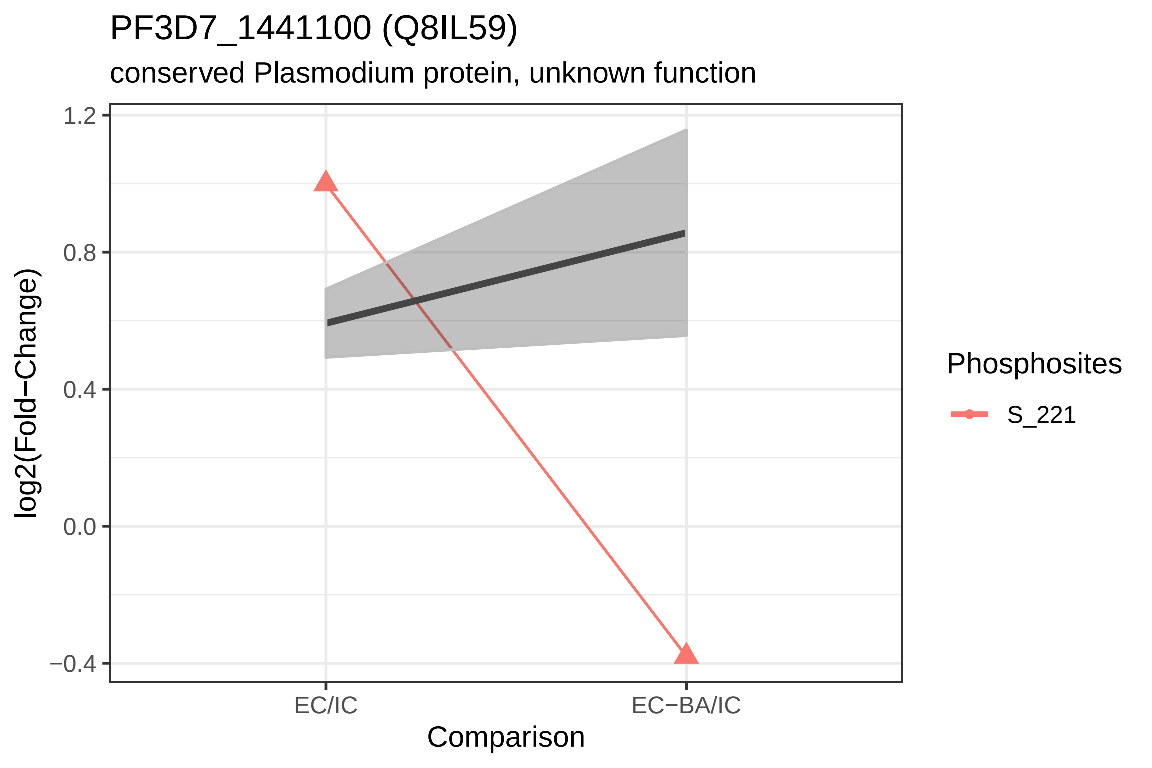
PF3D7_1441100 (Q8IL59)
conserved Plasmodium protein, unknown function
1.2
0.8
0.4
0.0
−0.4
log2(Fold−Change)
EC/IC
EC−BA/IC
Comparison
Phosphosites
S_221

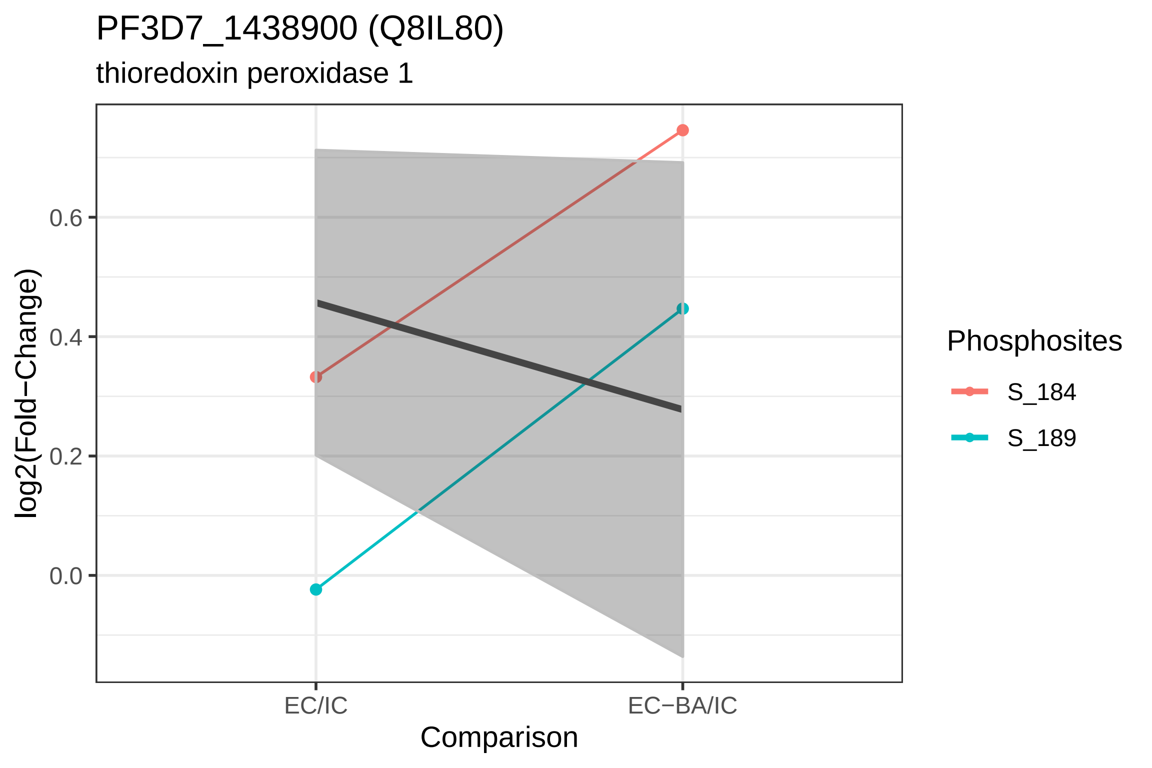

PF3D7_1438900 (Q8IL80)
thioredoxin peroxidase 1
log2(Fold−Change)
0.6
0.4
0.2
0.0
EC/IC
EC−BA/IC
Comparison
Phosphosites
S_184
S_189

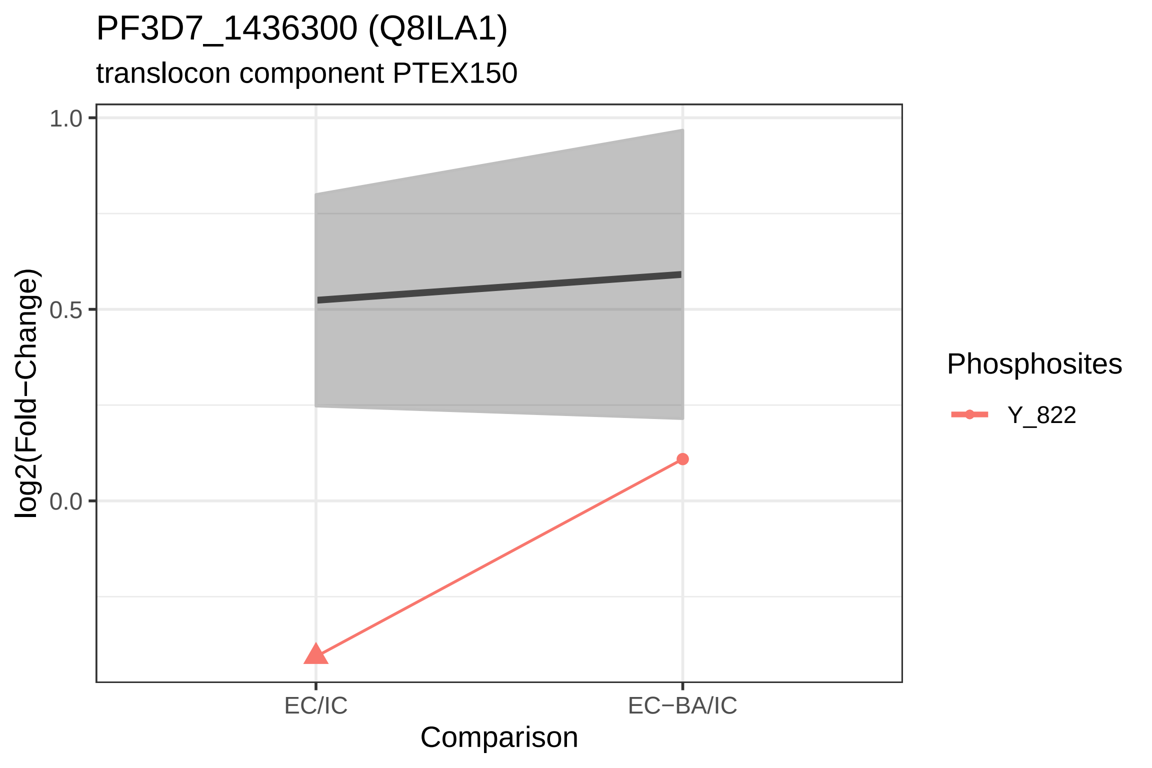
PF3D7_1436300 (Q8ILA1)
translocon component PTEX150
log2(Fold−Change)
1.0
0.5
0.0
EC/IC
EC−BA/IC
Comparison
Phosphosites
Y_822

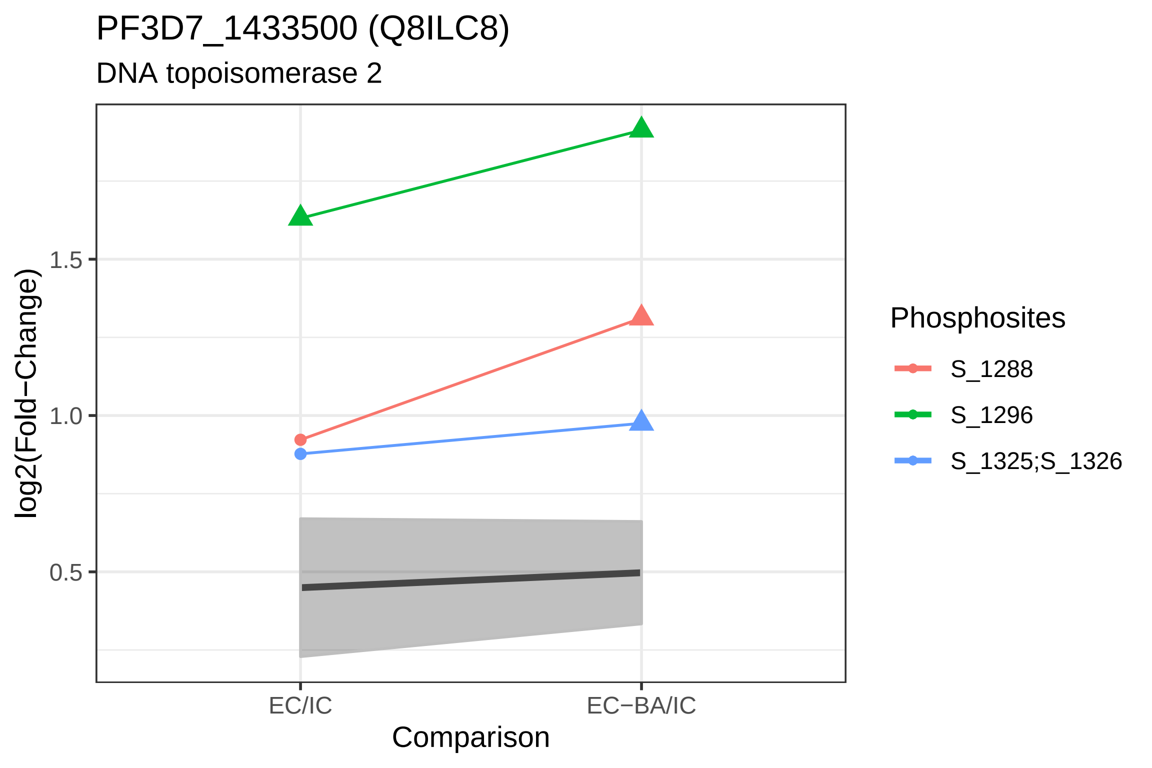

PF3D7_1433500 (Q8ILC8)
DNA topoisomerase 2
log2(Fold−Change)
1.5
1.0
0.5
EC/IC
EC−BA/IC
Comparison
Phosphosites
S_1288
S_1296
S_1325;S_1326

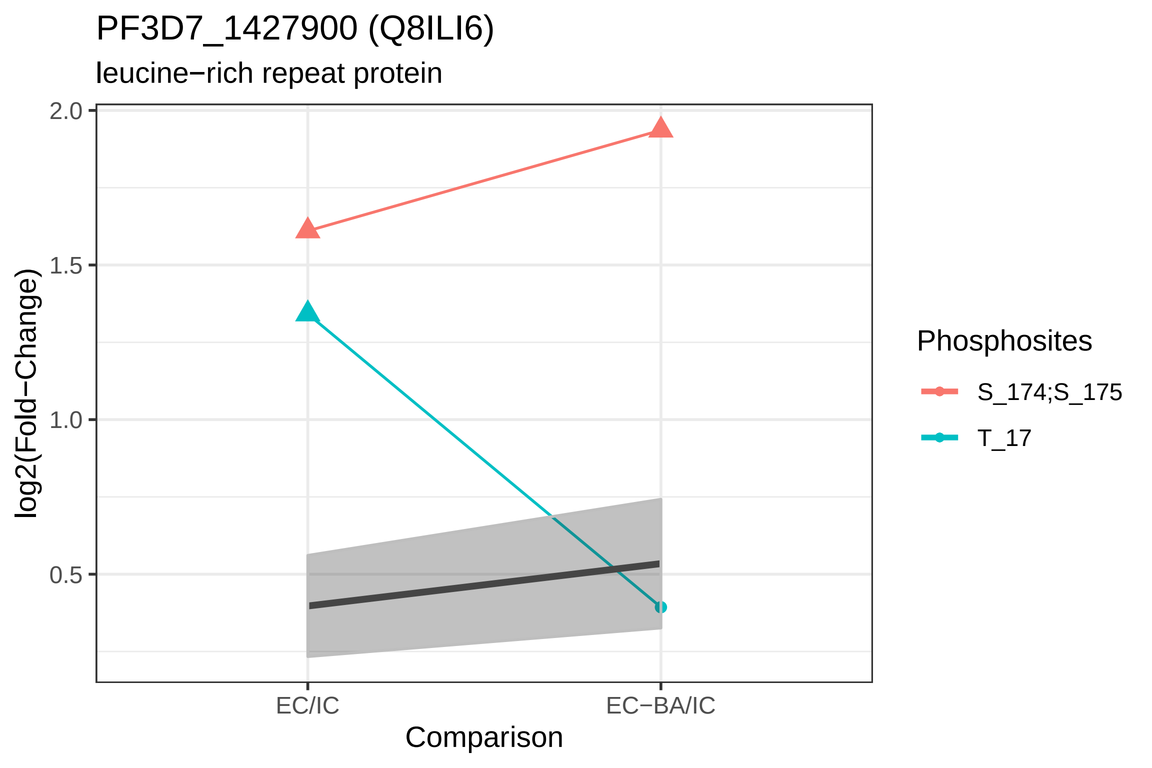

PF3D7_1427900 (Q8ILI6)
leucine−rich repeat protein
log2(Fold−Change)
2.0
1.5
1.0
0.5
EC/IC
EC−BA/IC
Comparison
Phosphosites
S_174;S_175
T_17

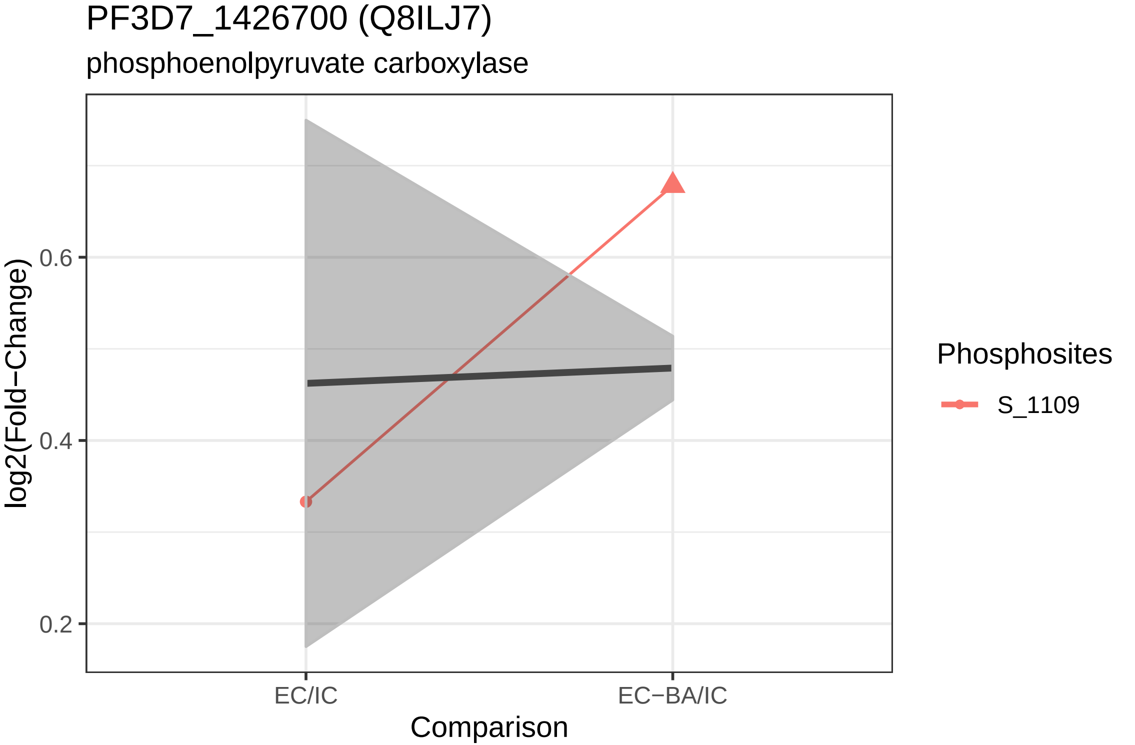
PF3D7_1426700 (Q8ILJ7)
phosphoenolpyruvate carboxylase
0.6
0.4
0.2
log2(Fold−Change)
EC/IC
EC−BA/IC
Comparison
Phosphosites
S_1109

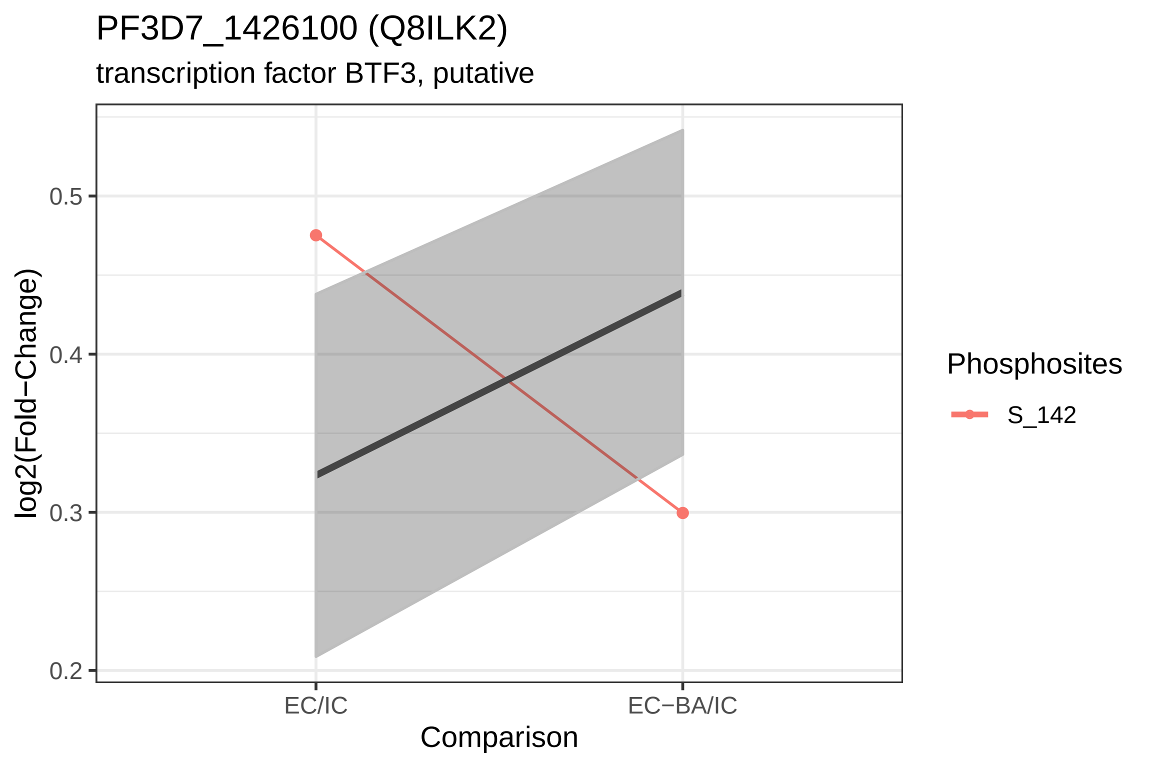
PF3D7_1426100 (Q8ILK2)
transcription factor BTF3, putative
log2(Fold−Change)
0.5
0.4
0.3
0.2
EC/IC
EC−BA/IC
Comparison
Phosphosites
S_142

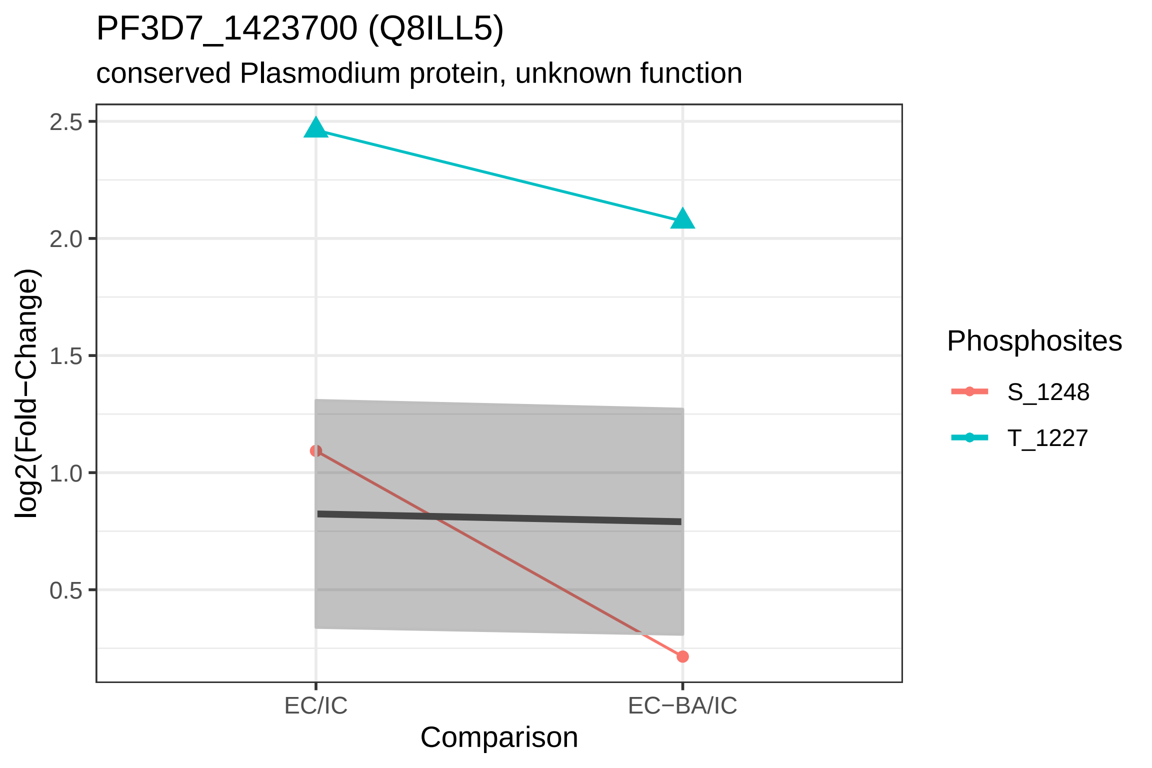
PF3D7_1423700 (Q8ILL5)
conserved Plasmodium protein, unknown function
log2(Fold−Change)
2.5
2.0
1.5
1.0
0.5
EC/IC
EC−BA/IC
Comparison
Phosphosites
S_1248
T_1227

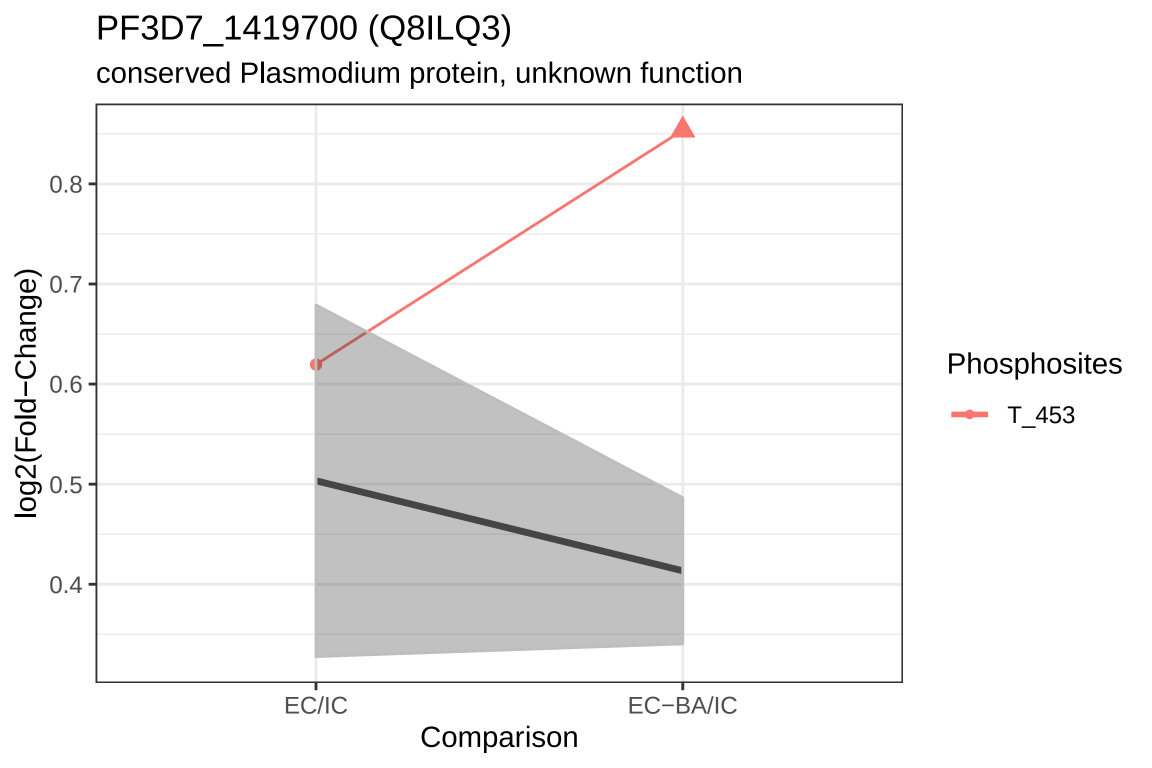

PF3D7_1419700 (Q8ILQ3)
conserved Plasmodium protein, unknown function
log2(Fold−Change)
0.8
0.7
0.6
0.5
0.4
EC/IC
EC−BA/IC
Comparison
Phosphosites
T_453

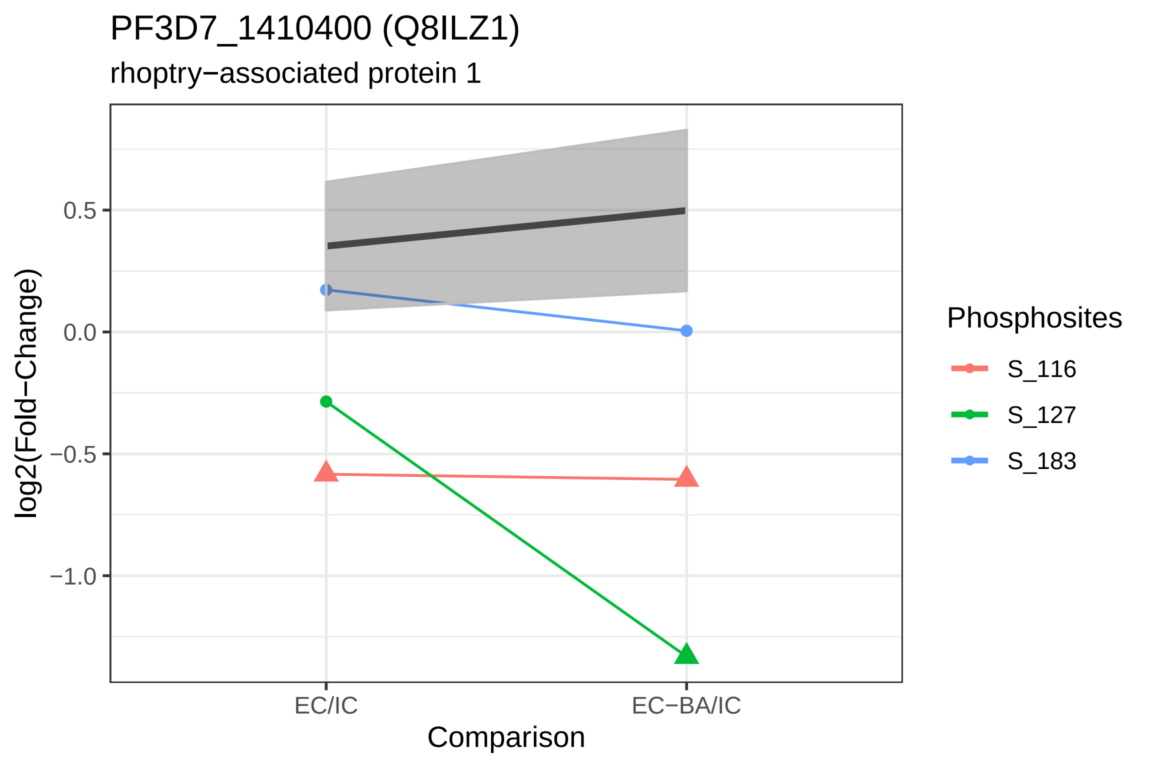

PF3D7_1410400 (Q8ILZ1)
rhoptry−associated protein 1
log2(Fold−Change)
0.5
0.0
−0.5
−1.0
EC/IC
EC−BA/IC
Comparison
Phosphosites
S_116
S_127
S_183

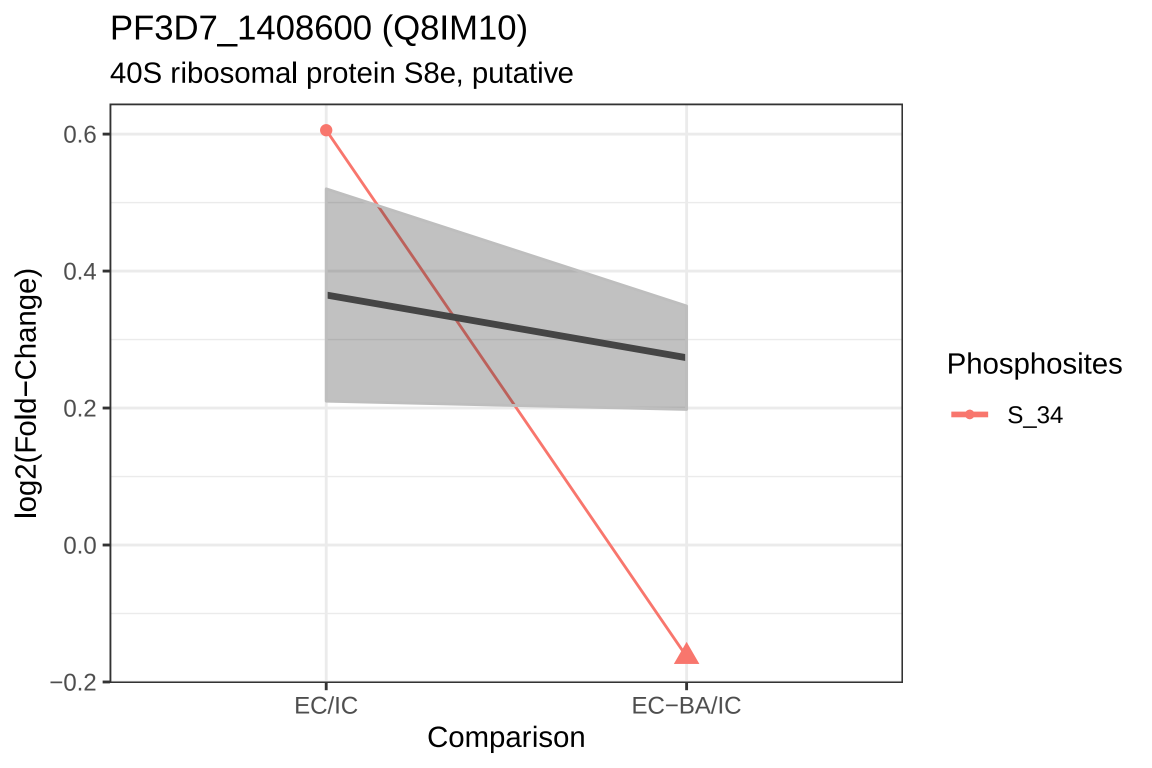

PF3D7_1408600 (Q8IM10)
40S ribosomal protein S8e, putative
log2(Fold−Change)
0.6
0.4
0.2
0.0
−0.2
EC/IC
EC−BA/IC
Comparison
Phosphosites
S_34

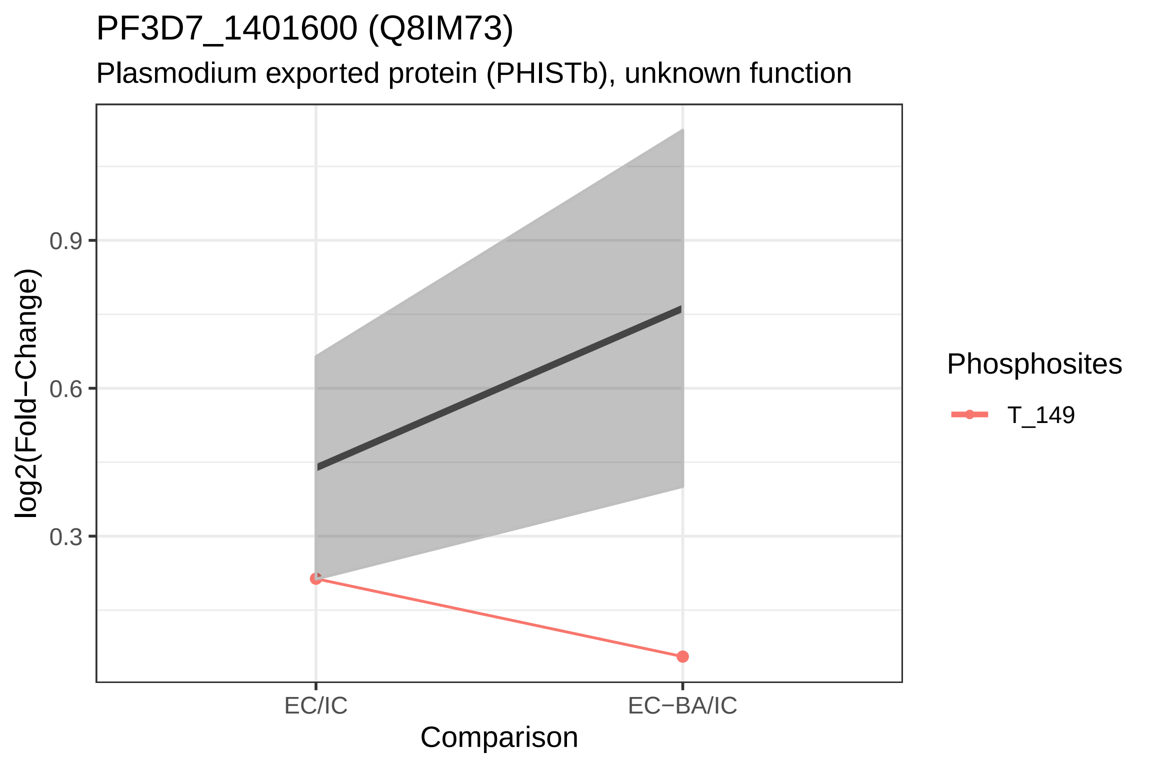
PF3D7_1401600 (Q8IM73)
Plasmodium exported protein (PHISTb), unknown function
log2(Fold−Change)
0.9
0.6
0.3
EC/IC
EC−BA/IC
Comparison
Phosphosites
T_149

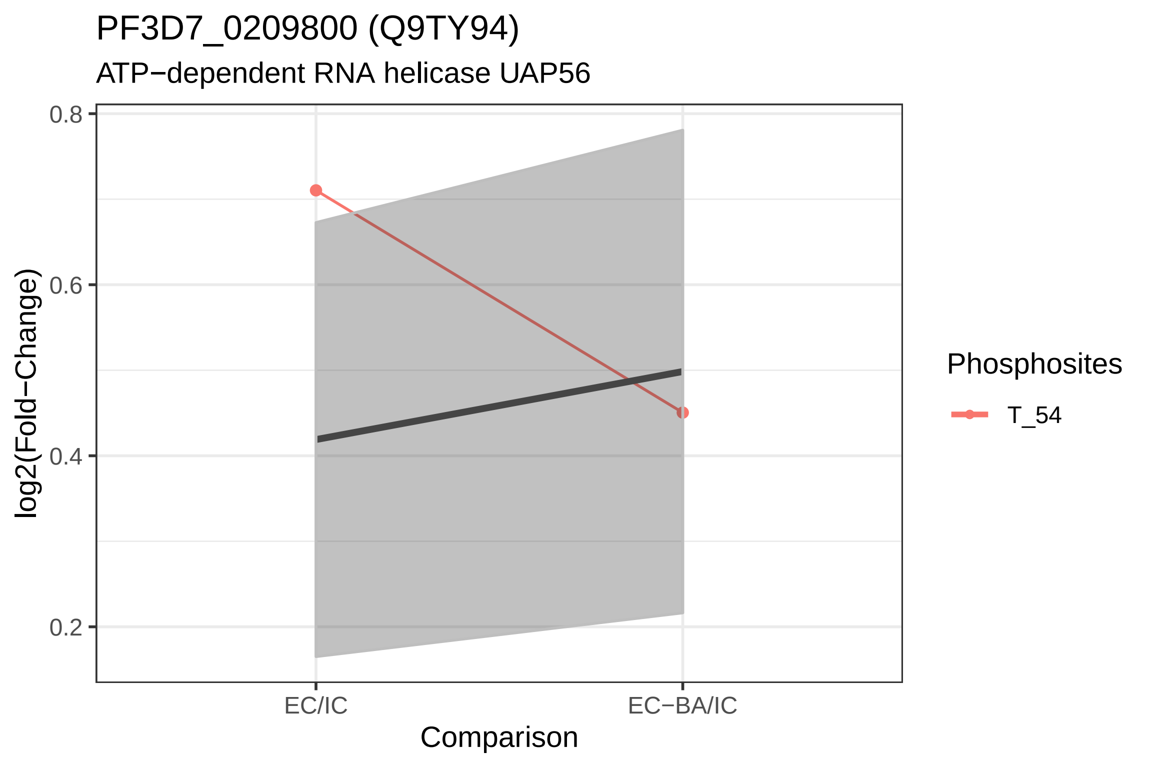

PF3D7_0209800 (Q9TY94)
ATP−dependent RNA helicase UAP56
log2(Fold−Change)
0.8
0.6
0.4
0.2
EC/IC
EC−BA/IC
Comparison
Phosphosites
T_54

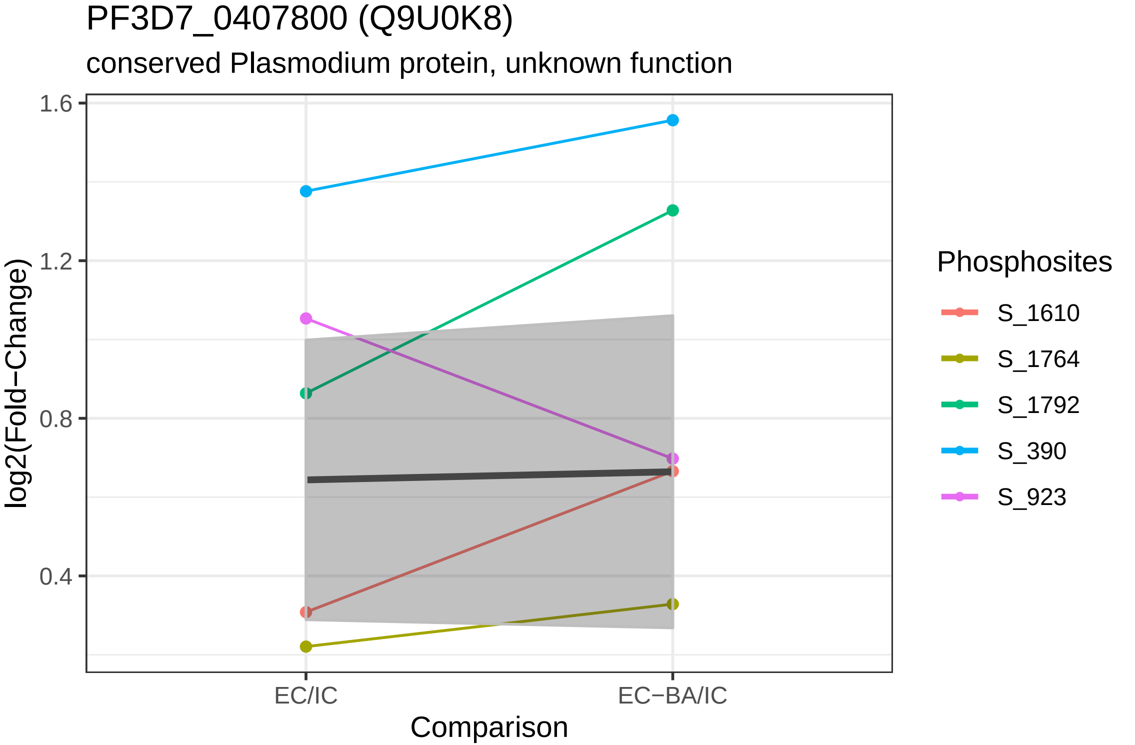

PF3D7_0407800 (Q9U0K8)
conserved Plasmodium protein, unknown function
log2(Fold−Change)
1.6
1.2
0.8
0.4
EC/IC
EC−BA/IC
Comparison
Phosphosites
S_1610
S_1764
S_1792
S_390
S_923
