# Supplementary material for: Phosphorylation-Dependent Assembly of a 14-3-3 Mediated Signaling Complex during Red Blood Cell Invasion by Plasmodium falciparum Merozoites
Source: mBio. 2020 Aug 18;11(4):e01287-20. doi: 10.1128/mBio.01287-20 (PMC7439480; doi:10.1128/mBio.01287-20)
Supplement: FIG S5 [file mBio.01287-20-sf005.pdf]

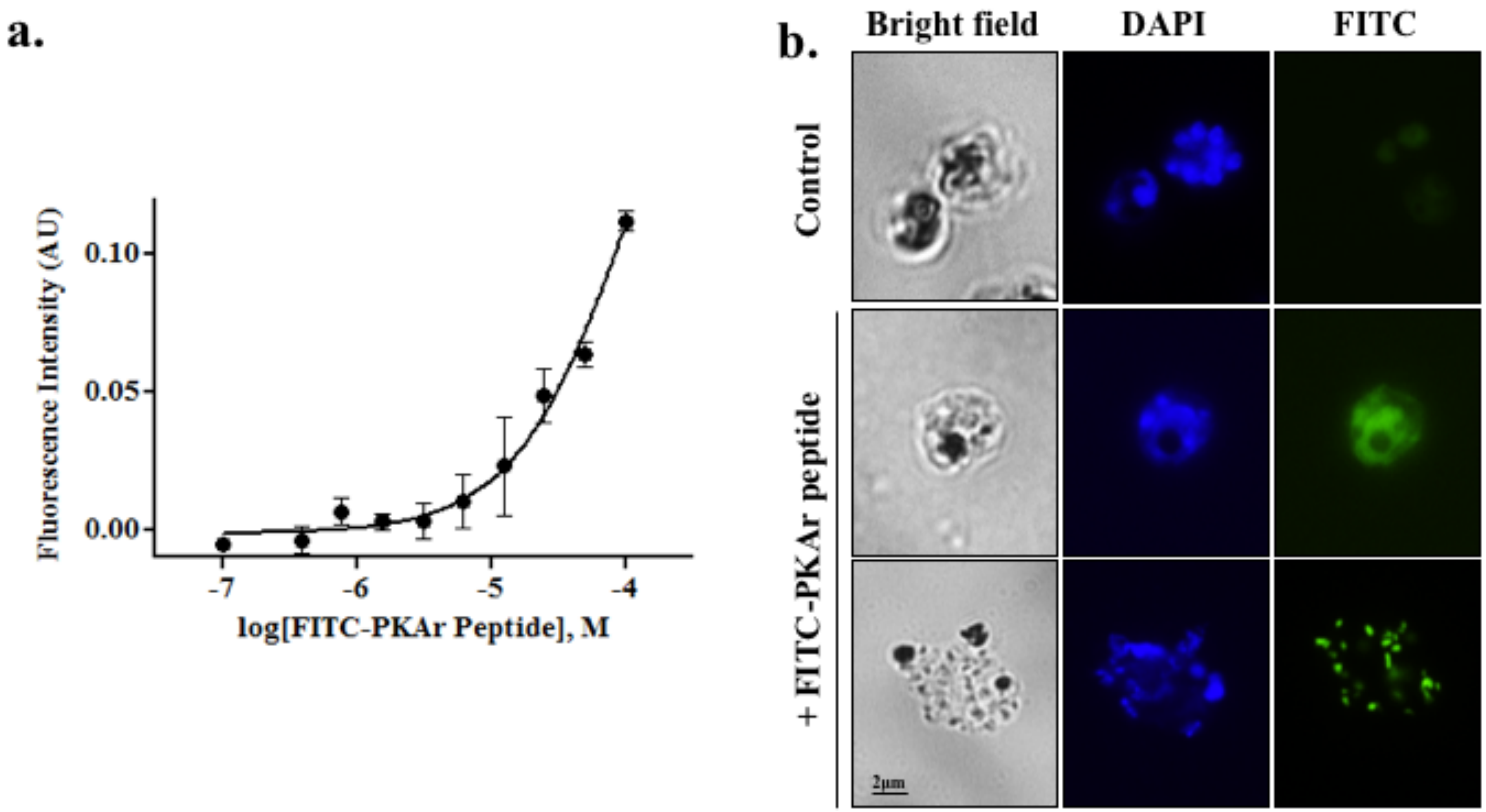

Figure S5. Entry of peptide P1 in *P. falciparum* schizonts and merozoites. Peptide P1, an 8 amino acid peptide based on PfPKAr sequence spanning Ser 113 and Ser 114 was conjugated to fluorophore FITC and incubated with *P. falciparum* cultures with late stage schizonts for 10 mins. Parasites were washed with RPMI1640 and uptake of Peptide P1-FITCwas measured by fluorimetry and observed by fluorescence microscopy. Significant uptake was observed in late stage schizonts and merozoites at concentrations above 25 μM.
